# Mean blood pressure according to the hypertension care cascade: Analysis of six national health surveys in Peru

**DOI:** 10.1016/j.lana.2021.100016

**Published:** 2021-07-13

**Authors:** Rodrigo M. Carrillo-Larco, Wilmer Cristobal Guzman-Vilca, Antonio Bernabe-Ortiz

**Affiliations:** a Department of Epidemiology and Biostatistics, School of Public Health, Imperial College London, London, UK; b CRONICAS Centre of Excellence in Chronic Diseases, Universidad Peruana Cayetano Heredia, Lima, Peru; c School of Medicine “Alberto Hurtado”, Universidad Peruana Cayetano Heredia, Lima, Peru; d Sociedad Científica de Estudiantes de Medicina Cayetano Heredia (SOCEMCH), Universidad Peruana Cayetano Heredia, Lima, Peru; e Universidad Científica del Sur, Lima, Peru

**Keywords:** Cardiovascular risk, Population health metrics, Cardio-metabolic risk factors

## Abstract

**Background:**

While we have good evidence about the hypertension care cascade, we do not know the mean blood pressure (BP) in these groups. We described the mean BP in four groups based on the hypertension care cascade at the national and sub-national levels in Peru.

**Methods:**

Descriptive analysis of six national health surveys. Blood pressure was measured twice and the second record herein analysed. We defined four groups: i) people with self-reported hypertension diagnosis receiving antihypertensive medication; ii) people with self-reported hypertension diagnosis not receiving antihypertensive medication; iii) people unaware they have hypertension with blood pressure ≥140 or 90 mmHg; and iv) otherwise healthy people.

**Findings:**

There were 125,066 people; mean age was 49.8 years and there were more women (51.7%). At the national level, in men and women and throughout the study period, we observed that the mean systolic BP (SBP) was the highest in people unaware they have hypertension; the mean SBP was similar between those with and without antihypertension medication, yet slightly higher in the former group. At the sub-national level, even though the mean SBP in the unaware group was usually the highest, there were some regions and years in which the mean SBP was the highest in the untreated and treated groups.

**Interpretation:**

These results complement the hypertension care cascade with a clinically relevant parameter: mean BP. The results point where policies may be needed to secure effective interventions to control hypertension in Peru, suggesting that improving early diagnosis and treatment coverage could be priorities.

**Funding:**

Wellcome Trust (214185/Z/18/Z).

**Research in context**

**Evidence before this study**

A literature search in PubMed (June 14^th^, 2021) with the following search strategy retrieved 5 original reports: "blood pressure" AND "hypertension care cascade". The little evidence on Latin America and the Caribbean (LAC), included in global endeavours, focused on the hypertension care cascade only. While this evidence revealed the proportion of people aware they have hypertension, how many people with hypertension are receiving medication and how many have achieved blood pressure control, we do not know the mean blood pressure level in these groups at the general population level. In other countries, for example in the US, a national survey reported higher mean systolic blood pressure in people with untreated hypertension than in their treated peers and those without hypertension. Overall, evidence leveraging on national estimates and elaborating above and beyond the steps of the hypertension care cascade is scarce.

**Added value of this study**

Following a descriptive approach, we summarised the mean systolic blood pressure (SBP) in four population groups using national health surveys in Peru between 2015 and 2020: people with self-reported hypertension diagnosis and receiving treatment; people with self-reported hypertension diagnosis not receiving treatment; people unaware they have hypertension, and otherwise healthy people. We delivered these estimates at the national and sub-national levels. The results suggest that the worst mean SBP was found in those unaware they have hypertension, followed by those with self-reported hypertension diagnosis receiving treatment closely followed by those with self-reported hypertension diagnosis not receiving medication. At the sub-national level there were heterogeneous profiles, whereby in some regions the highest means SBP was found in the untreated group yet in other regions the highest mean was observed in the treated group. Altogether, and accounting for the limitations of this analysis, these four population groups and their observed mean SBP would lead to three scenarios with clinical and public health implications. First, if the mean SBP were the highest in the unaware group, this would suggest that screening strategies need to be prioritized along with early diagnosis programs. Second, if the mean were the highest in the group of people with hypertension not receiving treatment, this would suggest that they need to strengthen treatment coverage. Third, if the mean SBP were the highest in the group of people with hypertension receiving treatment, then it would suggest that they need to reinforce treatment adherence or to revisit treatment schemes to secure effective treatment.

**Implications of all the available evidence**

This work complements the hypertension care cascade evidence with a clinically relevant parameter: mean SBP in the general population. This approach could be used in other countries and by global health researchers to provide further insights about the hypertension care cascade. In Peru, these results suggest that improving screening and early diagnosis is paramount, because the unaware group seems to have the highest mean SBP in the general population.

Alt-text: Unlabelled box

## 1 Introduction

There is global and local evidence about the hypertension care cascade (i.e., awareness, treatment and control), and how low- and middle-income countries still need to reach optimal levels of these metrics [1], [2], [3], [4], [5]. These hypertension care cascade estimates have informed population health indicators, including those for monitoring the health system response to non-communicable diseases [6]. However, although the hypertension care cascade has informed where awareness, treatment and control rates need improvement, these metrics do not inform about the underlying blood pressure profile in these groups. In other words, while the treatment cascade informs, for example, about the proportion of people with hypertension receiving antihypertensive treatment, it does not inform whether the mean blood pressure in this group is better or worse than in the general population or in those not receiving treatment. Understanding both -hypertension care cascade and the underlying blood pressure levels- could provide evidence to push policies to improve hypertension care. If in the general population we knew the proportion of people with hypertension receiving treatment, and the mean blood pressure were the highest in this group, it would suggest they need to secure effective treatment. Similarly, if we knew the proportion of people with hypertension not receiving antihypertensive treatment, and they had the highest mean blood pressure, then the priority could be on expanding treatment allocation.

In this work, following a descriptive approach, we complemented the hypertension care cascade information for Peru [4], and delivered mean blood pressure levels in four population groups: i) people with self-reported hypertension diagnosis receiving antihypertensive treatment; ii) people with self-reported hypertension diagnosis not receiving treatment; iii) people unaware they have hypertension; and iv) otherwise healthy people. These estimates will show where the priority should be on securing effective treatment, where the priority should be on expanding treatment allocation, and where the priority should be on early diagnosis. This evidence will strengthen the fight to improve hypertension care in Peru, while it could also provide complementary metrics to study in other countries and world regions.

## 2 Methods

### 2.1 Study design

We analysed six national health surveys in Peru, covering from 2015 to 2020. The National Demographic and Health Survey (ENDES for its name in Spanish) is conducted every year on a nationally representative sample of men and women. The ENDES surveys a representative sample at the national and regional levels. The ENDES collects socio-demographic and self-reported health information, along with anthropometrics and two blood pressure measurements.

### 2.2 Rationale

**Figure 1:**
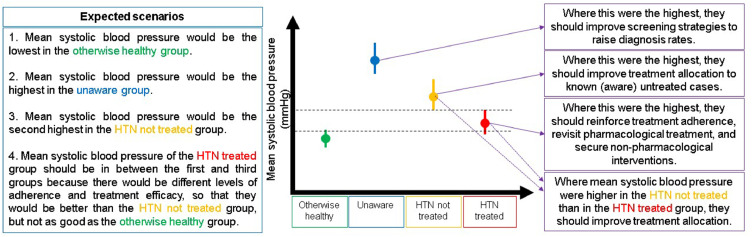
Description of the rationale and population groups. HTN: hypertension

Pragmatically, at the population level, there could be four groups: i) *people with self-reported hypertension diagnosis receiving treatment*; ii) *people with self-reported hypertension diagnosis not receiving treatment*; iii) *people unaware they have hypertension*; and iv) *otherwise healthy people* (Figure 1).

Ideally, the mean blood pressure in the otherwise healthy population would be the lowest. Conversely, the mean blood pressure in those unaware they have hypertension would be the highest. We would also anticipate that the mean blood pressure would be higher in the group of people with self-reported hypertension diagnosis not receiving treatment, than in their peers receiving antihypertensive medication (Figure 1).

These four population groups and their expected blood pressure means would lead to three scenarios with clinical and public health implications. First, if the mean blood pressure were the highest in the unaware group, this would suggest that screening strategies need to be prioritized along with early diagnosis programs. Second, if the mean were the highest in the group of people with hypertension not receiving treatment, this would suggest that they need to strengthen treatment coverage. Third, if the mean blood pressure were the highest in the group of people with hypertension receiving treatment, then it would suggest that they need to reinforce treatment adherence or to revisit treatment schemes to secure effective treatment (Figure 1).

### 2.3 Study population

We studied men and women aged 30 years and above because hypertension is seldom present in younger individuals and this is also consistent with the literature on the subject [1,3]. The ENDES collects two blood pressure measurements; we only used the second one. We excluded observations when the systolic blood pressure (SBP) was outside the range 70-270 mmHg, and the same decision was made for diastolic blood pressure (DBP) outside the range 30-150 mmHg as in previous reports [3,7]. We analysed a complete-case dataset regarding systolic and diastolic blood pressure, self-reported hypertension diagnosis and self-reported antihypertensive treatment.

### 2.4 Variables

To define the four groups, we used two questions. The first was about diagnosis of hypertension made by a health professional and reported by the participant: *at any point in your life, has a physician diagnosed you with hypertension* or *high blood pressure?* This question had three possible answers: *yes, no* and *do not know*; the last two options were collapsed, and this variable coded as *no* versus *yes*. The other question was about treatment for hypertension reported by the participant: *in the last twelve months, have you received or bought medicines to control your blood pressure?* This question had three possible answers: *yes, no* and *do not know*; the last two options were collapsed, and this variable coded as *no* versus *yes*. The other variables we used were study year, sex, age (<60 and 60+), and region; there are twenty-five regions in Peru.

The otherwise healthy group included people with blood pressure <140 and <90 mmHg, those who answered *no* to the self-reported diagnosis question and *no* to the self-reported antihypertensive treatment question. The unaware group included those who answered *no* to the self-reported diagnosis question and *no* to the self-reported antihypertensive treatment question, and their blood pressure was ≥140 or ≥90 mmHg. The group with hypertension not receiving medication included those who answered *yes* to the self-reported diagnosis question and *no* to the self-reported antihypertensive treatment question. The group with hypertension receiving medication included those who answered *yes* to the self-reported diagnosis question and *yes* to the self-reported antihypertensive treatment question.

### 2.5 Statistical analysis

We used R (version 3.6.1) and STATA (version 16.1, College Station, Texas 77845, USA). The analysis code and the datasets we analysed are available as supplementary materials. We cleaned and selected the relevant variables in each individual dataset and appended these together. Accounting for the complex survey design of the ENDES, we used descriptive statistics to summarize the mean SBP in each population group stratified by region, sex, age and survey year; the analysis was also conducted at the national level (i.e., not stratified by region). The mean SBP was computed along with the 95% Confidence Interval (95% CI). Results are also presented as the absolute differences (mmHg) between the unaware group and the other groups (e.g., mean SBP in the unaware group – mean SBP in the otherwise healthy population).

### 2.6 Ethics

The institutions who carried out the surveys were responsible for ethical clearance. For this analysis, we did not seek approval by an Institutional Review Board. We considered this wok as of minimal risk because we analysed de-identified surveys that are open access. The authors alone are responsible for the opinions in this work, and do not necessarily represent those of the institutions to which they belong. The authors are collectively responsible for the accuracy of the data and findings.

### Role of the funding source

The funder of the study had no role in study design, data collection, data analysis, data interpretation, or writing of the report. RMC-L and WCG-V had full access to the data in the study and vouch for the data accuracy. All authors collectively had final responsibility for the decision to submit for publication.

## 3 Results

### 3.1 Study population

Overall, the study population included 125,066 people, with a mean age of 49.8 (95% CI: 49.7-50.0) years; there was slightly more women (51.7%) than men (48.3%). The overall mean SBP was 123.6 (95% CI: 123.4-123.8) mmHg, and it was higher in men (127.5 (95% CI: 127.2-127.7) mmHg) than in women (120.0 (95% CI: 119.8-120.3) mmHg).

### 3.2 National estimates

Across the study years, the frequency of the four population groups was: otherwise healthy (74.6% (95% CI: 74.1%-75.0%)), unaware they have hypertension (12.2% (95% CI: 11.8%-12.6%)), self-reported hypertension diagnosis without treatment (3.7% (95% CI: 3.5%-3.8%)), and self-reported hypertension diagnosis with treatment (9.6% (95% CI: 9.3%-9.9%)). These metrics changed between 1% and 4% from 2015 to 2020 (Supplementary Table 1).

Overall, and following a descriptive approach, the mean SBP in the otherwise healthy group was 116.5 (95% CI: 116.3-116.6) mmHg, the mean SBP in the unaware group was 151.3 (95% CI: 150.9-151.7) mmHg, in the group with self-reported hypertension diagnosis without treatment was 131.4 (95% CI: 130.4-132.4) mmHg, and for people with self-reported hypertension diagnosis receiving treatment the mean SBP was 141.2 (95% CI: 140.4-141.9) mmHg. These numbers suggested that the largest difference was between the unaware and the otherwise healthy groups, with an absolute difference of 35 mmHg. A similar pattern was found for DBP: 70.6 (95% CI: 70.5-70.7) mmHg in the otherwise healthy group, 84.6 (95% CI: 84.3-84.9) mmHg in the unaware group, 76.1 (95% CI: 75.6-76.6) mmHg in the group with self-reported hypertension diagnosis without treatment, and 75.9 (95% CI: 75.5-76.3) mmHg in the group with self-reported hypertension diagnosis with treatment.

**Figure 2:**
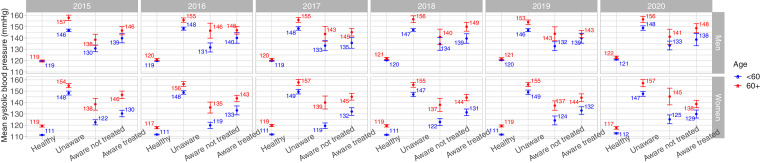
Mean systolic blood pressure by population groups stratified by sex, age group and year

The mean SBP in the otherwise healthy group appeared to always be the lowest (Figure 2; Supplementary Table 2). In the otherwise healthy women, the mean SBP was higher in those aged 60+ years than in their younger counterparts; this pattern was not observed in men, amongst whom there were no substantial differences between the two age groups in the otherwise heathy populations as suggested by the overlapping confidence intervals. Conversely, across study years for both men and women and regardless of the age group, the highest mean SBP appeared to be in the unaware group; this mean SBP was even higher in those aged 60+ years as suggested by the never overlapping confidence intervals. A similar array was observed for DBP (Supplementary Figure 1 and Supplementary Table 4).

The largest SBP difference seemed to be between the unaware and the otherwise healthy groups. The largest SBP difference in men aged <60 years was observed in 2016 and 2017 (29 mmHg); the largest difference in older (60+ years) men was observed in 2015 (38 mmHg). In women aged <60 years, the largest SBP absolute difference was found in 2017 and 2019 (38 mmHg); the largest difference in women aged 60+ years was observed in 2020 (40 mmHg). Results for DBP showed a similar profile (Supplementary Figure 1 and Supplementary Table 4).

### 3.3 Sub-national estimates

**Figure 3:**
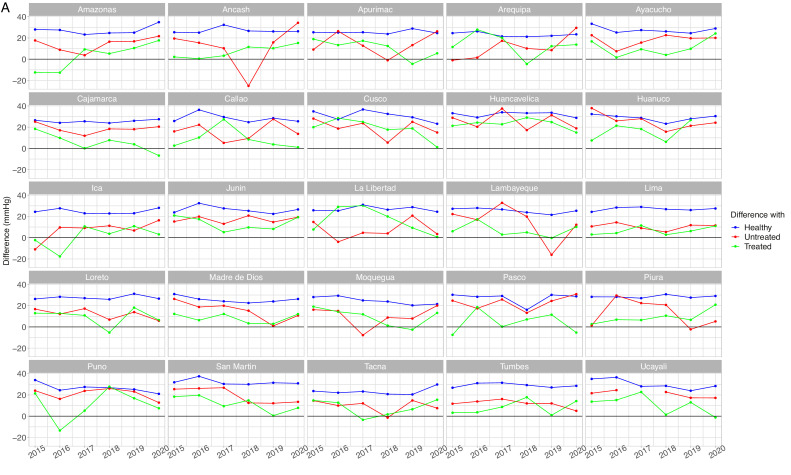

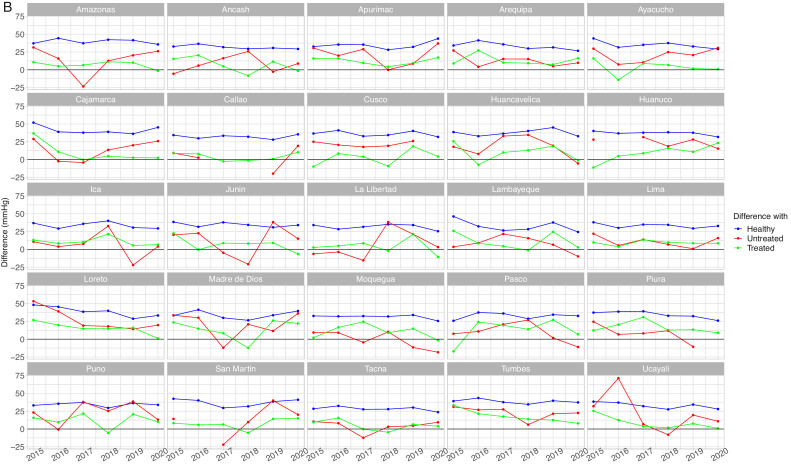
Mean systolic blood pressure differences by population group by year in men aged <60 years (A) and 60+ years (B) Absolute difference in relation to the unaware group (e.g., mean SBP in the unaware group – mean SBP in the otherwise healthy group). The unaware group was chosen as the reference because it most often showed the highest mean SBP. A small difference is not desirable because it would suggest it is close to the worst performing group (i.e., unaware group); similarly, a negative difference is not desirable because it would suggest it is above the worst performing group. Conversely, a positive large difference is desirable because it would suggest it is far apart from the worst performing group and thus closer to optimal SBP. SBP: systolic blood pressure.

**Figure 4:**
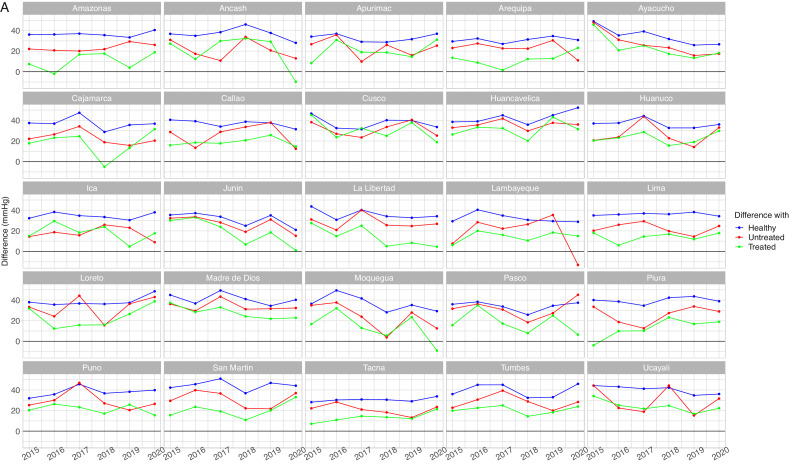

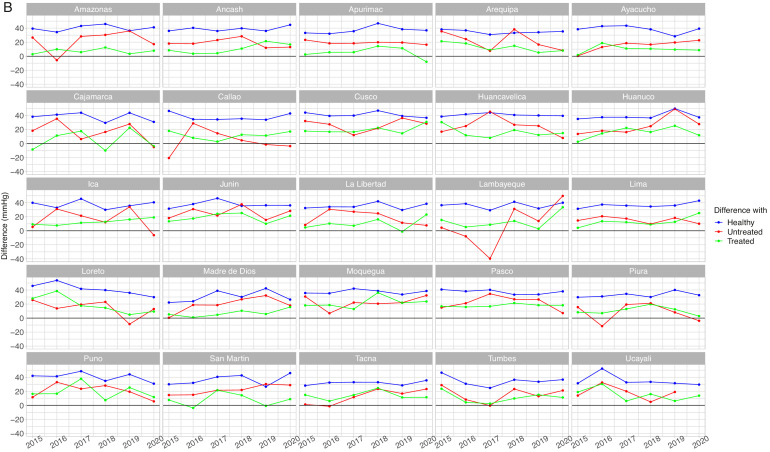
Mean systolic blood pressure differences by population group by year in women aged <60 years (A) and 60+ years (B) Absolute difference in relation to the unaware group (e.g., mean SBP in the unaware group – mean SBP un the otherwise healthy group). The unaware group was chosen as the reference because it most often showed the highest mean SBP. A small difference is not desirable because it would suggest it is close to the worst performing group (i.e., unaware group); similarly, a negative difference is not desirable because it would suggest it is above the worst performing group. Conversely, a positive large difference is desirable because it would suggest it is far apart from the worst performing group and thus closer to optimal SBP. SBP: systolic blood pressure.

The mean SBP in the unaware group was not always the highest across regions and years (Supplementary Table 3, Supplementary Figure 2 and Supplementary Figure 3). This is expressed by some negative differences between the mean SBP in the unaware group and the mean SBP in other groups (Figure 3A, Figure 3B, Figure 4A and Figure 4B). A similar scenario was found for DBP (Supplementary Table 4, Supplementary Figure 4 and Supplementary Figure 5)

In men aged <60 years, in 9 region-year points we observed that the difference with respect to the unaware group was negative for those with self-reported hypertension diagnosis without treatment (Figure 3A); in 15 region-year points the difference was negative for those with self-reported hypertension diagnosis receiving treatment. In men aged 60+ years, in 23 region-year points the difference was negative in the untreated group, whereas in 21 region-years the difference was negative in the treated group (Figure 3B). Therefore, at some region-year points, the mean SBP in the untreated group was the highest; also, in some region-year points, the mean SBP in the treated group was the highest. Results for DBP are shown in Supplementary Tables 4 and 5, and in Supplementary Figures 4 and 5.

In women aged <60 years, and with respect to the unaware group, in 5 region-year points we observed that the difference was negative for those with self-reported hypertension diagnosis with treatment group (Figure 4A). In women aged 60+ years, in 13 region-year points the difference was negative in the untreated group, whereas in 7 region-years the difference was negative in the treated group (Figure 4B). Therefore, at some region-year points in women aged <60 years, the mean SBP in the treated group was the highest; also, at some region-year points in women aged 60+ years, the mean SBP was the highest in the untreated group or in the treated group. Results for DBP are shown in Supplementary Tables 4 and 5, and in Supplementary Figures 4 and 5.

## 4 Discussion

### 4.1 Main findings

In this descriptive analysis of six (2015-2020) national health surveys in Peru, we studied mean blood pressure in four population groups: people with self-reported hypertension diagnosis and receiving treatment, people with self-reported hypertension diagnosis not receiving treatment, people unaware they have hypertension, and otherwise healthy people. At the national level we observed that the highest mean blood pressure seemed to always be in the unaware group, followed by the treated and untreated groups though with little difference between these two. These results suggest that, at the national level, the priority should be on screening and other early diagnosis strategies. At the sub-national level there was a heterogeneous profile. Although the mean SBP in the unaware group appeared to usually be the highest, in some years and regions, the other groups had a higher mean SBP. The unaware group was most often displaced by those with self-reported hypertension diagnosis receiving treatment, followed by those not receiving treatment. The results at the sub-national level would call to: i) strengthen screening and early diagnosis strategies; ii) enhance (effective) treatment allocation to reduce the hypertension group without treatment and their mean SBP; and iii) secure continuous effective treatment to reduce or maintain low mean blood pressure in those with hypertension receiving treatment.

### 4.2 Public health implications

The results at the national and sub-national level would alert that the most pressing problem is the population group unaware they have hypertension, amongst whom the mean blood pressure appeared to be the highest. As Peru moves towards universal health coverage [8], it is important that they secure strong screening and early diagnosis opportunities for key diseases like hypertension [2]. Universal health checks, particularly for those at higher risk (e.g., older people, obese, family history of hypertension), should be a cornerstone in primary prevention to reduce the unaware group and the mean SBP amongst them.

At the national level, our results suggested that people with self-reported hypertension diagnosis not receiving treatment appeared to have an average SBP lower than that of their peers receiving antihypertensive medication. Although largely speculative and warranting further verification, perhaps the former group have improved health-related behaviours (e.g., decreased consumption of salt and do more physical activity).

This work also suggests that, in some regions and years, the mean SBP appeared to be the highest among those with self-reported hypertension diagnosis not receiving treatment. This finding points out that effective treatment coverage needs to be expanded. Health professionals with adequate training [9,10], and also lay personnel (e.g., community health workers) [11], [12], [13], could unload the burden on doctors and even have a better outreach in the community securing that treatment reaches all patients. Health authorities should also improve structural barriers. For example, if commuting to the healthcare centre is a barrier to seek care and receive treatment [14], other options must be considered like tele-medicine along with remote devices [15], and community visits.

More often did we observe that the mean SBP would be highest in those with self-reported hypertension diagnosis receiving treatment, yet this happened in some regions and years. There could be some potential explanations for this. First, our results could have been influenced by recall or desirability bias; in other words, more people reported to have been receiving treatment than the real number. Second, in our definition of antihypertensive treatment we did not include any details about the treatment scheme or adherence. Even though they could have been receiving treatment, it is possible that this was not being taken as prescribed [16]. We would therefore suggest improving treatment adherence and, if needed, to revise local treatment schemes. There are multiple interventions that could improve treatment adherence [17], [18], [19], including mHealth technologies [20], interventions specific for some population groups (e.g., minorities) [21], and self-monitoring [22]. Which of these interventions would give the best results in Peru, is unknown but deservers either careful consideration to choose the most suitable option, or empirical research whereby these interventions are implemented and comprehensively evaluated. In any case, providing effective treatment should be a priority for general physicians, family doctors and cardiologists, along with authorities at local organizations (e.g., Regional Directorates of Health, Ministry of Health, among others). Collective work with national and local authorities, as well as practitioners and other healthcare professionals [9,10], is warranted to find the best solutions. Patients should also be involved to incorporate their needs and concerns [23].

Addressing the hypertension burden for each region would require tailored interventions; even within one region, different strategies may be warranted. First, regions have different revenues and income sources; consequently, they have different budgets to spend on health matters. Their health needs would include more conditions than just hypertension, some of which may be more urgent depending on the region (e.g., infectious diseases in the Amazon) and time of year (e.g., respiratory diseases during winter in the Highlands). In this line, resources for strategic interventions need to prioritize early hypertension diagnosis. Unfortunately, many regions have indicators to improve self-reported hypertension diagnosis (Supplementary Table 6). This would imply a passive process in which people should have contact with the healthcare system to be diagnosed. Large-scale screening strategies or periodic check-up programs could further reduce the unawareness rate and the high blood pressure in this group. As we discussed above, non-health professionals could provide valuable aid to the healthcare system. In one region it was identified that the lack of human resources was a strong reason for limited antihypertensive treatment coverage (Supplementary Table 6). Second, cultural background matters; understanding that some diseases may cause damage “silently”, may be new in places where infectious diseases (with faster manifestation) had been highly prevalent.

In addition to the mean estimates on which we have reported, the dispersion around these means should be considered. For example, at the national level in 2019 the mean SBP for people aged <60 years receiving antihypertensive treatment was below 140 mmHg, yet the 95% CI would go over 140 mmHg. This implies there is a nontrivial proportion of people with self-reported hypertension diagnosis and receiving treatment who would still have a mean SBP >140 mmHg. The means and their associated dispersions should be understood together when interpreting the results to inform policies and priorities. We would consider that the second priority should be on improving effective and continuous treatment so that the mean is (well) bellow 140 mmHg in those receiving antihypertensive medication.

### 4.3 Strengths and limitations

We analysed 2015-2020 national health surveys in an upper-middle-income country in Latin America. We therefore provided recent evidence to inform current and future policies and interventions to improve hypertension surveillance. We expanded the local and global evidence about the hypertension care cascade, with mean levels of SBP. In so doing, we complemented the treatment cascade metrics with a clinically relevant parameter at the population level.

There are limitations we acknowledge. First, our blood pressure measurements were based on only one record, the second of two measurements. Because we only used the second measurement, our mean blood pressure could be biased towards higher values. If so, then our mean blood pressure estimates are probably showing higher values. Nonetheless, the trends and many differences are consistent enough to substantially change if we had averaged two out of three blood pressure records. Second, the information about antihypertensive treatment was based on one question asking whether the participant received any pharmacological treatment in the last 12 months. That is, we did not have any additional information on, for example, type of medicine and dose. Although this information could have provided more insight about the antihypertensive treatment scheme, and could have been used to define more specific groups, we argue that our approach is simple and pragmatic and could be incorporated as an additional metric for the surveillance of the health system response to improve the hypertension care. This approach did not aim for precision, but for a general idea to complement the hypertension care cascade with a clinically relevant metric at the population level. In this line, we focused on pharmacological treatment alone, even though there could be non-pharmacological strategies [24], [25], [26]. Non-pharmacological interventions warrant additional attention so that they reduce blood pressure levels in the general population. Together with the question about antihypertensive treatment, history of hypertension diagnosis was also reported by the participant. Self-reporting, recall and desirability bias should be considered in a careful interpretation of the results. Third, we cannot rule out the possibility that some individuals were included in more than one survey year; this has not been documented and we believe it would be a highly unlikely scenario given that this is a random national sample. Finally, the ENDES is only representative at the national and regional levels, though Peru has three main geo-political levels (region > province > district). Where possible, our results should be replicated with greater geo-political granularity, so that interventions are delivered where precisely needed. Fourth, we undertook a descriptive analysis whereby claims of differences between groups were based on the fact that 95% CI did not overlap. The results and conclusions should be interpreted in line with the study design and other limitations of this work.

### 4.4 Conclusions

Consistently across study years, sex, and age groups, both at the national and sub-national levels, mean SBP appeared to be highest in the unaware group; this group was followed by the aware and treated group seconded by the aware untreated group. In Peru, improving hypertension screening and early diagnosis strategies seem to be a priority. Securing effective treatment is also urgent in some regions, where the results suggested that those receiving antihypertensive medication still had high mean blood pressure. In some regions, expanding treatment coverage would be needed to reach those with hypertension not receiving medication, and to reduce the mean blood pressure in this group. Hypertension requires solid clinical management and strategic policies to reduce the burden of the adverse outcomes associated with it (e.g., cardiovascular events).

## Contributors

RMC-L conceived the idea, conducted the analysis and wrote the first draft of the manuscript. WCG-V conducted the analysis and provided critical input. AB-O edited and provided critical input to improve the manuscript. All authors approved the submitted version.

## Declaration of interests

None.

## Appendix Supplementary materials

Image, application 1

Image, application 2
