# Supplementary material for: Mean blood pressure according to the hypertension care cascade: Analysis of six national health surveys in Peru
Source: Lancet Reg Health Am. 2021 Jul 13;1:100016. doi: 10.1016/j.lana.2021.100016 (PMC8442255; doi:10.1016/j.lana.2021.100016)
Supplement: Supplementary file 2 [file mmc2.pdf]

Mean blood pressure according to the hypertension care cascade: Analysis of six national health surveys in Peru

Supplementary Table 1. Proportion (95% confidence intervals) of each population group by year 2

Supplementary Table 2. At the national level, mean systolic blood pressure (95% confidence intervals) by population group stratified by sex and study year 3

Supplementary Table 3. At the sub-national level, mean systolic blood pressure (95% confidence intervals) by population group stratified by sex and study year 6

Supplementary Table 4. At the national level, mean diastolic blood pressure (95% confidence intervals) by population group stratified by sex and study year 89

Supplementary Table 5. At the sub-national level, mean diastolic blood pressure (95% confidence intervals) by population group stratified by sex and study year 92

Supplementary Table 6. Official documents from each region in Peru addressing hypertension and brief discussion of these 175

Supplementary Figure 1. Mean diastolic blood pressure by population groups stratified by sex, age group and year 178

Supplementary Figure 2. Mean systolic blood pressure (95% confidence interval) by population group, across regions and study years in men 179

Supplementary Figure 3. Mean systolic blood pressure (95% confidence interval) by population group, across regions and study years in women 180

Supplementary Figure 4. Mean diastolic blood pressure (95% confidence interval) by population group, across regions and study years in men 181

Supplementary Figure 5. Mean diastolic blood pressure (95% confidence interval) by population group, across regions and study years in women 182

Supplementary Table 1. Proportion (95% confidence intervals) of each population group by year

	Otherwise healthy	Unaware	Hypertension without treatment	Hypertension with treatment
2015	76% (75%-77%)	11% (10%-11%)	4% (4%-5%)	10% (9%-10%)
2016	76% (75%-77%)	11% (11%-12%)	4% (3%-4%)	9% (8%-10%)
2017	76% (74%-77%)	12% (12%-13%)	4% (3%-4%)	9% (8%-9%)
2018	74% (73%-75%)	13% (12%-14%)	4% (3%-4%)	10% (9%-10%)
2019	74% (73%-75%)	12% (11%-13%)	4% (3%-4%)	11% (10%-12%)
2020	71% (70%-73%)	15% (14%-16%)	3% (3%-4%)	11% (10%-12%)

Supplementary Table 2. At the national level, mean systolic blood pressure (95% confidence intervals) by population group stratified by sex and study year

Year	Sex	Age	Categories	Mean SBP	Lower limit	Upper limit
2015	Men	<60	Healthy	119.6	119.2	120.0
2015	Men	<60	Unaware	146.7	145.5	147.8
2015	Men	<60	Aware not treated	130.8	127.9	133.8
2015	Men	<60	Aware treated	139.8	135.7	143.8
2015	Women	<60	Healthy	111.3	111.0	111.7
2015	Women	<60	Unaware	148.3	146.6	150.0
2015	Women	<60	Aware not treated	122.5	120.2	124.8
2015	Women	<60	Aware treated	130.3	127.4	133.2
2015	Men	60+	Healthy	119.7	118.8	120.6
2015	Men	60+	Unaware	157.8	155.4	160.1
2015	Men	60+	Aware not treated	138.4	133.5	143.3
2015	Men	60+	Aware treated	146.5	142.7	150.3
2015	Women	60+	Healthy	119.3	118.3	120.2
2015	Women	60+	Unaware	154.8	153.0	156.7
2015	Women	60+	Aware not treated	138.5	133.3	143.7
2015	Women	60+	Aware treated	146.8	143.5	150.0
2016	Men	<60	Healthy	119.8	119.4	120.1
2016	Men	<60	Unaware	148.2	146.9	149.6
2016	Men	<60	Aware not treated	131.6	127.7	135.6
2016	Men	<60	Aware treated	140.0	135.4	144.6
2016	Women	<60	Healthy	111.8	111.4	112.2
2016	Women	<60	Unaware	148.8	147.3	150.3
2016	Women	<60	Aware not treated	119.9	117.1	122.6
2016	Women	<60	Aware treated	133.0	129.1	137.0
2016	Men	60+	Healthy	121.0	120.1	121.8
2016	Men	60+	Unaware	155.7	153.8	157.6
2016	Men	60+	Aware not treated	146.4	139.8	153.0
2016	Men	60+	Aware treated	146.8	143.4	150.2
2016	Women	60+	Healthy	117.9	116.9	118.9
2016	Women	60+	Unaware	156.3	154.2	158.3
2016	Women	60+	Aware not treated	135.6	130.7	140.4
2016	Women	60+	Aware treated	143.7	140.9	146.4
2017	Men	<60	Healthy	119.9	119.5	120.3
2017	Men	<60	Unaware	148.4	146.8	149.9
2017	Men	<60	Aware not treated	133.2	128.7	137.8
2017	Men	<60	Aware treated	135.6	131.0	140.3
2017	Women	<60	Healthy	111.8	111.4	112.2
2017	Women	<60	Unaware	149.5	147.5	151.5

2017	Women	<60	Aware not treated	119.5	117.0	122.0
2017	Women	<60	Aware treated	132.0	128.7	135.4
2017	Men	60+	Healthy	120.9	119.8	122.0
2017	Men	60+	Unaware	155.9	153.7	158.1
2017	Men	60+	Aware not treated	143.5	136.8	150.2
2017	Men	60+	Aware treated	145.1	141.7	148.4
2017	Women	60+	Healthy	119.7	118.7	120.7
2017	Women	60+	Unaware	157.7	155.1	160.3
2017	Women	60+	Aware not treated	140.0	134.2	145.7
2017	Women	60+	Aware treated	145.1	142.1	148.0
2018	Men	<60	Healthy	120.3	120.0	120.7
2018	Men	<60	Unaware	147.1	145.9	148.2
2018	Men	<60	Aware not treated	134.7	129.2	140.3
2018	Men	<60	Aware treated	139.5	135.4	143.7
2018	Women	<60	Healthy	111.6	111.2	112.0
2018	Women	<60	Unaware	147.1	145.1	149.0
2018	Women	<60	Aware not treated	122.8	119.7	125.9
2018	Women	<60	Aware treated	131.3	128.5	134.2
2018	Men	60+	Healthy	121.9	121.0	122.9
2018	Men	60+	Unaware	156.5	153.6	159.4
2018	Men	60+	Aware not treated	140.9	134.1	147.7
2018	Men	60+	Aware treated	149.9	146.0	153.7
2018	Women	60+	Healthy	119.5	118.4	120.6
2018	Women	60+	Unaware	155.6	153.5	157.7
2018	Women	60+	Aware not treated	137.9	132.1	143.7
2018	Women	60+	Aware treated	144.3	141.5	147.1
2019	Men	<60	Healthy	120.5	120.1	120.9
2019	Men	<60	Unaware	146.9	145.6	148.3
2019	Men	<60	Aware not treated	132.7	128.8	136.6
2019	Men	<60	Aware treated	139.8	135.5	144.1
2019	Women	<60	Healthy	111.8	111.4	112.2
2019	Women	<60	Unaware	149.0	147.2	150.9
2019	Women	<60	Aware not treated	124.2	120.3	128.0
2019	Women	<60	Aware treated	132.9	129.6	136.2
2019	Men	60+	Healthy	121.6	120.6	122.6
2019	Men	60+	Unaware	153.9	152.0	155.8
2019	Men	60+	Aware not treated	143.7	137.7	149.7
2019	Men	60+	Aware treated	143.3	140.2	146.5
2019	Women	60+	Healthy	119.3	118.4	120.3
2019	Women	60+	Unaware	155.9	154.0	157.8
2019	Women	60+	Aware not treated	137.3	133.3	141.3
2019	Women	60+	Aware treated	144.3	141.0	147.6
2020	Men	<60	Healthy	121.0	120.6	121.5
2020	Men	<60	Unaware	148.7	146.8	150.7
2020	Men	<60	Aware not treated	133.3	129.5	137.2

2020	Men	<60	Aware treated	138.5	133.1	144.0
2020	Women	<60	Healthy	112.8	112.2	113.4
2020	Women	<60	Unaware	147.7	145.6	149.8
2020	Women	<60	Aware not treated	125.1	121.3	129.0
2020	Women	<60	Aware treated	129.8	126.3	133.4
2020	Men	60+	Healthy	122.9	121.8	124.0
2020	Men	60+	Unaware	156.4	153.6	159.1
2020	Men	60+	Aware not treated	141.1	134.5	147.7
2020	Men	60+	Aware treated	148.6	144.6	152.6
2020	Women	60+	Healthy	117.6	116.5	118.8
2020	Women	60+	Unaware	157.0	153.9	160.2
2020	Women	60+	Aware not treated	145.3	137.9	152.7
2020	Women	60+	Aware treated	138.7	135.4	142.0

Supplementary Table 3. At the sub-national level, mean systolic blood pressure (95% confidence intervals) by population group stratified by sex and study year

Year	Region	Sex	Age	Categories	Mean SBP	Lower limit	Upper limit
2015	Amazonas	Men	<60	Healthy	117.6	116.0	119.1
2015	Amazonas	Men	<60	Unaware	145.5	142.3	148.7
2015	Amazonas	Men	<60	Aware not treated	127.9	117.6	138.2
2015	Amazonas	Men	<60	Aware treated	158.0	128.9	187.0
2015	Amazonas	Men	60+	Healthy	121.8	118.3	125.2
2015	Amazonas	Men	60+	Unaware	158.9	147.5	170.4
2015	Amazonas	Men	60+	Aware not treated	127.5	113.0	142.0
2015	Amazonas	Men	60+	Aware treated	148.3	136.5	160.0
2016	Amazonas	Men	<60	Healthy	118.7	117.4	120.0
2016	Amazonas	Men	<60	Unaware	146.2	141.5	150.9
2016	Amazonas	Men	<60	Aware not treated	137.3	119.9	154.7
2016	Amazonas	Men	<60	Aware treated	158.8	145.9	171.7
2016	Amazonas	Men	60+	Healthy	117.4	112.9	121.9
2016	Amazonas	Men	60+	Unaware	161.7	147.8	175.5
2016	Amazonas	Men	60+	Aware not treated	145.8	112.1	179.6
2016	Amazonas	Men	60+	Aware treated	156.7	139.7	173.7
2017	Amazonas	Men	<60	Healthy	120.4	119.3	121.5
2017	Amazonas	Men	<60	Unaware	143.6	137.2	150.1
2017	Amazonas	Men	<60	Aware not treated	139.9	134.5	145.3
2017	Amazonas	Men	<60	Aware treated	134.4	126.2	142.6
2017	Amazonas	Men	60+	Healthy	119.9	116.5	123.3
2017	Amazonas	Men	60+	Unaware	157.2	149.2	165.2

2017	Amazonas	Men	60+	Aware not treated	180.6	155.3	205.9
2017	Amazonas	Men	60+	Aware treated	150.6	136.3	165.0
2018	Amazonas	Men	<60	Healthy	118.6	117.3	120.0
2018	Amazonas	Men	<60	Unaware	143.3	139.4	147.2
2018	Amazonas	Men	<60	Aware not treated	126.8	120.7	133.0
2018	Amazonas	Men	<60	Aware treated	138.0	123.9	152.2
2018	Amazonas	Men	60+	Healthy	118.3	115.1	121.5
2018	Amazonas	Men	60+	Unaware	160.5	154.6	166.5
2018	Amazonas	Men	60+	Aware not treated	148.0	125.8	170.1
2018	Amazonas	Men	60+	Aware treated	149.2	130.8	167.6
2019	Amazonas	Men	<60	Healthy	119.3	117.9	120.6
2019	Amazonas	Men	<60	Unaware	144.2	138.6	149.8
2019	Amazonas	Men	<60	Aware not treated	127.5	121.4	133.5
2019	Amazonas	Men	<60	Aware treated	133.8	123.0	144.5
2019	Amazonas	Men	60+	Healthy	117.7	113.6	121.7
2019	Amazonas	Men	60+	Unaware	159.2	151.4	166.9
2019	Amazonas	Men	60+	Aware not treated	138.8	129.3	148.4
2019	Amazonas	Men	60+	Aware treated	149.1	136.6	161.6
2020	Amazonas	Men	<60	Healthy	118.8	117.5	120.2
2020	Amazonas	Men	<60	Unaware	153.7	144.7	162.6
2020	Amazonas	Men	<60	Aware not treated	132.0	127.6	136.4
2020	Amazonas	Men	<60	Aware treated	136.0	118.2	153.8
2020	Amazonas	Men	60+	Healthy	116.4	110.3	122.6
2020	Amazonas	Men	60+	Unaware	152.1	146.5	157.7
2020	Amazonas	Men	60+	Aware not treated	126.0	126.0	126.0
2020	Amazonas	Men	60+	Aware treated	153.6	144.5	162.7
2015	Amazonas	Women	<60	Healthy	113.3	111.8	114.9
2015	Amazonas	Women	<60	Unaware	149.5	143.1	155.9
2015	Amazonas	Women	<60	Aware not treated	127.3	115.2	139.3

2015	Amazonas	Women	<60	Aware treated	142.0	119.9	164.1
2015	Amazonas	Women	60+	Healthy	121.5	117.1	125.9
2015	Amazonas	Women	60+	Unaware	160.9	148.2	173.5
2015	Amazonas	Women	60+	Aware not treated	134.3	108.3	160.2
2015	Amazonas	Women	60+	Aware treated	158.0	144.7	171.4
2016	Amazonas	Women	<60	Healthy	112.8	111.1	114.4
2016	Amazonas	Women	<60	Unaware	149.1	142.8	155.4
2016	Amazonas	Women	<60	Aware not treated	128.3	117.9	138.7
2016	Amazonas	Women	<60	Aware treated	151.0	129.6	172.4
2016	Amazonas	Women	60+	Healthy	120.0	116.0	124.0
2016	Amazonas	Women	60+	Unaware	154.3	146.2	162.4
2016	Amazonas	Women	60+	Aware not treated	160.0	138.9	181.1
2016	Amazonas	Women	60+	Aware treated	144.5	134.5	154.4
2017	Amazonas	Women	<60	Healthy	112.5	111.3	113.8
2017	Amazonas	Women	<60	Unaware	149.5	144.9	154.2
2017	Amazonas	Women	<60	Aware not treated	129.4	106.0	152.8
2017	Amazonas	Women	<60	Aware treated	132.7	121.9	143.6
2017	Amazonas	Women	60+	Healthy	116.3	111.8	120.9
2017	Amazonas	Women	60+	Unaware	159.4	153.0	165.9
2017	Amazonas	Women	60+	Aware not treated	131.2	120.2	142.2
2017	Amazonas	Women	60+	Aware treated	153.7	143.5	163.8
2018	Amazonas	Women	<60	Healthy	111.5	110.1	112.9
2018	Amazonas	Women	<60	Unaware	147.1	135.6	158.5
2018	Amazonas	Women	<60	Aware not treated	125.2	114.6	135.7
2018	Amazonas	Women	<60	Aware treated	129.5	121.0	137.9
2018	Amazonas	Women	60+	Healthy	119.3	115.5	123.0
2018	Amazonas	Women	60+	Unaware	165.2	150.5	179.9
2018	Amazonas	Women	60+	Aware not treated	134.8	114.7	155.0
2018	Amazonas	Women	60+	Aware treated	152.8	139.5	166.1

2019	Amazonas	Women	<60	Healthy	112.2	110.7	113.6
2019	Amazonas	Women	<60	Unaware	145.5	138.5	152.5
2019	Amazonas	Women	<60	Aware not treated	116.2	107.9	124.4
2019	Amazonas	Women	<60	Aware treated	141.6	129.7	153.5
2019	Amazonas	Women	60+	Healthy	120.1	116.8	123.3
2019	Amazonas	Women	60+	Unaware	156.5	148.8	164.1
2019	Amazonas	Women	60+	Aware not treated	120.5	108.0	133.0
2019	Amazonas	Women	60+	Aware treated	153.1	147.1	159.1
2020	Amazonas	Women	<60	Healthy	113.5	111.5	115.4
2020	Amazonas	Women	<60	Unaware	154.0	143.6	164.4
2020	Amazonas	Women	<60	Aware not treated	127.9	109.5	146.4
2020	Amazonas	Women	<60	Aware treated	135.1	120.3	149.9
2020	Amazonas	Women	60+	Healthy	119.1	112.3	125.9
2020	Amazonas	Women	60+	Unaware	160.2	150.1	170.3
2020	Amazonas	Women	60+	Aware not treated	143.1	119.3	166.9
2020	Amazonas	Women	60+	Aware treated	152.4	134.1	170.6
2015	Huanuco	Men	<60	Healthy	117.2	115.9	118.5
2015	Huanuco	Men	<60	Unaware	149.6	144.4	154.8
2015	Huanuco	Men	<60	Aware not treated	111.7	102.4	121.0
2015	Huanuco	Men	<60	Aware treated	142.0	121.0	163.1
2015	Huanuco	Men	60+	Healthy	116.6	112.8	120.5
2015	Huanuco	Men	60+	Unaware	156.8	146.2	167.5
2015	Huanuco	Men	60+	Aware not treated	128.8	117.5	140.0
2015	Huanuco	Men	60+	Aware treated	168.0	137.8	198.1
2016	Huanuco	Men	<60	Healthy	117.6	116.3	118.9
2016	Huanuco	Men	<60	Unaware	147.8	144.8	150.9
2016	Huanuco	Men	<60	Aware not treated	121.9	114.2	129.6
2016	Huanuco	Men	<60	Aware treated	126.5	117.6	135.3
2016	Huanuco	Men	60+	Healthy	118.3	115.0	121.6

2016	Huanuco	Men	60+	Unaware	155.1	146.9	163.4
2016	Huanuco	Men	60+	Aware treated	150.3	138.9	161.6
2017	Huanuco	Men	<60	Healthy	117.2	115.8	118.7
2017	Huanuco	Men	<60	Unaware	146.0	141.2	150.8
2017	Huanuco	Men	<60	Aware not treated	118.0	112.0	124.0
2017	Huanuco	Men	<60	Aware treated	127.7	119.4	136.0
2017	Huanuco	Men	60+	Healthy	118.2	115.1	121.2
2017	Huanuco	Men	60+	Unaware	156.0	148.5	163.5
2017	Huanuco	Men	60+	Aware not treated	124.4	117.2	131.7
2017	Huanuco	Men	60+	Aware treated	147.1	135.4	158.8
2018	Huanuco	Men	<60	Healthy	117.4	116.2	118.7
2018	Huanuco	Men	<60	Unaware	140.6	137.9	143.4
2018	Huanuco	Men	<60	Aware not treated	124.9	114.8	135.0
2018	Huanuco	Men	<60	Aware treated	134.4	123.6	145.2
2018	Huanuco	Men	60+	Healthy	119.4	116.4	122.3
2018	Huanuco	Men	60+	Unaware	157.8	150.7	164.8
2018	Huanuco	Men	60+	Aware not treated	139.0	101.9	176.2
2018	Huanuco	Men	60+	Aware treated	141.9	122.9	160.9
2019	Huanuco	Men	<60	Healthy	118.3	117.0	119.5
2019	Huanuco	Men	<60	Unaware	146.1	142.0	150.3
2019	Huanuco	Men	<60	Aware not treated	124.8	115.3	134.3
2019	Huanuco	Men	<60	Aware treated	119.4	112.6	126.3
2019	Huanuco	Men	60+	Healthy	121.4	118.0	124.9
2019	Huanuco	Men	60+	Unaware	159.2	152.7	165.6
2019	Huanuco	Men	60+	Aware not treated	130.9	97.1	164.8
2019	Huanuco	Men	60+	Aware treated	148.4	139.3	157.5
2020	Huanuco	Men	<60	Healthy	118.6	116.8	120.4
2020	Huanuco	Men	<60	Unaware	149.0	145.5	152.5
2020	Huanuco	Men	<60	Aware not treated	124.8	111.4	138.3

2020	Huanuco	Men	60+	Healthy	121.4	116.4	126.5
2020	Huanuco	Men	60+	Unaware	153.3	147.3	159.3
2020	Huanuco	Men	60+	Aware not treated	137.8	124.1	151.5
2020	Huanuco	Men	60+	Aware treated	130.0	118.0	142.0
2015	Huanuco	Women	<60	Healthy	110.0	108.8	111.1
2015	Huanuco	Women	<60	Unaware	147.0	137.1	157.0
2015	Huanuco	Women	<60	Aware not treated	126.4	118.3	134.5
2015	Huanuco	Women	<60	Aware treated	126.7	116.0	137.3
2015	Huanuco	Women	60+	Healthy	120.5	117.0	123.9
2015	Huanuco	Women	60+	Unaware	155.5	148.3	162.7
2015	Huanuco	Women	60+	Aware not treated	141.9	126.0	157.8
2015	Huanuco	Women	60+	Aware treated	153.0	130.8	175.2
2016	Huanuco	Women	<60	Healthy	110.8	109.3	112.2
2016	Huanuco	Women	<60	Unaware	148.3	144.8	151.7
2016	Huanuco	Women	<60	Aware not treated	124.4	109.9	138.9
2016	Huanuco	Women	<60	Aware treated	125.1	114.5	135.6
2016	Huanuco	Women	60+	Healthy	116.0	112.9	119.2
2016	Huanuco	Women	60+	Unaware	153.4	147.5	159.4
2016	Huanuco	Women	60+	Aware not treated	135.3	126.0	144.6
2016	Huanuco	Women	60+	Aware treated	139.0	115.0	163.0
2017	Huanuco	Women	<60	Healthy	109.2	107.8	110.5
2017	Huanuco	Women	<60	Unaware	153.5	144.7	162.3
2017	Huanuco	Women	<60	Aware not treated	109.7	106.4	112.9
2017	Huanuco	Women	<60	Aware treated	124.8	115.4	134.3
2017	Huanuco	Women	60+	Healthy	112.3	109.3	115.3
2017	Huanuco	Women	60+	Unaware	149.7	145.2	154.2
2017	Huanuco	Women	60+	Aware not treated	133.6	105.3	161.8
2017	Huanuco	Women	60+	Aware treated	127.5	116.6	138.5
2018	Huanuco	Women	<60	Healthy	107.3	106.1	108.5

2018	Huanuco	Women	<60	Unaware	140.0	134.2	145.8
2018	Huanuco	Women	<60	Aware not treated	117.2	108.4	126.1
2018	Huanuco	Women	<60	Aware treated	124.4	116.5	132.4
2018	Huanuco	Women	60+	Healthy	116.7	113.0	120.4
2018	Huanuco	Women	60+	Unaware	153.2	147.0	159.3
2018	Huanuco	Women	60+	Aware not treated	128.5	124.8	132.2
2018	Huanuco	Women	60+	Aware treated	136.9	125.4	148.4
2019	Huanuco	Women	<60	Healthy	108.3	107.0	109.7
2019	Huanuco	Women	<60	Unaware	141.1	135.5	146.6
2019	Huanuco	Women	<60	Aware not treated	126.9	114.9	139.0
2019	Huanuco	Women	<60	Aware treated	122.1	114.5	129.7
2019	Huanuco	Women	60+	Healthy	115.6	111.7	119.5
2019	Huanuco	Women	60+	Unaware	165.3	146.5	184.1
2019	Huanuco	Women	60+	Aware not treated	116.2	107.0	125.3
2019	Huanuco	Women	60+	Aware treated	140.1	132.2	148.0
2020	Huanuco	Women	<60	Healthy	112.3	110.1	114.4
2020	Huanuco	Women	<60	Unaware	148.3	141.5	155.2
2020	Huanuco	Women	<60	Aware not treated	115.2	109.9	120.5
2020	Huanuco	Women	<60	Aware treated	118.5	103.2	133.9
2020	Huanuco	Women	60+	Healthy	115.6	111.3	119.8
2020	Huanuco	Women	60+	Unaware	152.8	146.9	158.7
2020	Huanuco	Women	60+	Aware not treated	125.2	112.5	137.8
2020	Huanuco	Women	60+	Aware treated	141.0	130.5	151.5
2015	Ica	Men	<60	Healthy	121.2	119.8	122.6
2015	Ica	Men	<60	Unaware	145.5	142.9	148.2
2015	Ica	Men	<60	Aware not treated	156.5	139.9	173.2
2015	Ica	Men	<60	Aware treated	147.9	135.5	160.2
2015	Ica	Men	60+	Healthy	121.6	118.6	124.7
2015	Ica	Men	60+	Unaware	158.6	151.6	165.6

2015	Ica	Men	60+	Aware not treated	147.8	122.7	172.9
2015	Ica	Men	60+	Aware treated	145.5	130.6	160.3
2016	Ica	Men	<60	Healthy	120.8	119.4	122.2
2016	Ica	Men	<60	Unaware	148.5	143.8	153.1
2016	Ica	Men	<60	Aware not treated	138.8	127.2	150.4
2016	Ica	Men	<60	Aware treated	166.1	153.5	178.8
2016	Ica	Men	60+	Healthy	120.5	117.4	123.6
2016	Ica	Men	60+	Unaware	149.8	146.2	153.3
2016	Ica	Men	60+	Aware not treated	145.9	118.6	173.2
2016	Ica	Men	60+	Aware treated	141.3	129.8	152.9
2017	Ica	Men	<60	Healthy	121.9	120.5	123.2
2017	Ica	Men	<60	Unaware	144.8	142.4	147.2
2017	Ica	Men	<60	Aware not treated	135.7	129.7	141.6
2017	Ica	Men	<60	Aware treated	134.2	121.2	147.2
2017	Ica	Men	60+	Healthy	124.3	120.6	128.1
2017	Ica	Men	60+	Unaware	160.2	154.5	165.9
2017	Ica	Men	60+	Aware not treated	152.5	142.5	162.4
2017	Ica	Men	60+	Aware treated	149.8	140.2	159.5
2018	Ica	Men	<60	Healthy	122.4	121.0	123.7
2018	Ica	Men	<60	Unaware	145.2	140.8	149.6
2018	Ica	Men	<60	Aware not treated	133.9	113.9	154.0
2018	Ica	Men	<60	Aware treated	141.5	130.2	152.7
2018	Ica	Men	60+	Healthy	121.1	117.6	124.7
2018	Ica	Men	60+	Unaware	161.2	154.4	168.0
2018	Ica	Men	60+	Aware not treated	128.4	116.8	140.0
2018	Ica	Men	60+	Aware treated	139.8	131.2	148.3
2019	Ica	Men	<60	Healthy	120.2	118.7	121.7
2019	Ica	Men	<60	Unaware	143.2	139.6	146.8
2019	Ica	Men	<60	Aware not treated	136.4	127.7	145.2

2019	Ica	Men	<60	Aware treated	132.4	121.1	143.6
2019	Ica	Men	60+	Healthy	124.5	121.7	127.3
2019	Ica	Men	60+	Unaware	155.1	150.0	160.2
2019	Ica	Men	60+	Aware not treated	177.1	110.4	243.9
2019	Ica	Men	60+	Aware treated	149.5	140.7	158.4
2020	Ica	Men	<60	Healthy	120.8	119.0	122.7
2020	Ica	Men	<60	Unaware	148.9	141.3	156.5
2020	Ica	Men	<60	Aware not treated	132.5	118.7	146.3
2020	Ica	Men	<60	Aware treated	145.8	121.6	169.9
2020	Ica	Men	60+	Healthy	122.1	118.4	125.8
2020	Ica	Men	60+	Unaware	151.6	145.2	158.0
2020	Ica	Men	60+	Aware not treated	147.4	121.4	173.4
2020	Ica	Men	60+	Aware treated	145.0	133.8	156.1
2015	Ica	Women	<60	Healthy	112.1	110.7	113.4
2015	Ica	Women	<60	Unaware	144.3	140.4	148.2
2015	Ica	Women	<60	Aware not treated	129.9	118.9	141.0
2015	Ica	Women	<60	Aware treated	129.4	121.2	137.5
2015	Ica	Women	60+	Healthy	117.3	112.7	121.9
2015	Ica	Women	60+	Unaware	157.2	144.8	169.7
2015	Ica	Women	60+	Aware not treated	151.8	142.8	160.8
2015	Ica	Women	60+	Aware treated	148.0	135.0	161.0
2016	Ica	Women	<60	Healthy	113.4	111.9	115.0
2016	Ica	Women	<60	Unaware	151.8	141.7	161.9
2016	Ica	Women	<60	Aware not treated	133.1	115.9	150.3
2016	Ica	Women	<60	Aware treated	122.3	112.4	132.2
2016	Ica	Women	60+	Healthy	120.6	116.3	124.9
2016	Ica	Women	60+	Unaware	153.5	147.6	159.5
2016	Ica	Women	60+	Aware not treated	122.5	110.1	134.9
2016	Ica	Women	60+	Aware treated	145.9	133.1	158.7

2017	Ica	Women	<60	Healthy	113.4	112.1	114.7
2017	Ica	Women	<60	Unaware	148.2	143.3	153.1
2017	Ica	Women	<60	Aware not treated	132.5	111.2	153.8
2017	Ica	Women	<60	Aware treated	129.9	117.5	142.3
2017	Ica	Women	60+	Healthy	117.6	113.0	122.2
2017	Ica	Women	60+	Unaware	163.1	150.8	175.5
2017	Ica	Women	60+	Aware not treated	141.7	123.7	159.7
2017	Ica	Women	60+	Aware treated	151.8	142.3	161.2
2018	Ica	Women	<60	Healthy	111.7	110.1	113.2
2018	Ica	Women	<60	Unaware	145.1	140.1	150.1
2018	Ica	Women	<60	Aware not treated	119.2	114.3	124.1
2018	Ica	Women	<60	Aware treated	121.1	113.9	128.4
2018	Ica	Women	60+	Healthy	120.9	117.4	124.3
2018	Ica	Women	60+	Unaware	150.8	143.3	158.4
2018	Ica	Women	60+	Aware not treated	138.7	129.8	147.6
2018	Ica	Women	60+	Aware treated	138.2	131.3	145.0
2019	Ica	Women	<60	Healthy	111.8	110.5	113.2
2019	Ica	Women	<60	Unaware	142.2	134.3	150.1
2019	Ica	Women	<60	Aware not treated	119.2	104.2	134.2
2019	Ica	Women	<60	Aware treated	137.5	125.0	149.9
2019	Ica	Women	60+	Healthy	119.9	116.5	123.2
2019	Ica	Women	60+	Unaware	155.5	147.9	163.1
2019	Ica	Women	60+	Aware not treated	121.4	106.9	135.9
2019	Ica	Women	60+	Aware treated	139.4	128.0	150.7
2020	Ica	Women	<60	Healthy	110.8	109.1	112.5
2020	Ica	Women	<60	Unaware	148.8	144.9	152.7
2020	Ica	Women	<60	Aware not treated	139.8	124.0	155.7
2020	Ica	Women	<60	Aware treated	131.1	113.1	149.2
2020	Ica	Women	60+	Healthy	119.3	113.9	124.7

2020	Ica	Women	60+	Unaware	159.8	149.8	169.8
2020	Ica	Women	60+	Aware not treated	166.1	164.1	168.1
2020	Ica	Women	60+	Aware treated	140.8	125.3	156.3
2015	Junin	Men	<60	Healthy	118.0	116.1	119.8
2015	Junin	Men	<60	Unaware	141.8	137.3	146.2
2015	Junin	Men	<60	Aware not treated	126.6	119.2	133.9
2015	Junin	Men	<60	Aware treated	120.9	104.7	137.1
2015	Junin	Men	60+	Healthy	121.0	117.6	124.5
2015	Junin	Men	60+	Unaware	159.6	152.9	166.3
2015	Junin	Men	60+	Aware not treated	139.2	125.4	153.0
2015	Junin	Men	60+	Aware treated	137.0	117.2	156.8
2016	Junin	Men	<60	Healthy	118.1	116.4	119.8
2016	Junin	Men	<60	Unaware	150.5	146.9	154.2
2016	Junin	Men	<60	Aware not treated	130.7	119.7	141.8
2016	Junin	Men	<60	Aware treated	133.2	117.3	149.0
2016	Junin	Men	60+	Healthy	121.0	117.6	124.5
2016	Junin	Men	60+	Unaware	152.7	146.8	158.6
2016	Junin	Men	60+	Aware not treated	130.0	130.0	130.0
2016	Junin	Men	60+	Aware treated	153.0	126.6	179.5
2017	Junin	Men	<60	Healthy	119.0	117.2	120.9
2017	Junin	Men	<60	Unaware	146.6	140.6	152.6
2017	Junin	Men	<60	Aware not treated	133.6	124.5	142.6
2017	Junin	Men	<60	Aware treated	141.4	111.9	170.8
2017	Junin	Men	60+	Healthy	117.1	112.8	121.3
2017	Junin	Men	60+	Unaware	155.0	148.0	162.0
2017	Junin	Men	60+	Aware not treated	159.8	134.2	185.3
2017	Junin	Men	60+	Aware treated	146.1	121.0	171.3
2018	Junin	Men	<60	Healthy	117.1	115.6	118.7
2018	Junin	Men	<60	Unaware	142.4	137.9	146.9

2018	Junin	Men	<60	Aware not treated	121.6	116.7	126.6
2018	Junin	Men	<60	Aware treated	132.7	127.7	137.7
2018	Junin	Men	60+	Healthy	120.8	117.1	124.5
2018	Junin	Men	60+	Unaware	155.1	146.9	163.4
2018	Junin	Men	60+	Aware not treated	176.0	132.7	219.2
2018	Junin	Men	60+	Aware treated	146.9	136.5	157.4
2019	Junin	Men	<60	Healthy	118.5	116.6	120.4
2019	Junin	Men	<60	Unaware	140.9	133.3	148.4
2019	Junin	Men	<60	Aware not treated	126.2	116.5	136.0
2019	Junin	Men	<60	Aware treated	132.7	123.8	141.5
2019	Junin	Men	60+	Healthy	120.9	117.8	124.1
2019	Junin	Men	60+	Unaware	151.9	144.6	159.2
2019	Junin	Men	60+	Aware not treated	113.7	80.6	146.7
2019	Junin	Men	60+	Aware treated	142.8	123.5	162.1
2020	Junin	Men	<60	Healthy	117.4	115.7	119.2
2020	Junin	Men	<60	Unaware	144.0	136.6	151.4
2020	Junin	Men	<60	Aware not treated	124.7	124.0	125.3
2020	Junin	Men	<60	Aware treated	124.8	121.9	127.7
2020	Junin	Men	60+	Healthy	120.9	117.0	124.9
2020	Junin	Men	60+	Unaware	155.1	144.2	165.9
2020	Junin	Men	60+	Aware not treated	140.0	140.0	140.0
2020	Junin	Men	60+	Aware treated	161.5	139.7	183.3
2015	Junin	Women	<60	Healthy	110.5	108.9	112.2
2015	Junin	Women	<60	Unaware	145.9	143.7	148.2
2015	Junin	Women	<60	Aware not treated	113.6	108.7	118.6
2015	Junin	Women	<60	Aware treated	115.8	106.6	125.0
2015	Junin	Women	60+	Healthy	118.7	114.2	123.2
2015	Junin	Women	60+	Unaware	150.2	144.3	156.2
2015	Junin	Women	60+	Aware not treated	132.1	119.6	144.6

2015	Junin	Women	60+	Aware treated	136.8	124.6	148.9
2016	Junin	Women	<60	Healthy	110.2	108.8	111.6
2016	Junin	Women	<60	Unaware	147.5	141.7	153.2
2016	Junin	Women	<60	Aware not treated	113.8	105.9	121.7
2016	Junin	Women	<60	Aware treated	114.4	95.0	133.8
2016	Junin	Women	60+	Healthy	116.2	112.6	119.7
2016	Junin	Women	60+	Unaware	154.3	148.6	159.9
2016	Junin	Women	60+	Aware not treated	123.4	109.6	137.1
2016	Junin	Women	60+	Aware treated	136.8	129.2	144.4
2017	Junin	Women	<60	Healthy	110.8	109.2	112.3
2017	Junin	Women	<60	Unaware	144.5	139.7	149.4
2017	Junin	Women	<60	Aware not treated	116.4	108.9	123.8
2017	Junin	Women	<60	Aware treated	120.6	109.3	132.0
2017	Junin	Women	60+	Healthy	119.0	116.0	122.0
2017	Junin	Women	60+	Unaware	165.2	154.9	175.6
2017	Junin	Women	60+	Aware not treated	143.4	129.6	157.3
2017	Junin	Women	60+	Aware treated	141.1	131.2	151.0
2018	Junin	Women	<60	Healthy	109.9	108.6	111.1
2018	Junin	Women	<60	Unaware	134.8	128.1	141.6
2018	Junin	Women	<60	Aware not treated	115.8	106.7	124.9
2018	Junin	Women	<60	Aware treated	128.0	114.7	141.3
2018	Junin	Women	60+	Healthy	118.7	115.1	122.3
2018	Junin	Women	60+	Unaware	154.2	149.3	159.0
2018	Junin	Women	60+	Aware not treated	116.5	105.3	127.6
2018	Junin	Women	60+	Aware treated	128.8	119.5	138.1
2019	Junin	Women	<60	Healthy	109.2	107.9	110.6
2019	Junin	Women	<60	Unaware	144.3	135.7	152.9
2019	Junin	Women	<60	Aware not treated	113.3	108.3	118.3
2019	Junin	Women	<60	Aware treated	125.8	113.0	138.7

2019	Junin	Women	60+	Healthy	118.6	114.2	123.0
2019	Junin	Women	60+	Unaware	154.9	148.9	161.0
2019	Junin	Women	60+	Aware not treated	139.5	110.7	168.2
2019	Junin	Women	60+	Aware treated	144.9	135.1	154.7
2020	Junin	Women	<60	Healthy	109.8	108.2	111.4
2020	Junin	Women	<60	Unaware	130.8	124.0	137.5
2020	Junin	Women	<60	Aware not treated	115.5	110.3	120.7
2020	Junin	Women	<60	Aware treated	129.6	115.7	143.5
2020	Junin	Women	60+	Healthy	116.7	113.2	120.2
2020	Junin	Women	60+	Unaware	152.9	143.3	162.5
2020	Junin	Women	60+	Aware not treated	124.5	114.1	135.0
2020	Junin	Women	60+	Aware treated	131.4	115.4	147.5
2015	La Libertad	Men	<60	Healthy	118.1	116.2	120.1
2015	La Libertad	Men	<60	Unaware	143.9	140.9	146.9
2015	La Libertad	Men	<60	Aware not treated	129.1	119.2	139.0
2015	La Libertad	Men	<60	Aware treated	136.2	134.6	137.8
2015	La Libertad	Men	60+	Healthy	119.3	115.6	123.0
2015	La Libertad	Men	60+	Unaware	153.4	143.5	163.4
2015	La Libertad	Men	60+	Aware not treated	159.8	150.9	168.7
2015	La Libertad	Men	60+	Aware treated	150.7	138.4	163.1
2016	La Libertad	Men	<60	Healthy	118.6	117.2	120.1
2016	La Libertad	Men	<60	Unaware	144.0	141.0	146.9
2016	La Libertad	Men	<60	Aware not treated	147.9	128.8	166.9
2016	La Libertad	Men	<60	Aware treated	115.0	115.0	115.0
2016	La Libertad	Men	60+	Healthy	123.3	119.1	127.6
2016	La Libertad	Men	60+	Unaware	151.8	145.4	158.1
2016	La Libertad	Men	60+	Aware not treated	155.4	136.6	174.2
2016	La Libertad	Men	60+	Aware treated	146.8	131.3	162.3
2017	La Libertad	Men	<60	Healthy	119.1	117.7	120.5

2017	La Libertad	Men	<60	Unaware	150.1	143.4	156.7
2017	La Libertad	Men	<60	Aware not treated	145.4	128.6	162.3
2017	La Libertad	Men	<60	Aware treated	119.9	117.7	122.1
2017	La Libertad	Men	60+	Healthy	123.7	120.5	127.0
2017	La Libertad	Men	60+	Unaware	155.4	143.7	167.1
2017	La Libertad	Men	60+	Aware not treated	170.8	120.7	221.0
2017	La Libertad	Men	60+	Aware treated	146.8	134.4	159.1
2018	La Libertad	Men	<60	Healthy	117.6	116.0	119.3
2018	La Libertad	Men	<60	Unaware	144.0	139.4	148.6
2018	La Libertad	Men	<60	Aware not treated	140.0	119.7	160.3
2018	La Libertad	Men	<60	Aware treated	124.0	105.8	142.3
2018	La Libertad	Men	60+	Healthy	121.2	117.3	125.1
2018	La Libertad	Men	60+	Unaware	156.5	149.6	163.3
2018	La Libertad	Men	60+	Aware not treated	118.1	110.9	125.3
2018	La Libertad	Men	60+	Aware treated	158.5	146.0	171.0
2019	La Libertad	Men	<60	Healthy	118.8	117.6	120.0
2019	La Libertad	Men	<60	Unaware	147.6	143.4	151.7
2019	La Libertad	Men	<60	Aware not treated	126.9	107.4	146.4
2019	La Libertad	Men	<60	Aware treated	138.3	125.4	151.2
2019	La Libertad	Men	60+	Healthy	121.9	117.3	126.4
2019	La Libertad	Men	60+	Unaware	156.0	148.6	163.5
2019	La Libertad	Men	60+	Aware not treated	134.7	120.4	149.1
2019	La Libertad	Men	60+	Aware treated	134.8	123.7	145.8
2020	La Libertad	Men	<60	Healthy	119.2	117.4	121.1
2020	La Libertad	Men	<60	Unaware	143.7	140.3	147.0
2020	La Libertad	Men	<60	Aware not treated	140.2	118.6	161.8
2020	La Libertad	Men	<60	Aware treated	143.0	143.0	143.0
2020	La Libertad	Men	60+	Healthy	120.6	114.6	126.6
2020	La Libertad	Men	60+	Unaware	146.1	140.4	151.7

2020	La Libertad	Men	60+	Aware not treated	142.9	97.7	188.0
2020	La Libertad	Men	60+	Aware treated	156.8	137.2	176.4
2015	La Libertad	Women	<60	Healthy	111.1	109.2	112.9
2015	La Libertad	Women	<60	Unaware	154.8	146.7	162.8
2015	La Libertad	Women	<60	Aware not treated	123.7	117.4	130.0
2015	La Libertad	Women	<60	Aware treated	127.3	118.3	136.3
2015	La Libertad	Women	60+	Healthy	120.6	115.8	125.3
2015	La Libertad	Women	60+	Unaware	153.0	148.3	157.7
2015	La Libertad	Women	60+	Aware not treated	144.7	125.7	163.8
2015	La Libertad	Women	60+	Aware treated	148.3	141.0	155.6
2016	La Libertad	Women	<60	Healthy	111.5	109.9	113.1
2016	La Libertad	Women	<60	Unaware	142.3	134.9	149.6
2016	La Libertad	Women	<60	Aware not treated	121.4	108.5	134.3
2016	La Libertad	Women	<60	Aware treated	127.5	117.7	137.2
2016	La Libertad	Women	60+	Healthy	119.6	114.6	124.5
2016	La Libertad	Women	60+	Unaware	153.7	148.0	159.4
2016	La Libertad	Women	60+	Aware not treated	123.1	112.0	134.2
2016	La Libertad	Women	60+	Aware treated	143.4	132.7	154.1
2017	La Libertad	Women	<60	Healthy	111.3	109.6	113.0
2017	La Libertad	Women	<60	Unaware	151.6	146.4	156.7
2017	La Libertad	Women	<60	Aware not treated	111.4	106.1	116.7
2017	La Libertad	Women	<60	Aware treated	126.6	113.3	139.8
2017	La Libertad	Women	60+	Healthy	121.7	118.2	125.3
2017	La Libertad	Women	60+	Unaware	155.8	151.5	160.1
2017	La Libertad	Women	60+	Aware not treated	128.6	113.3	143.9
2017	La Libertad	Women	60+	Aware treated	148.5	136.6	160.5
2018	La Libertad	Women	<60	Healthy	110.3	109.1	111.6
2018	La Libertad	Women	<60	Unaware	144.4	138.4	150.5
2018	La Libertad	Women	<60	Aware not treated	118.9	111.7	126.1

2018	La Libertad	Women	<60	Aware treated	139.4	127.7	151.0
2018	La Libertad	Women	60+	Healthy	119.8	115.5	124.2
2018	La Libertad	Women	60+	Unaware	161.8	152.7	171.0
2018	La Libertad	Women	60+	Aware not treated	137.0	123.3	150.7
2018	La Libertad	Women	60+	Aware treated	145.6	134.0	157.2
2019	La Libertad	Women	<60	Healthy	111.8	110.3	113.3
2019	La Libertad	Women	<60	Unaware	144.5	136.1	153.0
2019	La Libertad	Women	<60	Aware not treated	119.8	107.1	132.6
2019	La Libertad	Women	<60	Aware treated	136.1	126.0	146.3
2019	La Libertad	Women	60+	Healthy	119.2	115.7	122.7
2019	La Libertad	Women	60+	Unaware	148.8	144.9	152.7
2019	La Libertad	Women	60+	Aware not treated	137.4	131.1	143.7
2019	La Libertad	Women	60+	Aware treated	150.0	138.4	161.6
2020	La Libertad	Women	<60	Healthy	110.3	108.8	111.9
2020	La Libertad	Women	<60	Unaware	144.5	138.1	151.0
2020	La Libertad	Women	<60	Aware not treated	117.7	108.6	126.9
2020	La Libertad	Women	<60	Aware treated	140.0	126.2	153.7
2020	La Libertad	Women	60+	Healthy	115.6	110.1	121.2
2020	La Libertad	Women	60+	Unaware	154.1	147.6	160.7
2020	La Libertad	Women	60+	Aware not treated	146.4	136.9	155.9
2020	La Libertad	Women	60+	Aware treated	131.0	122.6	139.4
2015	Lambayeque	Men	<60	Healthy	121.3	119.9	122.7
2015	Lambayeque	Men	<60	Unaware	148.5	145.7	151.3
2015	Lambayeque	Men	<60	Aware not treated	126.3	124.2	128.3
2015	Lambayeque	Men	<60	Aware treated	142.4	120.7	164.2
2015	Lambayeque	Men	60+	Healthy	121.0	118.3	123.8
2015	Lambayeque	Men	60+	Unaware	167.3	150.8	183.8
2015	Lambayeque	Men	60+	Aware not treated	163.8	159.1	168.4
2015	Lambayeque	Men	60+	Aware treated	141.3	133.9	148.8

2016	Lambayeque	Men	<60	Healthy	121.8	120.6	123.0
2016	Lambayeque	Men	<60	Unaware	149.7	144.9	154.6
2016	Lambayeque	Men	<60	Aware not treated	132.9	106.6	159.2
2016	Lambayeque	Men	<60	Aware treated	132.4	111.4	153.4
2016	Lambayeque	Men	60+	Healthy	122.7	119.8	125.6
2016	Lambayeque	Men	60+	Unaware	155.0	147.4	162.6
2016	Lambayeque	Men	60+	Aware not treated	146.0	134.7	157.3
2016	Lambayeque	Men	60+	Aware treated	146.4	135.7	157.1
2017	Lambayeque	Men	<60	Healthy	120.0	118.5	121.4
2017	Lambayeque	Men	<60	Unaware	146.6	142.3	150.8
2017	Lambayeque	Men	<60	Aware not treated	113.8	113.8	113.8
2017	Lambayeque	Men	<60	Aware treated	143.6	136.7	150.5
2017	Lambayeque	Men	60+	Healthy	123.0	120.4	125.7
2017	Lambayeque	Men	60+	Unaware	149.7	145.4	153.9
2017	Lambayeque	Men	60+	Aware not treated	128.0	128.0	128.0
2017	Lambayeque	Men	60+	Aware treated	145.2	135.6	154.9
2018	Lambayeque	Men	<60	Healthy	121.8	120.4	123.2
2018	Lambayeque	Men	<60	Unaware	145.6	141.2	149.9
2018	Lambayeque	Men	<60	Aware not treated	125.7	108.4	143.0
2018	Lambayeque	Men	<60	Aware treated	140.6	131.5	149.7
2018	Lambayeque	Men	60+	Healthy	124.1	121.8	126.4
2018	Lambayeque	Men	60+	Unaware	152.5	146.6	158.4
2018	Lambayeque	Men	60+	Aware not treated	136.8	125.0	148.6
2018	Lambayeque	Men	60+	Aware treated	153.7	146.0	161.4
2019	Lambayeque	Men	<60	Healthy	120.4	119.1	121.7
2019	Lambayeque	Men	<60	Unaware	141.9	136.5	147.2
2019	Lambayeque	Men	<60	Aware not treated	157.9	146.1	169.8
2019	Lambayeque	Men	<60	Aware treated	142.2	134.5	149.8
2019	Lambayeque	Men	60+	Healthy	122.9	120.3	125.5

2019	Lambayeque	Men	60+	Unaware	160.7	148.9	172.6
2019	Lambayeque	Men	60+	Aware not treated	154.2	140.4	167.9
2019	Lambayeque	Men	60+	Aware treated	136.1	126.7	145.5
2020	Lambayeque	Men	<60	Healthy	121.8	120.4	123.1
2020	Lambayeque	Men	<60	Unaware	147.1	140.5	153.7
2020	Lambayeque	Men	<60	Aware not treated	134.9	123.6	146.2
2020	Lambayeque	Men	<60	Aware treated	136.9	122.6	151.2
2020	Lambayeque	Men	60+	Healthy	127.7	123.4	132.0
2020	Lambayeque	Men	60+	Unaware	152.2	140.5	163.9
2020	Lambayeque	Men	60+	Aware not treated	162.0	162.0	162.0
2020	Lambayeque	Men	60+	Aware treated	149.3	144.2	154.4
2015	Lambayeque	Women	<60	Healthy	112.7	111.1	114.3
2015	Lambayeque	Women	<60	Unaware	142.0	129.0	155.1
2015	Lambayeque	Women	<60	Aware not treated	134.4	121.5	147.3
2015	Lambayeque	Women	<60	Aware treated	135.9	129.1	142.8
2015	Lambayeque	Women	60+	Healthy	121.5	118.6	124.5
2015	Lambayeque	Women	60+	Unaware	158.0	153.0	163.0
2015	Lambayeque	Women	60+	Aware not treated	153.5	120.2	186.9
2015	Lambayeque	Women	60+	Aware treated	142.6	134.9	150.2
2016	Lambayeque	Women	<60	Healthy	113.1	111.6	114.5
2016	Lambayeque	Women	<60	Unaware	153.5	147.7	159.4
2016	Lambayeque	Women	<60	Aware not treated	125.1	122.2	128.0
2016	Lambayeque	Women	<60	Aware treated	133.5	126.2	140.8
2016	Lambayeque	Women	60+	Healthy	118.5	114.9	122.2
2016	Lambayeque	Women	60+	Unaware	157.1	149.3	164.9
2016	Lambayeque	Women	60+	Aware not treated	165.0	165.0	165.0
2016	Lambayeque	Women	60+	Aware treated	151.8	141.4	162.2
2017	Lambayeque	Women	<60	Healthy	112.8	111.5	114.2
2017	Lambayeque	Women	<60	Unaware	147.7	137.2	158.3

2017	Lambayeque	Women	<60	Aware not treated	125.5	116.0	135.1
2017	Lambayeque	Women	<60	Aware treated	131.7	124.1	139.3
2017	Lambayeque	Women	60+	Healthy	122.1	118.7	125.5
2017	Lambayeque	Women	60+	Unaware	151.3	146.6	156.1
2017	Lambayeque	Women	60+	Aware not treated	191.0	191.0	191.0
2017	Lambayeque	Women	60+	Aware treated	142.7	134.9	150.5
2018	Lambayeque	Women	<60	Healthy	111.9	110.7	113.1
2018	Lambayeque	Women	<60	Unaware	142.6	137.2	148.0
2018	Lambayeque	Women	<60	Aware not treated	116.2	109.3	123.1
2018	Lambayeque	Women	<60	Aware treated	132.0	123.1	140.9
2018	Lambayeque	Women	60+	Healthy	114.8	111.7	117.9
2018	Lambayeque	Women	60+	Unaware	156.2	151.0	161.3
2018	Lambayeque	Women	60+	Aware not treated	125.0	125.0	125.0
2018	Lambayeque	Women	60+	Aware treated	142.2	135.7	148.7
2019	Lambayeque	Women	<60	Healthy	112.3	111.2	113.4
2019	Lambayeque	Women	<60	Unaware	141.7	122.5	160.9
2019	Lambayeque	Women	<60	Aware not treated	106.3	100.5	112.0
2019	Lambayeque	Women	<60	Aware treated	123.4	114.7	132.1
2019	Lambayeque	Women	60+	Healthy	121.3	118.1	124.6
2019	Lambayeque	Women	60+	Unaware	153.2	144.9	161.5
2019	Lambayeque	Women	60+	Aware not treated	139.3	126.5	152.1
2019	Lambayeque	Women	60+	Aware treated	150.3	141.4	159.1
2020	Lambayeque	Women	<60	Healthy	112.6	110.8	114.5
2020	Lambayeque	Women	<60	Unaware	141.4	132.4	150.4
2020	Lambayeque	Women	<60	Aware not treated	154.5	117.4	191.7
2020	Lambayeque	Women	<60	Aware treated	126.4	119.3	133.5
2020	Lambayeque	Women	60+	Healthy	120.6	116.2	125.0
2020	Lambayeque	Women	60+	Unaware	160.7	149.5	171.9
2020	Lambayeque	Women	60+	Aware not treated	111.0	111.0	111.0

2020	Lambayeque	Women	60+	Aware treated	127.2	118.7	135.6
2015	Lima	Men	<60	Healthy	121.6	120.7	122.5
2015	Lima	Men	<60	Unaware	145.9	144.0	147.7
2015	Lima	Men	<60	Aware not treated	135.3	128.6	142.0
2015	Lima	Men	<60	Aware treated	142.9	133.2	152.5
2015	Lima	Men	60+	Healthy	121.6	119.3	123.8
2015	Lima	Men	60+	Unaware	159.6	152.8	166.5
2015	Lima	Men	60+	Aware not treated	137.6	128.0	147.2
2015	Lima	Men	60+	Aware treated	149.8	142.1	157.5
2016	Lima	Men	<60	Healthy	121.3	120.4	122.2
2016	Lima	Men	<60	Unaware	149.7	146.4	153.0
2016	Lima	Men	<60	Aware not treated	135.3	125.6	145.0
2016	Lima	Men	<60	Aware treated	145.4	136.1	154.6
2016	Lima	Men	60+	Healthy	124.8	122.7	126.9
2016	Lima	Men	60+	Unaware	154.9	150.9	158.9
2016	Lima	Men	60+	Aware not treated	149.4	129.6	169.1
2016	Lima	Men	60+	Aware treated	151.1	144.4	157.8
2017	Lima	Men	<60	Healthy	120.5	119.6	121.3
2017	Lima	Men	<60	Unaware	149.4	146.5	152.2
2017	Lima	Men	<60	Aware not treated	140.4	129.5	151.2
2017	Lima	Men	<60	Aware treated	137.9	127.1	148.6
2017	Lima	Men	60+	Healthy	121.4	118.6	124.2
2017	Lima	Men	60+	Unaware	156.2	151.7	160.8
2017	Lima	Men	60+	Aware not treated	142.3	130.5	154.2
2017	Lima	Men	60+	Aware treated	142.8	135.9	149.6
2018	Lima	Men	<60	Healthy	121.9	121.1	122.7
2018	Lima	Men	<60	Unaware	148.7	146.6	150.9
2018	Lima	Men	<60	Aware not treated	143.4	122.9	163.8
2018	Lima	Men	<60	Aware treated	145.9	136.3	155.5

2018	Lima	Men	60+	Healthy	124.7	122.6	126.7
2018	Lima	Men	60+	Unaware	159.1	152.8	165.5
2018	Lima	Men	60+	Aware not treated	152.3	137.8	166.8
2018	Lima	Men	60+	Aware treated	149.4	141.3	157.4
2019	Lima	Men	<60	Healthy	121.9	121.1	122.7
2019	Lima	Men	<60	Unaware	147.9	145.0	150.7
2019	Lima	Men	<60	Aware not treated	136.1	127.5	144.6
2019	Lima	Men	<60	Aware treated	141.6	134.1	149.1
2019	Lima	Men	60+	Healthy	123.1	120.8	125.5
2019	Lima	Men	60+	Unaware	152.6	148.8	156.4
2019	Lima	Men	60+	Aware not treated	151.7	143.7	159.6
2019	Lima	Men	60+	Aware treated	143.9	138.5	149.4
2020	Lima	Men	<60	Healthy	122.5	121.5	123.5
2020	Lima	Men	<60	Unaware	150.0	146.8	153.1
2020	Lima	Men	<60	Aware not treated	138.4	131.2	145.6
2020	Lima	Men	<60	Aware treated	138.9	129.9	148.0
2020	Lima	Men	60+	Healthy	124.9	122.3	127.5
2020	Lima	Men	60+	Unaware	157.9	153.2	162.5
2020	Lima	Men	60+	Aware not treated	142.0	129.4	154.5
2020	Lima	Men	60+	Aware treated	149.3	142.2	156.4
2015	Lima	Women	<60	Healthy	111.5	110.6	112.4
2015	Lima	Women	<60	Unaware	146.5	143.4	149.5
2015	Lima	Women	<60	Aware not treated	126.3	121.1	131.5
2015	Lima	Women	<60	Aware treated	128.5	123.6	133.5
2015	Lima	Women	60+	Healthy	122.0	119.5	124.6
2015	Lima	Women	60+	Unaware	153.4	148.9	157.9
2015	Lima	Women	60+	Aware not treated	138.8	125.7	151.8
2015	Lima	Women	60+	Aware treated	149.2	141.9	156.5
2016	Lima	Women	<60	Healthy	112.7	111.7	113.7

2016	Lima	Women	<60	Unaware	148.6	145.9	151.4
2016	Lima	Women	<60	Aware not treated	122.7	113.3	132.2
2016	Lima	Women	<60	Aware treated	142.7	130.5	154.8
2016	Lima	Women	60+	Healthy	119.8	117.0	122.6
2016	Lima	Women	60+	Unaware	157.2	152.1	162.2
2016	Lima	Women	60+	Aware not treated	136.5	126.9	146.0
2016	Lima	Women	60+	Aware treated	143.6	137.8	149.3
2017	Lima	Women	<60	Healthy	112.2	111.3	113.1
2017	Lima	Women	<60	Unaware	149.2	145.7	152.6
2017	Lima	Women	<60	Aware not treated	119.8	114.9	124.7
2017	Lima	Women	<60	Aware treated	134.7	127.6	141.8
2017	Lima	Women	60+	Healthy	122.1	119.9	124.4
2017	Lima	Women	60+	Unaware	158.0	152.3	163.7
2017	Lima	Women	60+	Aware not treated	140.6	128.2	153.0
2017	Lima	Women	60+	Aware treated	145.7	139.6	151.7
2018	Lima	Women	<60	Healthy	112.9	112.1	113.8
2018	Lima	Women	<60	Unaware	149.1	144.8	153.5
2018	Lima	Women	<60	Aware not treated	129.4	121.3	137.5
2018	Lima	Women	<60	Aware treated	132.4	125.9	138.8
2018	Lima	Women	60+	Healthy	121.0	118.4	123.7
2018	Lima	Women	60+	Unaware	155.6	151.7	159.5
2018	Lima	Women	60+	Aware not treated	146.0	134.6	157.4
2018	Lima	Women	60+	Aware treated	146.6	141.3	151.9
2019	Lima	Women	<60	Healthy	112.2	111.4	113.1
2019	Lima	Women	<60	Unaware	150.5	146.8	154.1
2019	Lima	Women	<60	Aware not treated	136.0	124.7	147.3
2019	Lima	Women	<60	Aware treated	138.6	131.4	145.7
2019	Lima	Women	60+	Healthy	120.0	117.7	122.3
2019	Lima	Women	60+	Unaware	156.1	152.7	159.4

2019	Lima	Women	60+	Aware not treated	137.6	130.6	144.7
2019	Lima	Women	60+	Aware treated	143.4	136.4	150.3
2020	Lima	Women	<60	Healthy	113.9	112.6	115.1
2020	Lima	Women	<60	Unaware	148.1	144.0	152.2
2020	Lima	Women	<60	Aware not treated	123.3	114.5	132.2
2020	Lima	Women	<60	Aware treated	130.3	123.1	137.6
2020	Lima	Women	60+	Healthy	117.9	115.2	120.6
2020	Lima	Women	60+	Unaware	160.8	153.2	168.4
2020	Lima	Women	60+	Aware not treated	150.7	132.4	169.0
2020	Lima	Women	60+	Aware treated	135.5	129.7	141.3
2015	Loreto	Men	<60	Healthy	120.7	119.7	121.8
2015	Loreto	Men	<60	Unaware	147.1	141.9	152.3
2015	Loreto	Men	<60	Aware not treated	130.3	119.9	140.7
2015	Loreto	Men	<60	Aware treated	134.1	122.8	145.5
2015	Loreto	Men	60+	Healthy	118.6	113.8	123.4
2015	Loreto	Men	60+	Unaware	166.8	158.9	174.7
2015	Loreto	Men	60+	Aware not treated	113.5	106.6	120.5
2015	Loreto	Men	60+	Aware treated	140.0	130.0	150.0
2016	Loreto	Men	<60	Healthy	120.5	119.3	121.7
2016	Loreto	Men	<60	Unaware	148.8	145.8	151.8
2016	Loreto	Men	<60	Aware not treated	136.7	129.7	143.6
2016	Loreto	Men	<60	Aware treated	136.0	128.7	143.4
2016	Loreto	Men	60+	Healthy	120.1	115.7	124.4
2016	Loreto	Men	60+	Unaware	165.5	156.9	174.2
2016	Loreto	Men	60+	Aware not treated	126.5	109.8	143.3
2016	Loreto	Men	60+	Aware treated	145.7	128.3	163.1
2017	Loreto	Men	<60	Healthy	120.2	118.9	121.4
2017	Loreto	Men	<60	Unaware	147.3	143.7	150.8
2017	Loreto	Men	<60	Aware not treated	130.0	112.8	147.2

2017	Loreto	Men	<60	Aware treated	136.4	112.2	160.6
2017	Loreto	Men	60+	Healthy	121.3	117.6	124.9
2017	Loreto	Men	60+	Unaware	159.8	149.7	170.0
2017	Loreto	Men	60+	Aware not treated	140.8	126.8	154.8
2017	Loreto	Men	60+	Aware treated	145.1	132.5	157.7
2018	Loreto	Men	<60	Healthy	120.1	118.7	121.5
2018	Loreto	Men	<60	Unaware	146.1	142.6	149.6
2018	Loreto	Men	<60	Aware not treated	139.2	129.0	149.4
2018	Loreto	Men	<60	Aware treated	151.4	138.2	164.6
2018	Loreto	Men	60+	Healthy	117.2	113.5	120.9
2018	Loreto	Men	60+	Unaware	157.1	147.6	166.5
2018	Loreto	Men	60+	Aware not treated	139.0	139.0	139.0
2018	Loreto	Men	60+	Aware treated	142.5	136.0	148.9
2019	Loreto	Men	<60	Healthy	118.0	116.6	119.5
2019	Loreto	Men	<60	Unaware	149.2	145.1	153.4
2019	Loreto	Men	<60	Aware not treated	135.3	126.0	144.6
2019	Loreto	Men	<60	Aware treated	131.1	117.1	145.0
2019	Loreto	Men	60+	Healthy	120.5	117.1	124.0
2019	Loreto	Men	60+	Unaware	149.3	143.7	154.9
2019	Loreto	Men	60+	Aware not treated	135.0	122.3	147.7
2019	Loreto	Men	60+	Aware treated	133.3	125.7	140.9
2020	Loreto	Men	<60	Healthy	120.7	118.8	122.5
2020	Loreto	Men	<60	Unaware	147.3	143.7	150.9
2020	Loreto	Men	<60	Aware not treated	141.5	137.9	145.1
2020	Loreto	Men	<60	Aware treated	140.8	128.2	153.5
2020	Loreto	Men	60+	Healthy	118.0	112.6	123.5
2020	Loreto	Men	60+	Unaware	151.3	143.9	158.7
2020	Loreto	Men	60+	Aware not treated	131.5	120.3	142.7
2020	Loreto	Men	60+	Aware treated	150.3	122.5	178.0

2015	Loreto	Women	<60	Healthy	112.9	111.1	114.6
2015	Loreto	Women	<60	Unaware	150.9	145.4	156.3
2015	Loreto	Women	<60	Aware not treated	117.6	109.5	125.7
2015	Loreto	Women	<60	Aware treated	119.0	114.3	123.7
2015	Loreto	Women	60+	Healthy	114.9	108.8	121.1
2015	Loreto	Women	60+	Unaware	160.8	144.3	177.3
2015	Loreto	Women	60+	Aware not treated	135.1	112.6	157.6
2015	Loreto	Women	60+	Aware treated	132.8	124.7	141.0
2016	Loreto	Women	<60	Healthy	112.7	111.4	114.0
2016	Loreto	Women	<60	Unaware	148.4	143.6	153.1
2016	Loreto	Women	<60	Aware not treated	124.1	115.4	132.8
2016	Loreto	Women	<60	Aware treated	136.1	127.4	144.8
2016	Loreto	Women	60+	Healthy	120.4	114.9	126.0
2016	Loreto	Women	60+	Unaware	174.2	161.1	187.2
2016	Loreto	Women	60+	Aware not treated	160.4	148.3	172.5
2016	Loreto	Women	60+	Aware treated	135.7	128.3	143.0
2017	Loreto	Women	<60	Healthy	112.0	110.4	113.6
2017	Loreto	Women	<60	Unaware	148.8	144.3	153.2
2017	Loreto	Women	<60	Aware not treated	104.5	97.5	111.5
2017	Loreto	Women	<60	Aware treated	133.0	121.2	144.8
2017	Loreto	Women	60+	Healthy	119.5	115.9	123.1
2017	Loreto	Women	60+	Unaware	161.1	150.8	171.5
2017	Loreto	Women	60+	Aware not treated	141.9	112.2	171.5
2017	Loreto	Women	60+	Aware treated	143.6	136.2	151.0
2018	Loreto	Women	<60	Healthy	111.1	109.4	112.7
2018	Loreto	Women	<60	Unaware	147.3	141.5	153.1
2018	Loreto	Women	<60	Aware not treated	131.7	119.7	143.7
2018	Loreto	Women	<60	Aware treated	131.3	120.2	142.5
2018	Loreto	Women	60+	Healthy	119.7	116.0	123.5

2018	Loreto	Women	60+	Unaware	159.6	147.1	172.1
2018	Loreto	Women	60+	Aware not treated	136.6	124.9	148.3
2018	Loreto	Women	60+	Aware treated	145.0	133.4	156.6
2019	Loreto	Women	<60	Healthy	110.5	109.0	111.9
2019	Loreto	Women	<60	Unaware	147.9	140.8	155.0
2019	Loreto	Women	<60	Aware not treated	111.5	102.7	120.2
2019	Loreto	Women	<60	Aware treated	121.3	115.0	127.6
2019	Loreto	Women	60+	Healthy	117.8	113.3	122.3
2019	Loreto	Women	60+	Unaware	153.8	144.1	163.6
2019	Loreto	Women	60+	Aware not treated	162.5	131.2	193.7
2019	Loreto	Women	60+	Aware treated	148.9	139.1	158.7
2020	Loreto	Women	<60	Healthy	112.7	110.9	114.6
2020	Loreto	Women	<60	Unaware	161.3	152.1	170.5
2020	Loreto	Women	<60	Aware not treated	118.3	110.2	126.4
2020	Loreto	Women	<60	Aware treated	122.5	115.3	129.7
2020	Loreto	Women	60+	Healthy	118.7	114.9	122.4
2020	Loreto	Women	60+	Unaware	148.5	143.9	153.0
2020	Loreto	Women	60+	Aware not treated	135.5	119.0	152.1
2020	Loreto	Women	60+	Aware treated	139.1	129.9	148.4
2015	Madre de Dios	Men	<60	Healthy	118.1	116.7	119.5
2015	Madre de Dios	Men	<60	Unaware	149.0	143.9	154.2
2015	Madre de Dios	Men	<60	Aware not treated	122.7	113.1	132.3
2015	Madre de Dios	Men	<60	Aware treated	136.8	121.3	152.2
2015	Madre de Dios	Men	60+	Healthy	124.6	119.7	129.5
2015	Madre de Dios	Men	60+	Unaware	157.9	153.2	162.6
2015	Madre de Dios	Men	60+	Aware not treated	124.3	118.9	129.7
2015	Madre de Dios	Men	60+	Aware treated	134.4	118.0	150.8
2016	Madre de Dios	Men	<60	Healthy	119.0	117.7	120.3
2016	Madre de Dios	Men	<60	Unaware	145.2	142.0	148.4

2016	Madre de Dios	Men	<60	Aware not treated	126.5	116.9	136.0
2016	Madre de Dios	Men	<60	Aware treated	138.9	115.4	162.3
2016	Madre de Dios	Men	60+	Healthy	118.9	113.9	124.0
2016	Madre de Dios	Men	60+	Unaware	160.3	156.0	164.6
2016	Madre de Dios	Men	60+	Aware not treated	130.2	121.6	138.8
2016	Madre de Dios	Men	60+	Aware treated	145.5	126.6	164.4
2017	Madre de Dios	Men	<60	Healthy	120.4	118.9	121.8
2017	Madre de Dios	Men	<60	Unaware	144.5	141.0	148.0
2017	Madre de Dios	Men	<60	Aware not treated	124.5	117.0	132.0
2017	Madre de Dios	Men	<60	Aware treated	132.3	118.4	146.2
2017	Madre de Dios	Men	60+	Healthy	118.6	114.3	122.9
2017	Madre de Dios	Men	60+	Unaware	148.5	144.9	152.2
2017	Madre de Dios	Men	60+	Aware not treated	160.6	133.7	187.4
2017	Madre de Dios	Men	60+	Aware treated	140.1	125.0	155.2
2018	Madre de Dios	Men	<60	Healthy	120.2	118.6	121.7
2018	Madre de Dios	Men	<60	Unaware	142.7	138.4	147.1
2018	Madre de Dios	Men	<60	Aware not treated	127.4	120.9	134.0
2018	Madre de Dios	Men	<60	Aware treated	139.4	113.9	164.8
2018	Madre de Dios	Men	60+	Healthy	124.5	120.9	128.1
2018	Madre de Dios	Men	60+	Unaware	151.0	147.5	154.6
2018	Madre de Dios	Men	60+	Aware not treated	130.2	115.3	145.0
2018	Madre de Dios	Men	60+	Aware treated	163.3	141.1	185.5
2019	Madre de Dios	Men	<60	Healthy	118.0	116.7	119.4
2019	Madre de Dios	Men	<60	Unaware	142.0	136.8	147.3
2019	Madre de Dios	Men	<60	Aware not treated	141.1	131.0	151.2
2019	Madre de Dios	Men	<60	Aware treated	139.1	126.0	152.3
2019	Madre de Dios	Men	60+	Healthy	120.6	116.1	125.1
2019	Madre de Dios	Men	60+	Unaware	154.2	146.8	161.6
2019	Madre de Dios	Men	60+	Aware not treated	142.7	126.2	159.2

2019	Madre de Dios	Men	60+	Aware treated	128.2	117.1	139.3
2020	Madre de Dios	Men	<60	Healthy	119.1	117.5	120.8
2020	Madre de Dios	Men	<60	Unaware	145.5	140.9	150.0
2020	Madre de Dios	Men	<60	Aware not treated	134.7	124.4	145.0
2020	Madre de Dios	Men	<60	Aware treated	133.4	126.5	140.2
2020	Madre de Dios	Men	60+	Healthy	117.3	112.5	122.2
2020	Madre de Dios	Men	60+	Unaware	156.9	147.9	165.9
2020	Madre de Dios	Men	60+	Aware not treated	120.8	108.4	133.2
2020	Madre de Dios	Men	60+	Aware treated	134.8	123.3	146.4
2015	Madre de Dios	Women	<60	Healthy	107.7	106.2	109.2
2015	Madre de Dios	Women	<60	Unaware	152.7	146.2	159.3
2015	Madre de Dios	Women	<60	Aware not treated	116.4	109.9	123.0
2015	Madre de Dios	Women	<60	Aware treated	115.4	109.7	121.0
2015	Madre de Dios	Women	60+	Healthy	124.7	119.6	129.8
2015	Madre de Dios	Women	60+	Unaware	146.9	138.9	155.0
2015	Madre de Dios	Women	60+	Aware not treated	146.5	121.7	171.3
2015	Madre de Dios	Women	60+	Aware treated	141.6	123.5	159.8
2016	Madre de Dios	Women	<60	Healthy	109.1	107.5	110.6
2016	Madre de Dios	Women	<60	Unaware	145.8	144.4	147.2
2016	Madre de Dios	Women	<60	Aware not treated	116.3	104.0	128.6
2016	Madre de Dios	Women	<60	Aware treated	117.4	112.2	122.7
2016	Madre de Dios	Women	60+	Healthy	120.1	110.4	129.8
2016	Madre de Dios	Women	60+	Unaware	144.0	144.0	144.0
2016	Madre de Dios	Women	60+	Aware not treated	125.3	113.9	136.7
2016	Madre de Dios	Women	60+	Aware treated	143.0	110.1	176.0
2017	Madre de Dios	Women	<60	Healthy	109.4	107.8	111.0
2017	Madre de Dios	Women	<60	Unaware	158.7	146.8	170.7
2017	Madre de Dios	Women	<60	Aware not treated	115.3	106.4	124.3
2017	Madre de Dios	Women	<60	Aware treated	125.8	119.2	132.3

2017	Madre de Dios	Women	60+	Healthy	112.5	108.0	117.1
2017	Madre de Dios	Women	60+	Unaware	151.5	139.7	163.3
2017	Madre de Dios	Women	60+	Aware not treated	133.0	133.0	133.0
2017	Madre de Dios	Women	60+	Aware treated	146.8	117.6	176.0
2018	Madre de Dios	Women	<60	Healthy	109.6	107.9	111.3
2018	Madre de Dios	Women	<60	Unaware	150.6	135.8	165.4
2018	Madre de Dios	Women	<60	Aware not treated	119.4	113.2	125.7
2018	Madre de Dios	Women	<60	Aware treated	126.5	115.1	137.8
2018	Madre de Dios	Women	60+	Healthy	118.7	113.6	123.9
2018	Madre de Dios	Women	60+	Unaware	148.8	143.0	154.6
2018	Madre de Dios	Women	60+	Aware not treated	122.1	109.9	134.3
2018	Madre de Dios	Women	60+	Aware treated	138.3	123.3	153.4
2019	Madre de Dios	Women	<60	Healthy	109.5	107.9	111.2
2019	Madre de Dios	Women	<60	Unaware	144.0	128.7	159.3
2019	Madre de Dios	Women	<60	Aware not treated	112.3	108.2	116.5
2019	Madre de Dios	Women	<60	Aware treated	122.1	113.4	130.8
2019	Madre de Dios	Women	60+	Healthy	111.3	105.2	117.3
2019	Madre de Dios	Women	60+	Unaware	153.6	147.3	160.0
2019	Madre de Dios	Women	60+	Aware not treated	121.5	111.8	131.1
2019	Madre de Dios	Women	60+	Aware treated	147.9	131.0	164.8
2020	Madre de Dios	Women	<60	Healthy	107.0	105.6	108.4
2020	Madre de Dios	Women	<60	Unaware	147.3	130.7	163.9
2020	Madre de Dios	Women	<60	Aware not treated	114.9	104.5	125.3
2020	Madre de Dios	Women	<60	Aware treated	124.5	114.4	134.6
2020	Madre de Dios	Women	60+	Healthy	118.1	111.4	124.9
2020	Madre de Dios	Women	60+	Unaware	144.5	139.3	149.8
2020	Madre de Dios	Women	60+	Aware not treated	126.6	123.9	129.4
2020	Madre de Dios	Women	60+	Aware treated	128.8	103.7	153.9
2015	Moquegua	Men	<60	Healthy	120.6	119.0	122.2

2015	Moquegua	Men	<60	Unaware	148.7	145.5	151.8
2015	Moquegua	Men	<60	Aware not treated	132.5	125.8	139.1
2015	Moquegua	Men	<60	Aware treated	129.6	125.1	134.0
2015	Moquegua	Men	60+	Healthy	123.2	119.7	126.7
2015	Moquegua	Men	60+	Unaware	155.9	151.4	160.3
2015	Moquegua	Men	60+	Aware not treated	146.6	138.2	154.9
2015	Moquegua	Men	60+	Aware treated	153.7	134.2	173.3
2016	Moquegua	Men	<60	Healthy	120.2	118.9	121.6
2016	Moquegua	Men	<60	Unaware	149.6	141.9	157.4
2016	Moquegua	Men	<60	Aware not treated	134.6	131.7	137.5
2016	Moquegua	Men	<60	Aware treated	135.3	120.6	150.1
2016	Moquegua	Men	60+	Healthy	122.5	119.6	125.4
2016	Moquegua	Men	60+	Unaware	154.6	148.8	160.3
2016	Moquegua	Men	60+	Aware not treated	145.4	135.3	155.6
2016	Moquegua	Men	60+	Aware treated	138.0	129.0	147.0
2017	Moquegua	Men	<60	Healthy	121.3	119.9	122.7
2017	Moquegua	Men	<60	Unaware	146.3	142.5	150.0
2017	Moquegua	Men	<60	Aware not treated	154.0	127.2	180.7
2017	Moquegua	Men	<60	Aware treated	134.4	123.0	145.7
2017	Moquegua	Men	60+	Healthy	122.6	119.5	125.7
2017	Moquegua	Men	60+	Unaware	155.0	148.4	161.6
2017	Moquegua	Men	60+	Aware not treated	159.5	135.9	183.2
2017	Moquegua	Men	60+	Aware treated	130.5	117.6	143.5
2018	Moquegua	Men	<60	Healthy	120.4	119.2	121.6
2018	Moquegua	Men	<60	Unaware	144.3	142.2	146.4
2018	Moquegua	Men	<60	Aware not treated	135.5	128.8	142.2
2018	Moquegua	Men	<60	Aware treated	143.1	129.0	157.1
2018	Moquegua	Men	60+	Healthy	119.6	116.2	122.9
2018	Moquegua	Men	60+	Unaware	151.5	145.1	157.8

2018	Moquegua	Men	60+	Aware not treated	141.2	125.6	156.8
2018	Moquegua	Men	60+	Aware treated	142.5	132.5	152.5
2019	Moquegua	Men	<60	Healthy	121.4	120.0	122.8
2019	Moquegua	Men	<60	Unaware	141.7	138.0	145.5
2019	Moquegua	Men	<60	Aware not treated	133.8	130.4	137.2
2019	Moquegua	Men	<60	Aware treated	144.3	130.4	158.1
2019	Moquegua	Men	60+	Healthy	121.5	119.0	124.0
2019	Moquegua	Men	60+	Unaware	155.5	148.2	162.8
2019	Moquegua	Men	60+	Aware not treated	167.0	142.0	191.9
2019	Moquegua	Men	60+	Aware treated	140.7	131.6	149.8
2020	Moquegua	Men	<60	Healthy	123.2	121.5	124.8
2020	Moquegua	Men	<60	Unaware	144.7	140.4	148.9
2020	Moquegua	Men	<60	Aware not treated	124.6	116.5	132.6
2020	Moquegua	Men	<60	Aware treated	131.5	115.8	147.1
2020	Moquegua	Men	60+	Healthy	124.1	120.8	127.4
2020	Moquegua	Men	60+	Unaware	149.7	146.7	152.6
2020	Moquegua	Men	60+	Aware not treated	168.0	158.1	177.9
2020	Moquegua	Men	60+	Aware treated	151.2	136.0	166.5
2015	Moquegua	Women	<60	Healthy	112.0	110.3	113.7
2015	Moquegua	Women	<60	Unaware	148.4	142.2	154.6
2015	Moquegua	Women	<60	Aware not treated	113.5	101.2	125.7
2015	Moquegua	Women	<60	Aware treated	131.7	124.9	138.5
2015	Moquegua	Women	60+	Healthy	120.1	115.8	124.4
2015	Moquegua	Women	60+	Unaware	155.8	147.9	163.6
2015	Moquegua	Women	60+	Aware not treated	125.1	110.5	139.7
2015	Moquegua	Women	60+	Aware treated	137.7	130.7	144.7
2016	Moquegua	Women	<60	Healthy	110.6	109.3	112.0
2016	Moquegua	Women	<60	Unaware	160.1	152.5	167.7
2016	Moquegua	Women	<60	Aware not treated	122.4	108.3	136.5

2016	Moquegua	Women	<60	Aware treated	128.0	115.2	140.7
2016	Moquegua	Women	60+	Healthy	120.8	117.4	124.1
2016	Moquegua	Women	60+	Unaware	156.1	151.5	160.7
2016	Moquegua	Women	60+	Aware not treated	149.2	129.8	168.6
2016	Moquegua	Women	60+	Aware treated	137.5	129.5	145.5
2017	Moquegua	Women	<60	Healthy	111.0	109.7	112.2
2017	Moquegua	Women	<60	Unaware	152.6	146.9	158.4
2017	Moquegua	Women	<60	Aware not treated	128.7	118.4	139.0
2017	Moquegua	Women	<60	Aware treated	139.7	126.6	152.8
2017	Moquegua	Women	60+	Healthy	116.7	112.8	120.6
2017	Moquegua	Women	60+	Unaware	158.7	149.8	167.6
2017	Moquegua	Women	60+	Aware not treated	136.6	119.5	153.6
2017	Moquegua	Women	60+	Aware treated	145.9	131.1	160.6
2018	Moquegua	Women	<60	Healthy	111.3	109.9	112.6
2018	Moquegua	Women	<60	Unaware	139.4	133.3	145.5
2018	Moquegua	Women	<60	Aware not treated	135.6	123.7	147.5
2018	Moquegua	Women	<60	Aware treated	133.7	126.5	140.9
2018	Moquegua	Women	60+	Healthy	119.1	115.1	123.1
2018	Moquegua	Women	60+	Unaware	157.8	145.7	169.9
2018	Moquegua	Women	60+	Aware not treated	137.2	122.8	151.7
2018	Moquegua	Women	60+	Aware treated	121.6	110.0	133.1
2019	Moquegua	Women	<60	Healthy	111.7	110.4	113.0
2019	Moquegua	Women	<60	Unaware	147.0	140.3	153.6
2019	Moquegua	Women	<60	Aware not treated	119.0	110.0	128.1
2019	Moquegua	Women	<60	Aware treated	123.6	114.8	132.4
2019	Moquegua	Women	60+	Healthy	117.5	114.3	120.7
2019	Moquegua	Women	60+	Unaware	151.1	143.9	158.3
2019	Moquegua	Women	60+	Aware not treated	129.2	117.7	140.7
2019	Moquegua	Women	60+	Aware treated	129.4	119.0	139.8

2020	Moquegua	Women	<60	Healthy	114.1	112.7	115.6
2020	Moquegua	Women	<60	Unaware	143.4	140.1	146.7
2020	Moquegua	Women	<60	Aware not treated	130.9	114.8	147.0
2020	Moquegua	Women	<60	Aware treated	152.4	128.0	176.9
2020	Moquegua	Women	60+	Healthy	118.6	114.6	122.7
2020	Moquegua	Women	60+	Unaware	157.2	147.9	166.6
2020	Moquegua	Women	60+	Aware not treated	124.9	102.2	147.7
2020	Moquegua	Women	60+	Aware treated	133.6	117.7	149.6
2015	Pasco	Men	<60	Healthy	117.3	115.9	118.7
2015	Pasco	Men	<60	Unaware	147.6	145.1	150.1
2015	Pasco	Men	<60	Aware not treated	122.9	117.7	128.1
2015	Pasco	Men	<60	Aware treated	155.1	144.0	166.1
2015	Pasco	Men	60+	Healthy	123.9	119.4	128.4
2015	Pasco	Men	60+	Unaware	149.8	144.5	155.0
2015	Pasco	Men	60+	Aware not treated	142.1	134.9	149.4
2015	Pasco	Men	60+	Aware treated	166.9	164.0	169.8
2016	Pasco	Men	<60	Healthy	117.4	115.9	119.0
2016	Pasco	Men	<60	Unaware	145.8	142.2	149.4
2016	Pasco	Men	<60	Aware not treated	128.3	122.3	134.4
2016	Pasco	Men	<60	Aware treated	127.2	115.1	139.3
2016	Pasco	Men	60+	Healthy	119.2	112.7	125.6
2016	Pasco	Men	60+	Unaware	156.7	149.9	163.5
2016	Pasco	Men	60+	Aware not treated	145.9	126.5	165.3
2016	Pasco	Men	60+	Aware treated	132.6	117.4	147.7
2017	Pasco	Men	<60	Healthy	117.4	116.0	118.9
2017	Pasco	Men	<60	Unaware	146.6	143.0	150.2
2017	Pasco	Men	<60	Aware not treated	120.8	113.7	127.8
2017	Pasco	Men	<60	Aware treated	146.3	140.6	151.9
2017	Pasco	Men	60+	Healthy	119.6	116.1	123.2

2017	Pasco	Men	60+	Unaware	155.7	147.8	163.6
2017	Pasco	Men	60+	Aware not treated	134.9	121.1	148.7
2017	Pasco	Men	60+	Aware treated	136.2	130.9	141.6
2018	Pasco	Men	<60	Healthy	117.8	116.2	119.3
2018	Pasco	Men	<60	Unaware	133.9	128.8	138.9
2018	Pasco	Men	<60	Aware not treated	120.6	116.5	124.8
2018	Pasco	Men	<60	Aware treated	126.8	103.4	150.2
2018	Pasco	Men	60+	Healthy	121.3	117.7	125.0
2018	Pasco	Men	60+	Unaware	150.0	144.4	155.6
2018	Pasco	Men	60+	Aware not treated	123.1	119.9	126.3
2018	Pasco	Men	60+	Aware treated	136.1	119.1	153.0
2019	Pasco	Men	<60	Healthy	118.2	116.9	119.5
2019	Pasco	Men	<60	Unaware	148.4	142.0	154.8
2019	Pasco	Men	<60	Aware not treated	123.9	119.5	128.4
2019	Pasco	Men	<60	Aware treated	136.9	114.4	159.3
2019	Pasco	Men	60+	Healthy	120.4	117.5	123.3
2019	Pasco	Men	60+	Unaware	154.8	145.5	164.1
2019	Pasco	Men	60+	Aware not treated	153.0	153.0	153.0
2019	Pasco	Men	60+	Aware treated	127.7	117.9	137.5
2020	Pasco	Men	<60	Healthy	118.0	116.1	119.8
2020	Pasco	Men	<60	Unaware	146.7	139.5	153.9
2020	Pasco	Men	<60	Aware not treated	115.9	109.2	122.5
2020	Pasco	Men	<60	Aware treated	152.0	152.0	152.0
2020	Pasco	Men	60+	Healthy	120.9	116.5	125.3
2020	Pasco	Men	60+	Unaware	153.6	144.7	162.5
2020	Pasco	Men	60+	Aware not treated	164.6	155.4	173.7
2020	Pasco	Men	60+	Aware treated	146.7	98.7	194.8
2015	Pasco	Women	<60	Healthy	110.8	109.3	112.3
2015	Pasco	Women	<60	Unaware	146.8	141.6	151.9

2015	Pasco	Women	<60	Aware not treated	115.1	105.3	124.9
2015	Pasco	Women	<60	Aware treated	131.1	114.0	148.2
2015	Pasco	Women	60+	Healthy	118.4	114.7	122.1
2015	Pasco	Women	60+	Unaware	159.1	153.5	164.7
2015	Pasco	Women	60+	Aware not treated	144.1	132.1	156.1
2015	Pasco	Women	60+	Aware treated	142.4	120.8	163.9
2016	Pasco	Women	<60	Healthy	112.4	110.8	113.9
2016	Pasco	Women	<60	Unaware	150.7	143.2	158.2
2016	Pasco	Women	<60	Aware not treated	114.5	109.5	119.5
2016	Pasco	Women	<60	Aware treated	115.7	104.9	126.5
2016	Pasco	Women	60+	Healthy	118.7	114.6	122.8
2016	Pasco	Women	60+	Unaware	156.9	148.4	165.4
2016	Pasco	Women	60+	Aware not treated	135.8	127.9	143.8
2016	Pasco	Women	60+	Aware treated	141.1	127.8	154.5
2017	Pasco	Women	<60	Healthy	111.8	110.2	113.3
2017	Pasco	Women	<60	Unaware	145.5	141.9	149.1
2017	Pasco	Women	<60	Aware not treated	114.7	107.8	121.5
2017	Pasco	Women	<60	Aware treated	128.4	116.3	140.5
2017	Pasco	Women	60+	Healthy	119.9	116.1	123.7
2017	Pasco	Women	60+	Unaware	160.1	154.8	165.5
2017	Pasco	Women	60+	Aware not treated	125.5	114.1	136.9
2017	Pasco	Women	60+	Aware treated	143.4	119.4	167.4
2018	Pasco	Women	<60	Healthy	110.5	108.6	112.3
2018	Pasco	Women	<60	Unaware	136.2	127.5	144.9
2018	Pasco	Women	<60	Aware not treated	117.8	109.5	126.2
2018	Pasco	Women	<60	Aware treated	128.3	114.9	141.7
2018	Pasco	Women	60+	Healthy	120.2	116.6	123.8
2018	Pasco	Women	60+	Unaware	153.6	144.3	162.9
2018	Pasco	Women	60+	Aware not treated	126.8	113.2	140.5

2018	Pasco	Women	60+	Aware treated	132.2	113.8	150.6
2019	Pasco	Women	<60	Healthy	111.9	110.7	113.1
2019	Pasco	Women	<60	Unaware	146.4	142.0	150.9
2019	Pasco	Women	<60	Aware not treated	119.1	105.2	133.1
2019	Pasco	Women	<60	Aware treated	121.4	106.3	136.6
2019	Pasco	Women	60+	Healthy	120.7	118.3	123.2
2019	Pasco	Women	60+	Unaware	154.4	147.7	161.1
2019	Pasco	Women	60+	Aware not treated	127.9	111.7	144.1
2019	Pasco	Women	60+	Aware treated	136.0	119.9	152.2
2020	Pasco	Women	<60	Healthy	110.9	109.1	112.7
2020	Pasco	Women	<60	Unaware	148.4	139.2	157.5
2020	Pasco	Women	<60	Aware not treated	103.2	97.4	109.1
2020	Pasco	Women	<60	Aware treated	141.9	129.5	154.3
2020	Pasco	Women	60+	Healthy	118.4	114.2	122.5
2020	Pasco	Women	60+	Unaware	156.4	143.5	169.3
2020	Pasco	Women	60+	Aware not treated	149.3	109.4	189.2
2020	Pasco	Women	60+	Aware treated	138.2	118.4	157.9
2015	Ancash	Men	<60	Healthy	118.1	116.7	119.4
2015	Ancash	Men	<60	Unaware	143.4	140.4	146.4
2015	Ancash	Men	<60	Aware not treated	124.0	115.8	132.2
2015	Ancash	Men	<60	Aware treated	141.3	132.0	150.5
2015	Ancash	Men	60+	Healthy	119.9	116.7	123.1
2015	Ancash	Men	60+	Unaware	152.6	148.0	157.2
2015	Ancash	Men	60+	Aware not treated	158.1	137.6	178.6
2015	Ancash	Men	60+	Aware treated	137.2	126.9	147.5
2016	Ancash	Men	<60	Healthy	120.1	118.5	121.8
2016	Ancash	Men	<60	Unaware	145.1	141.5	148.8
2016	Ancash	Men	<60	Aware not treated	129.7	120.6	138.8
2016	Ancash	Men	<60	Aware treated	144.7	133.0	156.3

2016	Ancash	Men	60+	Healthy	120.9	117.7	124.1
2016	Ancash	Men	60+	Unaware	157.4	147.7	167.1
2016	Ancash	Men	60+	Aware not treated	151.6	136.4	166.8
2016	Ancash	Men	60+	Aware treated	137.1	130.0	144.2
2017	Ancash	Men	<60	Healthy	119.8	118.4	121.2
2017	Ancash	Men	<60	Unaware	152.2	146.4	157.9
2017	Ancash	Men	<60	Aware not treated	141.9	126.1	157.6
2017	Ancash	Men	<60	Aware treated	148.9	140.4	157.3
2017	Ancash	Men	60+	Healthy	121.4	118.5	124.4
2017	Ancash	Men	60+	Unaware	153.2	147.7	158.8
2017	Ancash	Men	60+	Aware not treated	137.1	112.8	161.4
2017	Ancash	Men	60+	Aware treated	148.5	134.8	162.1
2018	Ancash	Men	<60	Healthy	120.3	118.8	121.8
2018	Ancash	Men	<60	Unaware	146.9	142.4	151.3
2018	Ancash	Men	<60	Aware not treated	172.0	172.0	172.0
2018	Ancash	Men	<60	Aware treated	135.4	124.6	146.1
2018	Ancash	Men	60+	Healthy	121.9	118.5	125.2
2018	Ancash	Men	60+	Unaware	151.3	147.2	155.4
2018	Ancash	Men	60+	Aware not treated	125.5	120.4	130.6
2018	Ancash	Men	60+	Aware treated	159.6	144.3	174.8
2019	Ancash	Men	<60	Healthy	120.7	119.2	122.2
2019	Ancash	Men	<60	Unaware	146.8	142.7	150.9
2019	Ancash	Men	<60	Aware not treated	131.0	112.9	149.1
2019	Ancash	Men	<60	Aware treated	136.5	122.6	150.4
2019	Ancash	Men	60+	Healthy	123.6	120.7	126.5
2019	Ancash	Men	60+	Unaware	154.3	148.0	160.6
2019	Ancash	Men	60+	Aware not treated	157.0	157.0	157.0
2019	Ancash	Men	60+	Aware treated	142.9	135.8	149.9
2020	Ancash	Men	<60	Healthy	123.0	121.4	124.6

2020	Ancash	Men	<60	Unaware	149.2	145.3	153.2
2020	Ancash	Men	<60	Aware not treated	115.0	115.0	115.0
2020	Ancash	Men	<60	Aware treated	134.0	134.0	134.0
2020	Ancash	Men	60+	Healthy	124.5	120.9	128.1
2020	Ancash	Men	60+	Unaware	153.8	147.1	160.4
2020	Ancash	Men	60+	Aware not treated	145.0	145.0	145.0
2020	Ancash	Men	60+	Aware treated	155.2	138.2	172.3
2015	Ancash	Women	<60	Healthy	111.2	109.9	112.6
2015	Ancash	Women	<60	Unaware	148.1	135.9	160.2
2015	Ancash	Women	<60	Aware not treated	117.1	103.4	130.7
2015	Ancash	Women	<60	Aware treated	120.8	107.8	133.7
2015	Ancash	Women	60+	Healthy	118.5	114.0	123.0
2015	Ancash	Women	60+	Unaware	154.6	149.6	159.5
2015	Ancash	Women	60+	Aware not treated	136.6	118.3	154.9
2015	Ancash	Women	60+	Aware treated	146.2	139.4	153.0
2016	Ancash	Women	<60	Healthy	111.7	110.1	113.3
2016	Ancash	Women	<60	Unaware	146.5	143.5	149.6
2016	Ancash	Women	<60	Aware not treated	129.1	96.2	162.0
2016	Ancash	Women	<60	Aware treated	134.1	125.6	142.6
2016	Ancash	Women	60+	Healthy	116.6	112.8	120.4
2016	Ancash	Women	60+	Unaware	157.0	150.7	163.4
2016	Ancash	Women	60+	Aware not treated	139.2	108.0	170.4
2016	Ancash	Women	60+	Aware treated	153.4	138.5	168.3
2017	Ancash	Women	<60	Healthy	113.2	111.9	114.5
2017	Ancash	Women	<60	Unaware	151.6	143.5	159.7
2017	Ancash	Women	<60	Aware not treated	140.8	118.5	163.1
2017	Ancash	Women	<60	Aware treated	121.7	109.8	133.7
2017	Ancash	Women	60+	Healthy	119.3	116.1	122.4
2017	Ancash	Women	60+	Unaware	155.2	151.4	159.0

2017	Ancash	Women	60+	Aware not treated	132.2	107.0	157.3
2017	Ancash	Women	60+	Aware treated	150.9	137.7	164.2
2018	Ancash	Women	<60	Healthy	110.5	109.2	111.9
2018	Ancash	Women	<60	Unaware	156.5	146.4	166.5
2018	Ancash	Women	<60	Aware not treated	122.6	113.1	132.2
2018	Ancash	Women	<60	Aware treated	124.1	114.5	133.7
2018	Ancash	Women	60+	Healthy	118.1	114.4	121.8
2018	Ancash	Women	60+	Unaware	157.9	151.6	164.1
2018	Ancash	Women	60+	Aware not treated	129.5	95.4	163.7
2018	Ancash	Women	60+	Aware treated	147.0	137.3	156.8
2019	Ancash	Women	<60	Healthy	111.1	109.8	112.3
2019	Ancash	Women	<60	Unaware	148.7	144.0	153.5
2019	Ancash	Women	<60	Aware not treated	128.1	86.7	169.4
2019	Ancash	Women	<60	Aware treated	119.5	111.5	127.5
2019	Ancash	Women	60+	Healthy	120.1	117.1	123.1
2019	Ancash	Women	60+	Unaware	156.1	147.4	164.8
2019	Ancash	Women	60+	Aware not treated	144.1	127.8	160.4
2019	Ancash	Women	60+	Aware treated	134.6	128.4	140.8
2020	Ancash	Women	<60	Healthy	113.9	112.0	115.7
2020	Ancash	Women	<60	Unaware	141.9	137.0	146.7
2020	Ancash	Women	<60	Aware not treated	128.9	108.4	149.4
2020	Ancash	Women	<60	Aware treated	151.6	136.4	166.9
2020	Ancash	Women	60+	Healthy	117.3	112.7	121.9
2020	Ancash	Women	60+	Unaware	162.0	153.2	170.9
2020	Ancash	Women	60+	Aware not treated	149.0	149.0	149.0
2020	Ancash	Women	60+	Aware treated	145.3	136.1	154.5
2015	Piura	Men	<60	Healthy	120.9	119.0	122.7
2015	Piura	Men	<60	Unaware	149.2	144.6	153.7
2015	Piura	Men	<60	Aware not treated	147.7	122.6	172.8

2015	Piura	Men	<60	Aware treated	146.5	135.2	157.8
2015	Piura	Men	60+	Healthy	121.2	115.7	126.7
2015	Piura	Men	60+	Unaware	158.5	150.5	166.5
2015	Piura	Men	60+	Aware not treated	134.0	134.0	134.0
2015	Piura	Men	60+	Aware treated	146.2	132.4	160.0
2016	Piura	Men	<60	Healthy	119.8	118.2	121.3
2016	Piura	Men	<60	Unaware	148.0	145.1	150.9
2016	Piura	Men	<60	Aware not treated	118.5	112.7	124.3
2016	Piura	Men	<60	Aware treated	141.2	127.0	155.4
2016	Piura	Men	60+	Healthy	123.1	119.9	126.3
2016	Piura	Men	60+	Unaware	161.8	148.8	174.7
2016	Piura	Men	60+	Aware not treated	155.0	155.0	155.0
2016	Piura	Men	60+	Aware treated	141.4	133.0	149.9
2017	Piura	Men	<60	Healthy	120.4	119.0	121.7
2017	Piura	Men	<60	Unaware	147.4	144.3	150.5
2017	Piura	Men	<60	Aware not treated	125.0	125.0	125.0
2017	Piura	Men	<60	Aware treated	140.9	119.0	162.9
2017	Piura	Men	60+	Healthy	119.9	115.7	124.0
2017	Piura	Men	60+	Unaware	159.0	152.9	165.1
2017	Piura	Men	60+	Aware not treated	150.9	129.7	172.0
2017	Piura	Men	60+	Aware treated	128.1	122.6	133.6
2018	Piura	Men	<60	Healthy	120.3	118.8	121.8
2018	Piura	Men	<60	Unaware	151.1	147.7	154.4
2018	Piura	Men	<60	Aware not treated	130.3	120.8	139.9
2018	Piura	Men	<60	Aware treated	140.5	131.0	150.0
2018	Piura	Men	60+	Healthy	122.8	118.5	127.2
2018	Piura	Men	60+	Unaware	155.7	147.3	164.1
2018	Piura	Men	60+	Aware not treated	143.8	122.2	165.5
2018	Piura	Men	60+	Aware treated	143.0	130.1	155.8

2019	Piura	Men	<60	Healthy	120.8	119.4	122.3
2019	Piura	Men	<60	Unaware	148.4	146.0	150.9
2019	Piura	Men	<60	Aware not treated	150.7	126.8	174.6
2019	Piura	Men	<60	Aware treated	141.8	122.9	160.7
2019	Piura	Men	60+	Healthy	120.0	115.3	124.6
2019	Piura	Men	60+	Unaware	152.3	145.6	159.1
2019	Piura	Men	60+	Aware not treated	162.9	158.7	167.2
2019	Piura	Men	60+	Aware treated	139.0	123.4	154.6
2020	Piura	Men	<60	Healthy	121.1	119.2	122.9
2020	Piura	Men	<60	Unaware	150.2	146.6	153.8
2020	Piura	Men	<60	Aware not treated	145.0	145.0	145.0
2020	Piura	Men	<60	Aware treated	129.3	117.7	140.9
2020	Piura	Men	60+	Healthy	125.5	121.7	129.3
2020	Piura	Men	60+	Unaware	151.6	143.9	159.3
2020	Piura	Men	60+	Aware treated	142.6	132.4	152.8
2015	Piura	Women	<60	Healthy	112.4	110.8	114.0
2015	Piura	Women	<60	Unaware	152.5	145.7	159.3
2015	Piura	Women	<60	Aware not treated	118.9	109.7	128.2
2015	Piura	Women	<60	Aware treated	156.4	146.6	166.2
2015	Piura	Women	60+	Healthy	124.3	120.2	128.4
2015	Piura	Women	60+	Unaware	154.0	147.4	160.6
2015	Piura	Women	60+	Aware not treated	138.5	110.4	166.5
2015	Piura	Women	60+	Aware treated	145.8	136.9	154.6
2016	Piura	Women	<60	Healthy	110.8	109.2	112.4
2016	Piura	Women	<60	Unaware	149.4	144.1	154.8
2016	Piura	Women	<60	Aware not treated	130.7	114.3	147.1
2016	Piura	Women	<60	Aware treated	139.5	131.5	147.5
2016	Piura	Women	60+	Healthy	118.9	116.1	121.7
2016	Piura	Women	60+	Unaware	149.8	145.8	153.7

2016	Piura	Women	60+	Aware not treated	161.3	150.2	172.4
2016	Piura	Women	60+	Aware treated	142.8	134.6	151.0
2017	Piura	Women	<60	Healthy	111.7	110.2	113.1
2017	Piura	Women	<60	Unaware	146.3	140.9	151.8
2017	Piura	Women	<60	Aware not treated	133.7	113.2	154.2
2017	Piura	Women	<60	Aware treated	136.1	123.6	148.7
2017	Piura	Women	60+	Healthy	118.7	114.9	122.6
2017	Piura	Women	60+	Unaware	153.3	148.3	158.2
2017	Piura	Women	60+	Aware not treated	133.9	101.2	166.7
2017	Piura	Women	60+	Aware treated	140.3	131.2	149.4
2018	Piura	Women	<60	Healthy	110.8	109.2	112.3
2018	Piura	Women	<60	Unaware	153.1	147.7	158.6
2018	Piura	Women	<60	Aware not treated	125.7	109.1	142.2
2018	Piura	Women	<60	Aware treated	129.9	122.5	137.4
2018	Piura	Women	60+	Healthy	122.4	118.0	126.8
2018	Piura	Women	60+	Unaware	152.3	147.1	157.5
2018	Piura	Women	60+	Aware not treated	131.3	118.1	144.4
2018	Piura	Women	60+	Aware treated	132.6	119.9	145.4
2019	Piura	Women	<60	Healthy	111.7	110.0	113.3
2019	Piura	Women	<60	Unaware	155.3	149.4	161.2
2019	Piura	Women	<60	Aware not treated	121.4	113.7	129.2
2019	Piura	Women	<60	Aware treated	138.5	127.1	149.9
2019	Piura	Women	60+	Healthy	118.9	114.5	123.2
2019	Piura	Women	60+	Unaware	159.0	150.0	167.9
2019	Piura	Women	60+	Aware not treated	150.9	125.2	176.5
2019	Piura	Women	60+	Aware treated	146.5	138.6	154.4
2020	Piura	Women	<60	Healthy	113.2	111.8	114.7
2020	Piura	Women	<60	Unaware	152.2	146.0	158.3
2020	Piura	Women	<60	Aware not treated	123.2	110.2	136.1

2020	Piura	Women	<60	Aware treated	133.2	124.9	141.4
2020	Piura	Women	60+	Healthy	117.1	110.8	123.4
2020	Piura	Women	60+	Unaware	149.7	145.8	153.6
2020	Piura	Women	60+	Aware not treated	153.6	136.0	171.1
2020	Piura	Women	60+	Aware treated	146.9	131.0	162.8
2015	Puno	Men	<60	Healthy	116.5	114.8	118.2
2015	Puno	Men	<60	Unaware	150.4	145.2	155.7
2015	Puno	Men	<60	Aware not treated	126.4	116.1	136.7
2015	Puno	Men	<60	Aware treated	129.0	129.0	129.0
2015	Puno	Men	60+	Healthy	116.9	113.6	120.3
2015	Puno	Men	60+	Unaware	150.1	144.8	155.4
2015	Puno	Men	60+	Aware not treated	127.0	117.4	136.5
2015	Puno	Men	60+	Aware treated	134.4	120.8	147.9
2016	Puno	Men	<60	Healthy	118.6	116.8	120.3
2016	Puno	Men	<60	Unaware	142.9	138.7	147.2
2016	Puno	Men	<60	Aware not treated	126.7	118.2	135.2
2016	Puno	Men	<60	Aware treated	156.4	135.2	177.7
2016	Puno	Men	60+	Healthy	118.5	115.9	121.2
2016	Puno	Men	60+	Unaware	153.9	148.2	159.6
2016	Puno	Men	60+	Aware not treated	154.6	106.7	202.6
2016	Puno	Men	60+	Aware treated	144.6	117.7	171.4
2017	Puno	Men	<60	Healthy	119.0	117.4	120.5
2017	Puno	Men	<60	Unaware	146.5	141.4	151.6
2017	Puno	Men	<60	Aware not treated	122.7	119.5	125.9
2017	Puno	Men	<60	Aware treated	141.3	123.1	159.4
2017	Puno	Men	60+	Healthy	119.0	116.1	121.8
2017	Puno	Men	60+	Unaware	156.2	149.4	163.0
2017	Puno	Men	60+	Aware not treated	118.2	106.8	129.7
2017	Puno	Men	60+	Aware treated	134.5	123.7	145.4

2018	Puno	Men	<60	Healthy	118.0	116.3	119.6
2018	Puno	Men	<60	Unaware	144.8	139.6	150.1
2018	Puno	Men	<60	Aware not treated	118.8	111.5	126.1
2018	Puno	Men	<60	Aware treated	117.1	112.1	122.1
2018	Puno	Men	60+	Healthy	118.9	114.9	123.0
2018	Puno	Men	60+	Unaware	148.4	144.4	152.5
2018	Puno	Men	60+	Aware not treated	123.2	112.8	133.6
2018	Puno	Men	60+	Aware treated	154.0	136.6	171.4
2019	Puno	Men	<60	Healthy	118.2	116.6	119.9
2019	Puno	Men	<60	Unaware	143.5	139.1	147.8
2019	Puno	Men	<60	Aware not treated	120.3	116.2	124.4
2019	Puno	Men	<60	Aware treated	126.6	110.6	142.6
2019	Puno	Men	60+	Healthy	120.3	117.1	123.5
2019	Puno	Men	60+	Unaware	156.5	151.4	161.7
2019	Puno	Men	60+	Aware not treated	117.9	106.6	129.2
2019	Puno	Men	60+	Aware treated	135.9	124.6	147.2
2020	Puno	Men	<60	Healthy	121.1	119.0	123.3
2020	Puno	Men	<60	Unaware	142.2	140.3	144.0
2020	Puno	Men	<60	Aware not treated	129.4	117.1	141.7
2020	Puno	Men	<60	Aware treated	134.7	120.7	148.8
2020	Puno	Men	60+	Healthy	121.1	118.4	123.8
2020	Puno	Men	60+	Unaware	154.9	146.9	162.9
2020	Puno	Men	60+	Aware not treated	141.8	105.1	178.5
2020	Puno	Men	60+	Aware treated	144.8	124.6	165.0
2015	Puno	Women	<60	Healthy	110.9	109.7	112.1
2015	Puno	Women	<60	Unaware	142.8	137.4	148.2
2015	Puno	Women	<60	Aware not treated	117.6	109.5	125.6
2015	Puno	Women	<60	Aware treated	122.5	104.7	140.4
2015	Puno	Women	60+	Healthy	116.6	114.5	118.7

2015	Puno	Women	60+	Unaware	158.7	151.7	165.7
2015	Puno	Women	60+	Aware not treated	147.1	123.5	170.7
2015	Puno	Women	60+	Aware treated	142.4	123.0	161.7
2016	Puno	Women	<60	Healthy	111.2	109.8	112.6
2016	Puno	Women	<60	Unaware	146.9	141.9	151.9
2016	Puno	Women	<60	Aware not treated	116.9	110.8	123.0
2016	Puno	Women	<60	Aware treated	120.6	114.5	126.6
2016	Puno	Women	60+	Healthy	116.3	113.1	119.4
2016	Puno	Women	60+	Unaware	157.6	149.7	165.6
2016	Puno	Women	60+	Aware not treated	124.6	108.2	141.0
2016	Puno	Women	60+	Aware treated	141.0	120.3	161.6
2017	Puno	Women	<60	Healthy	111.6	110.3	113.0
2017	Puno	Women	<60	Unaware	157.2	145.9	168.6
2017	Puno	Women	<60	Aware not treated	110.3	105.7	115.0
2017	Puno	Women	<60	Aware treated	134.0	110.6	157.5
2017	Puno	Women	60+	Healthy	115.8	113.4	118.2
2017	Puno	Women	60+	Unaware	164.5	151.4	177.6
2017	Puno	Women	60+	Aware not treated	140.9	122.0	159.8
2017	Puno	Women	60+	Aware treated	126.6	119.4	133.9
2018	Puno	Women	<60	Healthy	111.6	110.0	113.2
2018	Puno	Women	<60	Unaware	148.3	141.4	155.3
2018	Puno	Women	<60	Aware not treated	121.3	112.8	129.8
2018	Puno	Women	<60	Aware treated	131.2	114.3	148.1
2018	Puno	Women	60+	Healthy	117.7	114.7	120.8
2018	Puno	Women	60+	Unaware	152.6	141.4	163.8
2018	Puno	Women	60+	Aware not treated	124.4	111.6	137.2
2018	Puno	Women	60+	Aware treated	145.2	132.1	158.3
2019	Puno	Women	<60	Healthy	112.7	111.4	114.1
2019	Puno	Women	<60	Unaware	151.0	137.3	164.7

2019	Puno	Women	<60	Aware not treated	130.6	120.3	140.9
2019	Puno	Women	<60	Aware treated	125.3	111.5	139.0
2019	Puno	Women	60+	Healthy	118.5	116.1	121.0
2019	Puno	Women	60+	Unaware	162.7	156.2	169.2
2019	Puno	Women	60+	Aware not treated	143.3	132.5	154.0
2019	Puno	Women	60+	Aware treated	137.3	129.4	145.3
2020	Puno	Women	<60	Healthy	114.3	112.7	115.9
2020	Puno	Women	<60	Unaware	154.1	142.6	165.6
2020	Puno	Women	<60	Aware not treated	127.6	111.5	143.8
2020	Puno	Women	<60	Aware treated	138.7	110.6	166.8
2020	Puno	Women	60+	Healthy	118.7	115.3	122.1
2020	Puno	Women	60+	Unaware	149.6	145.9	153.3
2020	Puno	Women	60+	Aware not treated	143.9	127.4	160.4
2020	Puno	Women	60+	Aware treated	137.7	128.4	147.1
2015	San Martin	Men	<60	Healthy	117.7	116.4	118.9
2015	San Martin	Men	<60	Unaware	149.5	146.4	152.6
2015	San Martin	Men	<60	Aware not treated	124.1	114.4	133.8
2015	San Martin	Men	<60	Aware treated	131.1	119.6	142.7
2015	San Martin	Men	60+	Healthy	118.4	114.5	122.3
2015	San Martin	Men	60+	Unaware	160.8	151.9	169.8
2015	San Martin	Men	60+	Aware not treated	146.7	134.1	159.3
2015	San Martin	Men	60+	Aware treated	152.6	139.8	165.5
2016	San Martin	Men	<60	Healthy	118.1	116.6	119.6
2016	San Martin	Men	<60	Unaware	155.6	145.9	165.3
2016	San Martin	Men	<60	Aware not treated	129.5	115.9	143.0
2016	San Martin	Men	<60	Aware treated	136.0	127.0	144.9
2016	San Martin	Men	60+	Healthy	115.0	109.6	120.5
2016	San Martin	Men	60+	Unaware	155.1	148.8	161.5
2016	San Martin	Men	60+	Aware treated	149.4	135.4	163.3

2017	San Martin	Men	<60	Healthy	118.2	116.7	119.6
2017	San Martin	Men	<60	Unaware	148.5	144.0	152.9
2017	San Martin	Men	<60	Aware not treated	121.7	108.9	134.5
2017	San Martin	Men	<60	Aware treated	139.1	127.2	150.9
2017	San Martin	Men	60+	Healthy	120.7	116.7	124.7
2017	San Martin	Men	60+	Unaware	150.3	145.8	154.7
2017	San Martin	Men	60+	Aware not treated	172.3	131.5	213.0
2017	San Martin	Men	60+	Aware treated	144.0	131.9	156.1
2018	San Martin	Men	<60	Healthy	118.9	117.6	120.3
2018	San Martin	Men	<60	Unaware	148.9	145.0	152.9
2018	San Martin	Men	<60	Aware not treated	136.4	120.6	152.3
2018	San Martin	Men	<60	Aware treated	134.1	119.5	148.7
2018	San Martin	Men	60+	Healthy	119.0	113.8	124.3
2018	San Martin	Men	60+	Unaware	150.8	144.3	157.3
2018	San Martin	Men	60+	Aware not treated	141.1	113.0	169.2
2018	San Martin	Men	60+	Aware treated	156.2	142.5	169.9
2019	San Martin	Men	<60	Healthy	118.9	117.5	120.2
2019	San Martin	Men	<60	Unaware	150.3	144.6	156.1
2019	San Martin	Men	<60	Aware not treated	138.2	109.5	166.8
2019	San Martin	Men	<60	Aware treated	149.8	135.7	163.9
2019	San Martin	Men	60+	Healthy	120.5	116.1	124.8
2019	San Martin	Men	60+	Unaware	158.9	143.6	174.3
2019	San Martin	Men	60+	Aware not treated	119.0	119.0	119.0
2019	San Martin	Men	60+	Aware treated	144.8	127.6	162.0
2020	San Martin	Men	<60	Healthy	119.3	117.8	120.8
2020	San Martin	Men	<60	Unaware	150.2	145.1	155.2
2020	San Martin	Men	<60	Aware not treated	136.7	121.5	152.0
2020	San Martin	Men	<60	Aware treated	142.4	124.6	160.2
2020	San Martin	Men	60+	Healthy	120.8	116.6	125.1

2020	San Martin	Men	60+	Unaware	161.8	153.1	170.6
2020	San Martin	Men	60+	Aware not treated	141.8	114.3	169.2
2020	San Martin	Men	60+	Aware treated	146.8	123.6	170.0
2015	San Martin	Women	<60	Healthy	110.7	109.1	112.3
2015	San Martin	Women	<60	Unaware	153.0	146.4	159.6
2015	San Martin	Women	<60	Aware not treated	123.5	115.3	131.8
2015	San Martin	Women	<60	Aware treated	137.5	125.2	149.7
2015	San Martin	Women	60+	Healthy	121.5	117.7	125.2
2015	San Martin	Women	60+	Unaware	151.5	142.8	160.2
2015	San Martin	Women	60+	Aware not treated	136.8	120.9	152.8
2015	San Martin	Women	60+	Aware treated	143.8	131.6	156.1
2016	San Martin	Women	<60	Healthy	111.2	109.8	112.7
2016	San Martin	Women	<60	Unaware	156.8	147.0	166.6
2016	San Martin	Women	<60	Aware not treated	117.0	104.4	129.5
2016	San Martin	Women	<60	Aware treated	133.2	120.6	145.8
2016	San Martin	Women	60+	Healthy	114.9	108.9	120.8
2016	San Martin	Women	60+	Unaware	146.9	139.9	153.9
2016	San Martin	Women	60+	Aware not treated	131.9	115.4	148.3
2016	San Martin	Women	60+	Aware treated	150.6	141.5	159.6
2017	San Martin	Women	<60	Healthy	111.0	109.5	112.4
2017	San Martin	Women	<60	Unaware	162.0	152.0	171.9
2017	San Martin	Women	<60	Aware not treated	125.3	112.8	137.8
2017	San Martin	Women	<60	Aware treated	142.7	130.7	154.7
2017	San Martin	Women	60+	Healthy	121.1	118.4	123.9
2017	San Martin	Women	60+	Unaware	161.8	155.5	168.1
2017	San Martin	Women	60+	Aware not treated	140.2	134.9	145.5
2017	San Martin	Women	60+	Aware treated	140.2	129.3	151.1
2018	San Martin	Women	<60	Healthy	108.9	107.3	110.4
2018	San Martin	Women	<60	Unaware	145.7	139.3	152.1

2018	San Martin	Women	<60	Aware not treated	123.5	110.7	136.3
2018	San Martin	Women	<60	Aware treated	134.9	123.1	146.6
2018	San Martin	Women	60+	Healthy	120.9	116.5	125.3
2018	San Martin	Women	60+	Unaware	163.6	153.8	173.4
2018	San Martin	Women	60+	Aware not treated	141.7	97.7	185.6
2018	San Martin	Women	60+	Aware treated	149.2	138.4	160.0
2019	San Martin	Women	<60	Healthy	111.8	110.4	113.1
2019	San Martin	Women	<60	Unaware	158.7	149.1	168.2
2019	San Martin	Women	<60	Aware not treated	137.0	126.7	147.3
2019	San Martin	Women	<60	Aware treated	138.5	127.7	149.3
2019	San Martin	Women	60+	Healthy	123.2	119.3	127.1
2019	San Martin	Women	60+	Unaware	149.8	144.3	155.4
2019	San Martin	Women	60+	Aware not treated	119.5	99.7	139.3
2019	San Martin	Women	60+	Aware treated	150.6	141.5	159.7
2020	San Martin	Women	<60	Healthy	112.0	110.5	113.6
2020	San Martin	Women	<60	Unaware	156.2	148.2	164.2
2020	San Martin	Women	<60	Aware not treated	119.3	112.8	125.7
2020	San Martin	Women	<60	Aware treated	123.2	114.5	131.9
2020	San Martin	Women	60+	Healthy	113.1	106.9	119.3
2020	San Martin	Women	60+	Unaware	159.1	151.4	166.9
2020	San Martin	Women	60+	Aware not treated	130.3	93.0	167.5
2020	San Martin	Women	60+	Aware treated	150.3	138.4	162.1
2015	Tacna	Men	<60	Healthy	121.5	120.1	122.9
2015	Tacna	Men	<60	Unaware	145.2	142.9	147.5
2015	Tacna	Men	<60	Aware not treated	130.6	120.0	141.2
2015	Tacna	Men	<60	Aware treated	130.3	125.4	135.3
2015	Tacna	Men	60+	Healthy	126.3	123.1	129.6
2015	Tacna	Men	60+	Unaware	154.7	149.5	159.9
2015	Tacna	Men	60+	Aware not treated	144.3	129.4	159.3

2015	Tacna	Men	60+	Aware treated	145.2	130.7	159.8
2016	Tacna	Men	<60	Healthy	123.5	122.0	125.0
2016	Tacna	Men	<60	Unaware	145.6	141.7	149.5
2016	Tacna	Men	<60	Aware not treated	135.6	122.2	148.9
2016	Tacna	Men	<60	Aware treated	132.8	128.4	137.3
2016	Tacna	Men	60+	Healthy	121.6	118.0	125.3
2016	Tacna	Men	60+	Unaware	154.0	148.1	159.9
2016	Tacna	Men	60+	Aware not treated	145.9	126.9	164.9
2016	Tacna	Men	60+	Aware treated	138.5	124.3	152.6
2017	Tacna	Men	<60	Healthy	124.0	122.6	125.4
2017	Tacna	Men	<60	Unaware	147.2	144.1	150.4
2017	Tacna	Men	<60	Aware not treated	135.2	125.7	144.7
2017	Tacna	Men	<60	Aware treated	150.6	135.3	165.9
2017	Tacna	Men	60+	Healthy	123.4	120.4	126.4
2017	Tacna	Men	60+	Unaware	151.0	148.0	154.0
2017	Tacna	Men	60+	Aware not treated	163.3	145.1	181.6
2017	Tacna	Men	60+	Aware treated	151.3	139.4	163.2
2018	Tacna	Men	<60	Healthy	121.0	119.8	122.1
2018	Tacna	Men	<60	Unaware	141.7	138.7	144.7
2018	Tacna	Men	<60	Aware not treated	143.1	124.5	161.7
2018	Tacna	Men	<60	Aware treated	140.0	136.8	143.2
2018	Tacna	Men	60+	Healthy	123.6	120.4	126.8
2018	Tacna	Men	60+	Unaware	151.5	144.8	158.2
2018	Tacna	Men	60+	Aware not treated	148.4	121.0	175.8
2018	Tacna	Men	60+	Aware treated	156.1	143.8	168.3
2019	Tacna	Men	<60	Healthy	124.4	123.1	125.6
2019	Tacna	Men	<60	Unaware	144.8	141.3	148.3
2019	Tacna	Men	<60	Aware not treated	130.0	122.0	138.1
2019	Tacna	Men	<60	Aware treated	138.3	128.3	148.3

2019	Tacna	Men	60+	Healthy	123.3	120.5	126.0
2019	Tacna	Men	60+	Unaware	153.3	140.9	165.8
2019	Tacna	Men	60+	Aware not treated	148.8	137.5	160.1
2019	Tacna	Men	60+	Aware treated	146.9	134.4	159.4
2020	Tacna	Men	<60	Healthy	120.3	118.4	122.1
2020	Tacna	Men	<60	Unaware	150.1	143.9	156.3
2020	Tacna	Men	<60	Aware not treated	142.5	127.7	157.4
2020	Tacna	Men	<60	Aware treated	134.6	133.3	136.0
2020	Tacna	Men	60+	Healthy	123.9	120.2	127.5
2020	Tacna	Men	60+	Unaware	147.4	144.2	150.7
2020	Tacna	Men	60+	Aware not treated	138.1	95.7	180.4
2020	Tacna	Men	60+	Aware treated	143.5	128.5	158.6
2015	Tacna	Women	<60	Healthy	114.8	113.2	116.4
2015	Tacna	Women	<60	Unaware	142.9	140.1	145.7
2015	Tacna	Women	<60	Aware not treated	120.8	115.5	126.0
2015	Tacna	Women	<60	Aware treated	135.8	112.6	158.9
2015	Tacna	Women	60+	Healthy	121.1	117.0	125.1
2015	Tacna	Women	60+	Unaware	149.3	143.8	154.8
2015	Tacna	Women	60+	Aware not treated	148.0	133.0	163.0
2015	Tacna	Women	60+	Aware treated	134.3	123.7	144.9
2016	Tacna	Women	<60	Healthy	115.4	114.1	116.6
2016	Tacna	Women	<60	Unaware	145.7	141.7	149.7
2016	Tacna	Women	<60	Aware not treated	117.4	111.5	123.2
2016	Tacna	Women	<60	Aware treated	134.8	119.1	150.6
2016	Tacna	Women	60+	Healthy	118.8	115.0	122.6
2016	Tacna	Women	60+	Unaware	151.3	145.4	157.1
2016	Tacna	Women	60+	Aware not treated	152.6	134.4	170.8
2016	Tacna	Women	60+	Aware treated	145.1	134.7	155.6
2017	Tacna	Women	<60	Healthy	114.6	112.8	116.3

2017	Tacna	Women	<60	Unaware	145.4	129.7	161.1
2017	Tacna	Women	<60	Aware not treated	124.4	118.0	130.8
2017	Tacna	Women	<60	Aware treated	130.8	119.3	142.2
2017	Tacna	Women	60+	Healthy	121.2	118.0	124.4
2017	Tacna	Women	60+	Unaware	154.2	145.0	163.4
2017	Tacna	Women	60+	Aware not treated	142.5	136.6	148.4
2017	Tacna	Women	60+	Aware treated	139.4	128.6	150.3
2018	Tacna	Women	<60	Healthy	112.5	111.1	113.9
2018	Tacna	Women	<60	Unaware	143.0	136.6	149.4
2018	Tacna	Women	<60	Aware not treated	124.8	112.8	136.8
2018	Tacna	Women	<60	Aware treated	129.5	119.6	139.4
2018	Tacna	Women	60+	Healthy	119.0	115.2	122.8
2018	Tacna	Women	60+	Unaware	151.9	143.4	160.4
2018	Tacna	Women	60+	Aware not treated	128.5	113.6	143.4
2018	Tacna	Women	60+	Aware treated	127.3	114.1	140.5
2019	Tacna	Women	<60	Healthy	113.5	112.1	115.0
2019	Tacna	Women	<60	Unaware	142.5	137.1	147.9
2019	Tacna	Women	<60	Aware not treated	129.3	120.2	138.5
2019	Tacna	Women	<60	Aware treated	130.4	116.7	144.2
2019	Tacna	Women	60+	Healthy	123.5	119.9	127.1
2019	Tacna	Women	60+	Unaware	152.0	148.3	155.7
2019	Tacna	Women	60+	Aware not treated	135.2	118.2	152.2
2019	Tacna	Women	60+	Aware treated	140.7	132.4	149.0
2020	Tacna	Women	<60	Healthy	113.1	111.3	114.8
2020	Tacna	Women	<60	Unaware	146.8	138.7	154.9
2020	Tacna	Women	<60	Aware not treated	123.3	116.2	130.5
2020	Tacna	Women	<60	Aware treated	125.4	112.9	137.8
2020	Tacna	Women	60+	Healthy	120.0	114.6	125.4
2020	Tacna	Women	60+	Unaware	155.6	150.3	161.0

2020	Tacna	Women	60+	Aware not treated	132.4	114.3	150.5
2020	Tacna	Women	60+	Aware treated	144.1	135.5	152.7
2015	Tumbes	Men	<60	Healthy	119.8	118.4	121.2
2015	Tumbes	Men	<60	Unaware	146.5	144.2	148.9
2015	Tumbes	Men	<60	Aware not treated	134.8	123.4	146.2
2015	Tumbes	Men	<60	Aware treated	143.3	135.2	151.4
2015	Tumbes	Men	60+	Healthy	120.9	116.9	125.0
2015	Tumbes	Men	60+	Unaware	160.1	150.4	169.7
2015	Tumbes	Men	60+	Aware not treated	129.0	129.0	129.0
2015	Tumbes	Men	60+	Aware treated	126.7	116.1	137.2
2016	Tumbes	Men	<60	Healthy	119.5	118.0	121.1
2016	Tumbes	Men	<60	Unaware	150.6	145.9	155.4
2016	Tumbes	Men	<60	Aware not treated	136.8	129.1	144.5
2016	Tumbes	Men	<60	Aware treated	147.0	129.7	164.2
2016	Tumbes	Men	60+	Healthy	118.7	114.8	122.6
2016	Tumbes	Men	60+	Unaware	162.2	144.4	180.0
2016	Tumbes	Men	60+	Aware not treated	135.4	120.6	150.2
2016	Tumbes	Men	60+	Aware treated	140.8	133.1	148.4
2017	Tumbes	Men	<60	Healthy	119.2	117.7	120.6
2017	Tumbes	Men	<60	Unaware	150.6	144.6	156.6
2017	Tumbes	Men	<60	Aware not treated	134.5	125.0	143.9
2017	Tumbes	Men	<60	Aware treated	141.9	125.6	158.2
2017	Tumbes	Men	60+	Healthy	117.2	113.5	120.9
2017	Tumbes	Men	60+	Unaware	154.8	148.7	160.9
2017	Tumbes	Men	60+	Aware not treated	127.3	97.1	157.5
2017	Tumbes	Men	60+	Aware treated	137.4	127.4	147.3
2018	Tumbes	Men	<60	Healthy	117.3	116.0	118.6
2018	Tumbes	Men	<60	Unaware	146.6	142.1	151.0
2018	Tumbes	Men	<60	Aware not treated	134.6	130.3	138.8

2018	Tumbes	Men	<60	Aware treated	128.8	121.1	136.5
2018	Tumbes	Men	60+	Healthy	122.2	117.5	127.0
2018	Tumbes	Men	60+	Unaware	156.8	145.4	168.2
2018	Tumbes	Men	60+	Aware not treated	150.7	115.2	186.2
2018	Tumbes	Men	60+	Aware treated	142.9	131.6	154.3
2019	Tumbes	Men	<60	Healthy	119.9	118.5	121.4
2019	Tumbes	Men	<60	Unaware	147.0	142.8	151.1
2019	Tumbes	Men	<60	Aware not treated	134.9	130.2	139.7
2019	Tumbes	Men	<60	Aware treated	146.0	136.0	156.1
2019	Tumbes	Men	60+	Healthy	118.5	114.1	122.9
2019	Tumbes	Men	60+	Unaware	158.2	152.0	164.5
2019	Tumbes	Men	60+	Aware not treated	136.8	105.4	168.2
2019	Tumbes	Men	60+	Aware treated	145.7	133.8	157.6
2020	Tumbes	Men	<60	Healthy	119.8	118.1	121.6
2020	Tumbes	Men	<60	Unaware	148.3	142.4	154.3
2020	Tumbes	Men	<60	Aware not treated	143.3	120.1	166.6
2020	Tumbes	Men	<60	Aware treated	134.1	125.0	143.3
2020	Tumbes	Men	60+	Healthy	121.8	118.2	125.5
2020	Tumbes	Men	60+	Unaware	159.2	148.6	169.7
2020	Tumbes	Men	60+	Aware not treated	136.7	126.0	147.3
2020	Tumbes	Men	60+	Aware treated	151.3	132.4	170.2
2015	Tumbes	Women	<60	Healthy	109.4	107.7	111.1
2015	Tumbes	Women	<60	Unaware	145.4	140.1	150.7
2015	Tumbes	Women	<60	Aware not treated	122.6	111.1	134.2
2015	Tumbes	Women	<60	Aware treated	125.5	118.9	132.1
2015	Tumbes	Women	60+	Healthy	117.2	112.0	122.5
2015	Tumbes	Women	60+	Unaware	163.9	147.1	180.6
2015	Tumbes	Women	60+	Aware not treated	135.0	135.0	135.0
2015	Tumbes	Women	60+	Aware treated	140.2	129.6	150.8

2016	Tumbes	Women	<60	Healthy	108.5	106.9	110.1
2016	Tumbes	Women	<60	Unaware	153.5	145.3	161.7
2016	Tumbes	Women	<60	Aware not treated	122.9	113.5	132.3
2016	Tumbes	Women	<60	Aware treated	130.9	122.5	139.3
2016	Tumbes	Women	60+	Healthy	122.1	118.3	125.8
2016	Tumbes	Women	60+	Unaware	152.9	143.6	162.1
2016	Tumbes	Women	60+	Aware not treated	144.6	108.5	180.8
2016	Tumbes	Women	60+	Aware treated	148.6	132.4	164.7
2017	Tumbes	Women	<60	Healthy	109.4	108.3	110.5
2017	Tumbes	Women	<60	Unaware	154.4	140.8	168.1
2017	Tumbes	Women	<60	Aware not treated	115.0	106.5	123.6
2017	Tumbes	Women	<60	Aware treated	129.4	122.4	136.4
2017	Tumbes	Women	60+	Healthy	119.0	112.4	125.7
2017	Tumbes	Women	60+	Unaware	143.8	137.3	150.4
2017	Tumbes	Women	60+	Aware not treated	144.2	137.9	150.5
2017	Tumbes	Women	60+	Aware treated	141.3	131.4	151.3
2018	Tumbes	Women	<60	Healthy	109.6	108.0	111.2
2018	Tumbes	Women	<60	Unaware	142.0	137.1	146.8
2018	Tumbes	Women	<60	Aware not treated	113.2	107.7	118.6
2018	Tumbes	Women	<60	Aware treated	127.6	119.6	135.5
2018	Tumbes	Women	60+	Healthy	113.4	107.6	119.2
2018	Tumbes	Women	60+	Unaware	149.9	145.1	154.8
2018	Tumbes	Women	60+	Aware not treated	126.6	113.4	139.7
2018	Tumbes	Women	60+	Aware treated	140.1	132.0	148.3
2019	Tumbes	Women	<60	Healthy	108.1	106.6	109.6
2019	Tumbes	Women	<60	Unaware	141.0	134.8	147.2
2019	Tumbes	Women	<60	Aware not treated	121.1	111.3	130.9
2019	Tumbes	Women	<60	Aware treated	122.8	116.0	129.5
2019	Tumbes	Women	60+	Healthy	118.9	115.3	122.5

2019	Tumbes	Women	60+	Unaware	152.6	146.9	158.3
2019	Tumbes	Women	60+	Aware not treated	139.6	113.4	165.8
2019	Tumbes	Women	60+	Aware treated	137.5	130.8	144.2
2020	Tumbes	Women	<60	Healthy	114.0	112.6	115.5
2020	Tumbes	Women	<60	Unaware	160.0	148.5	171.4
2020	Tumbes	Women	<60	Aware not treated	131.7	113.8	149.6
2020	Tumbes	Women	<60	Aware treated	136.1	124.3	147.8
2020	Tumbes	Women	60+	Healthy	117.2	113.6	120.7
2020	Tumbes	Women	60+	Unaware	153.9	141.6	166.1
2020	Tumbes	Women	60+	Aware not treated	132.7	114.7	150.7
2020	Tumbes	Women	60+	Aware treated	142.6	130.1	155.1
2015	Ucayali	Men	<60	Healthy	117.9	116.5	119.2
2015	Ucayali	Men	<60	Unaware	152.9	145.8	160.0
2015	Ucayali	Men	<60	Aware not treated	131.3	125.1	137.5
2015	Ucayali	Men	<60	Aware treated	139.2	123.9	154.6
2015	Ucayali	Men	60+	Healthy	119.1	115.0	123.2
2015	Ucayali	Men	60+	Unaware	157.5	145.3	169.7
2015	Ucayali	Men	60+	Aware not treated	125.6	109.2	141.9
2015	Ucayali	Men	60+	Aware treated	132.1	120.3	143.8
2016	Ucayali	Men	<60	Healthy	117.5	116.0	118.9
2016	Ucayali	Men	<60	Unaware	154.0	144.2	163.7
2016	Ucayali	Men	<60	Aware not treated	129.5	126.2	132.8
2016	Ucayali	Men	<60	Aware treated	138.8	126.6	151.0
2016	Ucayali	Men	60+	Healthy	117.2	113.3	121.0
2016	Ucayali	Men	60+	Unaware	154.2	146.1	162.3
2016	Ucayali	Men	60+	Aware not treated	83.0	83.0	83.0
2016	Ucayali	Men	60+	Aware treated	141.7	128.1	155.3
2017	Ucayali	Men	<60	Healthy	118.6	116.8	120.4
2017	Ucayali	Men	<60	Unaware	146.7	142.1	151.3

2017	Ucayali	Men	<60	Aware treated	123.9	109.0	138.8
2017	Ucayali	Men	60+	Healthy	119.5	115.0	124.1
2017	Ucayali	Men	60+	Unaware	151.5	145.6	157.5
2017	Ucayali	Men	60+	Aware not treated	144.8	136.9	152.7
2017	Ucayali	Men	60+	Aware treated	147.8	125.8	169.8
2018	Ucayali	Men	<60	Healthy	118.2	116.7	119.6
2018	Ucayali	Men	<60	Unaware	146.7	140.2	153.2
2018	Ucayali	Men	<60	Aware not treated	124.0	114.7	133.2
2018	Ucayali	Men	<60	Aware treated	145.3	135.8	154.9
2018	Ucayali	Men	60+	Healthy	118.3	113.8	122.7
2018	Ucayali	Men	60+	Unaware	145.8	140.2	151.4
2018	Ucayali	Men	60+	Aware not treated	153.9	147.3	160.5
2018	Ucayali	Men	60+	Aware treated	144.1	130.8	157.4
2019	Ucayali	Men	<60	Healthy	119.5	118.2	120.9
2019	Ucayali	Men	<60	Unaware	143.4	136.9	150.0
2019	Ucayali	Men	<60	Aware not treated	126.2	122.4	130.0
2019	Ucayali	Men	<60	Aware treated	130.4	119.7	141.1
2019	Ucayali	Men	60+	Healthy	118.2	114.6	121.8
2019	Ucayali	Men	60+	Unaware	152.5	143.3	161.8
2019	Ucayali	Men	60+	Aware not treated	133.0	133.0	133.0
2019	Ucayali	Men	60+	Aware treated	145.0	128.9	161.1
2020	Ucayali	Men	<60	Healthy	119.3	117.8	120.8
2020	Ucayali	Men	<60	Unaware	147.6	143.4	151.9
2020	Ucayali	Men	<60	Aware not treated	130.5	114.9	146.0
2020	Ucayali	Men	<60	Aware treated	148.9	124.6	173.1
2020	Ucayali	Men	60+	Healthy	121.5	117.7	125.2
2020	Ucayali	Men	60+	Unaware	149.5	141.4	157.5
2020	Ucayali	Men	60+	Aware not treated	138.6	123.2	154.0
2020	Ucayali	Men	60+	Aware treated	148.6	137.7	159.6

2015	Ucayali	Women	<60	Healthy	108.7	107.3	110.1
2015	Ucayali	Women	<60	Unaware	152.9	145.0	160.8
2015	Ucayali	Women	<60	Aware not treated	108.6	100.2	117.1
2015	Ucayali	Women	<60	Aware treated	118.8	112.4	125.2
2015	Ucayali	Women	60+	Healthy	117.1	113.0	121.2
2015	Ucayali	Women	60+	Unaware	148.6	136.5	160.7
2015	Ucayali	Women	60+	Aware not treated	134.6	96.7	172.4
2015	Ucayali	Women	60+	Aware treated	129.6	118.0	141.1
2016	Ucayali	Women	<60	Healthy	108.2	106.8	109.5
2016	Ucayali	Women	<60	Unaware	151.3	149.5	153.1
2016	Ucayali	Women	<60	Aware not treated	128.8	119.0	138.6
2016	Ucayali	Women	<60	Aware treated	126.1	119.0	133.2
2016	Ucayali	Women	60+	Healthy	113.2	108.5	117.9
2016	Ucayali	Women	60+	Unaware	165.6	149.2	181.9
2016	Ucayali	Women	60+	Aware not treated	133.0	133.0	133.0
2016	Ucayali	Women	60+	Aware treated	135.0	123.5	146.5
2017	Ucayali	Women	<60	Healthy	110.1	108.7	111.5
2017	Ucayali	Women	<60	Unaware	151.3	138.0	164.6
2017	Ucayali	Women	<60	Aware not treated	132.5	121.4	143.5
2017	Ucayali	Women	<60	Aware treated	129.6	120.2	139.0
2017	Ucayali	Women	60+	Healthy	114.8	111.4	118.2
2017	Ucayali	Women	60+	Unaware	147.6	139.9	155.2
2017	Ucayali	Women	60+	Aware not treated	127.5	120.2	134.8
2017	Ucayali	Women	60+	Aware treated	141.4	132.6	150.2
2018	Ucayali	Women	<60	Healthy	108.9	107.4	110.3
2018	Ucayali	Women	<60	Unaware	151.0	145.5	156.5
2018	Ucayali	Women	<60	Aware not treated	106.7	94.0	119.5
2018	Ucayali	Women	<60	Aware treated	126.3	116.7	135.8
2018	Ucayali	Women	60+	Healthy	117.8	113.9	121.7

2018	Ucayali	Women	60+	Unaware	151.3	143.6	159.0
2018	Ucayali	Women	60+	Aware not treated	146.4	130.9	161.8
2018	Ucayali	Women	60+	Aware treated	135.2	126.4	144.1
2019	Ucayali	Women	<60	Healthy	109.6	108.3	110.9
2019	Ucayali	Women	<60	Unaware	144.3	138.6	150.1
2019	Ucayali	Women	<60	Aware not treated	129.1	106.2	152.0
2019	Ucayali	Women	<60	Aware treated	127.5	119.9	135.2
2019	Ucayali	Women	60+	Healthy	117.6	114.9	120.4
2019	Ucayali	Women	60+	Unaware	149.2	139.4	158.9
2019	Ucayali	Women	60+	Aware not treated	130.1	93.8	166.5
2019	Ucayali	Women	60+	Aware treated	142.9	129.8	156.0
2020	Ucayali	Women	<60	Healthy	111.3	109.7	113.0
2020	Ucayali	Women	<60	Unaware	147.4	141.9	152.9
2020	Ucayali	Women	<60	Aware not treated	115.8	107.0	124.6
2020	Ucayali	Women	<60	Aware treated	125.1	112.5	137.8
2020	Ucayali	Women	60+	Healthy	120.2	115.6	124.8
2020	Ucayali	Women	60+	Unaware	149.8	142.4	157.2
2020	Ucayali	Women	60+	Aware treated	136.0	123.4	148.5
2015	Apurimac	Men	<60	Healthy	118.6	117.1	120.2
2015	Apurimac	Men	<60	Unaware	143.9	139.7	148.2
2015	Apurimac	Men	<60	Aware not treated	134.9	120.6	149.2
2015	Apurimac	Men	<60	Aware treated	125.0	116.1	134.0
2015	Apurimac	Men	60+	Healthy	117.8	114.3	121.3
2015	Apurimac	Men	60+	Unaware	150.4	146.0	154.9
2015	Apurimac	Men	60+	Aware not treated	119.8	102.5	137.2
2015	Apurimac	Men	60+	Aware treated	134.5	112.9	156.1
2016	Apurimac	Men	<60	Healthy	118.3	116.8	119.8
2016	Apurimac	Men	<60	Unaware	143.4	141.2	145.6
2016	Apurimac	Men	<60	Aware not treated	117.0	108.2	125.9

2016	Apurimac	Men	<60	Aware treated	130.1	114.3	145.9
2016	Apurimac	Men	60+	Healthy	120.7	117.0	124.3
2016	Apurimac	Men	60+	Unaware	156.1	146.2	165.9
2016	Apurimac	Men	60+	Aware not treated	136.2	120.7	151.7
2016	Apurimac	Men	60+	Aware treated	140.2	125.8	154.5
2017	Apurimac	Men	<60	Healthy	119.7	118.1	121.3
2017	Apurimac	Men	<60	Unaware	145.0	141.2	148.9
2017	Apurimac	Men	<60	Aware not treated	132.4	120.1	144.6
2017	Apurimac	Men	<60	Aware treated	127.7	126.4	129.0
2017	Apurimac	Men	60+	Healthy	120.4	114.8	126.1
2017	Apurimac	Men	60+	Unaware	155.8	149.2	162.3
2017	Apurimac	Men	60+	Aware not treated	126.9	105.5	148.2
2017	Apurimac	Men	60+	Aware treated	146.2	121.8	170.6
2018	Apurimac	Men	<60	Healthy	118.2	117.0	119.5
2018	Apurimac	Men	<60	Unaware	141.9	137.2	146.6
2018	Apurimac	Men	<60	Aware not treated	143.0	128.1	157.9
2018	Apurimac	Men	<60	Aware treated	129.5	111.2	147.8
2018	Apurimac	Men	60+	Healthy	119.9	115.2	124.6
2018	Apurimac	Men	60+	Unaware	148.0	142.9	153.1
2018	Apurimac	Men	60+	Aware not treated	148.1	136.2	159.9
2018	Apurimac	Men	60+	Aware treated	143.7	127.6	159.8
2019	Apurimac	Men	<60	Healthy	118.1	116.8	119.3
2019	Apurimac	Men	<60	Unaware	146.9	143.2	150.5
2019	Apurimac	Men	<60	Aware not treated	133.6	117.3	150.0
2019	Apurimac	Men	<60	Aware treated	151.3	106.0	196.6
2019	Apurimac	Men	60+	Healthy	118.0	114.1	121.9
2019	Apurimac	Men	60+	Unaware	150.2	145.7	154.6
2019	Apurimac	Men	60+	Aware not treated	141.6	123.4	159.7
2019	Apurimac	Men	60+	Aware treated	140.8	127.3	154.3

2020	Apurimac	Men	<60	Healthy	117.8	115.9	119.8
2020	Apurimac	Men	<60	Unaware	142.4	138.2	146.7
2020	Apurimac	Men	<60	Aware not treated	116.2	109.8	122.6
2020	Apurimac	Men	<60	Aware treated	136.9	116.9	156.9
2020	Apurimac	Men	60+	Healthy	119.5	112.7	126.3
2020	Apurimac	Men	60+	Unaware	163.3	149.1	177.4
2020	Apurimac	Men	60+	Aware not treated	126.3	124.2	128.3
2020	Apurimac	Men	60+	Aware treated	146.1	130.6	161.6
2015	Apurimac	Women	<60	Healthy	111.9	110.1	113.8
2015	Apurimac	Women	<60	Unaware	146.1	139.9	152.2
2015	Apurimac	Women	<60	Aware not treated	119.3	112.3	126.3
2015	Apurimac	Women	<60	Aware treated	137.7	125.3	150.1
2015	Apurimac	Women	60+	Healthy	118.3	114.8	121.8
2015	Apurimac	Women	60+	Unaware	151.5	144.1	159.0
2015	Apurimac	Women	60+	Aware not treated	128.3	112.5	144.1
2015	Apurimac	Women	60+	Aware treated	149.0	136.8	161.3
2016	Apurimac	Women	<60	Healthy	112.3	110.8	113.9
2016	Apurimac	Women	<60	Unaware	149.3	144.8	153.8
2016	Apurimac	Women	<60	Aware not treated	113.5	107.1	119.8
2016	Apurimac	Women	<60	Aware treated	118.5	112.6	124.3
2016	Apurimac	Women	60+	Healthy	115.8	111.9	119.8
2016	Apurimac	Women	60+	Unaware	147.9	144.9	151.0
2016	Apurimac	Women	60+	Aware not treated	129.6	114.2	144.9
2016	Apurimac	Women	60+	Aware treated	142.3	130.2	154.4
2017	Apurimac	Women	<60	Healthy	112.5	111.2	113.8
2017	Apurimac	Women	<60	Unaware	141.5	138.0	145.1
2017	Apurimac	Women	<60	Aware not treated	131.6	121.0	142.2
2017	Apurimac	Women	<60	Aware treated	122.5	109.8	135.3
2017	Apurimac	Women	60+	Healthy	118.5	115.5	121.5

2017	Apurimac	Women	60+	Unaware	154.1	150.0	158.3
2017	Apurimac	Women	60+	Aware not treated	135.8	126.1	145.4
2017	Apurimac	Women	60+	Aware treated	148.4	133.7	163.1
2018	Apurimac	Women	<60	Healthy	111.9	110.3	113.6
2018	Apurimac	Women	<60	Unaware	140.7	135.0	146.4
2018	Apurimac	Women	<60	Aware not treated	114.6	108.1	121.1
2018	Apurimac	Women	<60	Aware treated	121.9	102.0	141.9
2018	Apurimac	Women	60+	Healthy	115.4	111.7	119.1
2018	Apurimac	Women	60+	Unaware	162.3	148.7	175.8
2018	Apurimac	Women	60+	Aware not treated	142.4	95.2	189.5
2018	Apurimac	Women	60+	Aware treated	147.9	130.8	165.0
2019	Apurimac	Women	<60	Healthy	111.0	109.4	112.6
2019	Apurimac	Women	<60	Unaware	142.6	135.8	149.5
2019	Apurimac	Women	<60	Aware not treated	126.6	109.0	144.2
2019	Apurimac	Women	<60	Aware treated	128.0	114.6	141.4
2019	Apurimac	Women	60+	Healthy	115.0	110.9	119.0
2019	Apurimac	Women	60+	Unaware	153.4	146.0	160.9
2019	Apurimac	Women	60+	Aware not treated	134.0	117.3	150.6
2019	Apurimac	Women	60+	Aware treated	142.0	135.1	148.9
2020	Apurimac	Women	<60	Healthy	109.8	108.2	111.5
2020	Apurimac	Women	<60	Unaware	146.8	144.3	149.3
2020	Apurimac	Women	<60	Aware not treated	121.4	114.6	128.2
2020	Apurimac	Women	<60	Aware treated	115.6	108.3	122.8
2020	Apurimac	Women	60+	Healthy	113.0	109.1	116.8
2020	Apurimac	Women	60+	Unaware	149.8	144.8	154.8
2020	Apurimac	Women	60+	Aware not treated	133.4	121.5	145.2
2020	Apurimac	Women	60+	Aware treated	157.8	133.8	181.7
2015	Arequipa	Men	<60	Healthy	120.4	118.8	122.0
2015	Arequipa	Men	<60	Unaware	144.9	141.4	148.4

2015	Arequipa	Men	<60	Aware not treated	145.9	134.0	157.8
2015	Arequipa	Men	<60	Aware treated	133.5	121.2	145.7
2015	Arequipa	Men	60+	Healthy	121.0	116.2	125.8
2015	Arequipa	Men	60+	Unaware	155.1	147.2	163.1
2015	Arequipa	Men	60+	Aware not treated	128.0	128.0	128.0
2015	Arequipa	Men	60+	Aware treated	146.2	135.0	157.5
2016	Arequipa	Men	<60	Healthy	120.0	118.4	121.6
2016	Arequipa	Men	<60	Unaware	146.2	141.2	151.2
2016	Arequipa	Men	<60	Aware not treated	144.7	121.3	168.2
2016	Arequipa	Men	<60	Aware treated	118.4	105.2	131.5
2016	Arequipa	Men	60+	Healthy	122.0	118.9	125.0
2016	Arequipa	Men	60+	Unaware	163.2	149.0	177.5
2016	Arequipa	Men	60+	Aware not treated	159.0	159.0	159.0
2016	Arequipa	Men	60+	Aware treated	136.1	124.8	147.3
2017	Arequipa	Men	<60	Healthy	120.2	118.8	121.7
2017	Arequipa	Men	<60	Unaware	141.6	138.7	144.6
2017	Arequipa	Men	<60	Aware not treated	124.3	119.2	129.4
2017	Arequipa	Men	<60	Aware treated	121.5	112.2	130.8
2017	Arequipa	Men	60+	Healthy	121.9	119.1	124.7
2017	Arequipa	Men	60+	Unaware	157.6	150.0	165.1
2017	Arequipa	Men	60+	Aware not treated	142.4	126.6	158.2
2017	Arequipa	Men	60+	Aware treated	147.4	140.1	154.8
2018	Arequipa	Men	<60	Healthy	119.6	118.1	121.1
2018	Arequipa	Men	<60	Unaware	140.8	137.3	144.2
2018	Arequipa	Men	<60	Aware not treated	130.6	122.9	138.4
2018	Arequipa	Men	<60	Aware treated	145.3	101.3	189.3
2018	Arequipa	Men	60+	Healthy	121.5	118.3	124.6
2018	Arequipa	Men	60+	Unaware	151.4	145.8	157.0
2018	Arequipa	Men	60+	Aware not treated	136.3	122.1	150.5

2018	Arequipa	Men	60+	Aware treated	142.1	132.7	151.5
2019	Arequipa	Men	<60	Healthy	123.4	122.1	124.7
2019	Arequipa	Men	<60	Unaware	145.4	142.1	148.7
2019	Arequipa	Men	<60	Aware not treated	136.9	124.6	149.1
2019	Arequipa	Men	<60	Aware treated	133.1	122.4	143.8
2019	Arequipa	Men	60+	Healthy	121.9	118.5	125.3
2019	Arequipa	Men	60+	Unaware	153.3	146.9	159.8
2019	Arequipa	Men	60+	Aware not treated	148.4	124.4	172.3
2019	Arequipa	Men	60+	Aware treated	145.9	138.5	153.3
2020	Arequipa	Men	<60	Healthy	122.1	120.2	124.0
2020	Arequipa	Men	<60	Unaware	145.5	137.0	153.9
2020	Arequipa	Men	<60	Aware not treated	116.0	116.0	116.0
2020	Arequipa	Men	<60	Aware treated	131.8	120.7	142.8
2020	Arequipa	Men	60+	Healthy	125.8	122.0	129.6
2020	Arequipa	Men	60+	Unaware	152.5	147.5	157.5
2020	Arequipa	Men	60+	Aware not treated	142.6	127.3	157.9
2020	Arequipa	Men	60+	Aware treated	136.3	124.5	148.2
2015	Arequipa	Women	<60	Healthy	111.5	110.0	113.0
2015	Arequipa	Women	<60	Unaware	141.0	136.9	145.0
2015	Arequipa	Women	<60	Aware not treated	117.9	111.4	124.3
2015	Arequipa	Women	<60	Aware treated	127.4	122.1	132.6
2015	Arequipa	Women	60+	Healthy	118.5	115.3	121.7
2015	Arequipa	Women	60+	Unaware	156.7	145.3	168.1
2015	Arequipa	Women	60+	Aware not treated	121.2	106.7	135.8
2015	Arequipa	Women	60+	Aware treated	135.4	126.2	144.6
2016	Arequipa	Women	<60	Healthy	111.5	110.0	113.0
2016	Arequipa	Women	<60	Unaware	143.8	140.2	147.4
2016	Arequipa	Women	<60	Aware not treated	116.4	107.6	125.2
2016	Arequipa	Women	<60	Aware treated	134.9	113.9	156.0

2016	Arequipa	Women	60+	Healthy	117.2	112.8	121.6
2016	Arequipa	Women	60+	Unaware	154.0	143.2	164.8
2016	Arequipa	Women	60+	Aware not treated	129.6	112.4	146.8
2016	Arequipa	Women	60+	Aware treated	135.7	119.7	151.8
2017	Arequipa	Women	<60	Healthy	110.4	109.0	111.8
2017	Arequipa	Women	<60	Unaware	137.4	129.8	144.9
2017	Arequipa	Women	<60	Aware not treated	114.7	109.0	120.3
2017	Arequipa	Women	<60	Aware treated	135.7	122.0	149.4
2017	Arequipa	Women	60+	Healthy	119.5	115.7	123.2
2017	Arequipa	Women	60+	Unaware	150.3	142.3	158.2
2017	Arequipa	Women	60+	Aware not treated	142.7	131.8	153.5
2017	Arequipa	Women	60+	Aware treated	141.5	129.1	153.9
2018	Arequipa	Women	<60	Healthy	111.0	109.6	112.4
2018	Arequipa	Women	<60	Unaware	142.4	136.7	148.1
2018	Arequipa	Women	<60	Aware not treated	120.0	112.4	127.6
2018	Arequipa	Women	<60	Aware treated	130.1	117.1	143.1
2018	Arequipa	Women	60+	Healthy	118.8	115.3	122.3
2018	Arequipa	Women	60+	Unaware	152.0	146.7	157.3
2018	Arequipa	Women	60+	Aware not treated	113.8	96.1	131.4
2018	Arequipa	Women	60+	Aware treated	137.2	129.8	144.6
2019	Arequipa	Women	<60	Healthy	112.0	110.7	113.4
2019	Arequipa	Women	<60	Unaware	146.7	141.7	151.8
2019	Arequipa	Women	<60	Aware not treated	116.3	109.3	123.2
2019	Arequipa	Women	<60	Aware treated	133.9	127.5	140.4
2019	Arequipa	Women	60+	Healthy	118.5	114.3	122.8
2019	Arequipa	Women	60+	Unaware	152.7	146.8	158.5
2019	Arequipa	Women	60+	Aware not treated	136.1	124.8	147.4
2019	Arequipa	Women	60+	Aware treated	147.5	140.0	154.9
2020	Arequipa	Women	<60	Healthy	112.6	111.1	114.1

2020	Arequipa	Women	<60	Unaware	143.4	138.6	148.2
2020	Arequipa	Women	<60	Aware not treated	132.3	117.8	146.9
2020	Arequipa	Women	<60	Aware treated	120.2	109.6	130.7
2020	Arequipa	Women	60+	Healthy	120.0	114.1	125.9
2020	Arequipa	Women	60+	Unaware	155.4	150.3	160.4
2020	Arequipa	Women	60+	Aware not treated	147.1	129.9	164.4
2020	Arequipa	Women	60+	Aware treated	147.3	136.9	157.8
2015	Ayacucho	Men	<60	Healthy	117.2	115.9	118.6
2015	Ayacucho	Men	<60	Unaware	150.6	144.5	156.6
2015	Ayacucho	Men	<60	Aware not treated	128.0	123.4	132.6
2015	Ayacucho	Men	<60	Aware treated	133.8	124.2	143.4
2015	Ayacucho	Men	60+	Healthy	118.8	115.2	122.3
2015	Ayacucho	Men	60+	Unaware	162.8	152.2	173.5
2015	Ayacucho	Men	60+	Aware not treated	133.1	119.5	146.8
2015	Ayacucho	Men	60+	Aware treated	146.9	131.7	162.1
2016	Ayacucho	Men	<60	Healthy	117.6	116.3	118.9
2016	Ayacucho	Men	<60	Unaware	142.7	139.0	146.4
2016	Ayacucho	Men	<60	Aware not treated	135.3	122.0	148.7
2016	Ayacucho	Men	<60	Aware treated	141.1	131.6	150.6
2016	Ayacucho	Men	60+	Healthy	119.0	116.1	122.0
2016	Ayacucho	Men	60+	Unaware	150.5	147.1	154.0
2016	Ayacucho	Men	60+	Aware not treated	143.0	131.5	154.5
2016	Ayacucho	Men	60+	Aware treated	164.6	153.1	176.1
2017	Ayacucho	Men	<60	Healthy	117.8	116.4	119.2
2017	Ayacucho	Men	<60	Unaware	145.2	142.1	148.3
2017	Ayacucho	Men	<60	Aware not treated	129.6	116.6	142.6
2017	Ayacucho	Men	<60	Aware treated	135.8	123.6	148.1
2017	Ayacucho	Men	60+	Healthy	119.2	116.2	122.1
2017	Ayacucho	Men	60+	Unaware	154.2	149.8	158.6

2017	Ayacucho	Men	60+	Aware not treated	143.8	134.3	153.4
2017	Ayacucho	Men	60+	Aware treated	145.2	135.9	154.6
2018	Ayacucho	Men	<60	Healthy	119.0	117.7	120.4
2018	Ayacucho	Men	<60	Unaware	145.2	142.4	147.9
2018	Ayacucho	Men	<60	Aware not treated	122.5	113.1	131.9
2018	Ayacucho	Men	<60	Aware treated	141.2	115.0	167.3
2018	Ayacucho	Men	60+	Healthy	121.0	117.7	124.4
2018	Ayacucho	Men	60+	Unaware	158.6	148.8	168.4
2018	Ayacucho	Men	60+	Aware not treated	133.7	124.9	142.6
2018	Ayacucho	Men	60+	Aware treated	151.9	134.6	169.3
2019	Ayacucho	Men	<60	Healthy	119.2	117.7	120.7
2019	Ayacucho	Men	<60	Unaware	143.7	140.2	147.1
2019	Ayacucho	Men	<60	Aware not treated	124.0	118.4	129.5
2019	Ayacucho	Men	<60	Aware treated	133.9	117.3	150.4
2019	Ayacucho	Men	60+	Healthy	119.8	116.0	123.6
2019	Ayacucho	Men	60+	Unaware	152.6	148.7	156.5
2019	Ayacucho	Men	60+	Aware not treated	132.0	125.4	138.6
2019	Ayacucho	Men	60+	Aware treated	150.8	140.6	161.0
2020	Ayacucho	Men	<60	Healthy	117.7	116.1	119.4
2020	Ayacucho	Men	<60	Unaware	146.6	139.7	153.5
2020	Ayacucho	Men	<60	Aware not treated	126.6	117.9	135.2
2020	Ayacucho	Men	<60	Aware treated	122.4	117.9	127.0
2020	Ayacucho	Men	60+	Healthy	123.4	119.1	127.7
2020	Ayacucho	Men	60+	Unaware	152.5	145.8	159.2
2020	Ayacucho	Men	60+	Aware not treated	121.8	114.0	129.5
2020	Ayacucho	Men	60+	Aware treated	151.7	131.9	171.4
2015	Ayacucho	Women	<60	Healthy	110.5	109.0	112.0
2015	Ayacucho	Women	<60	Unaware	159.5	151.0	167.9
2015	Ayacucho	Women	<60	Aware not treated	111.5	106.9	116.1

2015	Ayacucho	Women	<60	Aware treated	113.8	108.1	119.5
2015	Ayacucho	Women	60+	Healthy	114.6	111.7	117.5
2015	Ayacucho	Women	60+	Unaware	153.0	147.2	158.7
2015	Ayacucho	Women	60+	Aware not treated	152.5	129.1	175.9
2015	Ayacucho	Women	60+	Aware treated	151.3	133.1	169.6
2016	Ayacucho	Women	<60	Healthy	110.0	108.5	111.4
2016	Ayacucho	Women	<60	Unaware	145.3	138.0	152.5
2016	Ayacucho	Women	<60	Aware not treated	114.2	107.5	121.0
2016	Ayacucho	Women	<60	Aware treated	124.4	115.0	133.8
2016	Ayacucho	Women	60+	Healthy	118.0	114.7	121.3
2016	Ayacucho	Women	60+	Unaware	160.8	155.3	166.3
2016	Ayacucho	Women	60+	Aware not treated	147.8	132.1	163.5
2016	Ayacucho	Women	60+	Aware treated	142.1	134.1	150.0
2017	Ayacucho	Women	<60	Healthy	109.6	108.2	110.9
2017	Ayacucho	Women	<60	Unaware	148.8	143.7	153.8
2017	Ayacucho	Women	<60	Aware not treated	123.1	115.5	130.7
2017	Ayacucho	Women	<60	Aware treated	123.4	111.9	134.8
2017	Ayacucho	Women	60+	Healthy	115.2	111.7	118.7
2017	Ayacucho	Women	60+	Unaware	158.6	149.8	167.5
2017	Ayacucho	Women	60+	Aware not treated	140.1	123.2	157.0
2017	Ayacucho	Women	60+	Aware treated	147.7	134.4	161.0
2018	Ayacucho	Women	<60	Healthy	110.2	108.9	111.5
2018	Ayacucho	Women	<60	Unaware	142.0	136.9	147.1
2018	Ayacucho	Women	<60	Aware not treated	118.6	105.4	131.8
2018	Ayacucho	Women	<60	Aware treated	124.6	108.4	140.9
2018	Ayacucho	Women	60+	Healthy	116.4	113.6	119.1
2018	Ayacucho	Women	60+	Unaware	154.7	147.5	161.8
2018	Ayacucho	Women	60+	Aware not treated	138.0	114.0	162.0
2018	Ayacucho	Women	60+	Aware treated	144.1	136.6	151.5

2019	Ayacucho	Women	<60	Healthy	110.0	108.7	111.2
2019	Ayacucho	Women	<60	Unaware	135.8	130.8	140.8
2019	Ayacucho	Women	<60	Aware not treated	120.1	110.9	129.2
2019	Ayacucho	Women	<60	Aware treated	122.6	111.8	133.3
2019	Ayacucho	Women	60+	Healthy	118.2	115.3	121.2
2019	Ayacucho	Women	60+	Unaware	146.6	143.2	149.9
2019	Ayacucho	Women	60+	Aware not treated	127.0	109.9	144.1
2019	Ayacucho	Women	60+	Aware treated	137.1	127.1	147.1
2020	Ayacucho	Women	<60	Healthy	110.7	108.9	112.5
2020	Ayacucho	Women	<60	Unaware	137.4	131.8	143.0
2020	Ayacucho	Women	<60	Aware not treated	119.9	112.7	127.1
2020	Ayacucho	Women	<60	Aware treated	119.2	104.4	134.0
2020	Ayacucho	Women	60+	Healthy	114.9	111.1	118.6
2020	Ayacucho	Women	60+	Unaware	154.1	146.7	161.6
2020	Ayacucho	Women	60+	Aware not treated	131.4	114.6	148.3
2020	Ayacucho	Women	60+	Aware treated	145.5	136.3	154.7
2015	Cajamarca	Men	<60	Healthy	120.2	118.8	121.6
2015	Cajamarca	Men	<60	Unaware	146.7	139.0	154.5
2015	Cajamarca	Men	<60	Aware not treated	121.5	112.5	130.6
2015	Cajamarca	Men	<60	Aware treated	128.3	120.5	136.2
2015	Cajamarca	Men	60+	Healthy	117.3	112.5	122.1
2015	Cajamarca	Men	60+	Unaware	169.3	157.8	180.7
2015	Cajamarca	Men	60+	Aware not treated	140.3	123.1	157.6
2015	Cajamarca	Men	60+	Aware treated	132.5	120.2	144.8
2016	Cajamarca	Men	<60	Healthy	120.1	118.5	121.6
2016	Cajamarca	Men	<60	Unaware	144.1	141.5	146.8
2016	Cajamarca	Men	<60	Aware not treated	127.1	117.1	137.0
2016	Cajamarca	Men	<60	Aware treated	134.3	114.7	153.8
2016	Cajamarca	Men	60+	Healthy	118.6	115.4	121.8

2016	Cajamarca	Men	60+	Unaware	157.5	150.3	164.7
2016	Cajamarca	Men	60+	Aware not treated	159.9	135.0	184.7
2016	Cajamarca	Men	60+	Aware treated	146.6	134.4	158.8
2017	Cajamarca	Men	<60	Healthy	120.8	119.2	122.4
2017	Cajamarca	Men	<60	Unaware	146.3	142.6	150.1
2017	Cajamarca	Men	<60	Aware not treated	134.4	121.3	147.5
2017	Cajamarca	Men	<60	Aware treated	146.2	118.3	174.1
2017	Cajamarca	Men	60+	Healthy	122.8	117.1	128.5
2017	Cajamarca	Men	60+	Unaware	160.6	150.8	170.5
2017	Cajamarca	Men	60+	Aware not treated	164.8	123.4	206.2
2017	Cajamarca	Men	60+	Aware treated	160.9	151.4	170.3
2018	Cajamarca	Men	<60	Healthy	120.6	119.2	122.1
2018	Cajamarca	Men	<60	Unaware	144.5	138.8	150.2
2018	Cajamarca	Men	<60	Aware not treated	126.1	117.8	134.4
2018	Cajamarca	Men	<60	Aware treated	136.7	125.8	147.6
2018	Cajamarca	Men	60+	Healthy	120.1	117.0	123.2
2018	Cajamarca	Men	60+	Unaware	159.0	150.8	167.3
2018	Cajamarca	Men	60+	Aware not treated	145.5	123.6	167.3
2018	Cajamarca	Men	60+	Aware treated	154.2	141.7	166.7
2019	Cajamarca	Men	<60	Healthy	119.4	117.9	120.8
2019	Cajamarca	Men	<60	Unaware	145.3	140.6	150.1
2019	Cajamarca	Men	<60	Aware not treated	127.3	119.6	135.0
2019	Cajamarca	Men	<60	Aware treated	141.4	109.3	173.5
2019	Cajamarca	Men	60+	Healthy	120.6	117.7	123.4
2019	Cajamarca	Men	60+	Unaware	156.8	151.5	162.0
2019	Cajamarca	Men	60+	Aware not treated	136.7	119.9	153.4
2019	Cajamarca	Men	60+	Aware treated	154.1	132.4	175.8
2020	Cajamarca	Men	<60	Healthy	120.5	118.8	122.3
2020	Cajamarca	Men	<60	Unaware	148.0	143.1	153.0

2020	Cajamarca	Men	<60	Aware not treated	127.5	114.4	140.7
2020	Cajamarca	Men	<60	Aware treated	154.7	114.1	195.4
2020	Cajamarca	Men	60+	Healthy	120.7	116.4	125.0
2020	Cajamarca	Men	60+	Unaware	165.9	151.3	180.5
2020	Cajamarca	Men	60+	Aware not treated	139.9	137.3	142.6
2020	Cajamarca	Men	60+	Aware treated	163.5	141.4	185.6
2015	Cajamarca	Women	<60	Healthy	112.1	110.7	113.6
2015	Cajamarca	Women	<60	Unaware	149.6	142.3	156.9
2015	Cajamarca	Women	<60	Aware not treated	127.5	115.2	139.8
2015	Cajamarca	Women	<60	Aware treated	131.8	120.3	143.3
2015	Cajamarca	Women	60+	Healthy	118.3	114.5	122.2
2015	Cajamarca	Women	60+	Unaware	156.5	150.2	162.8
2015	Cajamarca	Women	60+	Aware not treated	138.3	116.5	160.0
2015	Cajamarca	Women	60+	Aware treated	165.0	146.3	183.7
2016	Cajamarca	Women	<60	Healthy	113.8	112.0	115.6
2016	Cajamarca	Women	<60	Unaware	150.7	141.6	159.9
2016	Cajamarca	Women	<60	Aware not treated	124.3	114.2	134.3
2016	Cajamarca	Women	<60	Aware treated	127.6	120.0	135.1
2016	Cajamarca	Women	60+	Healthy	117.1	113.1	121.2
2016	Cajamarca	Women	60+	Unaware	158.2	149.5	167.0
2016	Cajamarca	Women	60+	Aware not treated	123.0	111.5	134.4
2016	Cajamarca	Women	60+	Aware treated	147.0	131.6	162.5
2017	Cajamarca	Women	<60	Healthy	113.4	112.0	114.9
2017	Cajamarca	Women	<60	Unaware	160.9	149.9	171.9
2017	Cajamarca	Women	<60	Aware not treated	126.7	107.8	145.6
2017	Cajamarca	Women	<60	Aware treated	136.3	125.9	146.8
2017	Cajamarca	Women	60+	Healthy	119.7	116.1	123.2
2017	Cajamarca	Women	60+	Unaware	163.5	156.0	170.9
2017	Cajamarca	Women	60+	Aware not treated	157.2	146.8	167.6

2017	Cajamarca	Women	60+	Aware treated	145.9	134.8	156.9
2018	Cajamarca	Women	<60	Healthy	112.3	110.9	113.7
2018	Cajamarca	Women	<60	Unaware	141.1	134.7	147.5
2018	Cajamarca	Women	<60	Aware not treated	122.3	113.8	130.7
2018	Cajamarca	Women	<60	Aware treated	146.1	131.7	160.6
2018	Cajamarca	Women	60+	Healthy	118.9	115.7	122.2
2018	Cajamarca	Women	60+	Unaware	148.3	143.5	153.2
2018	Cajamarca	Women	60+	Aware not treated	131.9	119.2	144.6
2018	Cajamarca	Women	60+	Aware treated	158.3	150.2	166.5
2019	Cajamarca	Women	<60	Healthy	112.7	111.0	114.4
2019	Cajamarca	Women	<60	Unaware	148.3	139.6	157.0
2019	Cajamarca	Women	<60	Aware not treated	132.6	121.5	143.8
2019	Cajamarca	Women	<60	Aware treated	135.0	124.5	145.4
2019	Cajamarca	Women	60+	Healthy	119.1	116.2	121.9
2019	Cajamarca	Women	60+	Unaware	162.8	156.6	169.0
2019	Cajamarca	Women	60+	Aware not treated	135.1	113.1	157.0
2019	Cajamarca	Women	60+	Aware treated	140.3	125.2	155.5
2020	Cajamarca	Women	<60	Healthy	113.9	112.4	115.4
2020	Cajamarca	Women	<60	Unaware	150.7	144.3	157.1
2020	Cajamarca	Women	<60	Aware not treated	130.3	118.2	142.5
2020	Cajamarca	Women	<60	Aware treated	119.2	107.4	131.0
2020	Cajamarca	Women	60+	Healthy	118.1	113.5	122.6
2020	Cajamarca	Women	60+	Unaware	148.8	141.3	156.4
2020	Cajamarca	Women	60+	Aware not treated	154.0	132.7	175.3
2020	Cajamarca	Women	60+	Aware treated	152.7	130.0	175.5
2015	Callao	Men	<60	Healthy	120.2	118.7	121.7
2015	Callao	Men	<60	Unaware	146.0	142.9	149.1
2015	Callao	Men	<60	Aware not treated	130.0	116.7	143.3
2015	Callao	Men	<60	Aware treated	143.4	135.7	151.0

2015	Callao	Men	60+	Healthy	119.9	114.8	124.9
2015	Callao	Men	60+	Unaware	154.3	148.2	160.3
2015	Callao	Men	60+	Aware not treated	145.1	129.6	160.5
2015	Callao	Men	60+	Aware treated	145.4	133.8	157.0
2016	Callao	Men	<60	Healthy	121.0	119.7	122.3
2016	Callao	Men	<60	Unaware	157.3	147.8	166.9
2016	Callao	Men	<60	Aware not treated	135.1	131.7	138.5
2016	Callao	Men	<60	Aware treated	147.1	134.4	159.8
2016	Callao	Men	60+	Healthy	123.7	120.3	127.1
2016	Callao	Men	60+	Unaware	153.5	147.0	160.1
2016	Callao	Men	60+	Aware not treated	150.9	144.5	157.2
2016	Callao	Men	60+	Aware treated	145.6	136.2	155.0
2017	Callao	Men	<60	Healthy	121.1	119.8	122.4
2017	Callao	Men	<60	Unaware	150.7	146.1	155.3
2017	Callao	Men	<60	Aware not treated	145.5	135.5	155.4
2017	Callao	Men	<60	Aware treated	123.3	106.8	139.8
2017	Callao	Men	60+	Healthy	118.9	114.6	123.2
2017	Callao	Men	60+	Unaware	152.4	146.4	158.3
2017	Callao	Men	60+	Aware treated	154.9	139.1	170.6
2018	Callao	Men	<60	Healthy	121.7	120.5	122.9
2018	Callao	Men	<60	Unaware	146.4	143.5	149.3
2018	Callao	Men	<60	Aware not treated	137.0	123.1	150.9
2018	Callao	Men	<60	Aware treated	137.9	119.1	156.8
2018	Callao	Men	60+	Healthy	122.4	117.7	127.1
2018	Callao	Men	60+	Unaware	154.5	149.6	159.4
2018	Callao	Men	60+	Aware treated	155.8	143.5	168.1
2019	Callao	Men	<60	Healthy	119.8	118.4	121.2
2019	Callao	Men	<60	Unaware	148.3	143.9	152.7
2019	Callao	Men	<60	Aware not treated	120.7	110.4	130.9

2019	Callao	Men	<60	Aware treated	144.5	129.8	159.3
2019	Callao	Men	60+	Healthy	122.5	118.5	126.5
2019	Callao	Men	60+	Unaware	150.5	144.5	156.5
2019	Callao	Men	60+	Aware not treated	170.3	147.5	193.0
2019	Callao	Men	60+	Aware treated	149.6	139.8	159.5
2020	Callao	Men	<60	Healthy	121.1	119.1	123.1
2020	Callao	Men	<60	Unaware	146.6	143.4	149.9
2020	Callao	Men	<60	Aware not treated	132.9	124.9	141.0
2020	Callao	Men	<60	Aware treated	145.5	132.3	158.7
2020	Callao	Men	60+	Healthy	126.0	120.8	131.2
2020	Callao	Men	60+	Unaware	161.5	154.5	168.5
2020	Callao	Men	60+	Aware not treated	142.2	110.6	173.9
2020	Callao	Men	60+	Aware treated	151.2	136.1	166.3
2015	Callao	Women	<60	Healthy	111.8	110.4	113.3
2015	Callao	Women	<60	Unaware	152.4	147.3	157.5
2015	Callao	Women	<60	Aware not treated	123.7	108.8	138.6
2015	Callao	Women	<60	Aware treated	136.5	128.0	145.0
2015	Callao	Women	60+	Healthy	117.5	113.3	121.7
2015	Callao	Women	60+	Unaware	163.9	151.0	176.8
2015	Callao	Women	60+	Aware not treated	184.7	156.1	213.3
2015	Callao	Women	60+	Aware treated	146.0	137.9	154.1
2016	Callao	Women	<60	Healthy	111.3	109.8	112.8
2016	Callao	Women	<60	Unaware	150.6	141.6	159.5
2016	Callao	Women	<60	Aware not treated	137.2	104.9	169.6
2016	Callao	Women	<60	Aware treated	132.1	123.0	141.2
2016	Callao	Women	60+	Healthy	116.3	111.1	121.4
2016	Callao	Women	60+	Unaware	150.8	147.5	154.0
2016	Callao	Women	60+	Aware not treated	122.0	122.0	122.0
2016	Callao	Women	60+	Aware treated	142.8	135.7	149.8

2017	Callao	Women	<60	Healthy	113.4	111.8	114.9
2017	Callao	Women	<60	Unaware	147.4	141.4	153.4
2017	Callao	Women	<60	Aware not treated	118.5	108.0	129.1
2017	Callao	Women	<60	Aware treated	129.6	120.0	139.2
2017	Callao	Women	60+	Healthy	119.6	115.5	123.8
2017	Callao	Women	60+	Unaware	153.9	147.7	160.2
2017	Callao	Women	60+	Aware not treated	139.4	120.8	158.0
2017	Callao	Women	60+	Aware treated	151.2	142.8	159.6
2018	Callao	Women	<60	Healthy	113.0	111.6	114.4
2018	Callao	Women	<60	Unaware	151.8	144.3	159.3
2018	Callao	Women	<60	Aware not treated	118.1	109.9	126.3
2018	Callao	Women	<60	Aware treated	131.1	118.9	143.3
2018	Callao	Women	60+	Healthy	122.6	118.0	127.1
2018	Callao	Women	60+	Unaware	157.9	151.7	164.0
2018	Callao	Women	60+	Aware not treated	153.4	99.8	206.9
2018	Callao	Women	60+	Aware treated	145.4	136.2	154.5
2019	Callao	Women	<60	Healthy	113.7	112.2	115.2
2019	Callao	Women	<60	Unaware	151.4	145.3	157.5
2019	Callao	Women	<60	Aware not treated	113.5	108.7	118.2
2019	Callao	Women	<60	Aware treated	125.7	119.7	131.7
2019	Callao	Women	60+	Healthy	122.6	118.7	126.5
2019	Callao	Women	60+	Unaware	156.4	150.1	162.7
2019	Callao	Women	60+	Aware not treated	157.9	126.7	189.1
2019	Callao	Women	60+	Aware treated	145.1	137.9	152.3
2020	Callao	Women	<60	Healthy	113.0	111.4	114.7
2020	Callao	Women	<60	Unaware	144.5	140.4	148.6
2020	Callao	Women	<60	Aware not treated	132.1	117.5	146.7
2020	Callao	Women	<60	Aware treated	129.8	119.1	140.6
2020	Callao	Women	60+	Healthy	119.1	112.9	125.2

2020	Callao	Women	60+	Unaware	161.9	152.9	170.9
2020	Callao	Women	60+	Aware not treated	165.6	128.8	202.4
2020	Callao	Women	60+	Aware treated	144.8	136.4	153.2
2015	Cusco	Men	<60	Healthy	117.1	115.5	118.8
2015	Cusco	Men	<60	Unaware	152.0	140.2	163.7
2015	Cusco	Men	<60	Aware not treated	123.9	115.5	132.2
2015	Cusco	Men	<60	Aware treated	132.0	119.6	144.4
2015	Cusco	Men	60+	Healthy	114.8	111.2	118.5
2015	Cusco	Men	60+	Unaware	151.5	146.7	156.2
2015	Cusco	Men	60+	Aware not treated	126.4	118.7	134.1
2015	Cusco	Men	60+	Aware treated	161.3	147.3	175.4
2016	Cusco	Men	<60	Healthy	118.5	117.0	120.0
2016	Cusco	Men	<60	Unaware	145.9	141.2	150.6
2016	Cusco	Men	<60	Aware not treated	127.2	115.2	139.2
2016	Cusco	Men	<60	Aware treated	117.4	111.7	123.2
2016	Cusco	Men	60+	Healthy	117.6	113.7	121.5
2016	Cusco	Men	60+	Unaware	158.6	149.9	167.4
2016	Cusco	Men	60+	Aware not treated	138.0	131.3	144.7
2016	Cusco	Men	60+	Aware treated	150.2	135.7	164.7
2017	Cusco	Men	<60	Healthy	116.3	114.4	118.1
2017	Cusco	Men	<60	Unaware	153.0	143.5	162.4
2017	Cusco	Men	<60	Aware not treated	129.2	123.3	135.2
2017	Cusco	Men	<60	Aware treated	128.1	110.1	146.0
2017	Cusco	Men	60+	Healthy	119.6	116.8	122.4
2017	Cusco	Men	60+	Unaware	152.4	145.1	159.7
2017	Cusco	Men	60+	Aware not treated	134.7	118.3	151.0
2017	Cusco	Men	60+	Aware treated	148.4	141.0	155.8
2018	Cusco	Men	<60	Healthy	118.1	116.4	119.7
2018	Cusco	Men	<60	Unaware	150.5	140.9	160.2

2018	Cusco	Men	<60	Aware not treated	145.1	127.0	163.1
2018	Cusco	Men	<60	Aware treated	132.9	115.6	150.1
2018	Cusco	Men	60+	Healthy	117.5	114.2	120.8
2018	Cusco	Men	60+	Unaware	152.0	140.7	163.2
2018	Cusco	Men	60+	Aware not treated	132.7	115.3	150.1
2018	Cusco	Men	60+	Aware treated	161.1	138.7	183.4
2019	Cusco	Men	<60	Healthy	118.6	116.5	120.6
2019	Cusco	Men	<60	Unaware	147.9	142.9	152.8
2019	Cusco	Men	<60	Aware not treated	122.8	111.8	133.9
2019	Cusco	Men	<60	Aware treated	129.1	119.2	139.0
2019	Cusco	Men	60+	Healthy	117.5	113.6	121.3
2019	Cusco	Men	60+	Unaware	157.7	149.1	166.4
2019	Cusco	Men	60+	Aware not treated	131.6	121.1	142.2
2019	Cusco	Men	60+	Aware treated	139.1	129.5	148.7
2020	Cusco	Men	<60	Healthy	118.0	116.3	119.6
2020	Cusco	Men	<60	Unaware	141.2	135.5	146.9
2020	Cusco	Men	<60	Aware not treated	126.2	114.9	137.5
2020	Cusco	Men	<60	Aware treated	140.0	127.5	152.6
2020	Cusco	Men	60+	Healthy	115.6	111.8	119.3
2020	Cusco	Men	60+	Unaware	147.5	139.1	155.9
2020	Cusco	Men	60+	Aware treated	143.2	122.3	164.0
2015	Cusco	Women	<60	Healthy	108.9	107.4	110.4
2015	Cusco	Women	<60	Unaware	155.5	141.1	170.0
2015	Cusco	Women	<60	Aware not treated	117.3	107.3	127.3
2015	Cusco	Women	<60	Aware treated	110.2	101.2	119.3
2015	Cusco	Women	60+	Healthy	113.4	109.4	117.4
2015	Cusco	Women	60+	Unaware	157.4	148.4	166.4
2015	Cusco	Women	60+	Aware not treated	125.4	101.3	149.5
2015	Cusco	Women	60+	Aware treated	139.7	126.8	152.6

2016	Cusco	Women	<60	Healthy	110.6	109.1	112.1
2016	Cusco	Women	<60	Unaware	143.1	138.8	147.4
2016	Cusco	Women	<60	Aware not treated	116.1	107.2	125.0
2016	Cusco	Women	<60	Aware treated	119.5	110.6	128.4
2016	Cusco	Women	60+	Healthy	115.6	111.8	119.4
2016	Cusco	Women	60+	Unaware	154.9	146.1	163.7
2016	Cusco	Women	60+	Aware not treated	127.6	111.7	143.4
2016	Cusco	Women	60+	Aware treated	138.3	121.6	154.9
2017	Cusco	Women	<60	Healthy	110.0	108.4	111.6
2017	Cusco	Women	<60	Unaware	141.6	137.4	145.7
2017	Cusco	Women	<60	Aware not treated	118.1	103.7	132.5
2017	Cusco	Women	<60	Aware treated	109.2	91.1	127.2
2017	Cusco	Women	60+	Healthy	115.4	112.6	118.1
2017	Cusco	Women	60+	Unaware	155.2	151.0	159.3
2017	Cusco	Women	60+	Aware not treated	143.3	121.6	165.0
2017	Cusco	Women	60+	Aware treated	138.7	127.2	150.1
2018	Cusco	Women	<60	Healthy	108.7	107.4	110.0
2018	Cusco	Women	<60	Unaware	148.9	139.3	158.6
2018	Cusco	Women	<60	Aware not treated	115.3	109.2	121.3
2018	Cusco	Women	<60	Aware treated	123.9	115.9	131.9
2018	Cusco	Women	60+	Healthy	117.2	112.9	121.5
2018	Cusco	Women	60+	Unaware	164.0	148.3	179.7
2018	Cusco	Women	60+	Aware not treated	142.3	129.2	155.4
2018	Cusco	Women	60+	Aware treated	141.7	127.3	156.1
2019	Cusco	Women	<60	Healthy	110.5	108.9	112.1
2019	Cusco	Women	<60	Unaware	150.5	143.1	157.9
2019	Cusco	Women	<60	Aware not treated	110.0	102.5	117.4
2019	Cusco	Women	<60	Aware treated	112.6	104.3	120.9
2019	Cusco	Women	60+	Healthy	118.0	114.8	121.2

2019	Cusco	Women	60+	Unaware	157.2	145.2	169.2
2019	Cusco	Women	60+	Aware not treated	120.9	110.8	131.0
2019	Cusco	Women	60+	Aware treated	142.7	124.5	161.0
2020	Cusco	Women	<60	Healthy	110.5	108.7	112.3
2020	Cusco	Women	<60	Unaware	144.1	137.8	150.4
2020	Cusco	Women	<60	Aware not treated	118.8	108.7	129.0
2020	Cusco	Women	<60	Aware treated	125.3	114.0	136.5
2020	Cusco	Women	60+	Healthy	116.1	111.8	120.4
2020	Cusco	Women	60+	Unaware	152.7	144.8	160.6
2020	Cusco	Women	60+	Aware not treated	124.4	97.2	151.5
2020	Cusco	Women	60+	Aware treated	121.9	103.1	140.8
2015	Huancavelica	Men	<60	Healthy	116.3	114.5	118.0
2015	Huancavelica	Men	<60	Unaware	149.4	140.4	158.3
2015	Huancavelica	Men	<60	Aware not treated	120.5	112.7	128.4
2015	Huancavelica	Men	<60	Aware treated	128.1	117.2	139.0
2015	Huancavelica	Men	60+	Healthy	118.6	115.7	121.5
2015	Huancavelica	Men	60+	Unaware	157.3	149.5	165.1
2015	Huancavelica	Men	60+	Aware not treated	139.3	124.0	154.6
2015	Huancavelica	Men	60+	Aware treated	131.6	115.7	147.5
2016	Huancavelica	Men	<60	Healthy	116.1	114.7	117.5
2016	Huancavelica	Men	<60	Unaware	145.3	140.0	150.6
2016	Huancavelica	Men	<60	Aware not treated	124.9	118.3	131.5
2016	Huancavelica	Men	<60	Aware treated	121.0	108.1	133.8
2016	Huancavelica	Men	60+	Healthy	117.1	114.0	120.3
2016	Huancavelica	Men	60+	Unaware	150.0	144.7	155.3
2016	Huancavelica	Men	60+	Aware not treated	142.1	122.1	162.1
2016	Huancavelica	Men	60+	Aware treated	157.3	133.1	181.4
2017	Huancavelica	Men	<60	Healthy	116.1	114.5	117.7
2017	Huancavelica	Men	<60	Unaware	150.1	145.1	155.1

2017	Huancavelica	Men	<60	Aware not treated	112.6	106.0	119.1
2017	Huancavelica	Men	<60	Aware treated	127.3	114.3	140.4
2017	Huancavelica	Men	60+	Healthy	117.9	114.8	121.0
2017	Huancavelica	Men	60+	Unaware	154.4	148.5	160.3
2017	Huancavelica	Men	60+	Aware not treated	121.4	110.1	132.7
2017	Huancavelica	Men	60+	Aware treated	144.3	132.4	156.3
2018	Huancavelica	Men	<60	Healthy	117.8	116.3	119.3
2018	Huancavelica	Men	<60	Unaware	151.1	142.8	159.3
2018	Huancavelica	Men	<60	Aware not treated	133.8	126.8	140.8
2018	Huancavelica	Men	<60	Aware treated	122.0	110.1	134.0
2018	Huancavelica	Men	60+	Healthy	115.1	112.1	118.2
2018	Huancavelica	Men	60+	Unaware	155.3	150.3	160.3
2018	Huancavelica	Men	60+	Aware not treated	120.7	103.6	137.7
2018	Huancavelica	Men	60+	Aware treated	142.2	126.7	157.8
2019	Huancavelica	Men	<60	Healthy	115.6	113.9	117.4
2019	Huancavelica	Men	<60	Unaware	149.2	138.1	160.3
2019	Huancavelica	Men	<60	Aware not treated	117.9	112.1	123.8
2019	Huancavelica	Men	<60	Aware treated	124.4	117.5	131.4
2019	Huancavelica	Men	60+	Healthy	116.1	112.8	119.3
2019	Huancavelica	Men	60+	Unaware	161.1	154.7	167.5
2019	Huancavelica	Men	60+	Aware not treated	141.6	137.5	145.7
2019	Huancavelica	Men	60+	Aware treated	142.2	126.7	157.8
2020	Huancavelica	Men	<60	Healthy	117.3	115.6	118.9
2020	Huancavelica	Men	<60	Unaware	146.0	141.4	150.7
2020	Huancavelica	Men	<60	Aware not treated	127.2	110.2	144.1
2020	Huancavelica	Men	<60	Aware treated	131.1	120.2	141.9
2020	Huancavelica	Men	60+	Healthy	121.0	117.2	124.8
2020	Huancavelica	Men	60+	Unaware	153.8	149.2	158.4
2020	Huancavelica	Men	60+	Aware not treated	159.2	118.0	200.4

2020	Huancavelica	Men	60+	Aware treated	154.9	144.4	165.4
2015	Huancavelica	Women	<60	Healthy	109.0	107.4	110.5
2015	Huancavelica	Women	<60	Unaware	147.5	140.7	154.4
2015	Huancavelica	Women	<60	Aware not treated	114.5	108.4	120.7
2015	Huancavelica	Women	<60	Aware treated	121.2	110.9	131.5
2015	Huancavelica	Women	60+	Healthy	117.7	114.0	121.4
2015	Huancavelica	Women	60+	Unaware	156.1	150.0	162.2
2015	Huancavelica	Women	60+	Aware not treated	139.2	116.5	161.9
2015	Huancavelica	Women	60+	Aware treated	125.6	112.7	138.6
2016	Huancavelica	Women	<60	Healthy	109.0	107.6	110.4
2016	Huancavelica	Women	<60	Unaware	148.1	140.3	155.8
2016	Huancavelica	Women	<60	Aware not treated	112.5	107.7	117.2
2016	Huancavelica	Women	<60	Aware treated	114.7	105.2	124.1
2016	Huancavelica	Women	60+	Healthy	117.6	114.0	121.2
2016	Huancavelica	Women	60+	Unaware	159.3	151.0	167.6
2016	Huancavelica	Women	60+	Aware not treated	134.5	115.8	153.3
2016	Huancavelica	Women	60+	Aware treated	147.6	135.2	159.9
2017	Huancavelica	Women	<60	Healthy	108.6	107.4	109.9
2017	Huancavelica	Women	<60	Unaware	153.6	144.8	162.4
2017	Huancavelica	Women	<60	Aware not treated	111.8	106.3	117.4
2017	Huancavelica	Women	<60	Aware treated	121.3	109.8	132.7
2017	Huancavelica	Women	60+	Healthy	114.9	111.9	117.9
2017	Huancavelica	Women	60+	Unaware	158.9	153.2	164.7
2017	Huancavelica	Women	60+	Aware not treated	113.6	99.6	127.6
2017	Huancavelica	Women	60+	Aware treated	150.9	135.2	166.7
2018	Huancavelica	Women	<60	Healthy	109.0	107.5	110.5
2018	Huancavelica	Women	<60	Unaware	144.8	143.3	146.4
2018	Huancavelica	Women	<60	Aware not treated	115.0	107.9	122.1
2018	Huancavelica	Women	<60	Aware treated	124.7	114.6	134.7

2018	Huancavelica	Women	60+	Healthy	116.0	113.3	118.7
2018	Huancavelica	Women	60+	Unaware	156.6	152.0	161.3
2018	Huancavelica	Women	60+	Aware not treated	130.0	121.2	138.7
2018	Huancavelica	Women	60+	Aware treated	137.4	127.7	147.1
2019	Huancavelica	Women	<60	Healthy	110.1	108.5	111.6
2019	Huancavelica	Women	<60	Unaware	155.1	143.0	167.3
2019	Huancavelica	Women	<60	Aware not treated	117.4	108.2	126.7
2019	Huancavelica	Women	<60	Aware treated	111.9	106.7	117.2
2019	Huancavelica	Women	60+	Healthy	115.5	113.4	117.6
2019	Huancavelica	Women	60+	Unaware	155.5	149.4	161.5
2019	Huancavelica	Women	60+	Aware not treated	130.4	123.1	137.7
2019	Huancavelica	Women	60+	Aware treated	143.4	128.4	158.3
2020	Huancavelica	Women	<60	Healthy	109.6	107.9	111.2
2020	Huancavelica	Women	<60	Unaware	162.0	151.3	172.6
2020	Huancavelica	Women	<60	Aware not treated	126.0	114.7	137.2
2020	Huancavelica	Women	<60	Aware treated	130.4	111.8	149.1
2020	Huancavelica	Women	60+	Healthy	116.7	113.5	119.8
2020	Huancavelica	Women	60+	Unaware	156.1	146.1	166.1
2020	Huancavelica	Women	60+	Aware not treated	148.3	125.8	170.8
2020	Huancavelica	Women	60+	Aware treated	141.3	128.2	154.3

Supplementary Table 4. At the national level, mean diastolic blood pressure (95% confidence intervals) by population group stratified by sex and study year

Year	Sex	Age	Categories	Mean DBP	Lower limit	Upper limit
2015	Men	<60	Healthy	72.2	71.9	72.5
2015	Men	<60	Unaware	88.4	87.4	89.4
2015	Men	<60	Aware not treated	79.9	77.8	82.1
2015	Men	<60	Aware treated	83.3	80.6	86.1
2015	Women	<60	Healthy	68.6	68.3	68.8
2015	Women	<60	Unaware	86.9	85.5	88.2
2015	Women	<60	Aware not treated	74.0	72.7	75.4
2015	Women	<60	Aware treated	76.6	75.3	78.0
2015	Men	60+	Healthy	68.3	67.7	69.0
2015	Men	60+	Unaware	80.3	79.1	81.5
2015	Men	60+	Aware not treated	73.8	71.2	76.3
2015	Men	60+	Aware treated	75.8	74.1	77.5
2015	Women	60+	Healthy	65.6	65.0	66.2
2015	Women	60+	Unaware	75.9	74.6	77.2
2015	Women	60+	Aware not treated	70.9	68.6	73.2
2015	Women	60+	Aware treated	72.2	70.9	73.6
2016	Men	<60	Healthy	72.4	72.1	72.7
2016	Men	<60	Unaware	88.5	87.7	89.3
2016	Men	<60	Aware not treated	80.6	78.2	83.0
2016	Men	<60	Aware treated	84.2	80.9	87.5
2016	Women	<60	Healthy	68.7	68.5	69.0
2016	Women	<60	Unaware	85.3	83.9	86.6
2016	Women	<60	Aware not treated	73.9	72.4	75.4
2016	Women	<60	Aware treated	78.0	76.4	79.7
2016	Men	60+	Healthy	69.6	68.9	70.2
2016	Men	60+	Unaware	80.1	78.8	81.5
2016	Men	60+	Aware not treated	76.4	73.4	79.4
2016	Men	60+	Aware treated	75.9	74.4	77.4
2016	Women	60+	Healthy	65.2	64.4	65.9
2016	Women	60+	Unaware	75.6	74.3	76.8
2016	Women	60+	Aware not treated	72.0	69.8	74.2
2016	Women	60+	Aware treated	70.6	69.3	71.9
2017	Men	<60	Healthy	72.7	72.4	73.1
2017	Men	<60	Unaware	88.7	87.5	89.9
2017	Men	<60	Aware not treated	79.9	77.9	81.9
2017	Men	<60	Aware treated	83.2	80.4	85.9
2017	Women	<60	Healthy	68.8	68.5	69.1
2017	Women	<60	Unaware	85.6	84.3	86.8

2017	Women	<60	Aware not treated	71.9	70.5	73.4
2017	Women	<60	Aware treated	77.0	75.4	78.7
2017	Men	60+	Healthy	68.7	67.9	69.6
2017	Men	60+	Unaware	80.1	78.8	81.4
2017	Men	60+	Aware not treated	75.2	72.2	78.2
2017	Men	60+	Aware treated	75.8	74.2	77.4
2017	Women	60+	Healthy	66.1	65.3	66.8
2017	Women	60+	Unaware	76.0	74.8	77.3
2017	Women	60+	Aware not treated	71.8	69.3	74.4
2017	Women	60+	Aware treated	71.6	70.3	73.0
2018	Men	<60	Healthy	74.1	73.8	74.4
2018	Men	<60	Unaware	89.7	88.9	90.5
2018	Men	<60	Aware not treated	81.9	79.1	84.8
2018	Men	<60	Aware treated	84.7	82.1	87.4
2018	Women	<60	Healthy	70.1	69.9	70.4
2018	Women	<60	Unaware	88.2	87.0	89.4
2018	Women	<60	Aware not treated	75.8	74.2	77.5
2018	Women	<60	Aware treated	78.9	77.3	80.5
2018	Men	60+	Healthy	71.0	70.4	71.7
2018	Men	60+	Unaware	83.1	81.4	84.8
2018	Men	60+	Aware not treated	80.3	76.5	84.0
2018	Men	60+	Aware treated	77.5	75.5	79.5
2018	Women	60+	Healthy	67.1	66.4	67.8
2018	Women	60+	Unaware	77.1	75.4	78.7
2018	Women	60+	Aware not treated	72.5	70.5	74.4
2018	Women	60+	Aware treated	73.8	72.4	75.1
2019	Men	<60	Healthy	73.4	73.1	73.7
2019	Men	<60	Unaware	89.3	88.5	90.2
2019	Men	<60	Aware not treated	80.6	78.6	82.5
2019	Men	<60	Aware treated	84.2	81.4	87.0
2019	Women	<60	Healthy	69.8	69.5	70.1
2019	Women	<60	Unaware	87.3	86.0	88.7
2019	Women	<60	Aware not treated	75.7	73.7	77.7
2019	Women	<60	Aware treated	78.5	76.8	80.1
2019	Men	60+	Healthy	69.7	69.0	70.4
2019	Men	60+	Unaware	82.6	81.0	84.2
2019	Men	60+	Aware not treated	75.7	73.2	78.2
2019	Men	60+	Aware treated	75.4	73.5	77.4
2019	Women	60+	Healthy	67.1	66.4	67.7
2019	Women	60+	Unaware	77.0	75.4	78.5
2019	Women	60+	Aware not treated	72.4	69.9	75.0
2019	Women	60+	Aware treated	72.2	70.9	73.5
2020	Men	<60	Healthy	73.7	73.3	74.1
2020	Men	<60	Unaware	90.5	89.5	91.5
2020	Men	<60	Aware not treated	83.2	80.2	86.1

2020	Men	<60	Aware treated	82.4	79.0	85.9
2020	Women	<60	Healthy	70.4	70.0	70.8
2020	Women	<60	Unaware	87.2	86.0	88.5
2020	Women	<60	Aware not treated	77.1	74.4	79.9
2020	Women	<60	Aware treated	78.9	76.8	81.0
2020	Men	60+	Healthy	70.6	69.7	71.5
2020	Men	60+	Unaware	81.7	79.7	83.7
2020	Men	60+	Aware not treated	79.3	76.1	82.5
2020	Men	60+	Aware treated	77.1	74.0	80.3
2020	Women	60+	Healthy	66.5	65.7	67.2
2020	Women	60+	Unaware	78.5	76.9	80.1
2020	Women	60+	Aware not treated	75.6	72.5	78.7
2020	Women	60+	Aware treated	71.2	69.4	72.9

Supplementary Table 5. At the sub-national level, mean diastolic blood pressure (95% confidence intervals) by population group stratified by sex and study year

Year	Region	Sex	Age	Categories	Mean DBP	Lowe limit	Upper limit
2015	Amazonas	Men	<60	Healthy	70.5	69.4	71.6
2015	Amazonas	Men	<60	Unaware	85.3	82.1	88.5
2015	Amazonas	Men	<60	Aware not treated	76.1	68.8	83.4
2015	Amazonas	Men	<60	Aware treated	91.9	79.9	103.8
2015	Amazonas	Men	60+	Healthy	69.2	67.2	71.3
2015	Amazonas	Men	60+	Unaware	81.0	74.7	87.3
2015	Amazonas	Men	60+	Aware not treated	69.4	65.4	73.4
2015	Amazonas	Men	60+	Aware treated	79.4	73.8	84.9
2016	Amazonas	Men	<60	Healthy	71.2	70.2	72.2
2016	Amazonas	Men	<60	Unaware	88.7	85.9	91.5
2016	Amazonas	Men	<60	Aware not treated	82.8	75.1	90.6
2016	Amazonas	Men	<60	Aware treated	88.9	81.1	96.8
2016	Amazonas	Men	60+	Healthy	65.5	63.3	67.7
2016	Amazonas	Men	60+	Unaware	85.0	76.7	93.3
2016	Amazonas	Men	60+	Aware not treated	76.0	66.8	85.3
2016	Amazonas	Men	60+	Aware treated	83.4	78.1	88.8
2017	Amazonas	Men	<60	Healthy	72.0	70.9	73.1
2017	Amazonas	Men	<60	Unaware	92.1	89.4	94.8
2017	Amazonas	Men	<60	Aware not treated	83.9	78.4	89.3
2017	Amazonas	Men	<60	Aware treated	82.0	76.7	87.4
2017	Amazonas	Men	60+	Healthy	68.2	64.7	71.7
2017	Amazonas	Men	60+	Unaware	77.2	68.4	86.1
2017	Amazonas	Men	60+	Aware not treated	97.7	83.5	112.0

2017	Amazonas	Men	60+	Aware treated	79.2	71.0	87.4
2018	Amazonas	Men	<60	Healthy	73.0	72.0	74.0
2018	Amazonas	Men	<60	Unaware	89.1	86.4	91.8
2018	Amazonas	Men	<60	Aware not treated	79.2	75.7	82.8
2018	Amazonas	Men	<60	Aware treated	87.3	73.9	100.7
2018	Amazonas	Men	60+	Healthy	70.1	67.8	72.3
2018	Amazonas	Men	60+	Unaware	83.2	77.8	88.6
2018	Amazonas	Men	60+	Aware not treated	77.4	65.5	89.4
2018	Amazonas	Men	60+	Aware treated	82.6	69.8	95.3
2019	Amazonas	Men	<60	Healthy	72.4	71.4	73.5
2019	Amazonas	Men	<60	Unaware	91.6	88.3	94.9
2019	Amazonas	Men	<60	Aware not treated	78.0	72.1	84.0
2019	Amazonas	Men	<60	Aware treated	80.9	75.7	86.1
2019	Amazonas	Men	60+	Healthy	67.5	65.0	70.0
2019	Amazonas	Men	60+	Unaware	78.4	74.5	82.4
2019	Amazonas	Men	60+	Aware not treated	80.4	72.6	88.2
2019	Amazonas	Men	60+	Aware treated	77.9	72.1	83.7
2020	Amazonas	Men	<60	Healthy	71.3	70.1	72.5
2020	Amazonas	Men	<60	Unaware	86.1	80.8	91.5
2020	Amazonas	Men	<60	Aware not treated	78.0	71.4	84.6
2020	Amazonas	Men	<60	Aware treated	80.8	71.1	90.5
2020	Amazonas	Men	60+	Healthy	67.4	64.7	70.1
2020	Amazonas	Men	60+	Unaware	72.4	67.0	77.8
2020	Amazonas	Men	60+	Aware not treated	70.0	70.0	70.0
2020	Amazonas	Men	60+	Aware treated	74.4	70.1	78.6
2015	Amazonas	Women	<60	Healthy	70.1	69.1	71.1
2015	Amazonas	Women	<60	Unaware	87.4	84.3	90.4
2015	Amazonas	Women	<60	Aware not treated	78.9	72.1	85.6
2015	Amazonas	Women	<60	Aware treated	84.9	73.2	96.6

2015	Amazonas	Women	60+	Healthy	67.7	64.0	71.5
2015	Amazonas	Women	60+	Unaware	74.8	70.4	79.3
2015	Amazonas	Women	60+	Aware not treated	66.3	53.3	79.3
2015	Amazonas	Women	60+	Aware treated	75.4	68.7	82.2
2016	Amazonas	Women	<60	Healthy	69.1	68.0	70.1
2016	Amazonas	Women	<60	Unaware	84.3	79.8	88.9
2016	Amazonas	Women	<60	Aware not treated	78.2	72.4	84.1
2016	Amazonas	Women	<60	Aware treated	85.9	73.9	98.0
2016	Amazonas	Women	60+	Healthy	65.2	62.5	67.8
2016	Amazonas	Women	60+	Unaware	74.1	68.1	80.1
2016	Amazonas	Women	60+	Aware not treated	79.2	69.5	89.0
2016	Amazonas	Women	60+	Aware treated	70.3	65.0	75.5
2017	Amazonas	Women	<60	Healthy	70.0	69.0	71.0
2017	Amazonas	Women	<60	Unaware	87.7	82.7	92.7
2017	Amazonas	Women	<60	Aware not treated	79.2	66.8	91.6
2017	Amazonas	Women	<60	Aware treated	79.7	74.4	84.9
2017	Amazonas	Women	60+	Healthy	66.1	62.6	69.5
2017	Amazonas	Women	60+	Unaware	74.4	72.0	76.8
2017	Amazonas	Women	60+	Aware not treated	69.4	66.7	72.2
2017	Amazonas	Women	60+	Aware treated	75.2	71.6	78.8
2018	Amazonas	Women	<60	Healthy	71.8	70.8	72.9
2018	Amazonas	Women	<60	Unaware	90.5	84.3	96.8
2018	Amazonas	Women	<60	Aware not treated	73.7	68.1	79.4
2018	Amazonas	Women	<60	Aware treated	82.2	76.8	87.7
2018	Amazonas	Women	60+	Healthy	68.2	66.0	70.4
2018	Amazonas	Women	60+	Unaware	84.6	78.9	90.4
2018	Amazonas	Women	60+	Aware not treated	69.8	62.5	77.2
2018	Amazonas	Women	60+	Aware treated	77.4	70.9	83.9
2019	Amazonas	Women	<60	Healthy	70.1	69.1	71.2

2019	Amazonas	Women	<60	Unaware	85.2	81.6	88.9
2019	Amazonas	Women	<60	Aware not treated	69.5	65.0	74.0
2019	Amazonas	Women	<60	Aware treated	77.1	72.2	82.0
2019	Amazonas	Women	60+	Healthy	67.6	64.7	70.5
2019	Amazonas	Women	60+	Unaware	78.2	74.2	82.3
2019	Amazonas	Women	60+	Aware not treated	65.0	53.9	76.1
2019	Amazonas	Women	60+	Aware treated	72.8	68.7	76.8
2020	Amazonas	Women	<60	Healthy	70.4	68.9	71.8
2020	Amazonas	Women	<60	Unaware	92.1	87.2	96.9
2020	Amazonas	Women	<60	Aware not treated	76.0	60.8	91.2
2020	Amazonas	Women	<60	Aware treated	76.5	68.5	84.6
2020	Amazonas	Women	60+	Healthy	66.6	63.0	70.1
2020	Amazonas	Women	60+	Unaware	74.3	70.1	78.5
2020	Amazonas	Women	60+	Aware not treated	68.5	62.1	74.8
2020	Amazonas	Women	60+	Aware treated	71.5	64.7	78.3
2015	Huanuco	Men	<60	Healthy	71.5	70.5	72.4
2015	Huanuco	Men	<60	Unaware	90.0	88.1	91.9
2015	Huanuco	Men	<60	Aware not treated	67.2	61.9	72.5
2015	Huanuco	Men	<60	Aware treated	81.4	73.9	88.9
2015	Huanuco	Men	60+	Healthy	68.3	65.9	70.7
2015	Huanuco	Men	60+	Unaware	76.1	72.5	79.8
2015	Huanuco	Men	60+	Aware not treated	77.9	74.6	81.2
2015	Huanuco	Men	60+	Aware treated	79.4	67.8	91.1
2016	Huanuco	Men	<60	Healthy	70.6	69.3	72.0
2016	Huanuco	Men	<60	Unaware	87.4	84.2	90.5
2016	Huanuco	Men	<60	Aware not treated	74.4	68.5	80.3
2016	Huanuco	Men	<60	Aware treated	82.0	76.8	87.2
2016	Huanuco	Men	60+	Healthy	67.0	65.0	69.0
2016	Huanuco	Men	60+	Unaware	75.2	71.2	79.2

2016	Huanuco	Men	60+	Aware treated	78.5	74.3	82.6
2017	Huanuco	Men	<60	Healthy	70.6	69.5	71.8
2017	Huanuco	Men	<60	Unaware	88.7	86.1	91.3
2017	Huanuco	Men	<60	Aware not treated	72.1	67.9	76.3
2017	Huanuco	Men	<60	Aware treated	74.8	72.0	77.5
2017	Huanuco	Men	60+	Healthy	66.8	65.0	68.6
2017	Huanuco	Men	60+	Unaware	81.0	75.3	86.8
2017	Huanuco	Men	60+	Aware not treated	71.6	67.9	75.2
2017	Huanuco	Men	60+	Aware treated	75.6	63.0	88.2
2018	Huanuco	Men	<60	Healthy	73.3	72.4	74.3
2018	Huanuco	Men	<60	Unaware	89.6	87.0	92.2
2018	Huanuco	Men	<60	Aware not treated	79.3	75.5	83.1
2018	Huanuco	Men	<60	Aware treated	86.1	75.4	96.7
2018	Huanuco	Men	60+	Healthy	71.0	68.1	73.8
2018	Huanuco	Men	60+	Unaware	77.9	73.6	82.3
2018	Huanuco	Men	60+	Aware not treated	88.6	70.1	107.1
2018	Huanuco	Men	60+	Aware treated	77.8	70.3	85.4
2019	Huanuco	Men	<60	Healthy	73.3	72.3	74.4
2019	Huanuco	Men	<60	Unaware	90.5	86.5	94.6
2019	Huanuco	Men	<60	Aware not treated	77.6	72.1	83.2
2019	Huanuco	Men	<60	Aware treated	67.6	59.9	75.3
2019	Huanuco	Men	60+	Healthy	71.8	69.2	74.5
2019	Huanuco	Men	60+	Unaware	84.8	80.7	88.8
2019	Huanuco	Men	60+	Aware not treated	68.1	55.4	80.7
2019	Huanuco	Men	60+	Aware treated	80.5	75.3	85.6
2020	Huanuco	Men	<60	Healthy	71.3	69.9	72.7
2020	Huanuco	Men	<60	Unaware	87.6	84.7	90.5
2020	Huanuco	Men	<60	Aware not treated	78.8	73.6	84.1
2020	Huanuco	Men	60+	Healthy	72.4	69.4	75.3

2020	Huanuco	Men	60+	Unaware	83.3	76.2	90.3
2020	Huanuco	Men	60+	Aware not treated	82.6	74.9	90.2
2020	Huanuco	Men	60+	Aware treated	73.1	65.6	80.7
2015	Huanuco	Women	<60	Healthy	68.9	68.0	69.8
2015	Huanuco	Women	<60	Unaware	86.7	82.5	90.9
2015	Huanuco	Women	<60	Aware not treated	76.4	73.0	79.8
2015	Huanuco	Women	<60	Aware treated	78.4	71.7	85.0
2015	Huanuco	Women	60+	Healthy	67.4	65.7	69.0
2015	Huanuco	Women	60+	Unaware	78.0	72.4	83.7
2015	Huanuco	Women	60+	Aware not treated	70.5	67.0	74.1
2015	Huanuco	Women	60+	Aware treated	71.6	66.3	76.9
2016	Huanuco	Women	<60	Healthy	68.5	67.5	69.6
2016	Huanuco	Women	<60	Unaware	81.1	72.9	89.3
2016	Huanuco	Women	<60	Aware not treated	73.0	65.7	80.2
2016	Huanuco	Women	<60	Aware treated	73.9	69.6	78.1
2016	Huanuco	Women	60+	Healthy	66.2	64.6	67.9
2016	Huanuco	Women	60+	Unaware	75.5	71.1	79.9
2016	Huanuco	Women	60+	Aware not treated	72.2	65.9	78.6
2016	Huanuco	Women	60+	Aware treated	72.4	65.3	79.6
2017	Huanuco	Women	<60	Healthy	67.1	66.2	68.0
2017	Huanuco	Women	<60	Unaware	86.2	80.7	91.7
2017	Huanuco	Women	<60	Aware not treated	64.3	60.8	67.8
2017	Huanuco	Women	<60	Aware treated	77.1	69.1	85.1
2017	Huanuco	Women	60+	Healthy	64.2	62.1	66.3
2017	Huanuco	Women	60+	Unaware	73.9	69.6	78.3
2017	Huanuco	Women	60+	Aware not treated	71.3	58.8	83.7
2017	Huanuco	Women	60+	Aware treated	68.4	62.6	74.3
2018	Huanuco	Women	<60	Healthy	70.2	69.1	71.3
2018	Huanuco	Women	<60	Unaware	88.7	84.8	92.6

2018	Huanuco	Women	<60	Aware not treated	76.0	71.6	80.5
2018	Huanuco	Women	<60	Aware treated	79.1	71.9	86.3
2018	Huanuco	Women	60+	Healthy	66.6	64.1	69.1
2018	Huanuco	Women	60+	Unaware	78.0	73.4	82.6
2018	Huanuco	Women	60+	Aware not treated	68.5	63.9	73.1
2018	Huanuco	Women	60+	Aware treated	71.4	56.3	86.6
2019	Huanuco	Women	<60	Healthy	70.0	68.8	71.2
2019	Huanuco	Women	<60	Unaware	92.3	89.5	95.2
2019	Huanuco	Women	<60	Aware not treated	78.2	71.9	84.4
2019	Huanuco	Women	<60	Aware treated	74.6	67.9	81.2
2019	Huanuco	Women	60+	Healthy	66.5	64.4	68.6
2019	Huanuco	Women	60+	Unaware	80.8	75.2	86.5
2019	Huanuco	Women	60+	Aware not treated	69.0	66.6	71.3
2019	Huanuco	Women	60+	Aware treated	73.7	68.6	78.7
2020	Huanuco	Women	<60	Healthy	70.3	68.8	71.8
2020	Huanuco	Women	<60	Unaware	87.4	82.2	92.5
2020	Huanuco	Women	<60	Aware not treated	72.5	68.8	76.2
2020	Huanuco	Women	<60	Aware treated	77.0	71.4	82.6
2020	Huanuco	Women	60+	Healthy	66.8	64.4	69.2
2020	Huanuco	Women	60+	Unaware	76.5	67.5	85.4
2020	Huanuco	Women	60+	Aware not treated	75.1	70.3	80.0
2020	Huanuco	Women	60+	Aware treated	71.4	63.6	79.3
2015	Ica	Men	<60	Healthy	72.3	71.3	73.3
2015	Ica	Men	<60	Unaware	86.5	84.1	89.0
2015	Ica	Men	<60	Aware not treated	82.6	60.2	104.9
2015	Ica	Men	<60	Aware treated	84.6	71.3	97.8
2015	Ica	Men	60+	Healthy	68.5	65.4	71.6
2015	Ica	Men	60+	Unaware	82.6	76.2	89.0
2015	Ica	Men	60+	Aware not treated	69.3	58.5	80.1

2015	Ica	Men	60+	Aware treated	75.1	66.3	83.8
2016	Ica	Men	<60	Healthy	72.3	70.9	73.6
2016	Ica	Men	<60	Unaware	90.1	87.6	92.6
2016	Ica	Men	<60	Aware not treated	78.1	68.7	87.5
2016	Ica	Men	<60	Aware treated	95.8	85.8	105.8
2016	Ica	Men	60+	Healthy	67.3	63.9	70.7
2016	Ica	Men	60+	Unaware	77.5	74.7	80.2
2016	Ica	Men	60+	Aware not treated	71.4	59.8	83.0
2016	Ica	Men	60+	Aware treated	78.6	73.3	84.0
2017	Ica	Men	<60	Healthy	73.5	72.4	74.5
2017	Ica	Men	<60	Unaware	84.2	80.4	88.1
2017	Ica	Men	<60	Aware not treated	86.5	80.5	92.4
2017	Ica	Men	<60	Aware treated	81.3	73.9	88.6
2017	Ica	Men	60+	Healthy	70.3	67.6	73.0
2017	Ica	Men	60+	Unaware	79.2	76.1	82.3
2017	Ica	Men	60+	Aware not treated	77.7	73.5	82.0
2017	Ica	Men	60+	Aware treated	76.0	71.0	81.1
2018	Ica	Men	<60	Healthy	76.0	74.9	77.0
2018	Ica	Men	<60	Unaware	93.7	90.0	97.3
2018	Ica	Men	<60	Aware not treated	82.0	72.1	91.8
2018	Ica	Men	<60	Aware treated	87.2	80.3	94.1
2018	Ica	Men	60+	Healthy	69.3	66.9	71.8
2018	Ica	Men	60+	Unaware	86.1	81.4	90.9
2018	Ica	Men	60+	Aware not treated	77.0	71.1	82.9
2018	Ica	Men	60+	Aware treated	75.5	69.8	81.2
2019	Ica	Men	<60	Healthy	74.6	73.5	75.7
2019	Ica	Men	<60	Unaware	92.7	90.7	94.7
2019	Ica	Men	<60	Aware not treated	83.9	74.3	93.5
2019	Ica	Men	<60	Aware treated	78.9	72.5	85.4

2019	Ica	Men	60+	Healthy	72.1	69.6	74.6
2019	Ica	Men	60+	Unaware	82.4	74.6	90.2
2019	Ica	Men	60+	Aware not treated	88.5	81.5	95.5
2019	Ica	Men	60+	Aware treated	76.9	71.6	82.3
2020	Ica	Men	<60	Healthy	75.1	73.5	76.8
2020	Ica	Men	<60	Unaware	89.5	85.8	93.1
2020	Ica	Men	<60	Aware not treated	89.0	76.2	101.9
2020	Ica	Men	<60	Aware treated	79.8	73.0	86.6
2020	Ica	Men	60+	Healthy	71.5	67.5	75.6
2020	Ica	Men	60+	Unaware	83.3	76.1	90.6
2020	Ica	Men	60+	Aware not treated	76.2	64.0	88.3
2020	Ica	Men	60+	Aware treated	68.1	61.0	75.2
2015	Ica	Women	<60	Healthy	68.7	67.4	70.0
2015	Ica	Women	<60	Unaware	84.5	81.6	87.5
2015	Ica	Women	<60	Aware not treated	75.1	68.6	81.6
2015	Ica	Women	<60	Aware treated	77.0	72.6	81.4
2015	Ica	Women	60+	Healthy	66.2	63.2	69.3
2015	Ica	Women	60+	Unaware	80.6	72.7	88.5
2015	Ica	Women	60+	Aware not treated	81.2	75.2	87.2
2015	Ica	Women	60+	Aware treated	69.6	65.3	74.0
2016	Ica	Women	<60	Healthy	70.1	69.1	71.1
2016	Ica	Women	<60	Unaware	84.7	80.6	88.8
2016	Ica	Women	<60	Aware not treated	78.9	63.3	94.6
2016	Ica	Women	<60	Aware treated	72.5	65.2	79.9
2016	Ica	Women	60+	Healthy	62.3	59.5	65.1
2016	Ica	Women	60+	Unaware	75.2	70.5	80.0
2016	Ica	Women	60+	Aware not treated	63.8	57.2	70.3
2016	Ica	Women	60+	Aware treated	67.0	63.1	70.9
2017	Ica	Women	<60	Healthy	69.4	68.5	70.4

2017	Ica	Women	<60	Unaware	84.5	80.9	88.0
2017	Ica	Women	<60	Aware not treated	80.5	71.3	89.7
2017	Ica	Women	<60	Aware treated	77.3	71.0	83.7
2017	Ica	Women	60+	Healthy	64.3	61.9	66.8
2017	Ica	Women	60+	Unaware	78.0	73.0	83.0
2017	Ica	Women	60+	Aware not treated	74.0	67.4	80.5
2017	Ica	Women	60+	Aware treated	72.8	69.6	76.0
2018	Ica	Women	<60	Healthy	69.9	68.8	71.0
2018	Ica	Women	<60	Unaware	84.7	80.9	88.6
2018	Ica	Women	<60	Aware not treated	78.5	72.5	84.4
2018	Ica	Women	<60	Aware treated	74.5	70.2	78.8
2018	Ica	Women	60+	Healthy	69.1	67.0	71.3
2018	Ica	Women	60+	Unaware	76.7	73.1	80.3
2018	Ica	Women	60+	Aware not treated	77.5	70.5	84.5
2018	Ica	Women	60+	Aware treated	76.5	71.3	81.8
2019	Ica	Women	<60	Healthy	70.9	69.9	71.9
2019	Ica	Women	<60	Unaware	89.2	83.9	94.6
2019	Ica	Women	<60	Aware not treated	74.3	66.9	81.8
2019	Ica	Women	<60	Aware treated	86.9	81.6	92.2
2019	Ica	Women	60+	Healthy	69.1	66.1	72.1
2019	Ica	Women	60+	Unaware	79.0	72.4	85.7
2019	Ica	Women	60+	Aware not treated	71.2	66.0	76.5
2019	Ica	Women	60+	Aware treated	72.4	67.9	77.0
2020	Ica	Women	<60	Healthy	70.0	68.7	71.3
2020	Ica	Women	<60	Unaware	84.3	79.0	89.6
2020	Ica	Women	<60	Aware not treated	80.8	73.3	88.3
2020	Ica	Women	<60	Aware treated	82.0	76.3	87.7
2020	Ica	Women	60+	Healthy	67.4	63.1	71.8
2020	Ica	Women	60+	Unaware	76.2	63.4	89.0

2020	Ica	Women	60+	Aware not treated	90.1	77.6	102.7
2020	Ica	Women	60+	Aware treated	71.4	66.6	76.2
2015	Junin	Men	<60	Healthy	72.7	71.5	73.8
2015	Junin	Men	<60	Unaware	89.7	87.3	92.2
2015	Junin	Men	<60	Aware not treated	78.8	72.2	85.4
2015	Junin	Men	<60	Aware treated	75.4	64.3	86.6
2015	Junin	Men	60+	Healthy	69.2	67.1	71.4
2015	Junin	Men	60+	Unaware	79.5	74.2	84.8
2015	Junin	Men	60+	Aware not treated	72.1	63.1	81.0
2015	Junin	Men	60+	Aware treated	73.0	64.1	81.9
2016	Junin	Men	<60	Healthy	71.0	69.5	72.4
2016	Junin	Men	<60	Unaware	90.2	86.8	93.5
2016	Junin	Men	<60	Aware not treated	76.7	71.0	82.3
2016	Junin	Men	<60	Aware treated	84.4	68.4	100.3
2016	Junin	Men	60+	Healthy	69.4	66.8	72.0
2016	Junin	Men	60+	Unaware	79.5	73.3	85.6
2016	Junin	Men	60+	Aware not treated	76.0	76.0	76.0
2016	Junin	Men	60+	Aware treated	75.0	57.7	92.3
2017	Junin	Men	<60	Healthy	73.2	71.9	74.4
2017	Junin	Men	<60	Unaware	91.0	88.7	93.2
2017	Junin	Men	<60	Aware not treated	79.0	74.2	83.9
2017	Junin	Men	<60	Aware treated	82.3	71.0	93.6
2017	Junin	Men	60+	Healthy	68.5	65.8	71.1
2017	Junin	Men	60+	Unaware	80.8	77.6	84.1
2017	Junin	Men	60+	Aware not treated	83.9	72.5	95.3
2017	Junin	Men	60+	Aware treated	72.4	59.5	85.3
2018	Junin	Men	<60	Healthy	73.9	72.6	75.1
2018	Junin	Men	<60	Unaware	87.8	84.2	91.3
2018	Junin	Men	<60	Aware not treated	79.9	74.4	85.5

2018	Junin	Men	<60	Aware treated	85.9	77.6	94.2
2018	Junin	Men	60+	Healthy	73.3	69.8	76.7
2018	Junin	Men	60+	Unaware	80.3	74.5	86.2
2018	Junin	Men	60+	Aware not treated	91.2	85.7	96.7
2018	Junin	Men	60+	Aware treated	85.4	79.5	91.4
2019	Junin	Men	<60	Healthy	74.9	73.8	76.1
2019	Junin	Men	<60	Unaware	93.4	89.7	97.2
2019	Junin	Men	<60	Aware not treated	83.3	77.1	89.5
2019	Junin	Men	<60	Aware treated	80.5	73.0	88.0
2019	Junin	Men	60+	Healthy	73.5	70.3	76.7
2019	Junin	Men	60+	Unaware	81.0	75.4	86.7
2019	Junin	Men	60+	Aware not treated	75.4	66.9	83.9
2019	Junin	Men	60+	Aware treated	81.3	77.7	84.9
2020	Junin	Men	<60	Healthy	73.6	72.0	75.3
2020	Junin	Men	<60	Unaware	92.9	88.0	97.9
2020	Junin	Men	<60	Aware not treated	77.3	76.7	78.0
2020	Junin	Men	<60	Aware treated	65.7	61.4	70.0
2020	Junin	Men	60+	Healthy	71.0	67.4	74.5
2020	Junin	Men	60+	Unaware	74.0	70.2	77.9
2020	Junin	Men	60+	Aware not treated	83.0	83.0	83.0
2020	Junin	Men	60+	Aware treated	84.6	76.9	92.3
2015	Junin	Women	<60	Healthy	68.5	67.2	69.8
2015	Junin	Women	<60	Unaware	87.5	82.0	93.1
2015	Junin	Women	<60	Aware not treated	69.4	65.3	73.5
2015	Junin	Women	<60	Aware treated	72.6	68.2	77.0
2015	Junin	Women	60+	Healthy	66.3	63.3	69.3
2015	Junin	Women	60+	Unaware	78.7	72.3	85.1
2015	Junin	Women	60+	Aware not treated	74.1	68.3	79.9
2015	Junin	Women	60+	Aware treated	72.3	65.4	79.1

2016	Junin	Women	<60	Healthy	68.7	67.7	69.7
2016	Junin	Women	<60	Unaware	87.5	82.6	92.3
2016	Junin	Women	<60	Aware not treated	69.8	65.0	74.5
2016	Junin	Women	<60	Aware treated	71.6	59.5	83.7
2016	Junin	Women	60+	Healthy	63.8	61.3	66.2
2016	Junin	Women	60+	Unaware	79.5	75.4	83.6
2016	Junin	Women	60+	Aware not treated	64.7	54.3	75.1
2016	Junin	Women	60+	Aware treated	71.4	66.7	76.2
2017	Junin	Women	<60	Healthy	69.0	68.0	70.1
2017	Junin	Women	<60	Unaware	85.3	81.0	89.6
2017	Junin	Women	<60	Aware not treated	69.2	64.1	74.4
2017	Junin	Women	<60	Aware treated	68.3	60.9	75.6
2017	Junin	Women	60+	Healthy	68.3	66.4	70.2
2017	Junin	Women	60+	Unaware	74.9	66.5	83.3
2017	Junin	Women	60+	Aware not treated	72.2	65.9	78.4
2017	Junin	Women	60+	Aware treated	69.6	66.3	73.0
2018	Junin	Women	<60	Healthy	71.9	71.0	72.9
2018	Junin	Women	<60	Unaware	90.1	86.3	93.9
2018	Junin	Women	<60	Aware not treated	78.8	74.2	83.4
2018	Junin	Women	<60	Aware treated	80.6	74.1	87.0
2018	Junin	Women	60+	Healthy	69.4	67.1	71.7
2018	Junin	Women	60+	Unaware	87.7	82.8	92.5
2018	Junin	Women	60+	Aware not treated	68.5	65.7	71.3
2018	Junin	Women	60+	Aware treated	73.6	69.2	78.0
2019	Junin	Women	<60	Healthy	72.3	71.4	73.1
2019	Junin	Women	<60	Unaware	85.9	78.1	93.6
2019	Junin	Women	<60	Aware not treated	75.2	72.6	77.8
2019	Junin	Women	<60	Aware treated	80.3	74.2	86.4
2019	Junin	Women	60+	Healthy	71.0	68.0	74.0

2019	Junin	Women	60+	Unaware	80.2	72.5	87.9
2019	Junin	Women	60+	Aware not treated	83.8	82.0	85.6
2019	Junin	Women	60+	Aware treated	85.7	78.9	92.4
2020	Junin	Women	<60	Healthy	71.4	70.2	72.5
2020	Junin	Women	<60	Unaware	91.5	89.2	93.7
2020	Junin	Women	<60	Aware not treated	78.1	73.4	82.7
2020	Junin	Women	<60	Aware treated	79.1	67.9	90.4
2020	Junin	Women	60+	Healthy	68.2	65.2	71.1
2020	Junin	Women	60+	Unaware	76.4	70.0	82.9
2020	Junin	Women	60+	Aware not treated	75.9	69.7	82.2
2020	Junin	Women	60+	Aware treated	78.2	73.7	82.6
2015	La Libertad	Men	<60	Healthy	69.8	68.6	71.0
2015	La Libertad	Men	<60	Unaware	87.6	83.6	91.5
2015	La Libertad	Men	<60	Aware not treated	78.0	70.9	85.0
2015	La Libertad	Men	<60	Aware treated	87.8	86.2	89.4
2015	La Libertad	Men	60+	Healthy	65.3	62.9	67.6
2015	La Libertad	Men	60+	Unaware	76.0	70.5	81.5
2015	La Libertad	Men	60+	Aware not treated	77.6	67.4	87.9
2015	La Libertad	Men	60+	Aware treated	73.2	68.6	77.9
2016	La Libertad	Men	<60	Healthy	71.3	70.2	72.5
2016	La Libertad	Men	<60	Unaware	84.9	81.5	88.3
2016	La Libertad	Men	<60	Aware not treated	88.7	75.6	101.8
2016	La Libertad	Men	<60	Aware treated	66.0	66.0	66.0
2016	La Libertad	Men	60+	Healthy	69.9	67.8	72.1
2016	La Libertad	Men	60+	Unaware	78.1	72.8	83.4
2016	La Libertad	Men	60+	Aware not treated	83.1	75.0	91.3
2016	La Libertad	Men	60+	Aware treated	77.7	70.6	84.8
2017	La Libertad	Men	<60	Healthy	70.0	68.8	71.2
2017	La Libertad	Men	<60	Unaware	89.1	84.2	93.9

2017	La Libertad	Men	<60	Aware not treated	91.1	79.5	102.7
2017	La Libertad	Men	<60	Aware treated	76.4	71.0	81.8
2017	La Libertad	Men	60+	Healthy	66.6	64.1	69.1
2017	La Libertad	Men	60+	Unaware	80.9	76.9	84.8
2017	La Libertad	Men	60+	Aware not treated	81.9	61.1	102.7
2017	La Libertad	Men	60+	Aware treated	73.2	68.3	78.0
2018	La Libertad	Men	<60	Healthy	72.5	71.4	73.5
2018	La Libertad	Men	<60	Unaware	91.8	89.3	94.2
2018	La Libertad	Men	<60	Aware not treated	82.8	72.7	93.0
2018	La Libertad	Men	<60	Aware treated	77.3	65.1	89.5
2018	La Libertad	Men	60+	Healthy	70.7	68.4	73.1
2018	La Libertad	Men	60+	Unaware	81.3	75.8	86.7
2018	La Libertad	Men	60+	Aware not treated	66.9	66.7	67.1
2018	La Libertad	Men	60+	Aware treated	77.8	73.1	82.5
2019	La Libertad	Men	<60	Healthy	71.2	69.9	72.5
2019	La Libertad	Men	<60	Unaware	87.3	82.9	91.6
2019	La Libertad	Men	<60	Aware not treated	78.5	66.1	90.8
2019	La Libertad	Men	<60	Aware treated	79.4	68.0	90.9
2019	La Libertad	Men	60+	Healthy	67.3	64.2	70.5
2019	La Libertad	Men	60+	Unaware	81.2	78.2	84.2
2019	La Libertad	Men	60+	Aware not treated	71.2	61.9	80.5
2019	La Libertad	Men	60+	Aware treated	68.8	63.3	74.4
2020	La Libertad	Men	<60	Healthy	72.0	70.3	73.8
2020	La Libertad	Men	<60	Unaware	85.8	81.3	90.4
2020	La Libertad	Men	<60	Aware not treated	76.6	69.2	84.0
2020	La Libertad	Men	<60	Aware treated	76.0	76.0	76.0
2020	La Libertad	Men	60+	Healthy	68.1	64.1	72.0
2020	La Libertad	Men	60+	Unaware	81.6	75.3	87.9
2020	La Libertad	Men	60+	Aware not treated	81.2	58.0	104.3

2020	La Libertad	Men	60+	Aware treated	76.7	66.3	87.1
2015	La Libertad	Women	<60	Healthy	67.1	65.7	68.4
2015	La Libertad	Women	<60	Unaware	88.2	82.6	93.9
2015	La Libertad	Women	<60	Aware not treated	68.9	64.3	73.6
2015	La Libertad	Women	<60	Aware treated	74.8	70.4	79.2
2015	La Libertad	Women	60+	Healthy	65.5	62.9	68.1
2015	La Libertad	Women	60+	Unaware	73.1	70.7	75.6
2015	La Libertad	Women	60+	Aware not treated	68.8	61.3	76.3
2015	La Libertad	Women	60+	Aware treated	71.9	67.5	76.4
2016	La Libertad	Women	<60	Healthy	67.6	66.5	68.8
2016	La Libertad	Women	<60	Unaware	83.8	75.1	92.6
2016	La Libertad	Women	<60	Aware not treated	73.5	71.9	75.1
2016	La Libertad	Women	<60	Aware treated	77.6	73.1	82.1
2016	La Libertad	Women	60+	Healthy	65.1	61.7	68.6
2016	La Libertad	Women	60+	Unaware	73.3	68.2	78.5
2016	La Libertad	Women	60+	Aware not treated	66.1	59.7	72.6
2016	La Libertad	Women	60+	Aware treated	67.8	64.4	71.2
2017	La Libertad	Women	<60	Healthy	67.5	66.3	68.7
2017	La Libertad	Women	<60	Unaware	83.6	79.2	88.0
2017	La Libertad	Women	<60	Aware not treated	67.9	62.7	73.1
2017	La Libertad	Women	<60	Aware treated	71.7	65.2	78.2
2017	La Libertad	Women	60+	Healthy	66.0	64.1	67.8
2017	La Libertad	Women	60+	Unaware	74.1	70.8	77.4
2017	La Libertad	Women	60+	Aware not treated	69.9	61.2	78.5
2017	La Libertad	Women	60+	Aware treated	73.3	66.9	79.7
2018	La Libertad	Women	<60	Healthy	69.6	68.6	70.6
2018	La Libertad	Women	<60	Unaware	89.6	84.9	94.3
2018	La Libertad	Women	<60	Aware not treated	75.0	70.7	79.3
2018	La Libertad	Women	<60	Aware treated	84.7	78.9	90.4

2018	La Libertad	Women	60+	Healthy	67.6	64.9	70.3
2018	La Libertad	Women	60+	Unaware	73.8	67.2	80.4
2018	La Libertad	Women	60+	Aware not treated	76.0	67.6	84.5
2018	La Libertad	Women	60+	Aware treated	74.4	68.6	80.2
2019	La Libertad	Women	<60	Healthy	69.0	67.8	70.2
2019	La Libertad	Women	<60	Unaware	84.9	74.1	95.8
2019	La Libertad	Women	<60	Aware not treated	73.5	67.3	79.8
2019	La Libertad	Women	<60	Aware treated	75.6	71.2	80.1
2019	La Libertad	Women	60+	Healthy	65.4	62.8	67.9
2019	La Libertad	Women	60+	Unaware	74.9	70.7	79.1
2019	La Libertad	Women	60+	Aware not treated	69.6	63.2	76.1
2019	La Libertad	Women	60+	Aware treated	72.9	68.8	77.0
2020	La Libertad	Women	<60	Healthy	68.4	67.0	69.8
2020	La Libertad	Women	<60	Unaware	85.3	80.0	90.6
2020	La Libertad	Women	<60	Aware not treated	70.5	62.7	78.2
2020	La Libertad	Women	<60	Aware treated	72.1	65.8	78.3
2020	La Libertad	Women	60+	Healthy	65.3	62.6	68.0
2020	La Libertad	Women	60+	Unaware	73.8	67.0	80.5
2020	La Libertad	Women	60+	Aware not treated	67.6	57.2	78.0
2020	La Libertad	Women	60+	Aware treated	66.8	61.7	71.8
2015	Lambayeque	Men	<60	Healthy	71.9	70.7	73.0
2015	Lambayeque	Men	<60	Unaware	88.3	85.6	91.0
2015	Lambayeque	Men	<60	Aware not treated	77.3	74.6	80.0
2015	Lambayeque	Men	<60	Aware treated	84.6	71.1	98.1
2015	Lambayeque	Men	60+	Healthy	68.1	66.2	70.0
2015	Lambayeque	Men	60+	Unaware	82.6	76.4	88.9
2015	Lambayeque	Men	60+	Aware not treated	79.3	77.7	80.8
2015	Lambayeque	Men	60+	Aware treated	74.7	71.2	78.2
2016	Lambayeque	Men	<60	Healthy	72.1	71.1	73.1

2016	Lambayeque	Men	<60	Unaware	86.1	83.3	88.9
2016	Lambayeque	Men	<60	Aware not treated	84.3	63.0	105.6
2016	Lambayeque	Men	<60	Aware treated	76.5	64.1	88.9
2016	Lambayeque	Men	60+	Healthy	69.3	66.7	71.9
2016	Lambayeque	Men	60+	Unaware	80.4	75.7	85.2
2016	Lambayeque	Men	60+	Aware not treated	74.9	64.1	85.8
2016	Lambayeque	Men	60+	Aware treated	75.9	69.1	82.6
2017	Lambayeque	Men	<60	Healthy	71.1	70.0	72.2
2017	Lambayeque	Men	<60	Unaware	86.8	83.5	90.2
2017	Lambayeque	Men	<60	Aware not treated	68.4	68.4	68.4
2017	Lambayeque	Men	<60	Aware treated	86.0	76.1	95.8
2017	Lambayeque	Men	60+	Healthy	67.2	65.5	68.9
2017	Lambayeque	Men	60+	Unaware	77.5	74.7	80.3
2017	Lambayeque	Men	60+	Aware not treated	81.0	81.0	81.0
2017	Lambayeque	Men	60+	Aware treated	75.4	71.2	79.7
2018	Lambayeque	Men	<60	Healthy	75.4	74.5	76.4
2018	Lambayeque	Men	<60	Unaware	90.3	87.1	93.5
2018	Lambayeque	Men	<60	Aware not treated	74.8	61.2	88.4
2018	Lambayeque	Men	<60	Aware treated	78.7	74.3	83.1
2018	Lambayeque	Men	60+	Healthy	74.1	71.9	76.3
2018	Lambayeque	Men	60+	Unaware	86.9	81.9	91.8
2018	Lambayeque	Men	60+	Aware not treated	82.7	72.3	93.1
2018	Lambayeque	Men	60+	Aware treated	76.0	71.4	80.5
2019	Lambayeque	Men	<60	Healthy	73.2	72.4	74.0
2019	Lambayeque	Men	<60	Unaware	88.2	85.1	91.4
2019	Lambayeque	Men	<60	Aware not treated	92.5	88.8	96.2
2019	Lambayeque	Men	<60	Aware treated	88.7	79.4	98.0
2019	Lambayeque	Men	60+	Healthy	70.9	68.1	73.6
2019	Lambayeque	Men	60+	Unaware	79.8	72.3	87.4

2019	Lambayeque	Men	60+	Aware not treated	74.3	60.7	87.9
2019	Lambayeque	Men	60+	Aware treated	69.6	64.4	74.7
2020	Lambayeque	Men	<60	Healthy	73.6	72.3	74.9
2020	Lambayeque	Men	<60	Unaware	92.1	88.5	95.7
2020	Lambayeque	Men	<60	Aware not treated	90.4	83.5	97.3
2020	Lambayeque	Men	<60	Aware treated	80.6	76.0	85.2
2020	Lambayeque	Men	60+	Healthy	70.9	66.8	74.9
2020	Lambayeque	Men	60+	Unaware	79.2	68.0	90.4
2020	Lambayeque	Men	60+	Aware not treated	82.0	82.0	82.0
2020	Lambayeque	Men	60+	Aware treated	78.6	73.3	83.9
2015	Lambayeque	Women	<60	Healthy	67.8	66.6	68.9
2015	Lambayeque	Women	<60	Unaware	90.8	83.9	97.7
2015	Lambayeque	Women	<60	Aware not treated	76.1	66.9	85.3
2015	Lambayeque	Women	<60	Aware treated	81.9	76.5	87.3
2015	Lambayeque	Women	60+	Healthy	63.9	61.5	66.3
2015	Lambayeque	Women	60+	Unaware	74.1	67.1	81.1
2015	Lambayeque	Women	60+	Aware not treated	72.8	66.6	79.0
2015	Lambayeque	Women	60+	Aware treated	71.2	67.4	75.1
2016	Lambayeque	Women	<60	Healthy	68.3	67.3	69.3
2016	Lambayeque	Women	<60	Unaware	87.6	81.7	93.5
2016	Lambayeque	Women	<60	Aware not treated	74.2	72.4	76.1
2016	Lambayeque	Women	<60	Aware treated	76.8	73.3	80.2
2016	Lambayeque	Women	60+	Healthy	65.0	62.1	67.9
2016	Lambayeque	Women	60+	Unaware	71.6	65.2	78.0
2016	Lambayeque	Women	60+	Aware not treated	82.0	82.0	82.0
2016	Lambayeque	Women	60+	Aware treated	70.7	67.8	73.7
2017	Lambayeque	Women	<60	Healthy	67.5	66.5	68.5
2017	Lambayeque	Women	<60	Unaware	85.3	79.4	91.2
2017	Lambayeque	Women	<60	Aware not treated	75.8	69.0	82.5

2017	Lambayeque	Women	<60	Aware treated	76.2	71.4	80.9
2017	Lambayeque	Women	60+	Healthy	65.8	63.2	68.5
2017	Lambayeque	Women	60+	Unaware	73.0	66.5	79.5
2017	Lambayeque	Women	60+	Aware not treated	81.0	81.0	81.0
2017	Lambayeque	Women	60+	Aware treated	66.4	62.7	70.0
2018	Lambayeque	Women	<60	Healthy	70.8	69.8	71.8
2018	Lambayeque	Women	<60	Unaware	87.8	83.8	91.8
2018	Lambayeque	Women	<60	Aware not treated	74.7	66.9	82.5
2018	Lambayeque	Women	<60	Aware treated	82.4	77.2	87.7
2018	Lambayeque	Women	60+	Healthy	66.8	65.1	68.6
2018	Lambayeque	Women	60+	Unaware	78.2	70.3	86.1
2018	Lambayeque	Women	60+	Aware not treated	62.0	62.0	62.0
2018	Lambayeque	Women	60+	Aware treated	78.3	72.7	83.9
2019	Lambayeque	Women	<60	Healthy	69.7	68.6	70.8
2019	Lambayeque	Women	<60	Unaware	90.6	86.6	94.7
2019	Lambayeque	Women	<60	Aware not treated	66.3	58.7	73.8
2019	Lambayeque	Women	<60	Aware treated	71.9	65.9	77.8
2019	Lambayeque	Women	60+	Healthy	68.0	65.2	70.9
2019	Lambayeque	Women	60+	Unaware	78.9	73.2	84.5
2019	Lambayeque	Women	60+	Aware not treated	76.3	66.3	86.3
2019	Lambayeque	Women	60+	Aware treated	74.1	69.8	78.4
2020	Lambayeque	Women	<60	Healthy	69.4	68.0	70.8
2020	Lambayeque	Women	<60	Unaware	86.3	79.0	93.5
2020	Lambayeque	Women	<60	Aware not treated	93.5	65.1	121.9
2020	Lambayeque	Women	<60	Aware treated	77.8	72.8	82.8
2020	Lambayeque	Women	60+	Healthy	67.4	64.7	70.1
2020	Lambayeque	Women	60+	Unaware	80.6	77.5	83.6
2020	Lambayeque	Women	60+	Aware not treated	70.0	70.0	70.0
2020	Lambayeque	Women	60+	Aware treated	67.5	61.4	73.5

2015	Lima	Men	<60	Healthy	73.7	73.0	74.4
2015	Lima	Men	<60	Unaware	88.5	86.6	90.4
2015	Lima	Men	<60	Aware not treated	84.6	79.0	90.1
2015	Lima	Men	<60	Aware treated	86.6	79.8	93.3
2015	Lima	Men	60+	Healthy	69.6	67.9	71.3
2015	Lima	Men	60+	Unaware	81.0	77.4	84.7
2015	Lima	Men	60+	Aware not treated	74.8	69.2	80.4
2015	Lima	Men	60+	Aware treated	77.1	73.5	80.7
2016	Lima	Men	<60	Healthy	73.5	72.8	74.2
2016	Lima	Men	<60	Unaware	89.2	87.5	90.9
2016	Lima	Men	<60	Aware not treated	85.1	79.3	90.9
2016	Lima	Men	<60	Aware treated	86.4	79.9	92.8
2016	Lima	Men	60+	Healthy	71.1	69.6	72.7
2016	Lima	Men	60+	Unaware	80.3	76.9	83.8
2016	Lima	Men	60+	Aware not treated	72.7	65.7	79.6
2016	Lima	Men	60+	Aware treated	75.6	73.1	78.1
2017	Lima	Men	<60	Healthy	73.6	72.9	74.3
2017	Lima	Men	<60	Unaware	89.3	86.9	91.7
2017	Lima	Men	<60	Aware not treated	81.5	77.3	85.8
2017	Lima	Men	<60	Aware treated	88.7	82.2	95.2
2017	Lima	Men	60+	Healthy	69.4	67.2	71.7
2017	Lima	Men	60+	Unaware	80.3	77.4	83.1
2017	Lima	Men	60+	Aware not treated	72.6	67.1	78.1
2017	Lima	Men	60+	Aware treated	75.7	72.7	78.8
2018	Lima	Men	<60	Healthy	74.4	73.8	75.0
2018	Lima	Men	<60	Unaware	89.1	87.6	90.6
2018	Lima	Men	<60	Aware not treated	86.0	76.4	95.7
2018	Lima	Men	<60	Aware treated	88.1	81.8	94.4
2018	Lima	Men	60+	Healthy	71.8	70.5	73.2

2018	Lima	Men	60+	Unaware	83.3	79.8	86.8
2018	Lima	Men	60+	Aware not treated	85.0	76.6	93.5
2018	Lima	Men	60+	Aware treated	77.0	72.8	81.2
2019	Lima	Men	<60	Healthy	73.7	73.1	74.3
2019	Lima	Men	<60	Unaware	88.5	86.7	90.3
2019	Lima	Men	<60	Aware not treated	81.2	76.9	85.5
2019	Lima	Men	<60	Aware treated	84.7	79.9	89.5
2019	Lima	Men	60+	Healthy	68.3	66.6	70.1
2019	Lima	Men	60+	Unaware	84.1	80.8	87.4
2019	Lima	Men	60+	Aware not treated	77.6	73.1	82.1
2019	Lima	Men	60+	Aware treated	76.5	72.9	80.2
2020	Lima	Men	<60	Healthy	74.8	74.0	75.6
2020	Lima	Men	<60	Unaware	90.9	89.3	92.5
2020	Lima	Men	<60	Aware not treated	86.1	78.2	94.0
2020	Lima	Men	<60	Aware treated	83.2	77.1	89.3
2020	Lima	Men	60+	Healthy	71.9	69.3	74.4
2020	Lima	Men	60+	Unaware	81.2	77.8	84.6
2020	Lima	Men	60+	Aware not treated	81.2	77.4	84.9
2020	Lima	Men	60+	Aware treated	78.7	72.8	84.6
2015	Lima	Women	<60	Healthy	68.6	67.9	69.2
2015	Lima	Women	<60	Unaware	87.4	84.0	90.7
2015	Lima	Women	<60	Aware not treated	75.8	72.5	79.1
2015	Lima	Women	<60	Aware treated	76.1	73.6	78.6
2015	Lima	Women	60+	Healthy	66.3	64.7	67.9
2015	Lima	Women	60+	Unaware	76.0	72.8	79.1
2015	Lima	Women	60+	Aware not treated	67.3	59.5	75.1
2015	Lima	Women	60+	Aware treated	72.6	69.7	75.6
2016	Lima	Women	<60	Healthy	68.9	68.2	69.6
2016	Lima	Women	<60	Unaware	85.3	82.6	88.0

2016	Lima	Women	<60	Aware not treated	74.1	69.0	79.1
2016	Lima	Women	<60	Aware treated	80.5	75.4	85.6
2016	Lima	Women	60+	Healthy	64.4	62.1	66.8
2016	Lima	Women	60+	Unaware	75.4	72.2	78.6
2016	Lima	Women	60+	Aware not treated	76.3	69.1	83.4
2016	Lima	Women	60+	Aware treated	70.2	67.2	73.2
2017	Lima	Women	<60	Healthy	69.1	68.4	69.8
2017	Lima	Women	<60	Unaware	84.7	82.7	86.8
2017	Lima	Women	<60	Aware not treated	70.7	67.5	73.9
2017	Lima	Women	<60	Aware treated	78.1	74.7	81.5
2017	Lima	Women	60+	Healthy	67.0	65.2	68.8
2017	Lima	Women	60+	Unaware	75.9	73.2	78.5
2017	Lima	Women	60+	Aware not treated	70.2	64.6	75.7
2017	Lima	Women	60+	Aware treated	71.6	68.9	74.2
2018	Lima	Women	<60	Healthy	69.9	69.3	70.5
2018	Lima	Women	<60	Unaware	87.9	85.2	90.7
2018	Lima	Women	<60	Aware not treated	77.0	72.6	81.5
2018	Lima	Women	<60	Aware treated	78.7	75.0	82.3
2018	Lima	Women	60+	Healthy	65.6	64.0	67.2
2018	Lima	Women	60+	Unaware	75.7	72.8	78.6
2018	Lima	Women	60+	Aware not treated	73.0	68.9	77.0
2018	Lima	Women	60+	Aware treated	72.7	70.2	75.1
2019	Lima	Women	<60	Healthy	69.6	68.9	70.2
2019	Lima	Women	<60	Unaware	86.5	83.6	89.3
2019	Lima	Women	<60	Aware not treated	79.2	73.0	85.4
2019	Lima	Women	<60	Aware treated	79.7	76.2	83.2
2019	Lima	Women	60+	Healthy	66.5	64.9	68.1
2019	Lima	Women	60+	Unaware	75.5	72.8	78.3
2019	Lima	Women	60+	Aware not treated	72.7	66.8	78.6

2019	Lima	Women	60+	Aware treated	71.9	69.3	74.5
2020	Lima	Women	<60	Healthy	70.9	70.0	71.8
2020	Lima	Women	<60	Unaware	86.4	84.1	88.8
2020	Lima	Women	<60	Aware not treated	77.1	69.6	84.5
2020	Lima	Women	<60	Aware treated	81.7	77.5	85.8
2020	Lima	Women	60+	Healthy	66.0	64.1	67.9
2020	Lima	Women	60+	Unaware	81.6	78.3	85.0
2020	Lima	Women	60+	Aware not treated	79.7	72.3	87.1
2020	Lima	Women	60+	Aware treated	70.0	66.8	73.2
2015	Loreto	Men	<60	Healthy	71.2	70.1	72.3
2015	Loreto	Men	<60	Unaware	87.5	83.6	91.4
2015	Loreto	Men	<60	Aware not treated	78.3	72.2	84.5
2015	Loreto	Men	<60	Aware treated	82.4	77.2	87.5
2015	Loreto	Men	60+	Healthy	65.1	61.9	68.4
2015	Loreto	Men	60+	Unaware	83.1	78.1	88.1
2015	Loreto	Men	60+	Aware not treated	62.7	57.7	67.7
2015	Loreto	Men	60+	Aware treated	77.0	72.2	81.9
2016	Loreto	Men	<60	Healthy	71.8	70.8	72.9
2016	Loreto	Men	<60	Unaware	86.5	84.3	88.7
2016	Loreto	Men	<60	Aware not treated	86.4	83.1	89.8
2016	Loreto	Men	<60	Aware treated	78.0	73.6	82.4
2016	Loreto	Men	60+	Healthy	66.8	64.0	69.7
2016	Loreto	Men	60+	Unaware	78.9	72.6	85.1
2016	Loreto	Men	60+	Aware not treated	72.8	60.7	84.9
2016	Loreto	Men	60+	Aware treated	74.6	69.7	79.4
2017	Loreto	Men	<60	Healthy	71.1	70.1	72.1
2017	Loreto	Men	<60	Unaware	86.3	83.0	89.6
2017	Loreto	Men	<60	Aware not treated	77.7	69.2	86.3
2017	Loreto	Men	<60	Aware treated	81.4	69.8	93.1

2017	Loreto	Men	60+	Healthy	67.2	64.7	69.7
2017	Loreto	Men	60+	Unaware	81.3	75.9	86.6
2017	Loreto	Men	60+	Aware not treated	66.5	59.6	73.4
2017	Loreto	Men	60+	Aware treated	71.1	67.9	74.3
2018	Loreto	Men	<60	Healthy	74.3	73.1	75.4
2018	Loreto	Men	<60	Unaware	90.0	86.9	93.2
2018	Loreto	Men	<60	Aware not treated	81.0	72.6	89.3
2018	Loreto	Men	<60	Aware treated	90.9	82.6	99.2
2018	Loreto	Men	60+	Healthy	67.6	64.5	70.8
2018	Loreto	Men	60+	Unaware	80.9	76.0	85.8
2018	Loreto	Men	60+	Aware not treated	66.0	66.0	66.0
2018	Loreto	Men	60+	Aware treated	77.4	73.3	81.5
2019	Loreto	Men	<60	Healthy	73.0	72.0	74.0
2019	Loreto	Men	<60	Unaware	90.1	86.9	93.2
2019	Loreto	Men	<60	Aware not treated	79.5	74.8	84.3
2019	Loreto	Men	<60	Aware treated	84.5	72.1	96.9
2019	Loreto	Men	60+	Healthy	70.4	68.1	72.7
2019	Loreto	Men	60+	Unaware	82.0	75.3	88.7
2019	Loreto	Men	60+	Aware not treated	69.0	65.3	72.7
2019	Loreto	Men	60+	Aware treated	71.2	66.4	76.0
2020	Loreto	Men	<60	Healthy	71.9	70.3	73.5
2020	Loreto	Men	<60	Unaware	86.3	83.3	89.3
2020	Loreto	Men	<60	Aware not treated	83.1	76.5	89.7
2020	Loreto	Men	<60	Aware treated	79.5	70.5	88.6
2020	Loreto	Men	60+	Healthy	65.4	61.4	69.3
2020	Loreto	Men	60+	Unaware	74.9	68.4	81.5
2020	Loreto	Men	60+	Aware not treated	64.1	59.7	68.6
2020	Loreto	Men	60+	Aware treated	75.5	63.6	87.3
2015	Loreto	Women	<60	Healthy	68.7	67.5	69.9

2015	Loreto	Women	<60	Unaware	83.4	79.8	86.9
2015	Loreto	Women	<60	Aware not treated	70.3	65.1	75.4
2015	Loreto	Women	<60	Aware treated	72.1	68.8	75.3
2015	Loreto	Women	60+	Healthy	61.1	58.2	64.0
2015	Loreto	Women	60+	Unaware	70.4	64.7	76.1
2015	Loreto	Women	60+	Aware not treated	64.0	51.9	76.1
2015	Loreto	Women	60+	Aware treated	67.6	64.8	70.4
2016	Loreto	Women	<60	Healthy	69.0	68.2	69.9
2016	Loreto	Women	<60	Unaware	79.8	73.7	86.0
2016	Loreto	Women	<60	Aware not treated	73.6	69.1	78.1
2016	Loreto	Women	<60	Aware treated	79.1	75.2	83.0
2016	Loreto	Women	60+	Healthy	66.8	63.8	69.8
2016	Loreto	Women	60+	Unaware	72.4	66.8	78.1
2016	Loreto	Women	60+	Aware not treated	75.5	65.9	85.0
2016	Loreto	Women	60+	Aware treated	69.5	65.7	73.2
2017	Loreto	Women	<60	Healthy	68.3	67.3	69.4
2017	Loreto	Women	<60	Unaware	83.6	76.6	90.7
2017	Loreto	Women	<60	Aware not treated	64.0	58.5	69.5
2017	Loreto	Women	<60	Aware treated	78.4	73.5	83.2
2017	Loreto	Women	60+	Healthy	64.2	61.7	66.8
2017	Loreto	Women	60+	Unaware	73.0	69.4	76.6
2017	Loreto	Women	60+	Aware not treated	71.1	61.3	80.9
2017	Loreto	Women	60+	Aware treated	68.8	64.7	72.8
2018	Loreto	Women	<60	Healthy	70.8	69.7	72.0
2018	Loreto	Women	<60	Unaware	88.7	84.1	93.3
2018	Loreto	Women	<60	Aware not treated	76.8	70.9	82.6
2018	Loreto	Women	<60	Aware treated	76.2	72.6	79.8
2018	Loreto	Women	60+	Healthy	67.9	64.4	71.3
2018	Loreto	Women	60+	Unaware	82.7	76.1	89.4

2018	Loreto	Women	60+	Aware not treated	74.9	70.2	79.6
2018	Loreto	Women	60+	Aware treated	70.5	64.5	76.5
2019	Loreto	Women	<60	Healthy	70.4	69.4	71.4
2019	Loreto	Women	<60	Unaware	88.3	84.9	91.6
2019	Loreto	Women	<60	Aware not treated	72.5	67.6	77.4
2019	Loreto	Women	<60	Aware treated	75.3	71.7	78.8
2019	Loreto	Women	60+	Healthy	66.6	63.4	69.8
2019	Loreto	Women	60+	Unaware	70.0	60.2	79.8
2019	Loreto	Women	60+	Aware not treated	83.1	71.9	94.2
2019	Loreto	Women	60+	Aware treated	69.2	64.3	74.2
2020	Loreto	Women	<60	Healthy	69.4	68.0	70.8
2020	Loreto	Women	<60	Unaware	85.4	79.9	90.9
2020	Loreto	Women	<60	Aware not treated	69.5	63.1	75.8
2020	Loreto	Women	<60	Aware treated	73.2	69.7	76.7
2020	Loreto	Women	60+	Healthy	62.4	59.7	65.0
2020	Loreto	Women	60+	Unaware	72.6	69.7	75.6
2020	Loreto	Women	60+	Aware not treated	66.2	63.0	69.4
2020	Loreto	Women	60+	Aware treated	67.3	61.1	73.5
2015	Madre de Dios	Men	<60	Healthy	69.6	68.6	70.6
2015	Madre de Dios	Men	<60	Unaware	85.7	81.5	89.8
2015	Madre de Dios	Men	<60	Aware not treated	74.8	66.0	83.6
2015	Madre de Dios	Men	<60	Aware treated	79.1	70.0	88.2
2015	Madre de Dios	Men	60+	Healthy	70.2	67.7	72.7
2015	Madre de Dios	Men	60+	Unaware	81.3	76.0	86.5
2015	Madre de Dios	Men	60+	Aware not treated	75.2	70.0	80.5
2015	Madre de Dios	Men	60+	Aware treated	68.7	62.6	74.7
2016	Madre de Dios	Men	<60	Healthy	70.1	69.1	71.0
2016	Madre de Dios	Men	<60	Unaware	86.6	83.7	89.5
2016	Madre de Dios	Men	<60	Aware not treated	75.8	65.3	86.2

2016	Madre de Dios	Men	<60	Aware treated	81.9	67.7	96.2
2016	Madre de Dios	Men	60+	Healthy	69.3	66.2	72.4
2016	Madre de Dios	Men	60+	Unaware	74.0	67.4	80.7
2016	Madre de Dios	Men	60+	Aware not treated	73.8	71.8	75.8
2016	Madre de Dios	Men	60+	Aware treated	72.1	66.2	77.9
2017	Madre de Dios	Men	<60	Healthy	71.0	69.8	72.2
2017	Madre de Dios	Men	<60	Unaware	87.3	84.0	90.6
2017	Madre de Dios	Men	<60	Aware not treated	75.8	69.7	81.8
2017	Madre de Dios	Men	<60	Aware treated	75.4	64.1	86.7
2017	Madre de Dios	Men	60+	Healthy	65.0	62.5	67.4
2017	Madre de Dios	Men	60+	Unaware	81.1	76.4	85.8
2017	Madre de Dios	Men	60+	Aware not treated	86.4	71.8	101.0
2017	Madre de Dios	Men	60+	Aware treated	67.4	60.9	74.0
2018	Madre de Dios	Men	<60	Healthy	72.5	71.2	73.8
2018	Madre de Dios	Men	<60	Unaware	87.8	84.9	90.6
2018	Madre de Dios	Men	<60	Aware not treated	80.4	75.3	85.5
2018	Madre de Dios	Men	<60	Aware treated	84.1	67.9	100.3
2018	Madre de Dios	Men	60+	Healthy	70.2	66.7	73.7
2018	Madre de Dios	Men	60+	Unaware	80.7	75.2	86.2
2018	Madre de Dios	Men	60+	Aware not treated	78.0	68.5	87.4
2018	Madre de Dios	Men	60+	Aware treated	85.7	80.2	91.3
2019	Madre de Dios	Men	<60	Healthy	71.9	70.8	73.1
2019	Madre de Dios	Men	<60	Unaware	91.3	87.9	94.7
2019	Madre de Dios	Men	<60	Aware not treated	84.5	78.7	90.4
2019	Madre de Dios	Men	<60	Aware treated	87.6	78.7	96.4
2019	Madre de Dios	Men	60+	Healthy	68.7	65.2	72.3
2019	Madre de Dios	Men	60+	Unaware	86.9	81.8	92.0
2019	Madre de Dios	Men	60+	Aware not treated	79.6	71.6	87.5
2019	Madre de Dios	Men	60+	Aware treated	73.4	67.1	79.8

2020	Madre de Dios	Men	<60	Healthy	71.3	70.1	72.5
2020	Madre de Dios	Men	<60	Unaware	88.4	85.8	91.1
2020	Madre de Dios	Men	<60	Aware not treated	82.6	77.2	87.9
2020	Madre de Dios	Men	<60	Aware treated	81.4	74.5	88.3
2020	Madre de Dios	Men	60+	Healthy	66.2	63.5	69.0
2020	Madre de Dios	Men	60+	Unaware	77.2	69.7	84.8
2020	Madre de Dios	Men	60+	Aware not treated	73.1	66.6	79.5
2020	Madre de Dios	Men	60+	Aware treated	67.9	61.1	74.7
2015	Madre de Dios	Women	<60	Healthy	66.2	65.1	67.3
2015	Madre de Dios	Women	<60	Unaware	82.3	75.2	89.4
2015	Madre de Dios	Women	<60	Aware not treated	68.4	62.4	74.4
2015	Madre de Dios	Women	<60	Aware treated	68.0	63.9	72.1
2015	Madre de Dios	Women	60+	Healthy	65.2	61.4	69.0
2015	Madre de Dios	Women	60+	Unaware	68.8	62.5	75.1
2015	Madre de Dios	Women	60+	Aware not treated	75.9	62.4	89.5
2015	Madre de Dios	Women	60+	Aware treated	63.9	58.2	69.7
2016	Madre de Dios	Women	<60	Healthy	65.4	64.2	66.6
2016	Madre de Dios	Women	<60	Unaware	90.3	88.8	91.9
2016	Madre de Dios	Women	<60	Aware not treated	70.3	64.7	75.9
2016	Madre de Dios	Women	<60	Aware treated	71.6	65.9	77.2
2016	Madre de Dios	Women	60+	Healthy	65.9	63.1	68.6
2016	Madre de Dios	Women	60+	Unaware	81.0	81.0	81.0
2016	Madre de Dios	Women	60+	Aware not treated	59.3	57.4	61.3
2016	Madre de Dios	Women	60+	Aware treated	66.5	60.0	72.9
2017	Madre de Dios	Women	<60	Healthy	66.1	65.0	67.3
2017	Madre de Dios	Women	<60	Unaware	92.9	83.1	102.7
2017	Madre de Dios	Women	<60	Aware not treated	68.6	62.7	74.5
2017	Madre de Dios	Women	<60	Aware treated	75.0	71.1	78.9
2017	Madre de Dios	Women	60+	Healthy	63.8	61.1	66.6

2017	Madre de Dios	Women	60+	Unaware	70.5	68.2	72.8
2017	Madre de Dios	Women	60+	Aware not treated	59.0	59.0	59.0
2017	Madre de Dios	Women	60+	Aware treated	65.3	61.6	68.9
2018	Madre de Dios	Women	<60	Healthy	68.6	67.4	69.8
2018	Madre de Dios	Women	<60	Unaware	87.4	78.6	96.2
2018	Madre de Dios	Women	<60	Aware not treated	75.5	71.2	79.7
2018	Madre de Dios	Women	<60	Aware treated	76.9	71.6	82.3
2018	Madre de Dios	Women	60+	Healthy	66.6	63.3	69.9
2018	Madre de Dios	Women	60+	Unaware	83.5	74.9	92.1
2018	Madre de Dios	Women	60+	Aware not treated	65.9	62.5	69.2
2018	Madre de Dios	Women	60+	Aware treated	72.9	65.1	80.7
2019	Madre de Dios	Women	<60	Healthy	68.1	66.9	69.3
2019	Madre de Dios	Women	<60	Unaware	88.6	84.7	92.6
2019	Madre de Dios	Women	<60	Aware not treated	71.1	69.1	73.1
2019	Madre de Dios	Women	<60	Aware treated	75.7	70.0	81.3
2019	Madre de Dios	Women	60+	Healthy	67.7	63.7	71.7
2019	Madre de Dios	Women	60+	Unaware	80.9	73.7	88.0
2019	Madre de Dios	Women	60+	Aware not treated	68.4	54.2	82.6
2019	Madre de Dios	Women	60+	Aware treated	72.5	63.6	81.3
2020	Madre de Dios	Women	<60	Healthy	65.5	64.2	66.8
2020	Madre de Dios	Women	<60	Unaware	85.2	77.4	93.1
2020	Madre de Dios	Women	<60	Aware not treated	71.5	63.9	79.1
2020	Madre de Dios	Women	<60	Aware treated	74.7	70.0	79.4
2020	Madre de Dios	Women	60+	Healthy	63.8	60.5	67.1
2020	Madre de Dios	Women	60+	Unaware	70.9	65.5	76.2
2020	Madre de Dios	Women	60+	Aware not treated	66.0	62.6	69.5
2020	Madre de Dios	Women	60+	Aware treated	76.0	76.0	76.0
2015	Moquegua	Men	<60	Healthy	73.4	72.3	74.4
2015	Moquegua	Men	<60	Unaware	87.6	84.4	90.9

2015	Moquegua	Men	<60	Aware not treated	81.1	76.7	85.6
2015	Moquegua	Men	<60	Aware treated	81.6	76.3	86.9
2015	Moquegua	Men	60+	Healthy	70.1	67.3	72.8
2015	Moquegua	Men	60+	Unaware	78.3	72.9	83.8
2015	Moquegua	Men	60+	Aware not treated	75.6	67.7	83.5
2015	Moquegua	Men	60+	Aware treated	78.6	71.0	86.2
2016	Moquegua	Men	<60	Healthy	73.4	72.4	74.5
2016	Moquegua	Men	<60	Unaware	89.9	86.4	93.5
2016	Moquegua	Men	<60	Aware not treated	80.6	76.2	85.1
2016	Moquegua	Men	<60	Aware treated	79.9	69.1	90.7
2016	Moquegua	Men	60+	Healthy	72.6	70.2	75.0
2016	Moquegua	Men	60+	Unaware	83.4	81.2	85.6
2016	Moquegua	Men	60+	Aware not treated	73.6	60.5	86.7
2016	Moquegua	Men	60+	Aware treated	73.3	65.4	81.1
2017	Moquegua	Men	<60	Healthy	73.3	72.3	74.4
2017	Moquegua	Men	<60	Unaware	87.5	85.2	89.9
2017	Moquegua	Men	<60	Aware not treated	98.9	80.0	117.7
2017	Moquegua	Men	<60	Aware treated	84.0	78.7	89.3
2017	Moquegua	Men	60+	Healthy	69.9	67.2	72.5
2017	Moquegua	Men	60+	Unaware	84.2	81.0	87.4
2017	Moquegua	Men	60+	Aware not treated	82.6	78.4	86.9
2017	Moquegua	Men	60+	Aware treated	74.5	69.3	79.7
2018	Moquegua	Men	<60	Healthy	74.1	73.0	75.2
2018	Moquegua	Men	<60	Unaware	87.4	84.1	90.7
2018	Moquegua	Men	<60	Aware not treated	86.3	80.7	92.0
2018	Moquegua	Men	<60	Aware treated	84.9	78.6	91.2
2018	Moquegua	Men	60+	Healthy	68.7	66.3	71.0
2018	Moquegua	Men	60+	Unaware	82.6	77.2	88.0
2018	Moquegua	Men	60+	Aware not treated	73.8	64.8	82.9

2018	Moquegua	Men	60+	Aware treated	75.1	70.2	80.0
2019	Moquegua	Men	<60	Healthy	73.9	72.9	75.0
2019	Moquegua	Men	<60	Unaware	86.2	83.4	88.9
2019	Moquegua	Men	<60	Aware not treated	83.9	81.2	86.7
2019	Moquegua	Men	<60	Aware treated	81.4	73.9	88.9
2019	Moquegua	Men	60+	Healthy	69.1	66.8	71.3
2019	Moquegua	Men	60+	Unaware	76.6	73.6	79.6
2019	Moquegua	Men	60+	Aware not treated	73.5	70.2	76.8
2019	Moquegua	Men	60+	Aware treated	74.3	66.9	81.7
2020	Moquegua	Men	<60	Healthy	74.3	72.7	75.8
2020	Moquegua	Men	<60	Unaware	91.4	88.9	93.8
2020	Moquegua	Men	<60	Aware not treated	81.9	77.7	86.2
2020	Moquegua	Men	<60	Aware treated	79.4	69.3	89.5
2020	Moquegua	Men	60+	Healthy	69.8	67.5	72.1
2020	Moquegua	Men	60+	Unaware	82.4	78.1	86.7
2020	Moquegua	Men	60+	Aware not treated	87.4	82.6	92.2
2020	Moquegua	Men	60+	Aware treated	80.7	73.2	88.2
2015	Moquegua	Women	<60	Healthy	68.7	67.5	69.9
2015	Moquegua	Women	<60	Unaware	81.3	75.1	87.6
2015	Moquegua	Women	<60	Aware not treated	66.8	60.7	72.8
2015	Moquegua	Women	<60	Aware treated	79.4	73.6	85.1
2015	Moquegua	Women	60+	Healthy	64.7	62.1	67.2
2015	Moquegua	Women	60+	Unaware	80.4	73.7	87.1
2015	Moquegua	Women	60+	Aware not treated	62.7	50.9	74.6
2015	Moquegua	Women	60+	Aware treated	65.0	61.5	68.6
2016	Moquegua	Women	<60	Healthy	68.5	67.5	69.6
2016	Moquegua	Women	<60	Unaware	78.7	68.9	88.5
2016	Moquegua	Women	<60	Aware not treated	70.9	65.5	76.3
2016	Moquegua	Women	<60	Aware treated	75.3	69.4	81.3

2016	Moquegua	Women	60+	Healthy	65.7	63.2	68.2
2016	Moquegua	Women	60+	Unaware	77.9	71.7	84.1
2016	Moquegua	Women	60+	Aware not treated	82.4	58.3	106.5
2016	Moquegua	Women	60+	Aware treated	67.3	63.3	71.2
2017	Moquegua	Women	<60	Healthy	67.5	66.5	68.4
2017	Moquegua	Women	<60	Unaware	87.6	85.4	89.7
2017	Moquegua	Women	<60	Aware not treated	73.0	67.8	78.2
2017	Moquegua	Women	<60	Aware treated	76.8	72.0	81.7
2017	Moquegua	Women	60+	Healthy	65.1	62.0	68.2
2017	Moquegua	Women	60+	Unaware	74.5	72.5	76.5
2017	Moquegua	Women	60+	Aware not treated	70.5	63.2	77.8
2017	Moquegua	Women	60+	Aware treated	70.6	64.8	76.5
2018	Moquegua	Women	<60	Healthy	69.7	68.6	70.8
2018	Moquegua	Women	<60	Unaware	91.1	84.5	97.6
2018	Moquegua	Women	<60	Aware not treated	76.8	73.1	80.4
2018	Moquegua	Women	<60	Aware treated	83.7	77.5	89.8
2018	Moquegua	Women	60+	Healthy	68.0	64.5	71.5
2018	Moquegua	Women	60+	Unaware	79.2	72.3	86.2
2018	Moquegua	Women	60+	Aware not treated	67.2	64.7	69.6
2018	Moquegua	Women	60+	Aware treated	63.4	56.3	70.4
2019	Moquegua	Women	<60	Healthy	68.7	67.8	69.6
2019	Moquegua	Women	<60	Unaware	81.1	76.3	85.9
2019	Moquegua	Women	<60	Aware not treated	69.2	66.1	72.3
2019	Moquegua	Women	<60	Aware treated	73.9	68.6	79.2
2019	Moquegua	Women	60+	Healthy	65.4	62.1	68.7
2019	Moquegua	Women	60+	Unaware	79.2	74.1	84.3
2019	Moquegua	Women	60+	Aware not treated	68.6	62.6	74.6
2019	Moquegua	Women	60+	Aware treated	69.4	66.5	72.4
2020	Moquegua	Women	<60	Healthy	70.0	68.9	71.1

2020	Moquegua	Women	<60	Unaware	83.9	80.8	87.0
2020	Moquegua	Women	<60	Aware not treated	76.2	67.2	85.3
2020	Moquegua	Women	<60	Aware treated	83.8	74.4	93.1
2020	Moquegua	Women	60+	Healthy	65.9	63.0	68.8
2020	Moquegua	Women	60+	Unaware	75.2	72.9	77.5
2020	Moquegua	Women	60+	Aware not treated	61.8	49.0	74.6
2020	Moquegua	Women	60+	Aware treated	68.0	59.2	76.8
2015	Pasco	Men	<60	Healthy	71.0	69.7	72.3
2015	Pasco	Men	<60	Unaware	86.8	83.9	89.6
2015	Pasco	Men	<60	Aware not treated	78.2	73.9	82.6
2015	Pasco	Men	<60	Aware treated	94.9	86.5	103.3
2015	Pasco	Men	60+	Healthy	71.4	68.4	74.4
2015	Pasco	Men	60+	Unaware	77.7	71.4	84.0
2015	Pasco	Men	60+	Aware not treated	82.4	67.0	97.9
2015	Pasco	Men	60+	Aware treated	81.2	77.4	85.0
2016	Pasco	Men	<60	Healthy	71.9	70.6	73.2
2016	Pasco	Men	<60	Unaware	88.8	86.6	90.9
2016	Pasco	Men	<60	Aware not treated	77.9	72.8	83.0
2016	Pasco	Men	<60	Aware treated	72.9	62.4	83.4
2016	Pasco	Men	60+	Healthy	72.3	69.4	75.2
2016	Pasco	Men	60+	Unaware	77.9	73.7	82.1
2016	Pasco	Men	60+	Aware not treated	69.0	64.4	73.6
2016	Pasco	Men	60+	Aware treated	76.0	67.2	84.9
2017	Pasco	Men	<60	Healthy	72.6	71.4	73.8
2017	Pasco	Men	<60	Unaware	87.6	85.1	90.1
2017	Pasco	Men	<60	Aware not treated	73.7	68.2	79.1
2017	Pasco	Men	<60	Aware treated	86.1	82.3	89.9
2017	Pasco	Men	60+	Healthy	70.3	67.1	73.5
2017	Pasco	Men	60+	Unaware	81.5	76.9	86.0

2017	Pasco	Men	60+	Aware not treated	76.8	71.1	82.5
2017	Pasco	Men	60+	Aware treated	76.4	69.4	83.3
2018	Pasco	Men	<60	Healthy	74.8	73.6	76.1
2018	Pasco	Men	<60	Unaware	90.9	88.7	93.1
2018	Pasco	Men	<60	Aware not treated	78.7	76.9	80.5
2018	Pasco	Men	<60	Aware treated	80.8	64.9	96.7
2018	Pasco	Men	60+	Healthy	71.1	68.6	73.5
2018	Pasco	Men	60+	Unaware	89.4	85.7	93.2
2018	Pasco	Men	60+	Aware not treated	79.1	77.8	80.3
2018	Pasco	Men	60+	Aware treated	81.2	70.0	92.5
2019	Pasco	Men	<60	Healthy	74.7	73.8	75.6
2019	Pasco	Men	<60	Unaware	93.3	89.4	97.2
2019	Pasco	Men	<60	Aware not treated	73.8	70.5	77.0
2019	Pasco	Men	<60	Aware treated	87.5	73.2	101.8
2019	Pasco	Men	60+	Healthy	69.9	67.1	72.7
2019	Pasco	Men	60+	Unaware	80.8	74.8	86.8
2019	Pasco	Men	60+	Aware not treated	93.0	93.0	93.0
2019	Pasco	Men	60+	Aware treated	81.5	67.1	95.9
2020	Pasco	Men	<60	Healthy	71.3	69.7	72.9
2020	Pasco	Men	<60	Unaware	92.6	87.5	97.7
2020	Pasco	Men	<60	Aware not treated	75.2	72.4	78.0
2020	Pasco	Men	<60	Aware treated	95.0	95.0	95.0
2020	Pasco	Men	60+	Healthy	73.2	69.9	76.5
2020	Pasco	Men	60+	Unaware	83.2	78.9	87.6
2020	Pasco	Men	60+	Aware not treated	87.0	78.3	95.7
2020	Pasco	Men	60+	Aware treated	76.1	50.2	101.9
2015	Pasco	Women	<60	Healthy	68.2	67.0	69.5
2015	Pasco	Women	<60	Unaware	82.9	79.0	86.9
2015	Pasco	Women	<60	Aware not treated	72.0	66.9	77.0

2015	Pasco	Women	<60	Aware treated	78.9	70.4	87.5
2015	Pasco	Women	60+	Healthy	64.7	62.4	67.0
2015	Pasco	Women	60+	Unaware	78.4	75.3	81.4
2015	Pasco	Women	60+	Aware not treated	71.4	65.2	77.6
2015	Pasco	Women	60+	Aware treated	78.6	62.2	95.0
2016	Pasco	Women	<60	Healthy	68.9	67.6	70.1
2016	Pasco	Women	<60	Unaware	86.0	79.6	92.3
2016	Pasco	Women	<60	Aware not treated	70.7	67.0	74.5
2016	Pasco	Women	<60	Aware treated	74.2	64.6	83.7
2016	Pasco	Women	60+	Healthy	65.7	63.7	67.7
2016	Pasco	Women	60+	Unaware	77.1	70.6	83.6
2016	Pasco	Women	60+	Aware not treated	74.9	67.9	81.9
2016	Pasco	Women	60+	Aware treated	70.8	65.7	75.9
2017	Pasco	Women	<60	Healthy	69.0	67.7	70.3
2017	Pasco	Women	<60	Unaware	83.7	78.8	88.5
2017	Pasco	Women	<60	Aware not treated	71.3	68.3	74.3
2017	Pasco	Women	<60	Aware treated	77.0	68.5	85.5
2017	Pasco	Women	60+	Healthy	68.6	65.9	71.2
2017	Pasco	Women	60+	Unaware	76.5	71.9	81.1
2017	Pasco	Women	60+	Aware not treated	66.6	59.7	73.4
2017	Pasco	Women	60+	Aware treated	74.3	64.4	84.3
2018	Pasco	Women	<60	Healthy	71.5	70.3	72.6
2018	Pasco	Women	<60	Unaware	90.4	84.8	96.0
2018	Pasco	Women	<60	Aware not treated	73.1	68.2	78.0
2018	Pasco	Women	<60	Aware treated	76.0	67.7	84.2
2018	Pasco	Women	60+	Healthy	70.1	67.5	72.8
2018	Pasco	Women	60+	Unaware	84.2	80.4	88.0
2018	Pasco	Women	60+	Aware not treated	80.7	71.4	90.0
2018	Pasco	Women	60+	Aware treated	74.2	69.1	79.4

2019	Pasco	Women	<60	Healthy	70.4	69.4	71.4
2019	Pasco	Women	<60	Unaware	91.1	86.4	95.8
2019	Pasco	Women	<60	Aware not treated	68.3	62.2	74.4
2019	Pasco	Women	<60	Aware treated	77.0	68.6	85.5
2019	Pasco	Women	60+	Healthy	68.0	65.8	70.2
2019	Pasco	Women	60+	Unaware	75.4	72.8	78.0
2019	Pasco	Women	60+	Aware not treated	75.0	66.3	83.7
2019	Pasco	Women	60+	Aware treated	67.8	59.7	75.9
2020	Pasco	Women	<60	Healthy	70.2	69.2	71.2
2020	Pasco	Women	<60	Unaware	91.2	84.8	97.6
2020	Pasco	Women	<60	Aware not treated	63.8	59.6	67.9
2020	Pasco	Women	<60	Aware treated	84.4	78.6	90.2
2020	Pasco	Women	60+	Healthy	68.8	65.5	72.1
2020	Pasco	Women	60+	Unaware	87.4	79.9	94.9
2020	Pasco	Women	60+	Aware not treated	71.7	59.5	83.9
2020	Pasco	Women	60+	Aware treated	77.0	60.5	93.5
2015	Ancash	Men	<60	Healthy	71.5	70.5	72.5
2015	Ancash	Men	<60	Unaware	86.4	83.9	88.9
2015	Ancash	Men	<60	Aware not treated	77.1	73.3	81.0
2015	Ancash	Men	<60	Aware treated	75.3	69.3	81.3
2015	Ancash	Men	60+	Healthy	67.0	64.4	69.7
2015	Ancash	Men	60+	Unaware	77.8	75.3	80.3
2015	Ancash	Men	60+	Aware not treated	83.7	75.5	91.9
2015	Ancash	Men	60+	Aware treated	72.1	63.2	81.0
2016	Ancash	Men	<60	Healthy	72.3	71.0	73.5
2016	Ancash	Men	<60	Unaware	87.3	83.3	91.2
2016	Ancash	Men	<60	Aware not treated	80.1	74.9	85.4
2016	Ancash	Men	<60	Aware treated	81.7	69.9	93.6
2016	Ancash	Men	60+	Healthy	68.3	66.0	70.6

2016	Ancash	Men	60+	Unaware	79.7	74.6	84.8
2016	Ancash	Men	60+	Aware not treated	74.1	71.3	76.9
2016	Ancash	Men	60+	Aware treated	75.7	65.2	86.1
2017	Ancash	Men	<60	Healthy	73.1	72.1	74.1
2017	Ancash	Men	<60	Unaware	88.4	84.8	92.0
2017	Ancash	Men	<60	Aware not treated	83.0	74.1	92.0
2017	Ancash	Men	<60	Aware treated	84.5	79.8	89.3
2017	Ancash	Men	60+	Healthy	69.4	67.2	71.6
2017	Ancash	Men	60+	Unaware	79.9	77.1	82.8
2017	Ancash	Men	60+	Aware not treated	77.4	63.9	90.9
2017	Ancash	Men	60+	Aware treated	71.3	68.0	74.6
2018	Ancash	Men	<60	Healthy	72.6	71.7	73.5
2018	Ancash	Men	<60	Unaware	88.5	84.8	92.3
2018	Ancash	Men	<60	Aware not treated	84.0	84.0	84.0
2018	Ancash	Men	<60	Aware treated	82.4	72.3	92.5
2018	Ancash	Men	60+	Healthy	68.3	65.6	71.0
2018	Ancash	Men	60+	Unaware	78.1	74.6	81.7
2018	Ancash	Men	60+	Aware not treated	79.3	71.3	87.4
2018	Ancash	Men	60+	Aware treated	73.3	64.6	81.9
2019	Ancash	Men	<60	Healthy	73.6	72.5	74.8
2019	Ancash	Men	<60	Unaware	90.0	86.7	93.3
2019	Ancash	Men	<60	Aware not treated	85.0	66.9	103.1
2019	Ancash	Men	<60	Aware treated	80.6	71.1	90.2
2019	Ancash	Men	60+	Healthy	70.9	68.5	73.4
2019	Ancash	Men	60+	Unaware	78.9	75.3	82.5
2019	Ancash	Men	60+	Aware not treated	77.0	77.0	77.0
2019	Ancash	Men	60+	Aware treated	75.9	72.0	79.8
2020	Ancash	Men	<60	Healthy	73.7	72.1	75.3
2020	Ancash	Men	<60	Unaware	89.5	87.4	91.7

2020	Ancash	Men	<60	Aware not treated	65.0	65.0	65.0
2020	Ancash	Men	<60	Aware treated	81.0	81.0	81.0
2020	Ancash	Men	60+	Healthy	71.5	69.0	73.9
2020	Ancash	Men	60+	Unaware	82.3	76.1	88.5
2020	Ancash	Men	60+	Aware not treated	77.0	77.0	77.0
2020	Ancash	Men	60+	Aware treated	69.8	63.9	75.6
2015	Ancash	Women	<60	Healthy	68.0	67.0	69.0
2015	Ancash	Women	<60	Unaware	92.2	84.9	99.4
2015	Ancash	Women	<60	Aware not treated	70.5	59.5	81.5
2015	Ancash	Women	<60	Aware treated	73.1	69.8	76.3
2015	Ancash	Women	60+	Healthy	64.6	62.1	67.2
2015	Ancash	Women	60+	Unaware	71.7	66.3	77.2
2015	Ancash	Women	60+	Aware not treated	67.3	63.3	71.3
2015	Ancash	Women	60+	Aware treated	73.0	68.5	77.4
2016	Ancash	Women	<60	Healthy	69.3	68.1	70.4
2016	Ancash	Women	<60	Unaware	85.8	81.8	89.9
2016	Ancash	Women	<60	Aware not treated	77.4	67.4	87.4
2016	Ancash	Women	<60	Aware treated	79.6	74.1	85.0
2016	Ancash	Women	60+	Healthy	66.6	64.7	68.5
2016	Ancash	Women	60+	Unaware	73.6	70.0	77.2
2016	Ancash	Women	60+	Aware not treated	67.9	59.9	75.9
2016	Ancash	Women	60+	Aware treated	76.0	69.4	82.6
2017	Ancash	Women	<60	Healthy	69.3	68.3	70.3
2017	Ancash	Women	<60	Unaware	84.8	80.2	89.4
2017	Ancash	Women	<60	Aware not treated	81.2	72.1	90.3
2017	Ancash	Women	<60	Aware treated	72.1	65.3	78.9
2017	Ancash	Women	60+	Healthy	65.0	63.1	67.0
2017	Ancash	Women	60+	Unaware	73.1	70.3	75.8
2017	Ancash	Women	60+	Aware not treated	66.7	52.7	80.7

2017	Ancash	Women	60+	Aware treated	71.9	66.7	77.0
2018	Ancash	Women	<60	Healthy	67.7	66.8	68.6
2018	Ancash	Women	<60	Unaware	86.8	83.8	89.9
2018	Ancash	Women	<60	Aware not treated	70.7	67.6	73.9
2018	Ancash	Women	<60	Aware treated	70.8	65.8	75.7
2018	Ancash	Women	60+	Healthy	63.3	60.7	65.8
2018	Ancash	Women	60+	Unaware	75.9	72.1	79.7
2018	Ancash	Women	60+	Aware not treated	66.4	65.8	66.9
2018	Ancash	Women	60+	Aware treated	72.3	68.8	75.7
2019	Ancash	Women	<60	Healthy	68.3	67.3	69.2
2019	Ancash	Women	<60	Unaware	84.1	80.4	87.9
2019	Ancash	Women	<60	Aware not treated	83.8	57.3	110.4
2019	Ancash	Women	<60	Aware treated	78.6	72.3	85.0
2019	Ancash	Women	60+	Healthy	66.1	63.5	68.6
2019	Ancash	Women	60+	Unaware	76.0	73.3	78.6
2019	Ancash	Women	60+	Aware not treated	68.6	58.4	78.9
2019	Ancash	Women	60+	Aware treated	73.5	69.4	77.6
2020	Ancash	Women	<60	Healthy	69.4	67.9	71.0
2020	Ancash	Women	<60	Unaware	90.0	86.7	93.2
2020	Ancash	Women	<60	Aware not treated	71.7	57.3	86.0
2020	Ancash	Women	<60	Aware treated	85.0	81.5	88.5
2020	Ancash	Women	60+	Healthy	64.4	62.3	66.4
2020	Ancash	Women	60+	Unaware	75.3	69.9	80.7
2020	Ancash	Women	60+	Aware not treated	67.0	67.0	67.0
2020	Ancash	Women	60+	Aware treated	69.6	63.7	75.5
2015	Piura	Men	<60	Healthy	72.5	71.2	73.8
2015	Piura	Men	<60	Unaware	89.0	84.2	93.8
2015	Piura	Men	<60	Aware not treated	92.7	79.1	106.2
2015	Piura	Men	<60	Aware treated	85.7	78.7	92.6

2015	Piura	Men	60+	Healthy	68.4	64.2	72.6
2015	Piura	Men	60+	Unaware	84.6	80.7	88.4
2015	Piura	Men	60+	Aware not treated	80.0	80.0	80.0
2015	Piura	Men	60+	Aware treated	72.8	67.0	78.6
2016	Piura	Men	<60	Healthy	73.0	71.9	74.1
2016	Piura	Men	<60	Unaware	89.5	86.4	92.6
2016	Piura	Men	<60	Aware not treated	69.5	68.4	70.6
2016	Piura	Men	<60	Aware treated	85.2	77.9	92.6
2016	Piura	Men	60+	Healthy	69.0	66.2	71.9
2016	Piura	Men	60+	Unaware	75.3	67.9	82.6
2016	Piura	Men	60+	Aware not treated	79.0	79.0	79.0
2016	Piura	Men	60+	Aware treated	74.5	69.8	79.2
2017	Piura	Men	<60	Healthy	72.4	71.3	73.5
2017	Piura	Men	<60	Unaware	85.8	82.9	88.8
2017	Piura	Men	<60	Aware not treated	65.0	65.0	65.0
2017	Piura	Men	<60	Aware treated	84.8	74.7	95.0
2017	Piura	Men	60+	Healthy	66.2	63.3	69.1
2017	Piura	Men	60+	Unaware	80.4	76.6	84.3
2017	Piura	Men	60+	Aware not treated	77.6	69.9	85.3
2017	Piura	Men	60+	Aware treated	69.1	63.5	74.8
2018	Piura	Men	<60	Healthy	72.7	71.6	73.9
2018	Piura	Men	<60	Unaware	88.5	86.3	90.8
2018	Piura	Men	<60	Aware not treated	74.1	71.0	77.3
2018	Piura	Men	<60	Aware treated	82.9	73.9	92.0
2018	Piura	Men	60+	Healthy	69.8	67.5	72.1
2018	Piura	Men	60+	Unaware	80.0	74.4	85.7
2018	Piura	Men	60+	Aware not treated	77.0	70.3	83.7
2018	Piura	Men	60+	Aware treated	73.8	67.2	80.3
2019	Piura	Men	<60	Healthy	72.7	71.7	73.8

2019	Piura	Men	<60	Unaware	89.4	86.9	92.0
2019	Piura	Men	<60	Aware not treated	88.1	78.9	97.4
2019	Piura	Men	<60	Aware treated	86.6	74.6	98.7
2019	Piura	Men	60+	Healthy	68.8	65.9	71.8
2019	Piura	Men	60+	Unaware	78.7	74.6	82.8
2019	Piura	Men	60+	Aware not treated	77.4	71.0	83.8
2019	Piura	Men	60+	Aware treated	71.2	62.6	79.8
2020	Piura	Men	<60	Healthy	72.6	71.1	74.0
2020	Piura	Men	<60	Unaware	89.6	86.6	92.6
2020	Piura	Men	<60	Aware not treated	86.0	86.0	86.0
2020	Piura	Men	<60	Aware treated	78.7	72.0	85.4
2020	Piura	Men	60+	Healthy	69.9	67.6	72.2
2020	Piura	Men	60+	Unaware	80.1	75.0	85.3
2020	Piura	Men	60+	Aware treated	72.1	67.2	77.1
2015	Piura	Women	<60	Healthy	68.9	67.9	69.8
2015	Piura	Women	<60	Unaware	86.7	83.4	90.0
2015	Piura	Women	<60	Aware not treated	72.9	70.4	75.3
2015	Piura	Women	<60	Aware treated	86.1	80.5	91.7
2015	Piura	Women	60+	Healthy	64.0	60.8	67.2
2015	Piura	Women	60+	Unaware	75.3	67.3	83.3
2015	Piura	Women	60+	Aware not treated	70.7	60.6	80.8
2015	Piura	Women	60+	Aware treated	74.5	70.3	78.8
2016	Piura	Women	<60	Healthy	68.2	67.1	69.2
2016	Piura	Women	<60	Unaware	87.9	85.2	90.6
2016	Piura	Women	<60	Aware not treated	77.8	73.1	82.5
2016	Piura	Women	<60	Aware treated	79.6	75.9	83.3
2016	Piura	Women	60+	Healthy	66.0	63.8	68.3
2016	Piura	Women	60+	Unaware	76.8	72.5	81.1
2016	Piura	Women	60+	Aware not treated	74.2	63.2	85.3

2016	Piura	Women	60+	Aware treated	70.4	65.2	75.6
2017	Piura	Women	<60	Healthy	68.6	67.7	69.6
2017	Piura	Women	<60	Unaware	87.8	84.3	91.2
2017	Piura	Women	<60	Aware not treated	70.2	66.6	73.8
2017	Piura	Women	<60	Aware treated	76.5	70.3	82.7
2017	Piura	Women	60+	Healthy	65.0	62.9	67.1
2017	Piura	Women	60+	Unaware	77.7	72.8	82.6
2017	Piura	Women	60+	Aware not treated	77.0	61.3	92.7
2017	Piura	Women	60+	Aware treated	71.5	66.6	76.3
2018	Piura	Women	<60	Healthy	69.2	68.1	70.3
2018	Piura	Women	<60	Unaware	86.0	81.6	90.5
2018	Piura	Women	<60	Aware not treated	71.7	65.8	77.6
2018	Piura	Women	<60	Aware treated	76.6	71.9	81.3
2018	Piura	Women	60+	Healthy	67.3	64.3	70.3
2018	Piura	Women	60+	Unaware	70.6	65.6	75.7
2018	Piura	Women	60+	Aware not treated	70.5	58.1	83.0
2018	Piura	Women	60+	Aware treated	68.2	62.9	73.5
2019	Piura	Women	<60	Healthy	69.5	68.4	70.5
2019	Piura	Women	<60	Unaware	86.6	83.9	89.4
2019	Piura	Women	<60	Aware not treated	75.1	71.0	79.1
2019	Piura	Women	<60	Aware treated	80.6	74.9	86.4
2019	Piura	Women	60+	Healthy	66.0	63.8	68.1
2019	Piura	Women	60+	Unaware	78.1	72.1	84.0
2019	Piura	Women	60+	Aware not treated	68.2	64.1	72.3
2019	Piura	Women	60+	Aware treated	66.9	63.9	69.9
2020	Piura	Women	<60	Healthy	69.9	68.7	71.1
2020	Piura	Women	<60	Unaware	89.9	85.7	94.2
2020	Piura	Women	<60	Aware not treated	73.4	66.1	80.7
2020	Piura	Women	<60	Aware treated	78.1	72.9	83.2

2020	Piura	Women	60+	Healthy	63.7	59.8	67.6
2020	Piura	Women	60+	Unaware	73.3	69.2	77.4
2020	Piura	Women	60+	Aware not treated	72.0	66.2	77.8
2020	Piura	Women	60+	Aware treated	69.3	64.2	74.4
2015	Puno	Men	<60	Healthy	72.2	70.7	73.7
2015	Puno	Men	<60	Unaware	94.0	89.2	98.7
2015	Puno	Men	<60	Aware not treated	79.0	68.8	89.1
2015	Puno	Men	<60	Aware treated	76.0	76.0	76.0
2015	Puno	Men	60+	Healthy	68.1	65.6	70.5
2015	Puno	Men	60+	Unaware	80.7	76.5	85.0
2015	Puno	Men	60+	Aware not treated	72.1	67.5	76.7
2015	Puno	Men	60+	Aware treated	74.4	67.2	81.6
2016	Puno	Men	<60	Healthy	72.9	71.4	74.3
2016	Puno	Men	<60	Unaware	89.3	85.9	92.8
2016	Puno	Men	<60	Aware not treated	82.4	77.9	86.9
2016	Puno	Men	<60	Aware treated	104.0	84.8	123.2
2016	Puno	Men	60+	Healthy	72.0	68.6	75.5
2016	Puno	Men	60+	Unaware	79.0	74.9	83.1
2016	Puno	Men	60+	Aware not treated	65.3	60.5	70.1
2016	Puno	Men	60+	Aware treated	74.8	53.4	96.2
2017	Puno	Men	<60	Healthy	74.3	73.1	75.5
2017	Puno	Men	<60	Unaware	93.0	89.8	96.2
2017	Puno	Men	<60	Aware not treated	77.8	73.8	81.8
2017	Puno	Men	<60	Aware treated	90.3	79.3	101.4
2017	Puno	Men	60+	Healthy	70.3	68.2	72.4
2017	Puno	Men	60+	Unaware	81.8	77.8	85.7
2017	Puno	Men	60+	Aware not treated	70.3	66.1	74.6
2017	Puno	Men	60+	Aware treated	79.1	69.2	89.0
2018	Puno	Men	<60	Healthy	75.3	74.1	76.5

2018	Puno	Men	<60	Unaware	91.3	88.7	93.9
2018	Puno	Men	<60	Aware not treated	72.3	66.4	78.1
2018	Puno	Men	<60	Aware treated	74.9	69.6	80.2
2018	Puno	Men	60+	Healthy	72.1	69.8	74.5
2018	Puno	Men	60+	Unaware	83.7	79.6	87.8
2018	Puno	Men	60+	Aware not treated	75.8	70.3	81.3
2018	Puno	Men	60+	Aware treated	80.1	72.9	87.3
2019	Puno	Men	<60	Healthy	73.7	72.6	74.9
2019	Puno	Men	<60	Unaware	90.2	87.2	93.2
2019	Puno	Men	<60	Aware not treated	77.0	72.6	81.4
2019	Puno	Men	<60	Aware treated	77.1	72.5	81.8
2019	Puno	Men	60+	Healthy	71.5	69.0	73.9
2019	Puno	Men	60+	Unaware	82.7	79.8	85.5
2019	Puno	Men	60+	Aware not treated	72.3	64.2	80.3
2019	Puno	Men	60+	Aware treated	77.7	66.7	88.8
2020	Puno	Men	<60	Healthy	73.8	72.4	75.3
2020	Puno	Men	<60	Unaware	89.1	84.6	93.6
2020	Puno	Men	<60	Aware not treated	81.8	74.8	88.8
2020	Puno	Men	<60	Aware treated	76.5	69.0	83.9
2020	Puno	Men	60+	Healthy	70.8	69.2	72.4
2020	Puno	Men	60+	Unaware	82.3	78.8	85.8
2020	Puno	Men	60+	Aware not treated	84.9	63.0	106.8
2020	Puno	Men	60+	Aware treated	74.0	63.4	84.7
2015	Puno	Women	<60	Healthy	69.4	68.4	70.4
2015	Puno	Women	<60	Unaware	88.1	80.3	96.0
2015	Puno	Women	<60	Aware not treated	74.3	69.6	78.9
2015	Puno	Women	<60	Aware treated	78.6	73.9	83.2
2015	Puno	Women	60+	Healthy	65.0	63.1	66.8
2015	Puno	Women	60+	Unaware	76.3	73.2	79.4

2015	Puno	Women	60+	Aware not treated	79.9	72.8	87.0
2015	Puno	Women	60+	Aware treated	73.3	69.0	77.6
2016	Puno	Women	<60	Healthy	69.0	67.9	70.1
2016	Puno	Women	<60	Unaware	85.7	80.2	91.2
2016	Puno	Women	<60	Aware not treated	74.7	71.2	78.2
2016	Puno	Women	<60	Aware treated	80.3	74.1	86.5
2016	Puno	Women	60+	Healthy	66.0	64.1	67.9
2016	Puno	Women	60+	Unaware	76.1	72.4	79.7
2016	Puno	Women	60+	Aware not treated	68.7	64.5	72.9
2016	Puno	Women	60+	Aware treated	72.1	64.0	80.1
2017	Puno	Women	<60	Healthy	70.0	69.0	71.1
2017	Puno	Women	<60	Unaware	87.7	82.2	93.3
2017	Puno	Women	<60	Aware not treated	71.8	67.7	75.8
2017	Puno	Women	<60	Aware treated	77.6	67.2	88.0
2017	Puno	Women	60+	Healthy	65.8	64.1	67.5
2017	Puno	Women	60+	Unaware	77.7	72.2	83.2
2017	Puno	Women	60+	Aware not treated	74.7	66.9	82.4
2017	Puno	Women	60+	Aware treated	72.1	68.1	76.2
2018	Puno	Women	<60	Healthy	71.1	69.9	72.3
2018	Puno	Women	<60	Unaware	89.9	80.1	99.8
2018	Puno	Women	<60	Aware not treated	75.5	70.9	80.1
2018	Puno	Women	<60	Aware treated	79.6	68.0	91.2
2018	Puno	Women	60+	Healthy	68.2	65.7	70.6
2018	Puno	Women	60+	Unaware	80.3	74.2	86.5
2018	Puno	Women	60+	Aware not treated	72.7	68.1	77.3
2018	Puno	Women	60+	Aware treated	79.4	75.7	83.1
2019	Puno	Women	<60	Healthy	69.9	69.0	70.8
2019	Puno	Women	<60	Unaware	89.0	77.4	100.6
2019	Puno	Women	<60	Aware not treated	79.0	72.6	85.5

2019	Puno	Women	<60	Aware treated	78.8	73.4	84.1
2019	Puno	Women	60+	Healthy	66.9	65.4	68.5
2019	Puno	Women	60+	Unaware	77.5	74.1	80.9
2019	Puno	Women	60+	Aware not treated	81.1	73.3	89.0
2019	Puno	Women	60+	Aware treated	73.0	70.0	76.0
2020	Puno	Women	<60	Healthy	71.7	70.5	72.9
2020	Puno	Women	<60	Unaware	90.4	82.0	98.8
2020	Puno	Women	<60	Aware not treated	77.8	70.2	85.5
2020	Puno	Women	<60	Aware treated	87.4	71.9	102.9
2020	Puno	Women	60+	Healthy	67.9	66.2	69.7
2020	Puno	Women	60+	Unaware	82.2	75.6	88.9
2020	Puno	Women	60+	Aware not treated	77.2	71.9	82.5
2020	Puno	Women	60+	Aware treated	76.1	66.8	85.4
2015	San Martin	Men	<60	Healthy	69.7	68.7	70.6
2015	San Martin	Men	<60	Unaware	82.2	78.7	85.7
2015	San Martin	Men	<60	Aware not treated	72.5	65.3	79.7
2015	San Martin	Men	<60	Aware treated	76.3	70.2	82.4
2015	San Martin	Men	60+	Healthy	66.1	64.1	68.1
2015	San Martin	Men	60+	Unaware	79.6	75.0	84.1
2015	San Martin	Men	60+	Aware not treated	71.4	66.1	76.7
2015	San Martin	Men	60+	Aware treated	73.7	68.6	78.8
2016	San Martin	Men	<60	Healthy	69.9	68.9	70.9
2016	San Martin	Men	<60	Unaware	90.0	84.9	95.2
2016	San Martin	Men	<60	Aware not treated	76.2	71.4	81.1
2016	San Martin	Men	<60	Aware treated	79.2	72.5	85.8
2016	San Martin	Men	60+	Healthy	63.3	60.7	66.0
2016	San Martin	Men	60+	Unaware	77.5	73.2	81.7
2016	San Martin	Men	60+	Aware treated	78.4	71.1	85.7
2017	San Martin	Men	<60	Healthy	70.3	69.1	71.4

2017	San Martin	Men	<60	Unaware	86.1	83.4	88.8
2017	San Martin	Men	<60	Aware not treated	71.3	65.8	76.8
2017	San Martin	Men	<60	Aware treated	83.5	75.1	91.8
2017	San Martin	Men	60+	Healthy	66.6	64.4	68.9
2017	San Martin	Men	60+	Unaware	76.5	73.4	79.6
2017	San Martin	Men	60+	Aware not treated	86.7	81.9	91.5
2017	San Martin	Men	60+	Aware treated	73.8	66.2	81.4
2018	San Martin	Men	<60	Healthy	73.3	72.3	74.4
2018	San Martin	Men	<60	Unaware	91.5	88.5	94.5
2018	San Martin	Men	<60	Aware not treated	83.8	74.0	93.7
2018	San Martin	Men	<60	Aware treated	80.0	73.1	87.0
2018	San Martin	Men	60+	Healthy	66.4	63.3	69.5
2018	San Martin	Men	60+	Unaware	82.7	78.8	86.5
2018	San Martin	Men	60+	Aware not treated	79.5	64.4	94.6
2018	San Martin	Men	60+	Aware treated	83.9	79.9	87.8
2019	San Martin	Men	<60	Healthy	71.1	70.0	72.1
2019	San Martin	Men	<60	Unaware	87.0	83.9	90.2
2019	San Martin	Men	<60	Aware not treated	79.0	73.6	84.4
2019	San Martin	Men	<60	Aware treated	86.7	80.5	92.8
2019	San Martin	Men	60+	Healthy	67.5	65.3	69.7
2019	San Martin	Men	60+	Unaware	84.8	78.1	91.4
2019	San Martin	Men	60+	Aware not treated	79.0	79.0	79.0
2019	San Martin	Men	60+	Aware treated	72.0	63.5	80.5
2020	San Martin	Men	<60	Healthy	71.4	70.1	72.7
2020	San Martin	Men	<60	Unaware	89.6	85.9	93.3
2020	San Martin	Men	<60	Aware not treated	80.2	74.3	86.0
2020	San Martin	Men	<60	Aware treated	81.8	70.2	93.4
2020	San Martin	Men	60+	Healthy	68.8	66.2	71.4
2020	San Martin	Men	60+	Unaware	80.6	72.8	88.3

2020	San Martin	Men	60+	Aware not treated	83.5	74.9	92.0
2020	San Martin	Men	60+	Aware treated	74.0	65.9	82.0
2015	San Martin	Women	<60	Healthy	68.0	66.9	69.2
2015	San Martin	Women	<60	Unaware	83.4	80.2	86.7
2015	San Martin	Women	<60	Aware not treated	76.7	71.0	82.4
2015	San Martin	Women	<60	Aware treated	77.1	72.3	81.9
2015	San Martin	Women	60+	Healthy	64.9	62.0	67.7
2015	San Martin	Women	60+	Unaware	74.4	71.6	77.1
2015	San Martin	Women	60+	Aware not treated	70.5	66.5	74.5
2015	San Martin	Women	60+	Aware treated	71.4	66.4	76.4
2016	San Martin	Women	<60	Healthy	67.5	66.4	68.5
2016	San Martin	Women	<60	Unaware	87.1	80.7	93.5
2016	San Martin	Women	<60	Aware not treated	71.8	65.0	78.5
2016	San Martin	Women	<60	Aware treated	76.5	70.5	82.5
2016	San Martin	Women	60+	Healthy	61.1	58.1	64.1
2016	San Martin	Women	60+	Unaware	72.2	66.8	77.7
2016	San Martin	Women	60+	Aware not treated	70.3	65.6	75.0
2016	San Martin	Women	60+	Aware treated	72.4	67.6	77.3
2017	San Martin	Women	<60	Healthy	67.5	66.4	68.6
2017	San Martin	Women	<60	Unaware	89.8	84.5	95.1
2017	San Martin	Women	<60	Aware not treated	75.9	69.8	81.9
2017	San Martin	Women	<60	Aware treated	79.7	73.6	85.7
2017	San Martin	Women	60+	Healthy	64.0	61.8	66.2
2017	San Martin	Women	60+	Unaware	77.8	70.3	85.3
2017	San Martin	Women	60+	Aware not treated	69.6	63.8	75.4
2017	San Martin	Women	60+	Aware treated	69.4	64.0	74.9
2018	San Martin	Women	<60	Healthy	68.3	67.2	69.4
2018	San Martin	Women	<60	Unaware	88.5	85.1	91.8
2018	San Martin	Women	<60	Aware not treated	77.2	71.9	82.5

2018	San Martin	Women	<60	Aware treated	79.8	75.6	83.9
2018	San Martin	Women	60+	Healthy	68.4	65.8	71.0
2018	San Martin	Women	60+	Unaware	76.9	70.4	83.3
2018	San Martin	Women	60+	Aware not treated	71.6	59.4	83.9
2018	San Martin	Women	60+	Aware treated	75.4	70.8	80.0
2019	San Martin	Women	<60	Healthy	68.6	67.7	69.5
2019	San Martin	Women	<60	Unaware	88.0	82.4	93.6
2019	San Martin	Women	<60	Aware not treated	80.7	73.3	88.1
2019	San Martin	Women	<60	Aware treated	82.7	76.1	89.2
2019	San Martin	Women	60+	Healthy	62.3	59.6	65.1
2019	San Martin	Women	60+	Unaware	73.8	70.8	76.8
2019	San Martin	Women	60+	Aware not treated	66.5	58.2	74.8
2019	San Martin	Women	60+	Aware treated	71.7	67.9	75.6
2020	San Martin	Women	<60	Healthy	69.9	68.9	70.9
2020	San Martin	Women	<60	Unaware	87.8	83.2	92.5
2020	San Martin	Women	<60	Aware not treated	77.8	71.7	83.8
2020	San Martin	Women	<60	Aware treated	72.3	67.8	76.8
2020	San Martin	Women	60+	Healthy	64.5	61.1	67.9
2020	San Martin	Women	60+	Unaware	77.6	71.9	83.3
2020	San Martin	Women	60+	Aware not treated	76.0	61.9	90.0
2020	San Martin	Women	60+	Aware treated	72.8	68.4	77.1
2015	Tacna	Men	<60	Healthy	73.1	72.0	74.3
2015	Tacna	Men	<60	Unaware	88.9	86.4	91.3
2015	Tacna	Men	<60	Aware not treated	80.1	74.0	86.2
2015	Tacna	Men	<60	Aware treated	79.2	75.6	82.9
2015	Tacna	Men	60+	Healthy	71.5	67.6	75.4
2015	Tacna	Men	60+	Unaware	81.8	76.6	87.0
2015	Tacna	Men	60+	Aware not treated	76.9	68.7	85.1
2015	Tacna	Men	60+	Aware treated	73.9	65.7	82.1

2016	Tacna	Men	<60	Healthy	75.1	73.8	76.4
2016	Tacna	Men	<60	Unaware	88.2	85.6	90.9
2016	Tacna	Men	<60	Aware not treated	80.4	72.4	88.4
2016	Tacna	Men	<60	Aware treated	77.0	72.8	81.2
2016	Tacna	Men	60+	Healthy	68.5	65.9	71.1
2016	Tacna	Men	60+	Unaware	76.0	71.2	80.9
2016	Tacna	Men	60+	Aware not treated	84.9	70.6	99.2
2016	Tacna	Men	60+	Aware treated	75.7	70.6	80.8
2017	Tacna	Men	<60	Healthy	75.2	74.0	76.4
2017	Tacna	Men	<60	Unaware	87.2	84.9	89.4
2017	Tacna	Men	<60	Aware not treated	85.0	78.3	91.7
2017	Tacna	Men	<60	Aware treated	91.5	84.8	98.1
2017	Tacna	Men	60+	Healthy	69.4	66.6	72.2
2017	Tacna	Men	60+	Unaware	77.5	74.5	80.5
2017	Tacna	Men	60+	Aware not treated	87.6	77.5	97.7
2017	Tacna	Men	60+	Aware treated	78.7	72.4	85.1
2018	Tacna	Men	<60	Healthy	76.3	75.4	77.3
2018	Tacna	Men	<60	Unaware	91.3	89.5	93.1
2018	Tacna	Men	<60	Aware not treated	91.3	79.4	103.3
2018	Tacna	Men	<60	Aware treated	88.6	83.7	93.5
2018	Tacna	Men	60+	Healthy	71.4	67.8	74.9
2018	Tacna	Men	60+	Unaware	89.9	83.4	96.4
2018	Tacna	Men	60+	Aware not treated	83.2	78.6	87.9
2018	Tacna	Men	60+	Aware treated	79.8	75.6	84.0
2019	Tacna	Men	<60	Healthy	76.2	75.2	77.2
2019	Tacna	Men	<60	Unaware	91.1	88.9	93.2
2019	Tacna	Men	<60	Aware not treated	79.1	73.5	84.6
2019	Tacna	Men	<60	Aware treated	82.1	71.9	92.4
2019	Tacna	Men	60+	Healthy	72.9	70.6	75.2

2019	Tacna	Men	60+	Unaware	85.5	78.3	92.8
2019	Tacna	Men	60+	Aware not treated	89.8	79.8	99.8
2019	Tacna	Men	60+	Aware treated	81.0	75.7	86.4
2020	Tacna	Men	<60	Healthy	73.7	72.5	74.9
2020	Tacna	Men	<60	Unaware	92.6	88.4	96.8
2020	Tacna	Men	<60	Aware not treated	87.4	76.7	98.2
2020	Tacna	Men	<60	Aware treated	79.6	67.4	91.7
2020	Tacna	Men	60+	Healthy	72.4	69.0	75.9
2020	Tacna	Men	60+	Unaware	81.8	78.4	85.3
2020	Tacna	Men	60+	Aware not treated	75.8	51.1	100.5
2020	Tacna	Men	60+	Aware treated	73.6	68.6	78.7
2015	Tacna	Women	<60	Healthy	70.2	69.2	71.2
2015	Tacna	Women	<60	Unaware	85.0	81.3	88.7
2015	Tacna	Women	<60	Aware not treated	74.3	69.0	79.6
2015	Tacna	Women	<60	Aware treated	78.8	68.5	89.2
2015	Tacna	Women	60+	Healthy	69.0	65.1	72.9
2015	Tacna	Women	60+	Unaware	76.0	67.4	84.5
2015	Tacna	Women	60+	Aware not treated	72.4	68.5	76.2
2015	Tacna	Women	60+	Aware treated	69.6	64.6	74.6
2016	Tacna	Women	<60	Healthy	70.4	69.4	71.5
2016	Tacna	Women	<60	Unaware	84.4	78.9	89.9
2016	Tacna	Women	<60	Aware not treated	72.0	68.2	75.7
2016	Tacna	Women	<60	Aware treated	83.1	72.8	93.4
2016	Tacna	Women	60+	Healthy	67.2	64.9	69.6
2016	Tacna	Women	60+	Unaware	75.7	72.2	79.2
2016	Tacna	Women	60+	Aware not treated	79.4	72.8	86.1
2016	Tacna	Women	60+	Aware treated	68.6	65.1	72.0
2017	Tacna	Women	<60	Healthy	70.1	69.1	71.1
2017	Tacna	Women	<60	Unaware	85.8	78.8	92.8

2017	Tacna	Women	<60	Aware not treated	76.5	71.2	81.8
2017	Tacna	Women	<60	Aware treated	76.1	69.8	82.5
2017	Tacna	Women	60+	Healthy	66.3	64.2	68.4
2017	Tacna	Women	60+	Unaware	74.5	69.5	79.4
2017	Tacna	Women	60+	Aware not treated	70.9	66.1	75.7
2017	Tacna	Women	60+	Aware treated	69.4	65.7	73.1
2018	Tacna	Women	<60	Healthy	72.8	71.8	73.8
2018	Tacna	Women	<60	Unaware	88.7	85.7	91.7
2018	Tacna	Women	<60	Aware not treated	79.6	71.2	88.0
2018	Tacna	Women	<60	Aware treated	79.4	70.4	88.4
2018	Tacna	Women	60+	Healthy	69.1	65.6	72.7
2018	Tacna	Women	60+	Unaware	79.3	71.6	87.1
2018	Tacna	Women	60+	Aware not treated	79.1	67.6	90.6
2018	Tacna	Women	60+	Aware treated	75.3	70.0	80.5
2019	Tacna	Women	<60	Healthy	70.9	69.8	72.0
2019	Tacna	Women	<60	Unaware	85.9	81.0	90.8
2019	Tacna	Women	<60	Aware not treated	80.7	74.7	86.7
2019	Tacna	Women	<60	Aware treated	78.9	73.3	84.5
2019	Tacna	Women	60+	Healthy	71.1	68.9	73.3
2019	Tacna	Women	60+	Unaware	78.3	73.9	82.8
2019	Tacna	Women	60+	Aware not treated	72.0	63.7	80.2
2019	Tacna	Women	60+	Aware treated	71.0	66.9	75.0
2020	Tacna	Women	<60	Healthy	70.8	69.6	71.9
2020	Tacna	Women	<60	Unaware	82.5	74.9	90.1
2020	Tacna	Women	<60	Aware not treated	72.0	69.0	75.0
2020	Tacna	Women	<60	Aware treated	78.1	72.9	83.3
2020	Tacna	Women	60+	Healthy	68.9	64.7	73.1
2020	Tacna	Women	60+	Unaware	80.0	76.3	83.7
2020	Tacna	Women	60+	Aware not treated	60.1	56.4	63.8

2020	Tacna	Women	60+	Aware treated	73.3	68.8	77.9
2015	Tumbes	Men	<60	Healthy	71.2	70.0	72.4
2015	Tumbes	Men	<60	Unaware	85.2	82.7	87.7
2015	Tumbes	Men	<60	Aware not treated	80.7	75.4	86.0
2015	Tumbes	Men	<60	Aware treated	88.9	82.6	95.2
2015	Tumbes	Men	60+	Healthy	66.6	63.9	69.4
2015	Tumbes	Men	60+	Unaware	80.3	77.0	83.6
2015	Tumbes	Men	60+	Aware not treated	77.0	77.0	77.0
2015	Tumbes	Men	60+	Aware treated	70.8	67.0	74.6
2016	Tumbes	Men	<60	Healthy	71.5	70.2	72.7
2016	Tumbes	Men	<60	Unaware	87.9	85.4	90.4
2016	Tumbes	Men	<60	Aware not treated	79.4	74.1	84.7
2016	Tumbes	Men	<60	Aware treated	84.5	77.5	91.5
2016	Tumbes	Men	60+	Healthy	66.0	64.0	68.0
2016	Tumbes	Men	60+	Unaware	87.0	80.1	94.0
2016	Tumbes	Men	60+	Aware not treated	81.5	70.1	92.9
2016	Tumbes	Men	60+	Aware treated	72.1	66.4	77.9
2017	Tumbes	Men	<60	Healthy	71.1	70.1	72.1
2017	Tumbes	Men	<60	Unaware	90.0	85.8	94.2
2017	Tumbes	Men	<60	Aware not treated	77.6	73.0	82.2
2017	Tumbes	Men	<60	Aware treated	82.9	75.9	89.9
2017	Tumbes	Men	60+	Healthy	66.4	62.9	70.0
2017	Tumbes	Men	60+	Unaware	76.5	71.6	81.4
2017	Tumbes	Men	60+	Aware not treated	69.0	58.0	80.1
2017	Tumbes	Men	60+	Aware treated	76.4	70.3	82.6
2018	Tumbes	Men	<60	Healthy	73.9	72.8	75.0
2018	Tumbes	Men	<60	Unaware	93.0	90.5	95.5
2018	Tumbes	Men	<60	Aware not treated	83.1	79.0	87.2
2018	Tumbes	Men	<60	Aware treated	79.8	74.3	85.3

2018	Tumbes	Men	60+	Healthy	71.8	68.0	75.6
2018	Tumbes	Men	60+	Unaware	81.8	78.4	85.1
2018	Tumbes	Men	60+	Aware not treated	82.1	69.3	94.8
2018	Tumbes	Men	60+	Aware treated	76.1	70.8	81.4
2019	Tumbes	Men	<60	Healthy	73.5	72.5	74.5
2019	Tumbes	Men	<60	Unaware	88.7	86.1	91.3
2019	Tumbes	Men	<60	Aware not treated	86.3	81.6	91.1
2019	Tumbes	Men	<60	Aware treated	90.6	82.4	98.8
2019	Tumbes	Men	60+	Healthy	68.5	64.4	72.6
2019	Tumbes	Men	60+	Unaware	89.1	85.8	92.3
2019	Tumbes	Men	60+	Aware not treated	79.4	66.0	92.9
2019	Tumbes	Men	60+	Aware treated	75.6	68.7	82.5
2020	Tumbes	Men	<60	Healthy	72.3	70.9	73.6
2020	Tumbes	Men	<60	Unaware	94.7	91.8	97.5
2020	Tumbes	Men	<60	Aware not treated	83.1	76.3	89.9
2020	Tumbes	Men	<60	Aware treated	81.1	76.6	85.7
2020	Tumbes	Men	60+	Healthy	68.2	65.4	71.0
2020	Tumbes	Men	60+	Unaware	81.7	78.6	84.9
2020	Tumbes	Men	60+	Aware not treated	77.6	70.7	84.4
2020	Tumbes	Men	60+	Aware treated	78.3	66.2	90.3
2015	Tumbes	Women	<60	Healthy	67.3	66.1	68.5
2015	Tumbes	Women	<60	Unaware	83.4	77.4	89.3
2015	Tumbes	Women	<60	Aware not treated	75.2	67.4	82.9
2015	Tumbes	Women	<60	Aware treated	76.1	71.7	80.4
2015	Tumbes	Women	60+	Healthy	62.8	59.1	66.4
2015	Tumbes	Women	60+	Unaware	75.5	67.9	83.1
2015	Tumbes	Women	60+	Aware not treated	78.0	78.0	78.0
2015	Tumbes	Women	60+	Aware treated	67.6	64.4	70.7
2016	Tumbes	Women	<60	Healthy	67.4	66.4	68.4

2016	Tumbes	Women	<60	Unaware	89.3	82.3	96.4
2016	Tumbes	Women	<60	Aware not treated	75.0	70.5	79.6
2016	Tumbes	Women	<60	Aware treated	74.6	70.7	78.6
2016	Tumbes	Women	60+	Healthy	65.0	61.8	68.2
2016	Tumbes	Women	60+	Unaware	73.3	70.0	76.6
2016	Tumbes	Women	60+	Aware not treated	66.7	62.4	71.0
2016	Tumbes	Women	60+	Aware treated	73.5	69.2	77.7
2017	Tumbes	Women	<60	Healthy	68.1	67.2	69.0
2017	Tumbes	Women	<60	Unaware	93.7	88.6	98.8
2017	Tumbes	Women	<60	Aware not treated	72.2	67.1	77.3
2017	Tumbes	Women	<60	Aware treated	77.3	73.4	81.2
2017	Tumbes	Women	60+	Healthy	67.2	62.8	71.6
2017	Tumbes	Women	60+	Unaware	77.4	63.0	91.8
2017	Tumbes	Women	60+	Aware not treated	71.9	63.7	80.0
2017	Tumbes	Women	60+	Aware treated	71.2	66.3	76.1
2018	Tumbes	Women	<60	Healthy	70.4	69.3	71.5
2018	Tumbes	Women	<60	Unaware	88.9	84.8	93.0
2018	Tumbes	Women	<60	Aware not treated	74.1	70.2	77.9
2018	Tumbes	Women	<60	Aware treated	81.6	77.5	85.7
2018	Tumbes	Women	60+	Healthy	67.4	65.0	69.9
2018	Tumbes	Women	60+	Unaware	79.3	76.2	82.5
2018	Tumbes	Women	60+	Aware not treated	73.2	65.4	81.1
2018	Tumbes	Women	60+	Aware treated	71.4	66.9	76.0
2019	Tumbes	Women	<60	Healthy	69.6	68.6	70.6
2019	Tumbes	Women	<60	Unaware	92.4	90.2	94.6
2019	Tumbes	Women	<60	Aware not treated	76.2	69.3	83.0
2019	Tumbes	Women	<60	Aware treated	78.8	74.2	83.5
2019	Tumbes	Women	60+	Healthy	69.4	66.8	72.0
2019	Tumbes	Women	60+	Unaware	70.8	66.7	75.0

2019	Tumbes	Women	60+	Aware not treated	78.6	68.9	88.3
2019	Tumbes	Women	60+	Aware treated	70.8	67.9	73.8
2020	Tumbes	Women	<60	Healthy	71.6	70.5	72.7
2020	Tumbes	Women	<60	Unaware	89.2	81.6	96.7
2020	Tumbes	Women	<60	Aware not treated	83.7	73.0	94.4
2020	Tumbes	Women	<60	Aware treated	80.6	75.5	85.7
2020	Tumbes	Women	60+	Healthy	64.6	61.5	67.8
2020	Tumbes	Women	60+	Unaware	78.1	71.9	84.3
2020	Tumbes	Women	60+	Aware not treated	66.5	60.5	72.4
2020	Tumbes	Women	60+	Aware treated	75.3	69.6	81.0
2015	Ucayali	Men	<60	Healthy	69.2	68.4	70.1
2015	Ucayali	Men	<60	Unaware	83.2	79.9	86.5
2015	Ucayali	Men	<60	Aware not treated	77.1	73.3	80.9
2015	Ucayali	Men	<60	Aware treated	83.1	76.0	90.1
2015	Ucayali	Men	60+	Healthy	67.0	64.4	69.7
2015	Ucayali	Men	60+	Unaware	79.2	73.8	84.6
2015	Ucayali	Men	60+	Aware not treated	67.7	64.0	71.5
2015	Ucayali	Men	60+	Aware treated	69.7	59.0	80.4
2016	Ucayali	Men	<60	Healthy	69.5	68.5	70.5
2016	Ucayali	Men	<60	Unaware	84.4	79.0	89.7
2016	Ucayali	Men	<60	Aware not treated	76.6	67.2	85.9
2016	Ucayali	Men	<60	Aware treated	81.8	74.8	88.9
2016	Ucayali	Men	60+	Healthy	65.5	62.5	68.6
2016	Ucayali	Men	60+	Unaware	75.2	66.7	83.6
2016	Ucayali	Men	60+	Aware not treated	60.0	60.0	60.0
2016	Ucayali	Men	60+	Aware treated	73.0	67.9	78.2
2017	Ucayali	Men	<60	Healthy	69.9	68.8	71.1
2017	Ucayali	Men	<60	Unaware	88.6	85.4	91.8
2017	Ucayali	Men	<60	Aware treated	76.5	64.8	88.2

2017	Ucayali	Men	60+	Healthy	68.1	65.8	70.4
2017	Ucayali	Men	60+	Unaware	80.2	77.6	82.9
2017	Ucayali	Men	60+	Aware not treated	73.5	68.9	78.0
2017	Ucayali	Men	60+	Aware treated	71.4	63.9	79.0
2018	Ucayali	Men	<60	Healthy	73.4	72.3	74.4
2018	Ucayali	Men	<60	Unaware	88.3	84.4	92.3
2018	Ucayali	Men	<60	Aware not treated	82.2	79.3	85.1
2018	Ucayali	Men	<60	Aware treated	93.4	86.6	100.1
2018	Ucayali	Men	60+	Healthy	68.4	65.8	70.9
2018	Ucayali	Men	60+	Unaware	81.8	73.6	90.0
2018	Ucayali	Men	60+	Aware not treated	88.0	78.4	97.7
2018	Ucayali	Men	60+	Aware treated	80.3	71.8	88.7
2019	Ucayali	Men	<60	Healthy	71.2	70.0	72.3
2019	Ucayali	Men	<60	Unaware	92.1	88.2	95.9
2019	Ucayali	Men	<60	Aware not treated	75.0	72.9	77.2
2019	Ucayali	Men	<60	Aware treated	78.3	71.2	85.3
2019	Ucayali	Men	60+	Healthy	68.2	65.4	71.0
2019	Ucayali	Men	60+	Unaware	72.9	63.2	82.6
2019	Ucayali	Men	60+	Aware not treated	73.0	73.0	73.0
2019	Ucayali	Men	60+	Aware treated	73.8	69.0	78.5
2020	Ucayali	Men	<60	Healthy	71.1	69.7	72.5
2020	Ucayali	Men	<60	Unaware	87.2	83.6	90.8
2020	Ucayali	Men	<60	Aware not treated	78.7	72.0	85.4
2020	Ucayali	Men	<60	Aware treated	82.8	74.0	91.6
2020	Ucayali	Men	60+	Healthy	69.2	66.6	71.9
2020	Ucayali	Men	60+	Unaware	75.8	70.1	81.4
2020	Ucayali	Men	60+	Aware not treated	78.3	76.0	80.6
2020	Ucayali	Men	60+	Aware treated	72.5	67.8	77.2
2015	Ucayali	Women	<60	Healthy	65.1	64.1	66.0

2015	Ucayali	Women	<60	Unaware	85.4	83.1	87.6
2015	Ucayali	Women	<60	Aware not treated	65.6	62.5	68.8
2015	Ucayali	Women	<60	Aware treated	68.6	65.6	71.6
2015	Ucayali	Women	60+	Healthy	61.1	58.2	63.9
2015	Ucayali	Women	60+	Unaware	69.5	61.8	77.2
2015	Ucayali	Women	60+	Aware not treated	70.5	58.6	82.3
2015	Ucayali	Women	60+	Aware treated	65.5	59.7	71.3
2016	Ucayali	Women	<60	Healthy	64.8	63.7	65.9
2016	Ucayali	Women	<60	Unaware	75.7	68.1	83.3
2016	Ucayali	Women	<60	Aware not treated	76.0	67.2	84.7
2016	Ucayali	Women	<60	Aware treated	74.8	70.1	79.5
2016	Ucayali	Women	60+	Healthy	62.9	60.5	65.3
2016	Ucayali	Women	60+	Unaware	75.1	68.4	81.9
2016	Ucayali	Women	60+	Aware not treated	76.0	76.0	76.0
2016	Ucayali	Women	60+	Aware treated	70.0	66.2	73.9
2017	Ucayali	Women	<60	Healthy	66.6	65.6	67.7
2017	Ucayali	Women	<60	Unaware	78.4	73.7	83.1
2017	Ucayali	Women	<60	Aware not treated	77.1	70.9	83.3
2017	Ucayali	Women	<60	Aware treated	74.6	70.0	79.2
2017	Ucayali	Women	60+	Healthy	61.7	59.5	63.9
2017	Ucayali	Women	60+	Unaware	69.1	62.6	75.6
2017	Ucayali	Women	60+	Aware not treated	65.5	58.5	72.6
2017	Ucayali	Women	60+	Aware treated	69.0	65.1	72.9
2018	Ucayali	Women	<60	Healthy	69.6	68.6	70.5
2018	Ucayali	Women	<60	Unaware	84.8	80.5	89.1
2018	Ucayali	Women	<60	Aware not treated	69.4	64.6	74.1
2018	Ucayali	Women	<60	Aware treated	78.3	73.7	82.9
2018	Ucayali	Women	60+	Healthy	65.0	62.6	67.4
2018	Ucayali	Women	60+	Unaware	78.2	72.5	83.8

2018	Ucayali	Women	60+	Aware not treated	77.8	71.6	84.0
2018	Ucayali	Women	60+	Aware treated	71.0	65.8	76.2
2019	Ucayali	Women	<60	Healthy	67.8	66.7	69.0
2019	Ucayali	Women	<60	Unaware	82.8	78.1	87.5
2019	Ucayali	Women	<60	Aware not treated	75.3	64.5	86.0
2019	Ucayali	Women	<60	Aware treated	76.2	70.7	81.7
2019	Ucayali	Women	60+	Healthy	63.9	61.5	66.3
2019	Ucayali	Women	60+	Unaware	76.5	68.9	84.0
2019	Ucayali	Women	60+	Aware not treated	60.2	40.4	80.1
2019	Ucayali	Women	60+	Aware treated	71.7	67.4	76.0
2020	Ucayali	Women	<60	Healthy	67.8	66.6	69.1
2020	Ucayali	Women	<60	Unaware	81.5	75.8	87.1
2020	Ucayali	Women	<60	Aware not treated	70.9	65.4	76.3
2020	Ucayali	Women	<60	Aware treated	73.3	67.3	79.3
2020	Ucayali	Women	60+	Healthy	64.7	61.5	67.9
2020	Ucayali	Women	60+	Unaware	76.2	71.0	81.5
2020	Ucayali	Women	60+	Aware treated	66.1	59.8	72.5
2015	Apurimac	Men	<60	Healthy	72.7	71.4	73.9
2015	Apurimac	Men	<60	Unaware	89.6	87.0	92.3
2015	Apurimac	Men	<60	Aware not treated	80.2	72.0	88.4
2015	Apurimac	Men	<60	Aware treated	80.8	76.5	85.0
2015	Apurimac	Men	60+	Healthy	69.6	67.0	72.3
2015	Apurimac	Men	60+	Unaware	83.7	79.4	88.0
2015	Apurimac	Men	60+	Aware not treated	60.8	57.6	64.0
2015	Apurimac	Men	60+	Aware treated	77.0	68.0	85.9
2016	Apurimac	Men	<60	Healthy	72.3	71.4	73.3
2016	Apurimac	Men	<60	Unaware	89.7	86.9	92.4
2016	Apurimac	Men	<60	Aware not treated	68.2	58.4	78.0
2016	Apurimac	Men	<60	Aware treated	85.5	74.2	96.9

2016	Apurimac	Men	60+	Healthy	72.3	69.9	74.7
2016	Apurimac	Men	60+	Unaware	81.2	77.2	85.2
2016	Apurimac	Men	60+	Aware not treated	73.7	65.9	81.5
2016	Apurimac	Men	60+	Aware treated	71.6	57.0	86.1
2017	Apurimac	Men	<60	Healthy	73.1	72.0	74.3
2017	Apurimac	Men	<60	Unaware	87.9	85.3	90.5
2017	Apurimac	Men	<60	Aware not treated	81.0	75.3	86.7
2017	Apurimac	Men	<60	Aware treated	87.0	80.0	94.1
2017	Apurimac	Men	60+	Healthy	71.3	66.7	75.8
2017	Apurimac	Men	60+	Unaware	82.0	75.3	88.8
2017	Apurimac	Men	60+	Aware not treated	74.2	62.1	86.3
2017	Apurimac	Men	60+	Aware treated	79.4	70.5	88.2
2018	Apurimac	Men	<60	Healthy	74.6	73.7	75.5
2018	Apurimac	Men	<60	Unaware	92.6	89.7	95.5
2018	Apurimac	Men	<60	Aware not treated	77.4	74.7	80.1
2018	Apurimac	Men	<60	Aware treated	86.9	74.1	99.6
2018	Apurimac	Men	60+	Healthy	73.9	71.1	76.7
2018	Apurimac	Men	60+	Unaware	84.8	79.3	90.4
2018	Apurimac	Men	60+	Aware not treated	97.3	92.4	102.2
2018	Apurimac	Men	60+	Aware treated	77.7	71.4	83.9
2019	Apurimac	Men	<60	Healthy	74.3	73.2	75.4
2019	Apurimac	Men	<60	Unaware	91.4	88.5	94.4
2019	Apurimac	Men	<60	Aware not treated	84.1	76.9	91.3
2019	Apurimac	Men	<60	Aware treated	99.4	74.5	124.2
2019	Apurimac	Men	60+	Healthy	70.7	68.6	72.8
2019	Apurimac	Men	60+	Unaware	83.8	79.7	87.9
2019	Apurimac	Men	60+	Aware not treated	75.2	66.5	83.9
2019	Apurimac	Men	60+	Aware treated	80.6	72.4	88.8
2020	Apurimac	Men	<60	Healthy	72.9	71.7	74.0

2020	Apurimac	Men	<60	Unaware	89.3	85.7	92.9
2020	Apurimac	Men	<60	Aware not treated	76.6	75.3	78.0
2020	Apurimac	Men	<60	Aware treated	86.4	74.5	98.3
2020	Apurimac	Men	60+	Healthy	69.0	64.8	73.2
2020	Apurimac	Men	60+	Unaware	87.6	78.9	96.3
2020	Apurimac	Men	60+	Aware not treated	77.9	74.4	81.3
2020	Apurimac	Men	60+	Aware treated	77.2	68.0	86.3
2015	Apurimac	Women	<60	Healthy	70.0	68.7	71.3
2015	Apurimac	Women	<60	Unaware	83.2	79.5	86.8
2015	Apurimac	Women	<60	Aware not treated	72.2	67.8	76.6
2015	Apurimac	Women	<60	Aware treated	84.2	78.5	89.8
2015	Apurimac	Women	60+	Healthy	65.7	63.8	67.5
2015	Apurimac	Women	60+	Unaware	80.0	77.0	82.9
2015	Apurimac	Women	60+	Aware not treated	72.0	61.3	82.7
2015	Apurimac	Women	60+	Aware treated	76.7	72.0	81.4
2016	Apurimac	Women	<60	Healthy	70.7	69.6	71.7
2016	Apurimac	Women	<60	Unaware	89.5	86.5	92.4
2016	Apurimac	Women	<60	Aware not treated	72.5	69.3	75.6
2016	Apurimac	Women	<60	Aware treated	75.5	70.9	80.0
2016	Apurimac	Women	60+	Healthy	67.3	65.2	69.4
2016	Apurimac	Women	60+	Unaware	74.5	70.0	79.0
2016	Apurimac	Women	60+	Aware not treated	72.9	64.7	81.1
2016	Apurimac	Women	60+	Aware treated	74.1	67.0	81.2
2017	Apurimac	Women	<60	Healthy	70.5	69.7	71.3
2017	Apurimac	Women	<60	Unaware	90.0	86.6	93.5
2017	Apurimac	Women	<60	Aware not treated	78.5	73.3	83.6
2017	Apurimac	Women	<60	Aware treated	80.0	72.5	87.5
2017	Apurimac	Women	60+	Healthy	65.7	63.9	67.5
2017	Apurimac	Women	60+	Unaware	79.6	77.1	82.2

2017	Apurimac	Women	60+	Aware not treated	74.2	69.2	79.3
2017	Apurimac	Women	60+	Aware treated	76.6	71.7	81.5
2018	Apurimac	Women	<60	Healthy	72.3	71.3	73.4
2018	Apurimac	Women	<60	Unaware	91.3	88.9	93.7
2018	Apurimac	Women	<60	Aware not treated	75.1	70.1	80.1
2018	Apurimac	Women	<60	Aware treated	79.5	68.7	90.3
2018	Apurimac	Women	60+	Healthy	71.3	68.7	73.9
2018	Apurimac	Women	60+	Unaware	86.9	78.5	95.3
2018	Apurimac	Women	60+	Aware not treated	68.3	65.2	71.4
2018	Apurimac	Women	60+	Aware treated	79.6	73.9	85.4
2019	Apurimac	Women	<60	Healthy	71.3	70.1	72.5
2019	Apurimac	Women	<60	Unaware	92.4	88.8	96.0
2019	Apurimac	Women	<60	Aware not treated	80.9	72.0	89.7
2019	Apurimac	Women	<60	Aware treated	80.1	72.6	87.7
2019	Apurimac	Women	60+	Healthy	67.8	65.5	70.1
2019	Apurimac	Women	60+	Unaware	86.1	81.8	90.3
2019	Apurimac	Women	60+	Aware not treated	77.9	71.9	84.0
2019	Apurimac	Women	60+	Aware treated	78.5	73.7	83.3
2020	Apurimac	Women	<60	Healthy	69.2	68.0	70.5
2020	Apurimac	Women	<60	Unaware	78.0	74.0	82.0
2020	Apurimac	Women	<60	Aware not treated	76.3	70.3	82.3
2020	Apurimac	Women	<60	Aware treated	75.2	67.9	82.5
2020	Apurimac	Women	60+	Healthy	66.8	63.4	70.2
2020	Apurimac	Women	60+	Unaware	78.6	73.6	83.5
2020	Apurimac	Women	60+	Aware not treated	72.0	57.2	86.8
2020	Apurimac	Women	60+	Aware treated	76.5	70.2	82.8
2015	Arequipa	Men	<60	Healthy	72.7	71.5	73.9
2015	Arequipa	Men	<60	Unaware	89.7	86.9	92.4
2015	Arequipa	Men	<60	Aware not treated	89.7	83.9	95.4

2015	Arequipa	Men	<60	Aware treated	78.8	71.0	86.6
2015	Arequipa	Men	60+	Healthy	69.1	66.1	72.1
2015	Arequipa	Men	60+	Unaware	74.8	68.6	81.0
2015	Arequipa	Men	60+	Aware not treated	79.0	79.0	79.0
2015	Arequipa	Men	60+	Aware treated	79.2	72.5	86.0
2016	Arequipa	Men	<60	Healthy	74.1	73.0	75.2
2016	Arequipa	Men	<60	Unaware	86.6	83.5	89.8
2016	Arequipa	Men	<60	Aware not treated	85.6	76.5	94.7
2016	Arequipa	Men	<60	Aware treated	73.7	67.5	79.9
2016	Arequipa	Men	60+	Healthy	72.1	70.1	74.0
2016	Arequipa	Men	60+	Unaware	88.9	83.6	94.3
2016	Arequipa	Men	60+	Aware not treated	79.0	79.0	79.0
2016	Arequipa	Men	60+	Aware treated	75.2	70.4	80.0
2017	Arequipa	Men	<60	Healthy	74.2	73.1	75.3
2017	Arequipa	Men	<60	Unaware	87.8	85.4	90.2
2017	Arequipa	Men	<60	Aware not treated	76.4	72.9	79.8
2017	Arequipa	Men	<60	Aware treated	72.8	67.9	77.7
2017	Arequipa	Men	60+	Healthy	71.3	69.6	73.0
2017	Arequipa	Men	60+	Unaware	82.9	78.3	87.6
2017	Arequipa	Men	60+	Aware not treated	79.3	69.3	89.2
2017	Arequipa	Men	60+	Aware treated	77.1	73.0	81.1
2018	Arequipa	Men	<60	Healthy	76.0	74.9	77.0
2018	Arequipa	Men	<60	Unaware	92.6	90.6	94.6
2018	Arequipa	Men	<60	Aware not treated	83.0	74.5	91.4
2018	Arequipa	Men	<60	Aware treated	91.6	73.7	109.6
2018	Arequipa	Men	60+	Healthy	70.2	67.8	72.6
2018	Arequipa	Men	60+	Unaware	84.2	81.0	87.4
2018	Arequipa	Men	60+	Aware not treated	80.7	75.0	86.4
2018	Arequipa	Men	60+	Aware treated	75.5	68.8	82.2

2019	Arequipa	Men	<60	Healthy	75.2	74.3	76.2
2019	Arequipa	Men	<60	Unaware	89.9	87.1	92.8
2019	Arequipa	Men	<60	Aware not treated	80.0	75.2	84.8
2019	Arequipa	Men	<60	Aware treated	84.6	77.9	91.4
2019	Arequipa	Men	60+	Healthy	73.8	71.6	76.1
2019	Arequipa	Men	60+	Unaware	83.6	79.3	87.9
2019	Arequipa	Men	60+	Aware not treated	76.1	66.8	85.5
2019	Arequipa	Men	60+	Aware treated	76.0	72.1	79.9
2020	Arequipa	Men	<60	Healthy	74.7	73.0	76.4
2020	Arequipa	Men	<60	Unaware	90.4	87.9	92.9
2020	Arequipa	Men	<60	Aware not treated	78.0	78.0	78.0
2020	Arequipa	Men	<60	Aware treated	86.7	77.9	95.5
2020	Arequipa	Men	60+	Healthy	72.5	69.6	75.5
2020	Arequipa	Men	60+	Unaware	84.2	76.0	92.4
2020	Arequipa	Men	60+	Aware not treated	77.0	70.7	83.3
2020	Arequipa	Men	60+	Aware treated	71.2	64.5	77.9
2015	Arequipa	Women	<60	Healthy	69.2	68.1	70.4
2015	Arequipa	Women	<60	Unaware	85.3	82.2	88.5
2015	Arequipa	Women	<60	Aware not treated	70.4	64.5	76.3
2015	Arequipa	Women	<60	Aware treated	79.4	76.7	82.1
2015	Arequipa	Women	60+	Healthy	66.9	64.0	69.8
2015	Arequipa	Women	60+	Unaware	79.0	74.0	84.1
2015	Arequipa	Women	60+	Aware not treated	62.2	56.9	67.5
2015	Arequipa	Women	60+	Aware treated	68.4	64.0	72.9
2016	Arequipa	Women	<60	Healthy	69.7	68.7	70.7
2016	Arequipa	Women	<60	Unaware	82.0	77.9	86.1
2016	Arequipa	Women	<60	Aware not treated	72.9	66.0	79.7
2016	Arequipa	Women	<60	Aware treated	78.1	70.1	86.1
2016	Arequipa	Women	60+	Healthy	66.0	63.5	68.4

2016	Arequipa	Women	60+	Unaware	72.3	62.0	82.6
2016	Arequipa	Women	60+	Aware not treated	70.3	61.6	79.0
2016	Arequipa	Women	60+	Aware treated	69.5	65.3	73.8
2017	Arequipa	Women	<60	Healthy	69.2	68.2	70.2
2017	Arequipa	Women	<60	Unaware	88.8	87.0	90.6
2017	Arequipa	Women	<60	Aware not treated	71.9	67.5	76.3
2017	Arequipa	Women	<60	Aware treated	79.6	71.6	87.5
2017	Arequipa	Women	60+	Healthy	66.8	64.3	69.4
2017	Arequipa	Women	60+	Unaware	73.2	66.2	80.1
2017	Arequipa	Women	60+	Aware not treated	77.1	73.4	80.7
2017	Arequipa	Women	60+	Aware treated	72.6	66.3	79.0
2018	Arequipa	Women	<60	Healthy	72.2	71.2	73.2
2018	Arequipa	Women	<60	Unaware	92.5	89.5	95.5
2018	Arequipa	Women	<60	Aware not treated	77.3	71.6	83.0
2018	Arequipa	Women	<60	Aware treated	81.3	74.5	88.2
2018	Arequipa	Women	60+	Healthy	71.3	69.2	73.4
2018	Arequipa	Women	60+	Unaware	83.0	77.8	88.3
2018	Arequipa	Women	60+	Aware not treated	70.2	61.8	78.7
2018	Arequipa	Women	60+	Aware treated	74.9	70.5	79.2
2019	Arequipa	Women	<60	Healthy	69.0	68.1	69.9
2019	Arequipa	Women	<60	Unaware	87.7	83.7	91.6
2019	Arequipa	Women	<60	Aware not treated	72.2	66.1	78.3
2019	Arequipa	Women	<60	Aware treated	84.2	78.2	90.2
2019	Arequipa	Women	60+	Healthy	67.1	64.6	69.6
2019	Arequipa	Women	60+	Unaware	80.8	74.3	87.2
2019	Arequipa	Women	60+	Aware not treated	75.0	67.6	82.3
2019	Arequipa	Women	60+	Aware treated	70.7	67.5	73.9
2020	Arequipa	Women	<60	Healthy	70.3	68.9	71.6
2020	Arequipa	Women	<60	Unaware	87.3	84.6	90.0

2020	Arequipa	Women	<60	Aware not treated	79.4	76.0	82.9
2020	Arequipa	Women	<60	Aware treated	71.3	64.8	77.8
2020	Arequipa	Women	60+	Healthy	69.2	64.9	73.5
2020	Arequipa	Women	60+	Unaware	80.7	74.5	86.9
2020	Arequipa	Women	60+	Aware not treated	80.3	73.0	87.6
2020	Arequipa	Women	60+	Aware treated	74.4	69.4	79.4
2015	Ayacucho	Men	<60	Healthy	71.8	70.8	72.9
2015	Ayacucho	Men	<60	Unaware	90.6	86.7	94.4
2015	Ayacucho	Men	<60	Aware not treated	76.4	72.0	80.7
2015	Ayacucho	Men	<60	Aware treated	84.5	80.6	88.5
2015	Ayacucho	Men	60+	Healthy	69.1	66.3	71.9
2015	Ayacucho	Men	60+	Unaware	81.5	76.6	86.5
2015	Ayacucho	Men	60+	Aware not treated	74.8	65.1	84.5
2015	Ayacucho	Men	60+	Aware treated	79.2	71.2	87.2
2016	Ayacucho	Men	<60	Healthy	72.3	71.3	73.2
2016	Ayacucho	Men	<60	Unaware	87.9	83.9	92.0
2016	Ayacucho	Men	<60	Aware not treated	85.0	75.9	94.0
2016	Ayacucho	Men	<60	Aware treated	87.6	79.5	95.7
2016	Ayacucho	Men	60+	Healthy	69.4	67.8	71.0
2016	Ayacucho	Men	60+	Unaware	83.3	79.4	87.2
2016	Ayacucho	Men	60+	Aware not treated	78.3	70.7	85.8
2016	Ayacucho	Men	60+	Aware treated	85.8	82.6	89.0
2017	Ayacucho	Men	<60	Healthy	72.6	71.5	73.7
2017	Ayacucho	Men	<60	Unaware	92.4	90.2	94.7
2017	Ayacucho	Men	<60	Aware not treated	79.5	70.7	88.3
2017	Ayacucho	Men	<60	Aware treated	91.8	82.0	101.5
2017	Ayacucho	Men	60+	Healthy	67.4	65.1	69.6
2017	Ayacucho	Men	60+	Unaware	82.4	78.2	86.6
2017	Ayacucho	Men	60+	Aware not treated	81.1	73.9	88.3

2017	Ayacucho	Men	60+	Aware treated	77.6	72.3	82.9
2018	Ayacucho	Men	<60	Healthy	74.7	73.5	76.0
2018	Ayacucho	Men	<60	Unaware	92.1	89.2	95.0
2018	Ayacucho	Men	<60	Aware not treated	80.1	73.2	87.0
2018	Ayacucho	Men	<60	Aware treated	92.7	79.5	106.0
2018	Ayacucho	Men	60+	Healthy	73.4	70.9	75.9
2018	Ayacucho	Men	60+	Unaware	85.0	80.4	89.7
2018	Ayacucho	Men	60+	Aware not treated	78.8	70.6	87.1
2018	Ayacucho	Men	60+	Aware treated	77.6	72.2	82.9
2019	Ayacucho	Men	<60	Healthy	74.5	73.4	75.7
2019	Ayacucho	Men	<60	Unaware	92.2	89.0	95.3
2019	Ayacucho	Men	<60	Aware not treated	78.0	72.8	83.2
2019	Ayacucho	Men	<60	Aware treated	77.6	69.4	85.8
2019	Ayacucho	Men	60+	Healthy	72.6	70.5	74.7
2019	Ayacucho	Men	60+	Unaware	87.8	84.5	91.0
2019	Ayacucho	Men	60+	Aware not treated	78.9	73.8	84.1
2019	Ayacucho	Men	60+	Aware treated	83.2	78.5	87.8
2020	Ayacucho	Men	<60	Healthy	73.2	71.9	74.4
2020	Ayacucho	Men	<60	Unaware	92.6	87.3	97.8
2020	Ayacucho	Men	<60	Aware not treated	81.2	76.7	85.7
2020	Ayacucho	Men	<60	Aware treated	75.0	73.2	76.8
2020	Ayacucho	Men	60+	Healthy	74.2	70.9	77.4
2020	Ayacucho	Men	60+	Unaware	84.9	79.8	89.9
2020	Ayacucho	Men	60+	Aware not treated	72.2	61.5	83.0
2020	Ayacucho	Men	60+	Aware treated	79.8	69.1	90.5
2015	Ayacucho	Women	<60	Healthy	68.3	67.3	69.3
2015	Ayacucho	Women	<60	Unaware	86.0	81.4	90.7
2015	Ayacucho	Women	<60	Aware not treated	69.8	65.4	74.1
2015	Ayacucho	Women	<60	Aware treated	70.9	65.2	76.6

2015	Ayacucho	Women	60+	Healthy	64.7	62.2	67.1
2015	Ayacucho	Women	60+	Unaware	76.5	71.3	81.6
2015	Ayacucho	Women	60+	Aware not treated	81.6	70.6	92.5
2015	Ayacucho	Women	60+	Aware treated	80.1	71.6	88.6
2016	Ayacucho	Women	<60	Healthy	68.3	67.3	69.3
2016	Ayacucho	Women	<60	Unaware	87.6	84.9	90.4
2016	Ayacucho	Women	<60	Aware not treated	69.2	65.2	73.2
2016	Ayacucho	Women	<60	Aware treated	78.2	73.3	83.2
2016	Ayacucho	Women	60+	Healthy	67.0	64.9	69.0
2016	Ayacucho	Women	60+	Unaware	78.1	74.1	82.1
2016	Ayacucho	Women	60+	Aware not treated	76.7	69.6	83.8
2016	Ayacucho	Women	60+	Aware treated	73.1	70.0	76.2
2017	Ayacucho	Women	<60	Healthy	68.1	67.1	69.1
2017	Ayacucho	Women	<60	Unaware	89.2	84.8	93.5
2017	Ayacucho	Women	<60	Aware not treated	73.2	66.5	80.0
2017	Ayacucho	Women	<60	Aware treated	78.3	71.0	85.6
2017	Ayacucho	Women	60+	Healthy	65.2	63.1	67.3
2017	Ayacucho	Women	60+	Unaware	78.5	74.2	82.9
2017	Ayacucho	Women	60+	Aware not treated	68.8	63.1	74.4
2017	Ayacucho	Women	60+	Aware treated	73.6	68.8	78.5
2018	Ayacucho	Women	<60	Healthy	70.8	69.9	71.8
2018	Ayacucho	Women	<60	Unaware	92.4	87.5	97.3
2018	Ayacucho	Women	<60	Aware not treated	76.3	69.0	83.6
2018	Ayacucho	Women	<60	Aware treated	80.9	70.2	91.6
2018	Ayacucho	Women	60+	Healthy	69.6	67.8	71.5
2018	Ayacucho	Women	60+	Unaware	81.2	75.2	87.1
2018	Ayacucho	Women	60+	Aware not treated	66.5	63.0	70.1
2018	Ayacucho	Women	60+	Aware treated	82.4	78.4	86.4
2019	Ayacucho	Women	<60	Healthy	71.5	70.7	72.4

2019	Ayacucho	Women	<60	Unaware	90.6	88.6	92.6
2019	Ayacucho	Women	<60	Aware not treated	80.8	76.0	85.6
2019	Ayacucho	Women	<60	Aware treated	79.8	74.6	85.0
2019	Ayacucho	Women	60+	Healthy	70.1	68.3	71.9
2019	Ayacucho	Women	60+	Unaware	81.5	78.0	84.9
2019	Ayacucho	Women	60+	Aware not treated	72.2	66.5	77.8
2019	Ayacucho	Women	60+	Aware treated	75.9	72.1	79.7
2020	Ayacucho	Women	<60	Healthy	70.3	68.9	71.6
2020	Ayacucho	Women	<60	Unaware	89.1	84.9	93.2
2020	Ayacucho	Women	<60	Aware not treated	79.0	74.8	83.1
2020	Ayacucho	Women	<60	Aware treated	71.3	64.7	77.9
2020	Ayacucho	Women	60+	Healthy	67.7	65.9	69.5
2020	Ayacucho	Women	60+	Unaware	77.7	73.8	81.7
2020	Ayacucho	Women	60+	Aware not treated	82.4	71.7	93.2
2020	Ayacucho	Women	60+	Aware treated	76.9	71.7	82.2
2015	Cajamarca	Men	<60	Healthy	72.7	71.5	73.9
2015	Cajamarca	Men	<60	Unaware	87.0	82.3	91.7
2015	Cajamarca	Men	<60	Aware not treated	73.4	66.9	79.9
2015	Cajamarca	Men	<60	Aware treated	80.0	75.9	84.1
2015	Cajamarca	Men	60+	Healthy	68.2	65.4	71.0
2015	Cajamarca	Men	60+	Unaware	82.7	79.0	86.5
2015	Cajamarca	Men	60+	Aware not treated	67.0	63.7	70.3
2015	Cajamarca	Men	60+	Aware treated	78.4	75.6	81.1
2016	Cajamarca	Men	<60	Healthy	72.4	71.2	73.7
2016	Cajamarca	Men	<60	Unaware	84.9	82.7	87.1
2016	Cajamarca	Men	<60	Aware not treated	79.0	68.0	90.0
2016	Cajamarca	Men	<60	Aware treated	82.5	69.7	95.2
2016	Cajamarca	Men	60+	Healthy	69.6	67.3	72.0
2016	Cajamarca	Men	60+	Unaware	83.9	79.1	88.8

2016	Cajamarca	Men	60+	Aware not treated	86.7	72.7	100.7
2016	Cajamarca	Men	60+	Aware treated	77.0	69.7	84.2
2017	Cajamarca	Men	<60	Healthy	73.5	72.3	74.8
2017	Cajamarca	Men	<60	Unaware	88.7	86.0	91.4
2017	Cajamarca	Men	<60	Aware not treated	82.7	72.1	93.2
2017	Cajamarca	Men	<60	Aware treated	81.7	74.1	89.3
2017	Cajamarca	Men	60+	Healthy	71.2	65.7	76.7
2017	Cajamarca	Men	60+	Unaware	77.4	72.0	82.7
2017	Cajamarca	Men	60+	Aware not treated	84.1	67.3	100.9
2017	Cajamarca	Men	60+	Aware treated	81.8	77.1	86.5
2018	Cajamarca	Men	<60	Healthy	74.3	73.2	75.5
2018	Cajamarca	Men	<60	Unaware	91.2	88.1	94.3
2018	Cajamarca	Men	<60	Aware not treated	81.5	73.8	89.2
2018	Cajamarca	Men	<60	Aware treated	81.3	73.6	89.0
2018	Cajamarca	Men	60+	Healthy	71.0	67.8	74.1
2018	Cajamarca	Men	60+	Unaware	84.3	80.2	88.4
2018	Cajamarca	Men	60+	Aware not treated	80.6	77.7	83.5
2018	Cajamarca	Men	60+	Aware treated	81.5	75.0	88.0
2019	Cajamarca	Men	<60	Healthy	72.5	71.3	73.7
2019	Cajamarca	Men	<60	Unaware	87.9	85.0	90.9
2019	Cajamarca	Men	<60	Aware not treated	76.8	70.1	83.5
2019	Cajamarca	Men	<60	Aware treated	83.4	66.3	100.4
2019	Cajamarca	Men	60+	Healthy	69.9	67.3	72.5
2019	Cajamarca	Men	60+	Unaware	78.3	72.8	83.8
2019	Cajamarca	Men	60+	Aware not treated	74.9	67.9	81.9
2019	Cajamarca	Men	60+	Aware treated	77.2	69.1	85.4
2020	Cajamarca	Men	<60	Healthy	72.8	71.4	74.2
2020	Cajamarca	Men	<60	Unaware	90.4	87.5	93.2
2020	Cajamarca	Men	<60	Aware not treated	87.0	82.8	91.2

2020	Cajamarca	Men	<60	Aware treated	86.2	77.2	95.2
2020	Cajamarca	Men	60+	Healthy	69.0	64.8	73.3
2020	Cajamarca	Men	60+	Unaware	88.0	78.6	97.5
2020	Cajamarca	Men	60+	Aware not treated	73.4	67.4	79.4
2020	Cajamarca	Men	60+	Aware treated	82.5	65.7	99.3
2015	Cajamarca	Women	<60	Healthy	70.2	69.2	71.1
2015	Cajamarca	Women	<60	Unaware	85.7	79.5	92.0
2015	Cajamarca	Women	<60	Aware not treated	79.0	74.3	83.8
2015	Cajamarca	Women	<60	Aware treated	73.8	67.9	79.6
2015	Cajamarca	Women	60+	Healthy	67.0	64.8	69.1
2015	Cajamarca	Women	60+	Unaware	71.8	68.4	75.2
2015	Cajamarca	Women	60+	Aware not treated	71.2	58.7	83.8
2015	Cajamarca	Women	60+	Aware treated	77.0	70.1	84.0
2016	Cajamarca	Women	<60	Healthy	70.5	69.2	71.7
2016	Cajamarca	Women	<60	Unaware	83.6	78.3	88.8
2016	Cajamarca	Women	<60	Aware not treated	76.3	70.3	82.3
2016	Cajamarca	Women	<60	Aware treated	77.4	73.0	81.8
2016	Cajamarca	Women	60+	Healthy	65.8	62.6	68.9
2016	Cajamarca	Women	60+	Unaware	82.2	76.5	87.9
2016	Cajamarca	Women	60+	Aware not treated	71.6	64.8	78.5
2016	Cajamarca	Women	60+	Aware treated	71.8	66.0	77.7
2017	Cajamarca	Women	<60	Healthy	69.7	68.6	70.7
2017	Cajamarca	Women	<60	Unaware	89.1	84.0	94.3
2017	Cajamarca	Women	<60	Aware not treated	77.8	70.6	84.9
2017	Cajamarca	Women	<60	Aware treated	79.4	72.8	86.0
2017	Cajamarca	Women	60+	Healthy	66.1	62.7	69.5
2017	Cajamarca	Women	60+	Unaware	78.7	75.2	82.1
2017	Cajamarca	Women	60+	Aware not treated	80.8	73.9	87.6
2017	Cajamarca	Women	60+	Aware treated	75.1	68.4	81.8

2018	Cajamarca	Women	<60	Healthy	70.8	69.8	71.8
2018	Cajamarca	Women	<60	Unaware	86.5	82.9	90.0
2018	Cajamarca	Women	<60	Aware not treated	78.3	71.9	84.7
2018	Cajamarca	Women	<60	Aware treated	85.1	78.8	91.4
2018	Cajamarca	Women	60+	Healthy	70.0	67.6	72.5
2018	Cajamarca	Women	60+	Unaware	72.6	65.8	79.3
2018	Cajamarca	Women	60+	Aware not treated	72.8	65.2	80.3
2018	Cajamarca	Women	60+	Aware treated	79.7	76.0	83.4
2019	Cajamarca	Women	<60	Healthy	71.6	70.4	72.7
2019	Cajamarca	Women	<60	Unaware	88.1	82.5	93.8
2019	Cajamarca	Women	<60	Aware not treated	76.8	72.3	81.3
2019	Cajamarca	Women	<60	Aware treated	77.5	71.6	83.4
2019	Cajamarca	Women	60+	Healthy	68.6	66.3	70.8
2019	Cajamarca	Women	60+	Unaware	83.1	78.4	87.8
2019	Cajamarca	Women	60+	Aware not treated	70.9	65.6	76.3
2019	Cajamarca	Women	60+	Aware treated	70.8	64.7	77.0
2020	Cajamarca	Women	<60	Healthy	70.4	69.2	71.6
2020	Cajamarca	Women	<60	Unaware	88.7	84.8	92.7
2020	Cajamarca	Women	<60	Aware not treated	80.2	75.1	85.4
2020	Cajamarca	Women	<60	Aware treated	74.8	66.2	83.5
2020	Cajamarca	Women	60+	Healthy	66.8	63.0	70.7
2020	Cajamarca	Women	60+	Unaware	75.7	69.9	81.5
2020	Cajamarca	Women	60+	Aware not treated	75.0	67.6	82.4
2020	Cajamarca	Women	60+	Aware treated	77.5	66.2	88.8
2015	Callao	Men	<60	Healthy	72.9	71.8	74.1
2015	Callao	Men	<60	Unaware	88.9	86.5	91.3
2015	Callao	Men	<60	Aware not treated	82.3	74.9	89.8
2015	Callao	Men	<60	Aware treated	84.5	81.0	88.0
2015	Callao	Men	60+	Healthy	70.0	65.6	74.5

2015	Callao	Men	60+	Unaware	78.5	74.5	82.6
2015	Callao	Men	60+	Aware not treated	77.5	65.4	89.5
2015	Callao	Men	60+	Aware treated	74.4	68.5	80.3
2016	Callao	Men	<60	Healthy	73.5	72.4	74.7
2016	Callao	Men	<60	Unaware	94.5	89.6	99.4
2016	Callao	Men	<60	Aware not treated	89.7	83.0	96.4
2016	Callao	Men	<60	Aware treated	88.3	84.1	92.4
2016	Callao	Men	60+	Healthy	71.1	68.1	74.1
2016	Callao	Men	60+	Unaware	83.7	80.5	86.8
2016	Callao	Men	60+	Aware not treated	81.6	79.1	84.0
2016	Callao	Men	60+	Aware treated	74.6	69.7	79.6
2017	Callao	Men	<60	Healthy	73.0	72.1	73.9
2017	Callao	Men	<60	Unaware	89.3	86.0	92.5
2017	Callao	Men	<60	Aware not treated	92.2	87.2	97.2
2017	Callao	Men	<60	Aware treated	75.1	64.7	85.4
2017	Callao	Men	60+	Healthy	65.9	63.2	68.6
2017	Callao	Men	60+	Unaware	80.8	77.6	83.9
2017	Callao	Men	60+	Aware treated	78.5	68.8	88.2
2018	Callao	Men	<60	Healthy	73.7	72.7	74.6
2018	Callao	Men	<60	Unaware	91.1	89.0	93.3
2018	Callao	Men	<60	Aware not treated	84.2	72.7	95.7
2018	Callao	Men	<60	Aware treated	78.1	70.4	85.8
2018	Callao	Men	60+	Healthy	72.1	69.4	74.8
2018	Callao	Men	60+	Unaware	81.3	75.3	87.2
2018	Callao	Men	60+	Aware treated	81.4	76.4	86.3
2019	Callao	Men	<60	Healthy	73.5	72.3	74.7
2019	Callao	Men	<60	Unaware	89.6	86.9	92.3
2019	Callao	Men	<60	Aware not treated	81.3	74.2	88.5
2019	Callao	Men	<60	Aware treated	88.6	78.6	98.5

2019	Callao	Men	60+	Healthy	68.5	65.7	71.3
2019	Callao	Men	60+	Unaware	85.9	80.8	90.9
2019	Callao	Men	60+	Aware not treated	80.5	79.8	81.2
2019	Callao	Men	60+	Aware treated	75.5	69.4	81.6
2020	Callao	Men	<60	Healthy	74.7	73.0	76.3
2020	Callao	Men	<60	Unaware	94.0	91.6	96.3
2020	Callao	Men	<60	Aware not treated	86.2	80.5	91.8
2020	Callao	Men	<60	Aware treated	90.8	84.2	97.4
2020	Callao	Men	60+	Healthy	72.2	67.9	76.5
2020	Callao	Men	60+	Unaware	90.1	85.5	94.6
2020	Callao	Men	60+	Aware not treated	81.7	67.3	96.1
2020	Callao	Men	60+	Aware treated	80.5	72.1	88.9
2015	Callao	Women	<60	Healthy	69.4	68.4	70.4
2015	Callao	Women	<60	Unaware	91.4	88.6	94.1
2015	Callao	Women	<60	Aware not treated	72.5	66.6	78.5
2015	Callao	Women	<60	Aware treated	80.0	73.6	86.5
2015	Callao	Women	60+	Healthy	67.0	64.3	69.8
2015	Callao	Women	60+	Unaware	75.3	70.8	79.8
2015	Callao	Women	60+	Aware not treated	77.7	75.3	80.1
2015	Callao	Women	60+	Aware treated	71.0	66.9	75.0
2016	Callao	Women	<60	Healthy	69.2	68.1	70.3
2016	Callao	Women	<60	Unaware	86.6	83.7	89.6
2016	Callao	Women	<60	Aware not treated	92.6	72.3	113.0
2016	Callao	Women	<60	Aware treated	76.5	71.6	81.4
2016	Callao	Women	60+	Healthy	64.6	60.6	68.7
2016	Callao	Women	60+	Unaware	68.5	63.3	73.7
2016	Callao	Women	60+	Aware not treated	75.0	75.0	75.0
2016	Callao	Women	60+	Aware treated	69.6	65.9	73.3
2017	Callao	Women	<60	Healthy	69.3	68.2	70.4

2017	Callao	Women	<60	Unaware	86.7	82.5	90.8
2017	Callao	Women	<60	Aware not treated	75.0	70.4	79.6
2017	Callao	Women	<60	Aware treated	77.2	72.4	82.0
2017	Callao	Women	60+	Healthy	64.4	61.9	67.0
2017	Callao	Women	60+	Unaware	78.4	74.3	82.6
2017	Callao	Women	60+	Aware not treated	69.9	68.2	71.6
2017	Callao	Women	60+	Aware treated	73.4	69.1	77.6
2018	Callao	Women	<60	Healthy	70.4	69.5	71.3
2018	Callao	Women	<60	Unaware	87.4	83.6	91.1
2018	Callao	Women	<60	Aware not treated	76.0	70.2	81.8
2018	Callao	Women	<60	Aware treated	76.3	69.8	82.8
2018	Callao	Women	60+	Healthy	66.8	64.6	69.0
2018	Callao	Women	60+	Unaware	75.0	69.7	80.2
2018	Callao	Women	60+	Aware not treated	73.4	66.2	80.5
2018	Callao	Women	60+	Aware treated	74.2	70.5	77.8
2019	Callao	Women	<60	Healthy	70.7	69.6	71.8
2019	Callao	Women	<60	Unaware	89.7	85.5	93.8
2019	Callao	Women	<60	Aware not treated	72.9	70.3	75.6
2019	Callao	Women	<60	Aware treated	74.9	70.7	79.0
2019	Callao	Women	60+	Healthy	68.5	66.3	70.6
2019	Callao	Women	60+	Unaware	78.3	72.9	83.8
2019	Callao	Women	60+	Aware not treated	63.5	57.8	69.1
2019	Callao	Women	60+	Aware treated	72.7	68.6	76.8
2020	Callao	Women	<60	Healthy	72.2	70.9	73.5
2020	Callao	Women	<60	Unaware	87.3	83.4	91.2
2020	Callao	Women	<60	Aware not treated	81.3	76.6	86.1
2020	Callao	Women	<60	Aware treated	82.6	73.0	92.2
2020	Callao	Women	60+	Healthy	68.6	64.9	72.3
2020	Callao	Women	60+	Unaware	81.0	75.8	86.3

2020	Callao	Women	60+	Aware not treated	78.0	65.5	90.5
2020	Callao	Women	60+	Aware treated	77.3	71.6	82.9
2015	Cusco	Men	<60	Healthy	71.8	70.7	73.0
2015	Cusco	Men	<60	Unaware	90.8	84.0	97.6
2015	Cusco	Men	<60	Aware not treated	75.4	69.1	81.7
2015	Cusco	Men	<60	Aware treated	77.5	73.3	81.8
2015	Cusco	Men	60+	Healthy	66.9	64.6	69.3
2015	Cusco	Men	60+	Unaware	78.0	73.7	82.3
2015	Cusco	Men	60+	Aware not treated	81.8	78.6	85.1
2015	Cusco	Men	60+	Aware treated	81.8	73.9	89.7
2016	Cusco	Men	<60	Healthy	72.7	71.5	73.9
2016	Cusco	Men	<60	Unaware	90.0	86.1	93.9
2016	Cusco	Men	<60	Aware not treated	77.1	70.6	83.6
2016	Cusco	Men	<60	Aware treated	69.6	63.1	76.1
2016	Cusco	Men	60+	Healthy	69.2	66.4	72.0
2016	Cusco	Men	60+	Unaware	80.2	76.6	83.8
2016	Cusco	Men	60+	Aware not treated	79.6	74.1	85.0
2016	Cusco	Men	60+	Aware treated	75.1	67.7	82.4
2017	Cusco	Men	<60	Healthy	71.7	70.3	73.1
2017	Cusco	Men	<60	Unaware	87.4	82.5	92.3
2017	Cusco	Men	<60	Aware not treated	78.4	74.7	82.0
2017	Cusco	Men	<60	Aware treated	74.9	67.1	82.6
2017	Cusco	Men	60+	Healthy	68.8	66.3	71.3
2017	Cusco	Men	60+	Unaware	77.5	73.1	82.0
2017	Cusco	Men	60+	Aware not treated	72.6	65.8	79.4
2017	Cusco	Men	60+	Aware treated	86.2	75.2	97.1
2018	Cusco	Men	<60	Healthy	72.8	71.5	74.2
2018	Cusco	Men	<60	Unaware	86.6	83.7	89.5
2018	Cusco	Men	<60	Aware not treated	87.4	81.8	93.0

2018	Cusco	Men	<60	Aware treated	83.0	75.6	90.3
2018	Cusco	Men	60+	Healthy	70.4	67.7	73.0
2018	Cusco	Men	60+	Unaware	89.0	84.1	93.8
2018	Cusco	Men	60+	Aware not treated	75.8	68.5	83.2
2018	Cusco	Men	60+	Aware treated	87.7	75.2	100.3
2019	Cusco	Men	<60	Healthy	73.8	72.5	75.0
2019	Cusco	Men	<60	Unaware	90.2	86.8	93.6
2019	Cusco	Men	<60	Aware not treated	77.0	66.0	88.1
2019	Cusco	Men	<60	Aware treated	77.8	68.7	86.8
2019	Cusco	Men	60+	Healthy	69.5	67.1	71.9
2019	Cusco	Men	60+	Unaware	80.0	74.9	85.1
2019	Cusco	Men	60+	Aware not treated	67.9	61.2	74.7
2019	Cusco	Men	60+	Aware treated	80.4	74.2	86.7
2020	Cusco	Men	<60	Healthy	73.6	72.3	74.9
2020	Cusco	Men	<60	Unaware	88.2	85.7	90.7
2020	Cusco	Men	<60	Aware not treated	82.1	73.9	90.3
2020	Cusco	Men	<60	Aware treated	79.4	66.6	92.2
2020	Cusco	Men	60+	Healthy	70.9	68.7	73.2
2020	Cusco	Men	60+	Unaware	81.6	73.2	89.9
2020	Cusco	Men	60+	Aware treated	88.9	72.1	105.7
2015	Cusco	Women	<60	Healthy	67.9	66.8	69.1
2015	Cusco	Women	<60	Unaware	85.6	76.1	95.1
2015	Cusco	Women	<60	Aware not treated	72.7	67.0	78.4
2015	Cusco	Women	<60	Aware treated	63.8	57.7	69.8
2015	Cusco	Women	60+	Healthy	65.3	63.4	67.2
2015	Cusco	Women	60+	Unaware	83.4	78.1	88.7
2015	Cusco	Women	60+	Aware not treated	69.3	60.5	78.2
2015	Cusco	Women	60+	Aware treated	70.1	65.7	74.6
2016	Cusco	Women	<60	Healthy	68.9	67.7	70.2

2016	Cusco	Women	<60	Unaware	84.0	80.0	88.0
2016	Cusco	Women	<60	Aware not treated	74.3	67.9	80.6
2016	Cusco	Women	<60	Aware treated	76.1	69.7	82.5
2016	Cusco	Women	60+	Healthy	65.5	63.0	67.9
2016	Cusco	Women	60+	Unaware	78.1	74.0	82.3
2016	Cusco	Women	60+	Aware not treated	72.7	66.2	79.1
2016	Cusco	Women	60+	Aware treated	72.8	63.5	82.0
2017	Cusco	Women	<60	Healthy	68.0	66.7	69.3
2017	Cusco	Women	<60	Unaware	82.8	73.2	92.4
2017	Cusco	Women	<60	Aware not treated	74.6	69.3	80.0
2017	Cusco	Women	<60	Aware treated	71.7	62.1	81.2
2017	Cusco	Women	60+	Healthy	64.4	62.7	66.2
2017	Cusco	Women	60+	Unaware	76.2	69.2	83.1
2017	Cusco	Women	60+	Aware not treated	79.5	68.5	90.4
2017	Cusco	Women	60+	Aware treated	72.3	66.5	78.0
2018	Cusco	Women	<60	Healthy	69.6	68.6	70.5
2018	Cusco	Women	<60	Unaware	87.9	84.2	91.5
2018	Cusco	Women	<60	Aware not treated	74.5	70.1	78.8
2018	Cusco	Women	<60	Aware treated	77.4	73.9	80.9
2018	Cusco	Women	60+	Healthy	65.9	63.4	68.3
2018	Cusco	Women	60+	Unaware	85.0	78.0	91.9
2018	Cusco	Women	60+	Aware not treated	72.4	69.2	75.7
2018	Cusco	Women	60+	Aware treated	75.6	70.7	80.6
2019	Cusco	Women	<60	Healthy	69.6	68.4	70.8
2019	Cusco	Women	<60	Unaware	88.3	83.8	92.8
2019	Cusco	Women	<60	Aware not treated	68.4	62.3	74.4
2019	Cusco	Women	<60	Aware treated	68.6	65.1	72.1
2019	Cusco	Women	60+	Healthy	67.4	64.8	69.9
2019	Cusco	Women	60+	Unaware	74.7	69.8	79.5

2019	Cusco	Women	60+	Aware not treated	71.2	64.8	77.6
2019	Cusco	Women	60+	Aware treated	73.0	67.1	78.9
2020	Cusco	Women	<60	Healthy	71.5	70.1	72.8
2020	Cusco	Women	<60	Unaware	95.9	90.7	101.1
2020	Cusco	Women	<60	Aware not treated	76.0	68.5	83.6
2020	Cusco	Women	<60	Aware treated	78.3	69.6	87.0
2020	Cusco	Women	60+	Healthy	68.4	65.9	71.0
2020	Cusco	Women	60+	Unaware	82.5	74.1	90.9
2020	Cusco	Women	60+	Aware not treated	72.1	59.0	85.2
2020	Cusco	Women	60+	Aware treated	77.5	67.8	87.1
2015	Huancavelica	Men	<60	Healthy	70.4	69.2	71.6
2015	Huancavelica	Men	<60	Unaware	85.2	79.2	91.1
2015	Huancavelica	Men	<60	Aware not treated	72.2	64.3	80.0
2015	Huancavelica	Men	<60	Aware treated	76.5	71.3	81.6
2015	Huancavelica	Men	60+	Healthy	69.0	66.8	71.3
2015	Huancavelica	Men	60+	Unaware	80.2	75.9	84.6
2015	Huancavelica	Men	60+	Aware not treated	69.0	55.7	82.4
2015	Huancavelica	Men	60+	Aware treated	71.1	63.4	78.8
2016	Huancavelica	Men	<60	Healthy	70.4	69.0	71.7
2016	Huancavelica	Men	<60	Unaware	85.2	79.8	90.5
2016	Huancavelica	Men	<60	Aware not treated	75.3	71.7	79.0
2016	Huancavelica	Men	<60	Aware treated	75.5	63.4	87.6
2016	Huancavelica	Men	60+	Healthy	67.1	64.6	69.6
2016	Huancavelica	Men	60+	Unaware	80.2	76.1	84.3
2016	Huancavelica	Men	60+	Aware not treated	70.0	63.2	76.8
2016	Huancavelica	Men	60+	Aware treated	78.0	68.2	87.8
2017	Huancavelica	Men	<60	Healthy	70.0	69.0	71.1
2017	Huancavelica	Men	<60	Unaware	86.7	82.9	90.5
2017	Huancavelica	Men	<60	Aware not treated	67.9	63.9	72.0

2017	Huancavelica	Men	<60	Aware treated	77.2	67.3	87.1
2017	Huancavelica	Men	60+	Healthy	68.5	66.2	70.7
2017	Huancavelica	Men	60+	Unaware	79.2	75.5	82.9
2017	Huancavelica	Men	60+	Aware not treated	68.4	63.1	73.7
2017	Huancavelica	Men	60+	Aware treated	80.3	76.3	84.3
2018	Huancavelica	Men	<60	Healthy	71.2	69.5	72.9
2018	Huancavelica	Men	<60	Unaware	87.7	83.1	92.3
2018	Huancavelica	Men	<60	Aware not treated	80.9	74.6	87.2
2018	Huancavelica	Men	<60	Aware treated	69.7	62.9	76.6
2018	Huancavelica	Men	60+	Healthy	66.7	64.6	68.8
2018	Huancavelica	Men	60+	Unaware	79.6	75.3	83.9
2018	Huancavelica	Men	60+	Aware not treated	66.9	62.8	70.9
2018	Huancavelica	Men	60+	Aware treated	75.6	68.7	82.6
2019	Huancavelica	Men	<60	Healthy	69.6	68.2	71.0
2019	Huancavelica	Men	<60	Unaware	90.6	83.6	97.6
2019	Huancavelica	Men	<60	Aware not treated	72.2	66.9	77.5
2019	Huancavelica	Men	<60	Aware treated	76.5	70.5	82.5
2019	Huancavelica	Men	60+	Healthy	68.5	66.1	71.0
2019	Huancavelica	Men	60+	Unaware	79.1	75.7	82.6
2019	Huancavelica	Men	60+	Aware not treated	78.3	72.7	83.9
2019	Huancavelica	Men	60+	Aware treated	76.9	71.4	82.4
2020	Huancavelica	Men	<60	Healthy	70.1	68.8	71.4
2020	Huancavelica	Men	<60	Unaware	83.5	78.5	88.6
2020	Huancavelica	Men	<60	Aware not treated	76.8	68.7	85.0
2020	Huancavelica	Men	<60	Aware treated	76.3	75.2	77.3
2020	Huancavelica	Men	60+	Healthy	67.7	64.3	71.0
2020	Huancavelica	Men	60+	Unaware	77.0	73.4	80.6
2020	Huancavelica	Men	60+	Aware not treated	84.5	69.6	99.4
2020	Huancavelica	Men	60+	Aware treated	82.0	74.8	89.2

2015	Huancavelica	Women	<60	Healthy	67.8	66.7	68.9
2015	Huancavelica	Women	<60	Unaware	84.1	78.3	89.9
2015	Huancavelica	Women	<60	Aware not treated	71.4	66.4	76.5
2015	Huancavelica	Women	<60	Aware treated	72.3	66.1	78.5
2015	Huancavelica	Women	60+	Healthy	66.2	64.3	68.1
2015	Huancavelica	Women	60+	Unaware	77.0	72.5	81.5
2015	Huancavelica	Women	60+	Aware not treated	69.9	66.6	73.1
2015	Huancavelica	Women	60+	Aware treated	69.6	63.3	75.9
2016	Huancavelica	Women	<60	Healthy	66.4	65.3	67.5
2016	Huancavelica	Women	<60	Unaware	90.1	84.6	95.6
2016	Huancavelica	Women	<60	Aware not treated	70.4	67.1	73.7
2016	Huancavelica	Women	<60	Aware treated	71.0	64.9	77.1
2016	Huancavelica	Women	60+	Healthy	63.7	61.8	65.6
2016	Huancavelica	Women	60+	Unaware	78.2	72.9	83.5
2016	Huancavelica	Women	60+	Aware not treated	71.5	61.6	81.4
2016	Huancavelica	Women	60+	Aware treated	73.3	67.2	79.3
2017	Huancavelica	Women	<60	Healthy	66.4	65.5	67.3
2017	Huancavelica	Women	<60	Unaware	87.5	81.4	93.7
2017	Huancavelica	Women	<60	Aware not treated	71.0	67.6	74.4
2017	Huancavelica	Women	<60	Aware treated	70.6	61.1	80.1
2017	Huancavelica	Women	60+	Healthy	64.1	62.3	66.0
2017	Huancavelica	Women	60+	Unaware	77.6	74.1	81.2
2017	Huancavelica	Women	60+	Aware not treated	62.6	57.4	67.7
2017	Huancavelica	Women	60+	Aware treated	77.8	73.7	81.9
2018	Huancavelica	Women	<60	Healthy	67.6	66.6	68.7
2018	Huancavelica	Women	<60	Unaware	82.1	76.1	88.2
2018	Huancavelica	Women	<60	Aware not treated	69.6	65.6	73.7
2018	Huancavelica	Women	<60	Aware treated	75.4	68.9	81.8
2018	Huancavelica	Women	60+	Healthy	65.9	63.9	67.9

2018	Huancavelica	Women	60+	Unaware	75.5	71.7	79.3
2018	Huancavelica	Women	60+	Aware not treated	69.3	64.8	73.9
2018	Huancavelica	Women	60+	Aware treated	71.7	66.0	77.4
2019	Huancavelica	Women	<60	Healthy	67.6	66.5	68.8
2019	Huancavelica	Women	<60	Unaware	91.3	85.4	97.2
2019	Huancavelica	Women	<60	Aware not treated	73.9	67.0	80.9
2019	Huancavelica	Women	<60	Aware treated	71.3	67.4	75.2
2019	Huancavelica	Women	60+	Healthy	64.8	63.1	66.5
2019	Huancavelica	Women	60+	Unaware	77.0	72.2	81.9
2019	Huancavelica	Women	60+	Aware not treated	70.6	65.3	75.9
2019	Huancavelica	Women	60+	Aware treated	75.5	70.3	80.7
2020	Huancavelica	Women	<60	Healthy	68.2	67.0	69.3
2020	Huancavelica	Women	<60	Unaware	85.0	83.0	86.9
2020	Huancavelica	Women	<60	Aware not treated	76.1	67.5	84.6
2020	Huancavelica	Women	<60	Aware treated	78.4	69.3	87.4
2020	Huancavelica	Women	60+	Healthy	65.1	62.7	67.5
2020	Huancavelica	Women	60+	Unaware	72.8	66.8	78.9
2020	Huancavelica	Women	60+	Aware not treated	75.1	69.4	80.9
2020	Huancavelica	Women	60+	Aware treated	73.1	67.5	78.7

Supplementary Table 6. Official documents from each region in Peru addressing hypertension and brief discussion of these

We sought for policies, plans, reports and other official documents for each region (25 regions) in Peru. These documents were herein summarised and briefly discussed to complement the associated paragraph in the main manuscript.

Region	Discussion
Amazonas	A 2018 inform by the Regional Government suggested that a limitation to *give treatment to people with hypertension was the lack of financial and human resources* (https://bit.ly/2R5kY55).<br><br>This could explain why the mean SBP was, at some time points, higher in the untreated or treated groups than that of the unaware group. Close follow-up of people with hypertension to either secure the receive treatment or they take the treatment as prescribed, could be suggested.
Ancash	In a 2017 inform by the Regional Health Office, amongst the main recommendations there was *to monitor the treatment and control of people with hypertension* (https://bit.ly/3i1jTXa).<br><br>This could explain why, at some given time points, the mean SBP was not in the unaware group, but in the untreated or treated group. Close follow-up of people with hypertension to either secure the receive treatment or they take the treatment as prescribed, could be suggested.
Apurimac	
Arequipa	In 2013 the Regional Government of Arequipa issued an operations plan (https://bit.ly/2SId5Ty); in this document they set targets, strategies and budget. For example, one specific aim in this document was to *develop guidelines for non-communicable diseases* (including hypertension); other was *to prioritize the interventions to prevent non-communicable diseases* (including hypertension).<br><br>In Arequipa the mean SBP was mostly highest in the unaware group. This could suggest that the commitment with the prevention of non-communicable diseases has not been successful (though to the best of our knowledge this has not been formally quantified). Future strategies need to better implemented to improve early diagnosis.
Ayacucho	
Cajamarca	
Callao	
Cusco	A progress evaluation of the first semester in Cusco, they claimed that despite the COVID-19 pandemic they managed to deliver telemedicine consultations to people with hypertension and diabetes (https://bit.ly/3fAA3Fb).

	Except for two time points (men, 60+ years), in Cusco the mean SBP was always the highest in the unaware group. The fact that they managed to conduct telemedicine for these patients could suggest that they already had an adequate program to care for these patients.
Huancavelica	
Huanuco	
Ica	
Junin	
La Libertad	The regional strategic plan for the period 2020-2023 proposed a specific indicator for hypertension (and diabetes): *percentage of adults with diagnosis and treatment of hypertension (and diabetes)* (https://bit.ly/3wlNJDV).<br><br>Our results could further inform this indicator by showing that improving treatment is urgent, followed by improving treatment allocation in men and improving treatment efficacy in women. This sex difference, that is also observed in other regions, could be explained by: i) men less often attend the healthcare system, hence the worse mean SBP in the untreated men; ii) although women could more often attend the healthcare system, the worse mean SBP in the treated group could be explained by poor adherence, poor follow-up to secure optimal treatment, or poor diseases consciousness.
Lambayeque	The institutional operational plan 2020 has an indicator to timely identify the risk for non-communicable diseases (percentage of people aged 15+ with hypertension) (https://bit.ly/3g13Iql).<br><br>Except for the untreated group in some years, the unaware group had always the worst mean SBP. Consequently, their plan to improve hypertension diagnosis should be a priority and must be carefully thought to observe promising results.
Lima	
Loreto	
Madre de Dios	
Moquegua	The regional strategic plan 2018-2020 has an indicator about hypertension diagnosis, with the aim to increase the awareness (self-reported diagnosis) (https://bit.ly/3uv2Qzm).<br><br>This would appear to be an adequate response to our findings whereby the unaware group had the worst mean SBP.
Pasco	
Piura	
Puno	
San Martin	A 2019 progress report described two indicators addressing hypertension: inform and increase consciousness about hypertension; and improve treatment for patients with hypertension (https://bit.ly/3i56pJZ).<br><br>The need to improve hypertension diagnosis is urgent based on our findings, which also call to secure effective treatment.
Tacna	
Tumbes	In 2017 a progress report had an indicator to *strengthen all action that contribute to the reduction of morbidity related with hypertension (diabetes, dyslipidaemia and obesity)* (https://bit.ly/3c6BxF1).

	The mean SBP was always the worst in the unaware group. We could argue that this indicator concerns only to those with hypertension and to reduce the complications that follow hypertension. Our results could suggest that this would not be enough, and improve diagnosis is urgent.
Ucayali	

SBP: systolic blood pressure. For some regions we could not find any specific information, these cells were left in the table for completeness.

Supplementary Figure 1. Mean diastolic blood pressure by population groups stratified by sex, age group and year

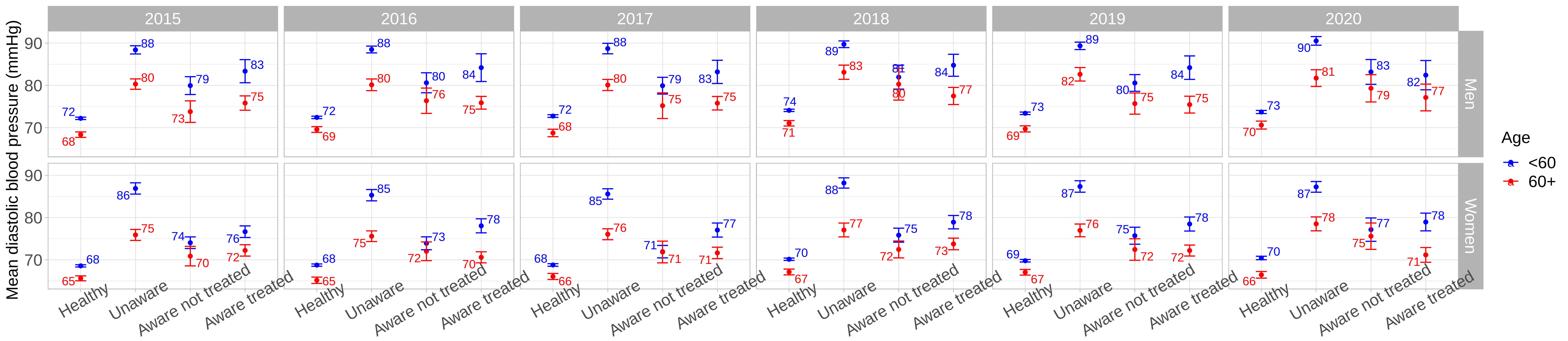

2015
2016
2017
2018
2019
2020
Men
Women
Mean diastolic blood pressure (mmHg)
Healthy
Unaware
Aware not treated
Aware treated
Age
<60
60+

Supplementary Figure 2. Mean systolic blood pressure (95% confidence interval) by population group, across regions and study years in men

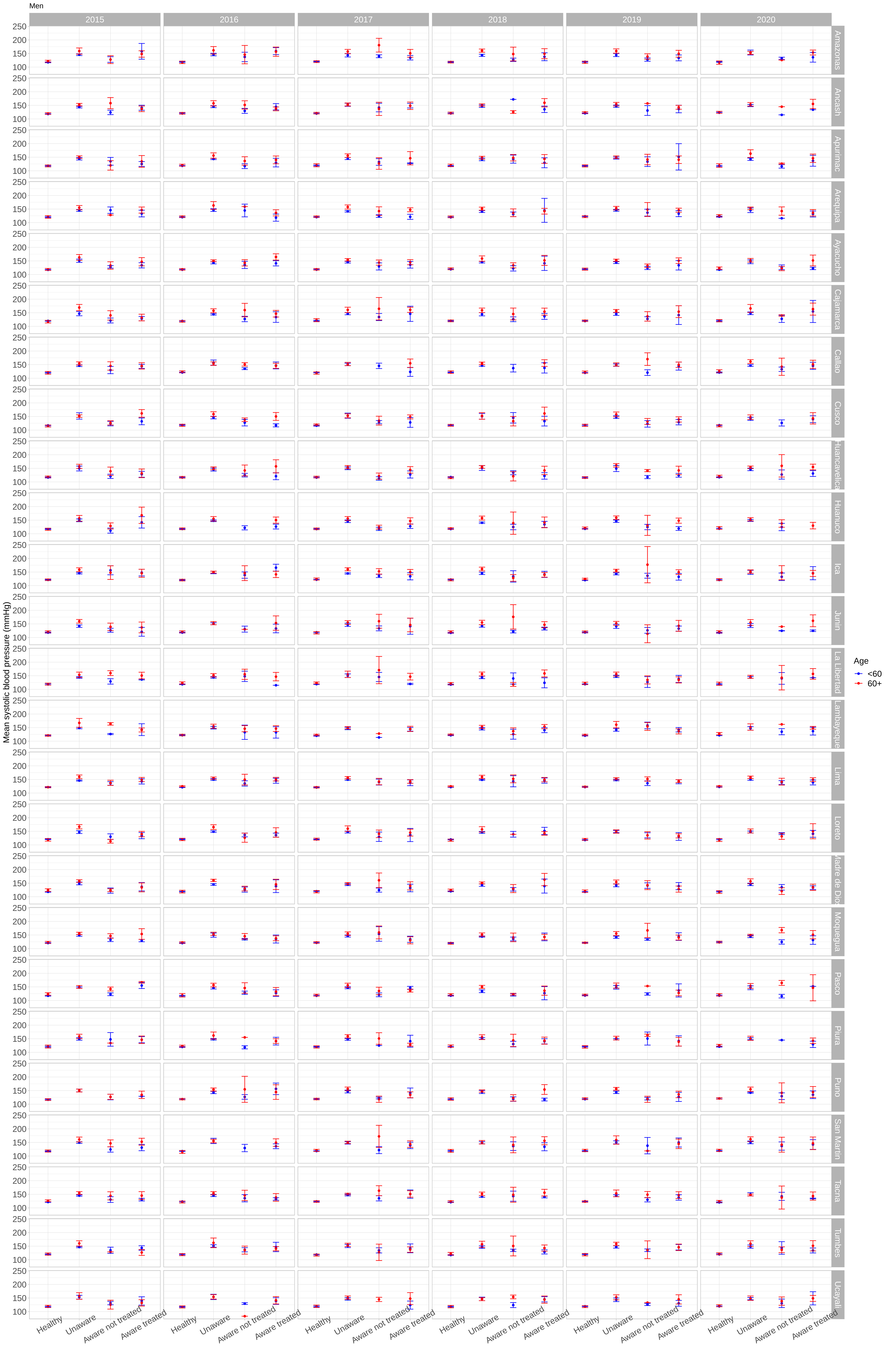
Men
2015
2016
2017
2018
2019
2020
Amazonas
Ancash
Apurimac
Arequipa
Ayacucho
Cajamarca
Callao
Cusco
Huancavelica
Huanuco
Ica
Junin
La Libertad
Lambayeque
Lima
Loreto
Madre de Dios
Moquegua
Pasco
Piura
Puno
San Martin
Tacna
Tumbes
Ucayali
Mean systolic blood pressure (mmHg)
250
200
150
100
Healthy
Unaware
Aware not treated
Aware treated
Age
<60
60+

Supplementary Figure 3. Mean systolic blood pressure (95% confidence interval) by population group, across regions and study years in women

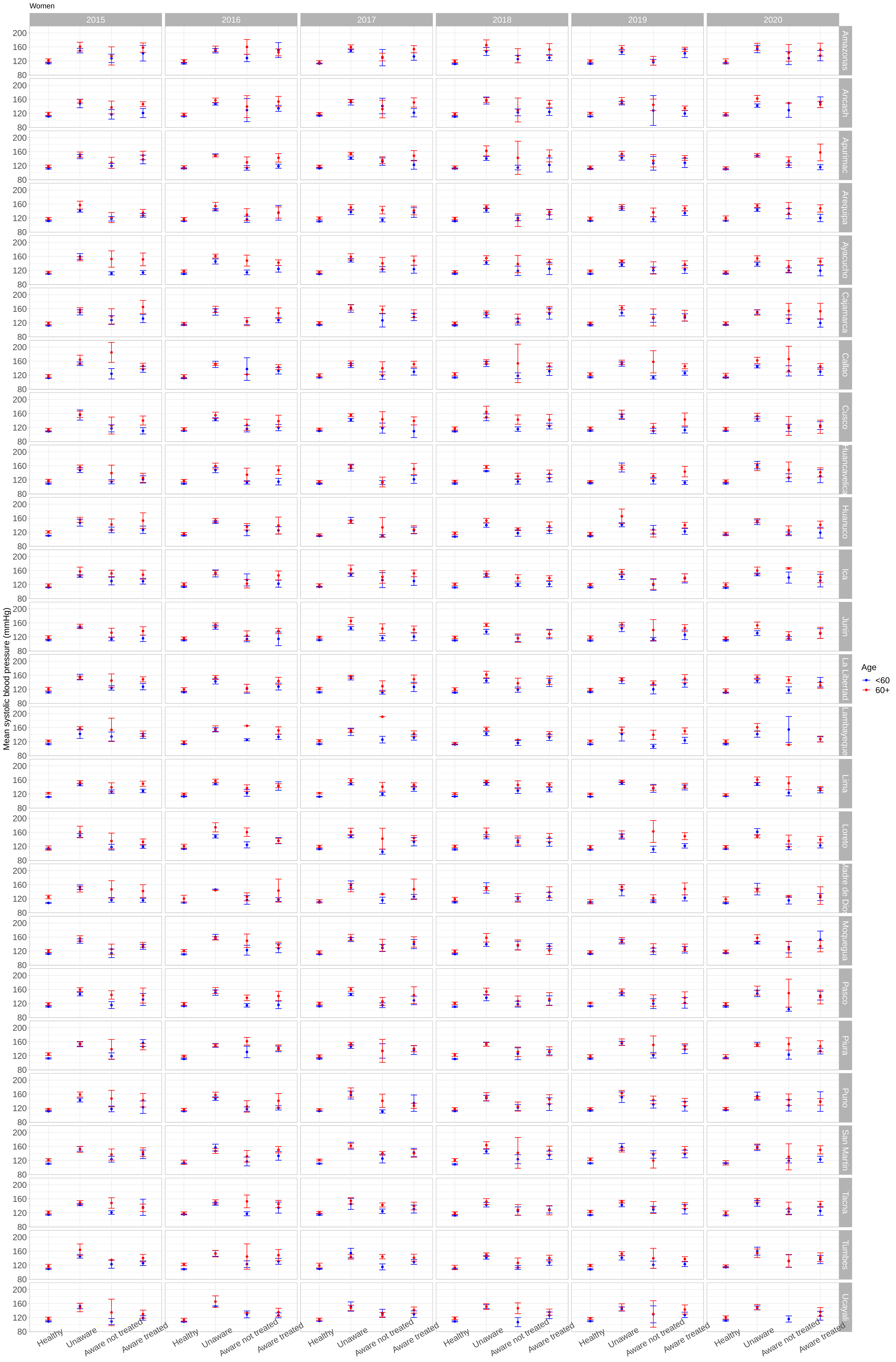

Women
2015
2016
2017
2018
2019
2020
Amazonas
Ancash
Apurimac
Arequipa
Ayacucho
Cajamarca
Callao
Cusco
Huancavelica
Huanuco
Ica
Junin
La Libertad
Lambayeque
Lima
Loreto
Madre de Dios
Moquegua
Pasco
Piura
Puno
San Martin
Tacna
Tumbes
Ucayali
Mean systolic blood pressure (mmHg)
Healthy
Unaware
Aware not treated
Aware treated
Age
<60
60+

Supplementary Figure 4. Mean diastolic blood pressure (95% confidence interval) by population group, across regions and study years in men

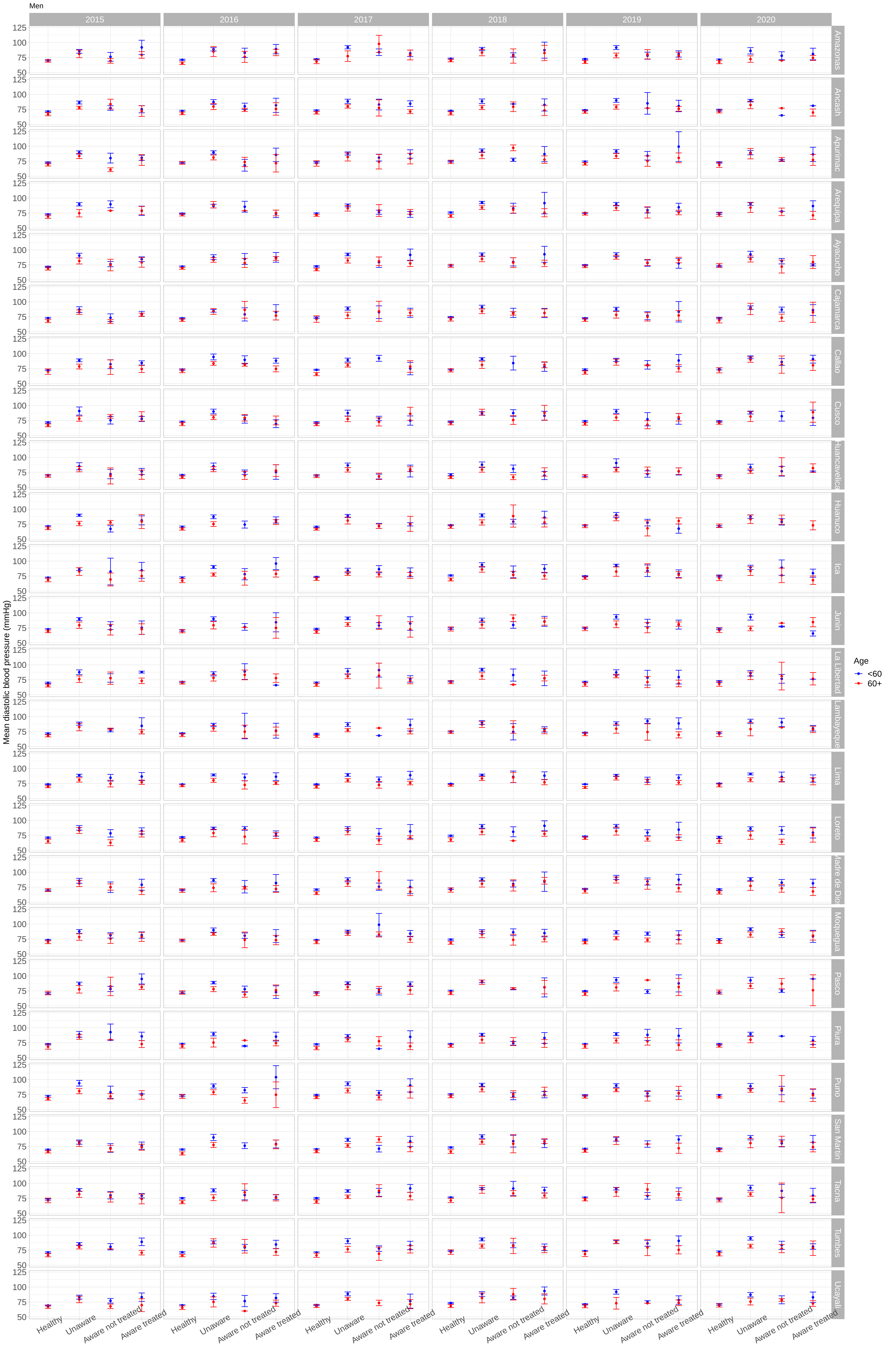

Men
2015
2016
2017
2018
2019
2020
Amazonas
Ancash
Apurimac
Arequipa
Ayacucho
Cajamarca
Callao
Cusco
Huancavelica
Huanuco
Ica
Junin
La Libertad
Lambayeque
Lima
Loreto
Madre de Dios
Moquegua
Pasco
Piura
Puno
San Martin
Tacna
Tumbes
Ucayali
Mean diastolic blood pressure (mmHg)
Healthy
Unaware
Aware not treated
Aware treated
Age
<60
60+

Supplementary Figure 5. Mean diastolic blood pressure (95% confidence interval) by population group, across regions and study years in women

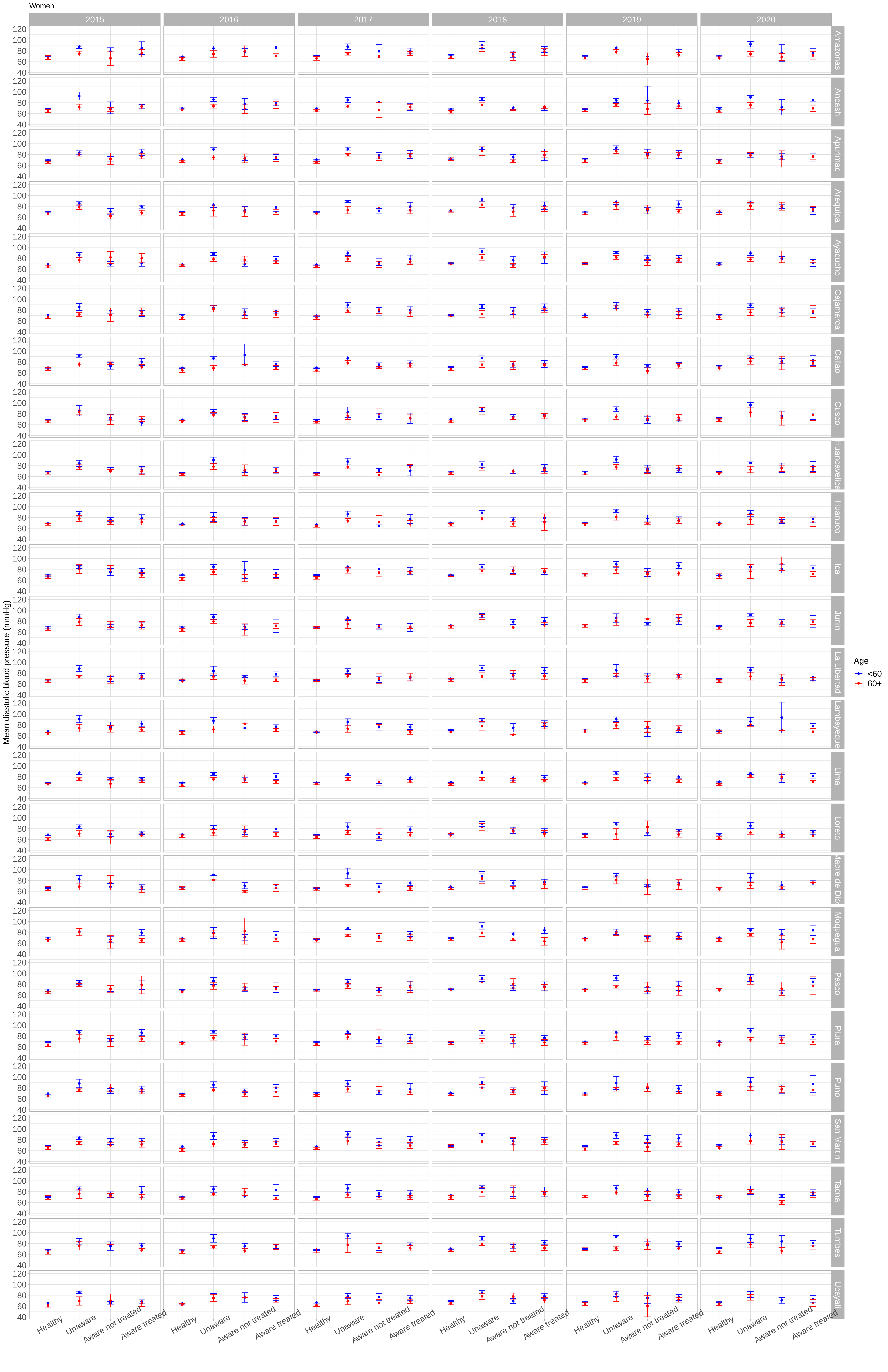

Women
2015
2016
2017
2018
2019
2020
Amazonas
Ancash
Apurimac
Arequipa
Ayacucho
Cajamarca
Callao
Cusco
Huancavelica
Huanuco
Ica
Junin
La Libertad
Lambayeque
Lima
Loreto
Madre de Dios
Moquegua
Pasco
Piura
Puno
San Martin
Tacna
Tumbes
Ucayali
Mean diastolic blood pressure (mmHg)
Healthy
Unaware
Aware not treated
Aware treated
Age
<60
60+
